# Tribenzo[*b*,*d*,*f*]azepines as Minimalistic Purely Organic Photocatalysts

**DOI:** 10.1021/acs.orglett.6c03716

**Published:** 2026-10-25

**Authors:** Gabriele Pozza, Gustavo Mondragón-Solórzano, Simone Baldon, Jorge Humbrías-Martín, Jonas Žurauskas, Elizaveta Meshcheriakova, Luca Dell’Amico, Katy Medrano-Uribe

**Affiliations:** † Department of Chemical Sciences, University of Padova, Via Francesco Marzolo 1, 35131 Padova, Italy; ‡ Department of Chemistry and Chemical Biology, Northeastern University, Boston, Massachusetts 02115, United States; § Department of Organic Chemistry, Vilnius University, Naugarduko, LT-03225 Vilnius, Lithuania; ∥ Chemistry Department, Lomonosov Moscow State University, Leninskie Gory 1/3, 119991 Moscow, Russia

## Abstract

Few examples of organic photocatalysts (PCs) can be used in singlet electron transfer (SET) and energy transfer (EnT) processes and generally include relatively complex scaffolds based on transition-metal complexes or IPN-based TADF emitters. In this work, we introduce a family of minimalist bifunctional organic photocatalysts based on tribenzo­[*b*,*d*,*f*]­azepine that can promote both activation types. The study shows that, for SET, the stability of radical-ion PC intermediates is crucial. In contrast, a slow intersystem crossing increases the level of triplet formation, thereby enhancing EnT reactivity.

## Graphical abstract

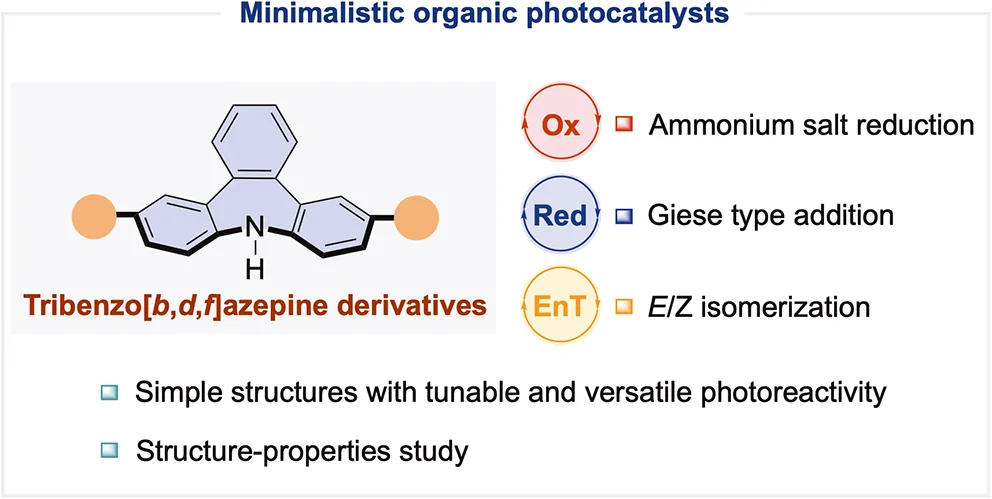

In recent years, we have observed an increase in the use of organic molecules as photocatalysts (PCs), replacing metal-based ones and facilitating the development of greener and more sustainable reaction conditions.^1^ In this regard, the reactivity of PCs is often focused on achieving single-electron transfer (SET) or energy transfer (EnT) pathways (Figure 1a), but it is difficult to find examples in the literature of a PC that can operate via both mechanisms equally well. A few donor–acceptor (D-A) molecules originally designed for materials chemistry have been identified as powerful candidates for mediating the aforementioned photocatalytic processes.^2^ Nevertheless, those D-A compounds are characterized by complex substitution patterns in the main core. Examples include the widely known 4CzIPN^3^ and other sulfur-based D-A photocatalysts^4^ that are capable of undergoing oxidative and reductive quenching cycles, while the EnT mechanism is unblocked by facilitated intersystem crossing (ISC).

**Figure 1:**
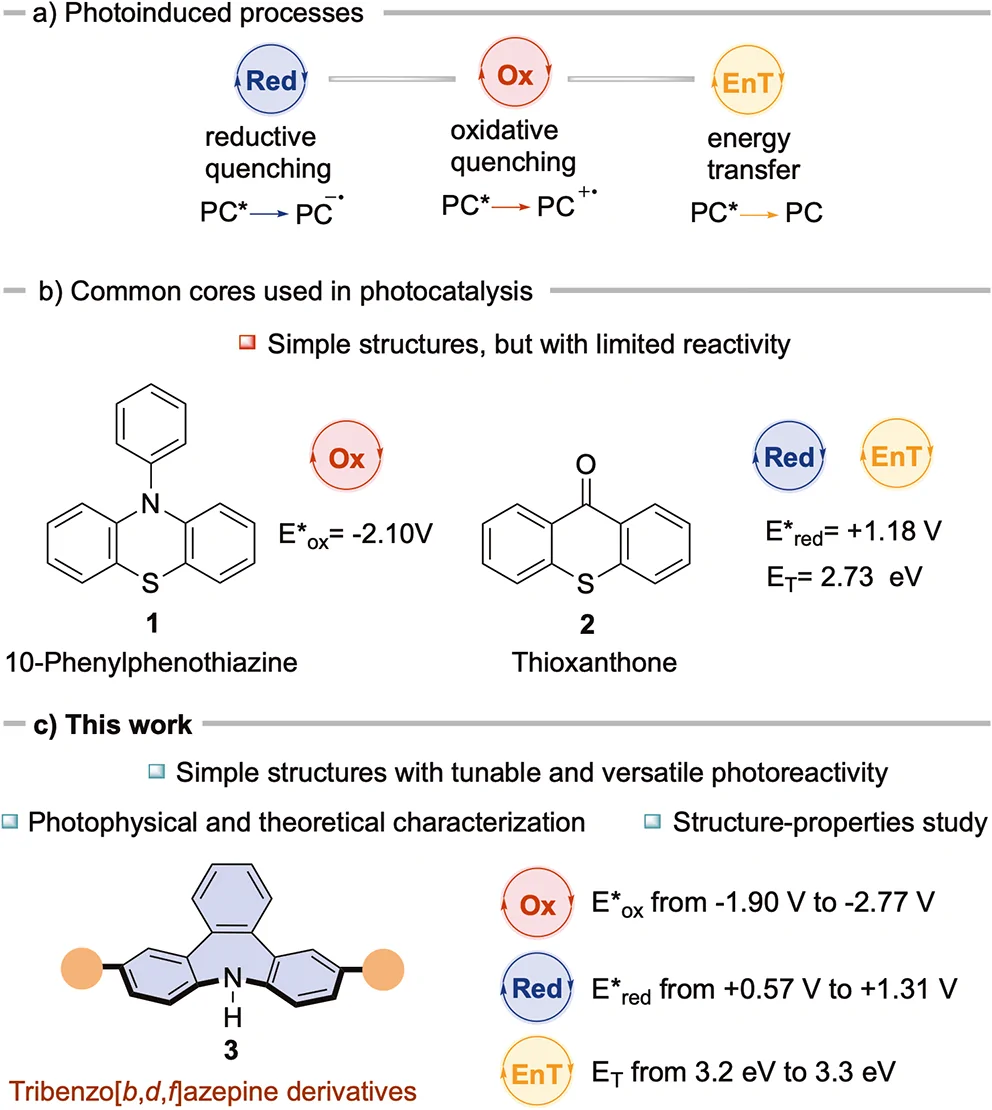
a) Traditional photoinduced processes. b) Common photocatalyst based on simple structures. c) Our approach for versatile minimalistic photocatalysts.

In materials chemistry, azepine-containing frameworks are valued for their excited-state properties. Incorporating its derivative tribenzo­[*b*,*d*,*f*]­azepine (TBA, **3**) into optoelectronic materials enhances luminescence through its distorted geometry.^5^ TBA integration into multiple-resonance emitters creates twisted structures that reduce aggregation and enable reverse intersystem crossing. Azepine engineering allows control over emission energy, singlet–triplet gaps, and spin dynamics, producing efficient deep-blue OLEDs with high purity and stability.^6^ Both scaffolds serve as tunable platforms for excited-state control. Inspired by their possible transition from material to photochemical applications, we used TBA as a donor in sulfur-based D-A photocatalysts, achieving high reaction yields.^7^ Motivated by these results, we further explored TBA as the main core in developing new minimalistic photocatalysts with unique excited-state features.

Here, we report the synthesis of a new family of TBA-based molecules that, upon excitation, can catalyze oxidative and reductive quenching cycles as well as energy transfer processes.

The structural design demonstrated reactive stability via intermediates PC^•+^ and PC^•–^ for the SET manifold. EnT activation occurs through a slow ISC linked to the mixed nature of the singlet state involved and small SOC values. Unlike simple organic photocatalysts like **1** and **2**, which have limited reactivity (Figure 1b), our minimalistic design retains the versatility of complex D-A systems, while offering the advantages of a simpler and more accessible synthesis (Figure 1c).

The synthesis of tribenzo­[*b*,*d*,*f*]­azepine has been previously reported, with authors exploring derivatization methods.^8^ However, using dibrominated TBA as the key precursor, we obtained the arylated tribenzo­[*b*,*d*,*f*]­azepines via Suzuki coupling (36–62% yields, see the Supporting Information (SI)).

In TBA (**3**), its antiaromatic nature stems from a 16π ring (dibenzoazepine, DBA), which according to Baird’s rule, is a 4n compound that stabilizes in the excited state due to planarity.^9^ The fused aromatic ring C in C7 and C8 (see **3** in Figure 2) balances this effect because it is aromatic in the ground state (6π ring, following Huckel’s rule). In other words, in the excited state, the 16π core will tend to adopt a planar conformation, gaining stabilization and increasing the delocalization of the shared double bond of the fused aromatic ring C into the main core, while the latter is destabilized.^10^ This duality prompted us to study TBA derivatives in both states and their potential impact on photoreactivity.

**Figure 2:**
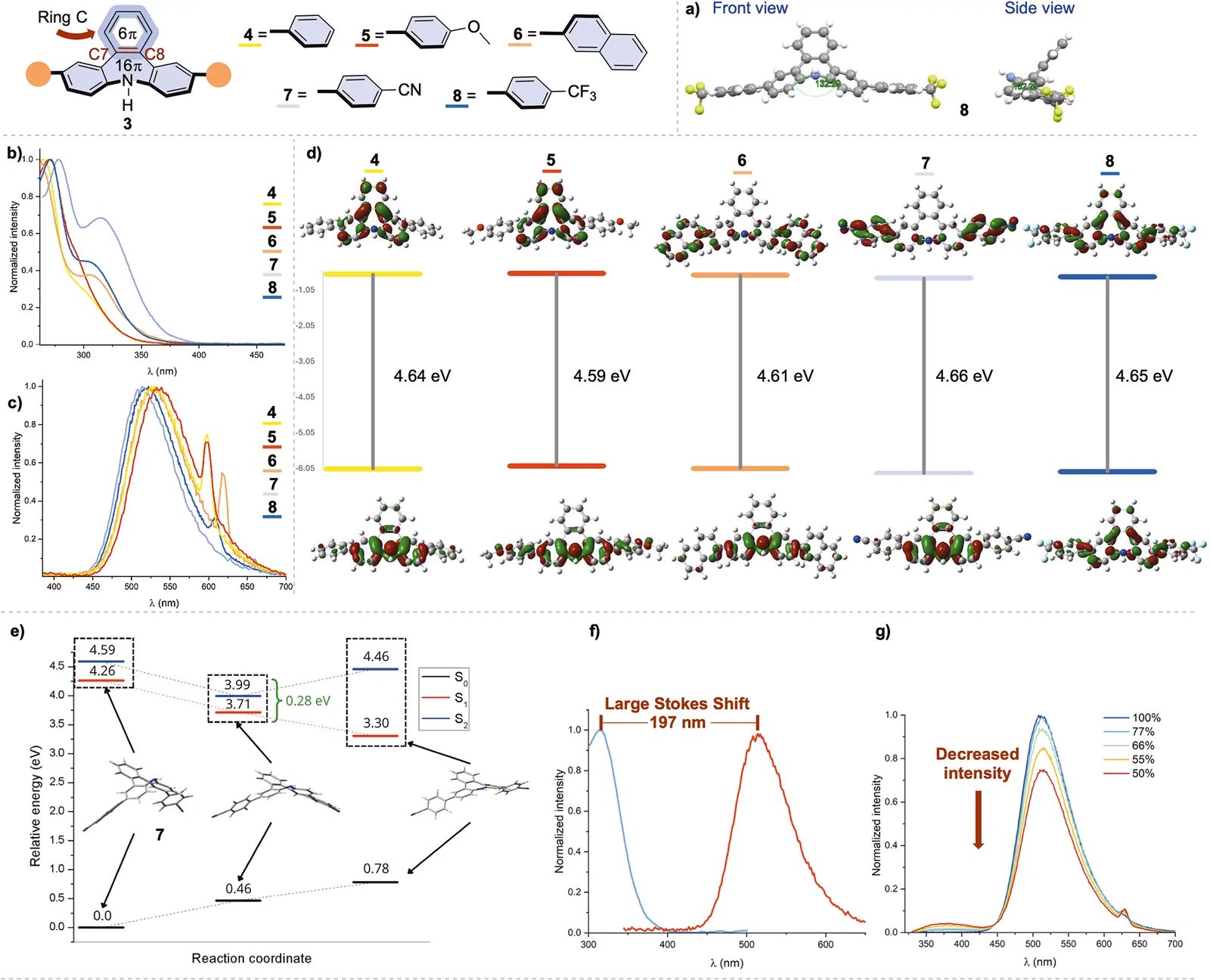
a) Crystalline structure of **8**, showing the bent conformation. b) and c) Absorption and emission spectra measured at room temperature in MeCN. d) Selective frontier MOs for compounds **4**–**8**. e) Representative example of the photophysical pathway in **7**. f) Large Stokes shift of **7**, as an example of the general behavior of TBA-based PCs. g) MeOH–glycerol experiment performed using **7** to study the bent-to-planar behavior.

For the optical measurements in MeCN performed at room temperature, absorption revealed a new band above 320 nm for molecules **6**, **7**, and **8**. The bathochromic shift in molecule **7** was significant, likely due to a “push–pull” effect from the electron-withdrawing CN group, differing from CF_3_ in molecule **8** by resonance effects (Figure 2b). Fluorescence measurements showed only minor λemm differences among all PCs (Figure 2c).

In HOMO–LUMO analysis of compounds **4**–**8**, HOMO delocalizes along the molecule with minimal contribution from ring C, due to its different plane position (see X-ray side view, Figure 2a). LUMO variation is greater in compounds **4** and **5**, mainly along the TBA core, while in **6**–**8**, substituents contribute more (Figure 2d). The HOMO–LUMO gap remains nearly constant, from 4.59 to 4.66 eV, lowest in compound **5** due to the electron-donating OMe group. The slight gap variation aligns with minimal charge transfer observed in solvatochromism (see SI), supported by CT and Δr calculations (Table S14).

DBA is reported as the nitrogen analog of oxepine, exhibiting a large Stokes shift due to photoinduced structural planarization (PISP).^11^ Both compounds undergo significant electronic and conformational changes in their excited states. To understand structural changes in our TBAs with ring C, we proposed a photophysical pathway in molecule **7** using excited-state geometry optimizations of S_1_ and S_2_. As shown in Figure 2e, a small energy gap (0.28 eV) between S_1_ and S_2_ facilitates internal conversion to a quasi-planar structure with red-shifted emission (491 nm). This aligns with our experiments, where the bent conformation decreases conjugation, causing a hypsochromic shift in absorption (264 to 315 nm, Table 1), while excitation leads to a quasi-planar conformation and red-shifted emission (512 to 534 nm, Table 1). Like DBA and oxepine, our photocatalysts show a large Stokes shift (197 to 264 nm) despite the hindrance of complete planarization by ring C, as shown in compound **7** (Figure 2f).

**Table 1:** Summary of Photoredox Properties^a^

PC	λ_max,abs_ (nm)	λ_max,em_ (nm)	E_0,0_ exp (eV)	E_0,0_ calc (eV)	Stokes Shift (nm)	E_ox_ ^d^ (V)	E_red_ ^d^ (V)	E*_ox_ (V)	E*_red_ (V)	E_T_ exp (eV)	E_T_ calc (eV)
**4**	264	527	3.8^b^	3.30	263	0.68	–^e^	–2.62	–	3.3	3.34
**5**	270	534	3.8^b^	3.29	264	0.89	–^e^	–2.40	–	3.2	2.97
**6**	304	527	2.9	3.29	223	0.66	–2.33	–2.24	0.57	3.3	3.33
**7**	315	512	3.0	3.31	197	1.10	–2.00	–1.90	1.0	3.3	3.37
**8**	303	520	3.5	---^c^	218	0.73	–2.19	–2.77	1.31	3.3	3.45

a MeCN was used for all of the characterization performed at room temperature. MeTHF was used for triplet-state characterization performed at 77 K.

b Using the tangent approximation.

c Not determined because of an imaginary frequency.

d For CV measurements, potentials are reported V vs SCE. Supporting electrolyte: 0.1 M [Bu_4_N]­[PF_6_] solution. The scan rate for each compound is given in the SI.

e No presence of a reduction peak using MeCN as solvent.

Moreover, because of this large Stokes shift, it was sometimes difficult to identify the intersection between absorption and emission for the E_0,0_ value in some TBA derivatives. We calculated the experimental value using the tangent approximation^12^ and confirmed it with the Franck–Condon–Herzberg–Teller (FCHT) theoretical calculation. The theoretical values aligned with the optical data; for example, for molecule **6**, the E_0,0_ value is 2.9 eV experimentally and 3.29 eV theoretically. To prevent overestimating the redox potential in the excited state, we used the lowest values from both approximations (Table 1).

To confirm conformational change in the excited state, we performed viscosity experiments with methanol–glycerol mixtures to see whether restricted movement changes fluorescence. All molecules (**4**–**8**) showed a continuous decrease in the band, regardless of EDG or EWG presence. For example, molecule **7**’s 511 nm band decreased sharply, as seen in Figure 2g. Raman signals from glycerol were also observed in all measurements (see SI).

Triplet-state lifetimes and energies were determined from emission studies at 77 K and corroborated by TD-DFT calculations using the Tamm–Dancoff approximation at the ωB97X-D/Def2-TZVP level. Phosphorescence analysis suggests values of 3.2 to 3.3 eV, beneficial for compounds **4**–**8** in energy transfer manifolds. TD-DFT associates experimental E_T_ with emission from a higher triplet state in some PCs;^13^ e.g., for molecule **7**, T_3_ is 3.37 eV, close to the experimental E_T_ of 3.3 eV. For **4**, the most reliable value relates to E_T_ from T_2_. The proximity of energy levels and similar electronic transitions from T_1_-T_3_ make it difficult to identify a main contribution (Table S15). A slow ISC via the El-Sayed rule, from S_n_ (π­(n)−π*) to T_n_ (π–π*), facilitates the process in all PCs. The ISC was supported by calculating SOC constants (see SI, section H).

Regarding the cyclic voltammetry (CV) performed for all the TBA derivatives, we found that compounds **4** and **5** did not present a clear reduction peak using acetonitrile (MeCN) as the solvent. This observation can be explained by the limited redox window provided by the MeCN, or it can also be attributed to a less favorable formation of a less stable radical anion (PC^•–^) in the absence of an electron-withdrawing group, while a clear E_ox_ value was observed for all the PC candidates.

The photocatalytic reactivity and versatility of TBA derivatives were tested using the three main mechanisms in organic synthesis: oxidative quenching, reductive quenching, and energy transfer. Substrates were selected based on redox potential and E_T_ values to evaluate **4**–**8**. Their performance was compared with **1** and **2**, which, like our PCs, have a simple structure (Figure 1).

We first studied the photoreactivity toward reducing ammonium salt **9** (E_red_ = −1.74 V) via an oxidative quenching cycle. **9** can block an alternative XAT mechanism,^14^ often associated with aryl halide reduction, and is a challenging transformation typically performed under photoelectrochemical or multiphoton conditions.^15^ After 24 h of irradiation, we observed formation of molecule **10**. The PCs (**4**–**8**) showed consistent performance (16–49% NMR yield) regardless of substituents, indicating their reductant ability depends on forming the PC^•+^ more than the electronic nature of attached groups (EDG or EWG). This aligns with electrochemical data, where each TBA- derivative showed a clear oxidation peak.

As we expected, phenothiazine (**1**) was able to perform the reaction in a 49% NMR yield, similar to the best performance obtained with our PC **4**. PC **2** remained unreacted, as it is known to be a better oxidant and photosensitizer than a reductant (Table 2).

**Table 2:**
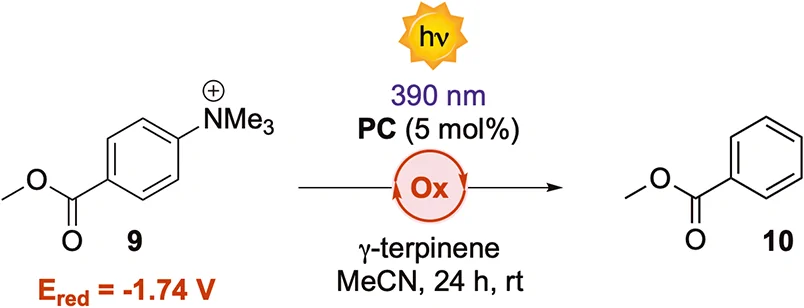
Reduction of Ammonium Salt **9**

Entry	PC	^1^H NMR Yield^a^ (%)
1	**4**	49
2	**5**	34
3	**6**	43
4	**7**	16
5	**8**	32
6	**1**	49
7	**2**	N.R.

a CH_2_Br_2_ was used as an internal standard. N.R. = no reaction.

We next investigated the possibility of using the TBA-based photocatalyst in a reductive quenching employing the decarboxylative addition of N-Cbz-Pro (**11**) to dimethyl maleate (**12**).^4,7,16^ In this mechanism, the PC must be able to oxidize the N-Cbz-Pro carboxylate (E_ox_ = 0.95 V). In this case, we observed no conversion of the starting material when using compounds **4**, **5**, and **6**, probably related to the limited possibility of achieving this type of transformation involving the generation of PC^•–^, according to the cyclic voltammetry study, and considering the highest LUMO values related to the other members of the family (Table S13).

In the reductive quenching manifold, the electronic characteristics of the substituents significantly influence reactivity, especially with molecules **7** and **8**, which have strong EWGs and E*_red_ values of +1.00 and +1.31 V, respectively. The latter was demonstrated to be the most effective PC, yielding product **13** in a 70% yield. In this case, the reductant phenothiazine (**1**) could not conduct the oxidation of the carboxylate form of compound 11. In contrast, PC **2** catalyzed the reaction efficiently, giving molecule **13** in 77% yield, in line with a previous report^17^ (Table 3).

**Table 3:**
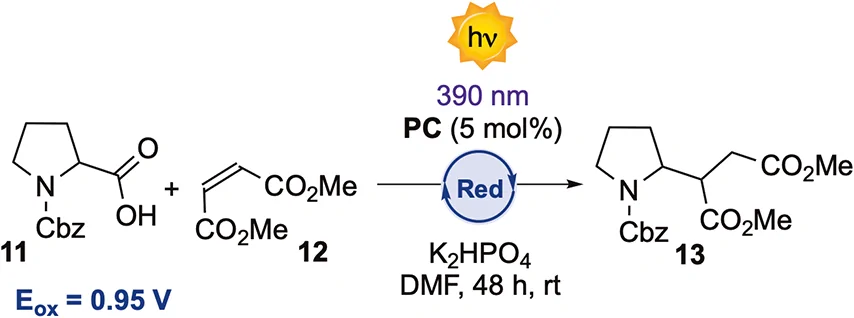
Giese Addition Using N-Cbz-Pro (**11**) and Dimethyl Maleate (**12**)

Entry	PC	^1^H NMR Yield^a^ (%)
1	**4**	N.R.
2	**5**	N.R.
3	**6**	Traces
4	**7**	63
5	**8**	70 (61)
6	**1**	N.R.
7	**2**	77

a CH_2_Br_2_ as internal standard. N.R. = no reaction. The isolated yield is presented in parentheses.

After studying this new family of photocatalysts for mediating SET processes, we also explored their effectiveness as photosensitizers via EnT reactivity. In this regard, we performed the *E*–*Z* isomerization of *trans*-stilbene (E_T_ = 2.2 eV), a well reported benchmark reaction.^18^ For this reaction, all the TBA derivatives facilitated the photoisomerization in good yields (from 77 to 85% NMR yield, Table 4). Even when our minimalistic photocatalysts presented high values of E_T_, we believe that the activation of more challenging substrates such as **15** ^19^ or dimethyl fumarate^20^ through this Dexter energy transfer mechanism is not efficient. This can be explained by considering the peculiar geometry of our TBA derivatives in the excited state, avoiding a good electronic overlap between the donor and the acceptor,^21^ also corroborated by theoretical calculations for **15** (see SI, Section H).

**Table 4:**
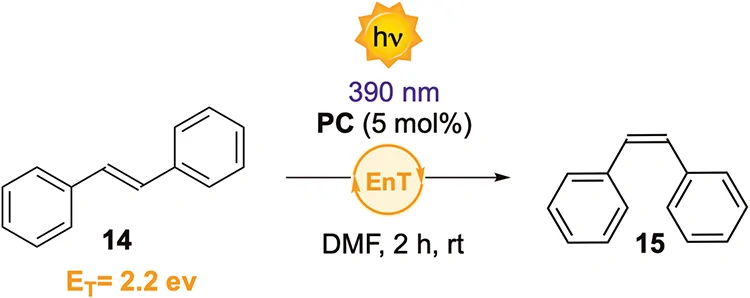
Photoisomerization of *trans*-Stilbene (**14**)

Entry	PC	^1^H NMR Yield^a^ (%)
1	**4**	81 (74)
2	**5**	77
3	**6**	78
4	**7**	85
5	**8**	85
6	**1**	N.R.
7	**2**	56

a CH_2_Br_2_ as internal standard. N.R. = no reaction. Isolated yield is presented in parentheses.

In this EnT protocol, PC **2** achieved a 56% NMR yield, which is consistent with our expectations. This result proves the capability of the TBA derivatives to facilitate the EnT processes.

In summary, we have introduced TBA derivatives as a novel class of organic bifunctional photocatalysts to conduct oxidative and reductive quenching cycles and energy transfer mechanisms. In the SET processes, these compounds showed a structural dependency related to the ability to form stable PC^•–^ and PC^•+^, where the latter matches better with the capacity of TBA to be oxidized. EnT was found to be facilitated by a slow ISC between the singlet and triplet states involved. Interestingly, we found that the triplet state of the molecules, has a mixed contribution of diverse triplet states due to the similar nature between them. Under our conditions, these minimalistic catalysts are as efficient as simple cores like thioxanthone and phenothiazine, but they also show versatility comparable to more complex D-A compounds previously reported. We also studied the bend-to-planar conformational change experimentally and theoretically, and established the photophysical pathway of this process. The ground- and excited-state behavior was determined using electrochemical, optical, and theoretical analyses. These mechanistic insights may provide a foundation for expanding research on the design of multifaceted organic photocatalysts, finding new applications, and discovering new reactivity.

## Supplementary Material



## Data Availability

The data underlying this study are available in the published article and its Supporting Information.

## Notes

Experimental procedures, full characterization data, X-ray characterization, theoretical studies, and NMR spectra (PDF)
