# Supplementary material for: Tribenzo[b,d,f]azepines as Minimalistic Purely Organic Photocatalysts
Source: Org Lett. 2026 Oct 25;28(44):13648–53. doi: 10.1021/acs.orglett.6c03716 (PMC13750074; doi:10.1021/acs.orglett.6c03716)
Supplement: Supplementary file 1 [file ol6c03716_si_001.pdf]

Supporting Information

for

Tribenzo[*b*,*d*,*f*]azepines as minimalistic purely organic photocatalysts.

Gabriele Pozza,[a] Gustavo Mondragón-Solórzano,[b] Simone Baldon,[a] Jorge Humbrías-Martín,[a] Jonas Žurauskas,[c] Elizaveta Meshcheriakova,[d] Luca Dell'Amico*,[a] and Katy Medrano-Uribe*.[a]

Addresses:
[a] Department of Chemical Sciences, University of Padova, Via Francesco Marzolo 1, 35131, Padova, Italy.
[b] Department of Chemistry and Chemical Biology, Northeastern University, 02115, Boston, Massachusetts, USA.
[c] Faculty of Chemistry and Geosciences, Vilnius University, Naugarduko 24, LT-03225, Vilnius, Lithuania.
[d] Chemistry Department, Lomonosov Moscow State University, Leninskie Gory 1/3, 119991, Moscow, Russia.

Contents

A. General Information 3

B. Experimental setup for light irradiation: Reaction setup with Kessil LED PR160L 5

C. Synthesis of the tribenzo[*b,d,f*]azepine and 6,12-dibromo-9H-tribenzo[*b,d,f*]azepine. 6

Tribenzo[*b,d,f*]azepine (**17**) 6

6,12-dibromo-9H-tribenzo[*b,d,f*]azepine (**18**) 6

D. Synthesis of TBA-based photocatalysts. 7

General procedure A 7

6,12-diphenyl-9H-tribenzo[*b,d,f*]azepine (**4**) 7

6,12-bis(4-methoxyphenyl)-9H-tribenzo[*b,d,f*]azepine (**5**) 7

6,12-di(naphthalen-2-yl)-9H-tribenzo[*b,d,f*]azepine (**6**) 8

4,4'-(9H-tribenzo[*b,d,f*]azepine-6,12-diyl)dibenzonitrile (**7**) 8

6,12-bis(4-(trifluoromethyl)phenyl)-9H-tribenzo[*b,d,f*]azepine (**8**) 8

E. Photoredox properties 9

9-(4-(phenylsulfonyl)phenyl)-9*H*-tribenzo[*b*,*d*,*f*]azepine (**4**) 10

6,12-bis(4-methoxyphenyl)-9H-tribenzo[*b,d,f*]azepine (**5**) 14

6,12-di(naphthalen-2-yl)-9H-tribenzo[*b,d,f*]azepine (**6**) 18
4,4'-(9H-tribenzo[*b,d,f*]azepine-6,12-diyl)dibenzonitrile (**7**) 22
6,12-bis(4-(trifluoromethyl)phenyl)-9H-tribenzo[*b*,*d*,*f*]azepine (**8**) 26
F. X-ray characterization. 31
G. Evaluation of photocatalytic activity 40
Oxidative quenching cycle 40
General Procedure B 40
Reductive quenching cycle 41
General Procedure C 41
Energy transfer 42
General Procedure D 42
H. Theoretical studies 45
I. References 72
J. NMR spectra 74

A. General Information

All reactions were carried out in anhydrous solvents purchased from commercial suppliers over molecular sieves in a sealed bottle which were used without further purification. Chemicals were purchased from commercial sources (Sigma–Aldrich, Fluorochem or TCI) and used without further purification. Organic solvents were purchased from Sigma–Aldrich. Reactions were monitored by thin- layer chromatography on silica gel 60F254, and/or by NMR spectroscopy.

NMR spectra were collected on a Bruker 400 Avance III HD spectrometer equipped with a BBI-z grad probehead, at the denoted spectrometer frequency given in MHz for the specified nucleus. Reported coupling constants and chemical shifts were based on a first-order analysis. The internal reference for ^{1}H NMR was the residual peak of $CDCl_3$ (7.26 ppm). The internal reference for ^{13}C NMR was the residual peak of $CDCl_3$ (77.16 ppm). All coupling constants (J) are reported in Hz with the following abbreviations: s = singlet, d = doublet, dd = double doublet, ddd= double of doublets of doublets, t = triplet, dt = double triplet, q = quadruplet, m = multiplet, br = broad.

Thin-layer chromatography (TLC) analysis was performed on pre-coated Merck TLC plates (silica gel 60G F254, 0.25 mm). Chromatographic purification of the products was accomplished using flash chromatography on silica gel (SiO_2, 0.04-0.0063 mm) purchased from Sigma-Aldrich, with the indicated solvent system according to the standard techniques, or with pre-coated Merck preparative TLC plates (silica gel 60G F254, 20x20 cm). Organic solutions were concentrated under reduced pressure on a Büchi rotary evaporator.

Steady-state absorption spectroscopy studies were performed at room temperature on a Varian Cary 50 UV-vis; 10 mm path length Hellma Analytics 100 QS quartz cuvettes were used.

Steady-state emission spectroscopy studies were performed at room temperature on a Varian Cary Eclipse Fluorescence spectrophotometer; 10 mm path length Hellma Analytics 117.100F QS quartz cuvettes were used.

Excited-state lifetimes measurements were performed at room temperature on a FLS1000 UV/Vis/NIR photoluminescence spectrometer (Edinburgh Instruments) at the PanLab facility of the Department of Chemical Sciences, University of Padova, founded by the MIUR-"Dipartimenti di Eccellenza" grant NExuS. The instrument was coupled with a high-speed detector with amplifier, operating in the spectral range: 230-850 nm and with a response time width.

The electrochemical characterizations were carried out in acetonitrile (MeCN)/0.1 M tetrabutylammonium hexafluorophosphate ($TBAPF_6$) at room temperature, on an BASi EC Epsilon potentiostat-galvanostat in a glass cell. All the cyclic voltammograms were recorded with a scan rate of 0.1 V/s. A typical three-electrode cell was employed, which was composed of a glassy carbon (GC) working electrode (3 mm diameter), a platinum wire as counter electrode and an Ag/AgCl electrode as reference electrode. The glass electrochemical cell was kept closed with a stopper annexed to the potentiostat. Oxygen was removed by purging the solvent with high-purity Nitrogen (N_2), introduced from a line into the cell by means of a glass pipe. The potential of ferrocenium/ferrocene (Fc^+/Fc) couple was used as internal reference system to calibrate the potentiostat. All the results were subsequently converted in V vs SCE, in agreement with the value reported in literature [$E_{1/2}(Fc^+/Fc)$ = +0.395 V vs SCE]. The IR compensation implemented within the potentiostat was used, and every effort was

made throughout the experiments to minimize the resistance of the solution. The full electrochemical reversibility of the voltammetric wave of ferrocene was taken as an indicator of the absence of uncompensated resistance effects. The GC electrode was polished before any measurement with diamond paste, carefully rinsed with de-ionized water, ethanol, acetone and ultrasonically rinsed with a methanol/ethanol/acetone 1:1:1 (v/v) mixture for 5 minutes. After each series of CV experiments, the electrochemical cell was carefully rinsed with ethanol, acetone and de- ionized water; afterwards, the cell and the magnetic stirrer were sonicated for 5-10 min with acetone.

High-resolution mass spectrometry (HRMS) analyses were performed on MicroTOF-Q Bruker (ESI) and a GC Thermo Scientific Trace 1300 GC unit coupled to an APPI MasCom source mounted on a Thermo Scientific Exactive Plus EMR mass unit (Orbitrap FT-HRMS analyzer) or on a Xevo G2-XS QToF.

The Kessil lamps PR160L (50W) were purchased from Kessil webpage: https://kessil.com/products/science_PR160L.php

B. Experimental setup for light irradiation: Reaction setup with Kessil LED PR160L

Figure S1 shows the general setup of a batch reaction performed under Kessil LED PR160L light irradiation (390 nm). The reaction mixture is placed at a fixed distance (about 3 cm) from the light source and stirred vigorously. A maximum of three reaction vessels were irradiated at the same time, following the lines of homogeneous irradiance provided by the producer. To maintain a stable reaction temperature, two fans were placed above the irradiated vials. All the reactions were carried out using 100% intensity.

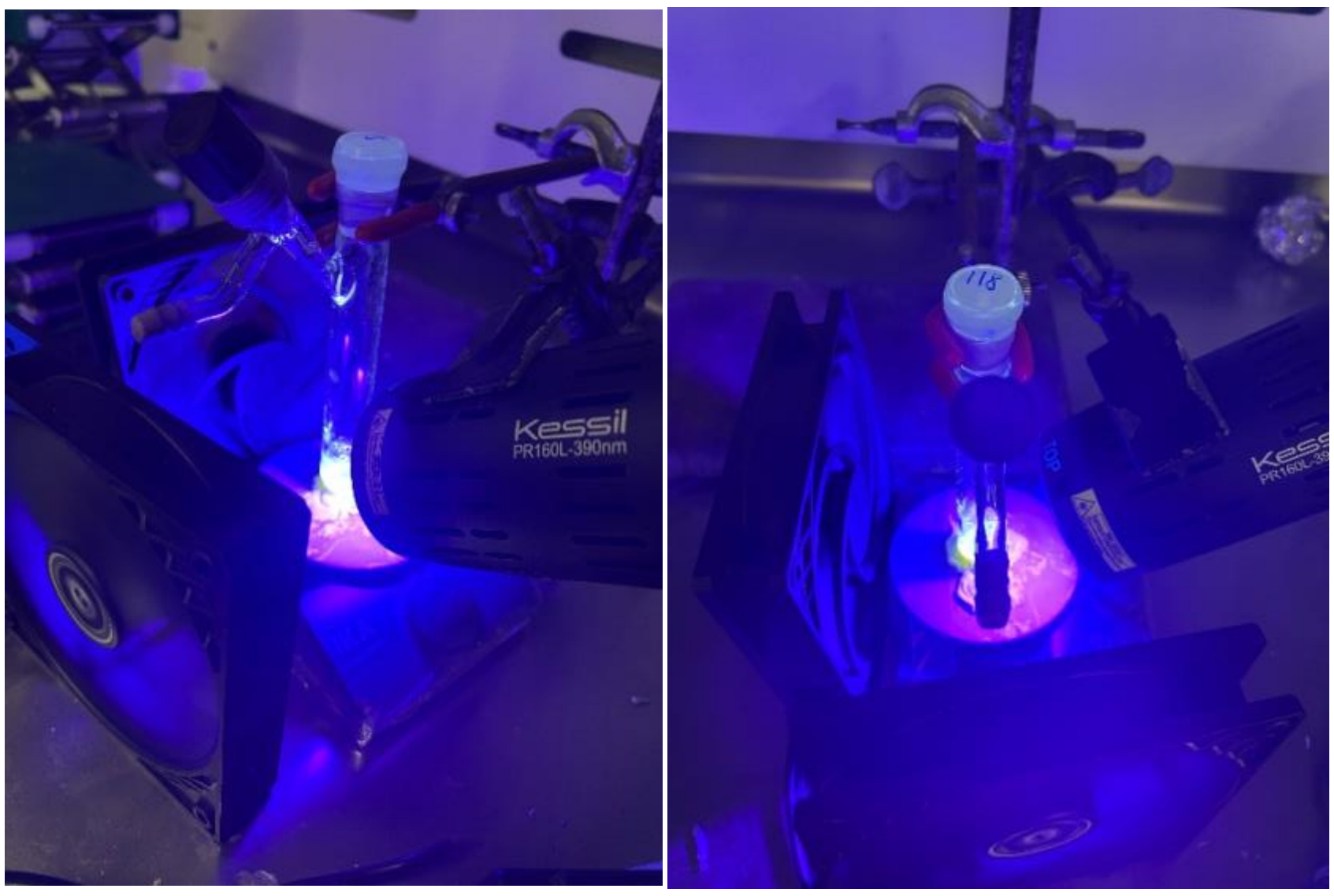

Figure S1: *Reaction setup using Kessil lights.*

C. Synthesis of the tribenzo[*b,d,f*]azepine and 6,12-dibromo-9H-tribenzo[*b,d,f*]azepine.

Tribenzo[*b,d,f*]azepine (**17**)

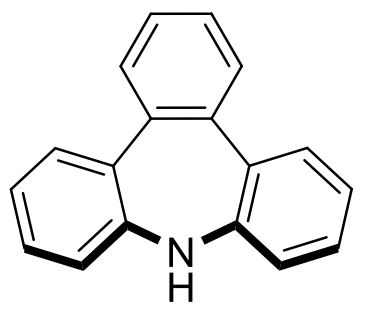

Tribenzo[*b,d,f*]azepine (**17**) was synthesized following a reported procedure.[1] The spectral data matched those reported in the literature. ^{1}H NMR (400 MHz, $CDCl_3$) δ 7.53 – 7.47 (m, 4H), 7.44 (dt, *J* = 5.8, 3.5 Hz, 2H), 7.23 (ddd, *J* = 9.3, 6.9, 3.1 Hz, 2H), 7.13 (td, *J* = 7.6, 1.4 Hz, 2H), 6.90 (dd, *J* = 7.8, 1.3 Hz, 2H), 5.20 (s, 1H). ^{13}C NMR (101 MHz, $CDCl_3$) δ 151.1, 139.5, 132.8, 130.3, 130.24, 128.6, 127.9, 124.3, 119.9. **HRMS (ESI+)**: m/z $[M+H]^+$ calcd. for $C_{18}H_{14}N$: 244.1121; Found: 244.1118.

6,12-dibromo-9H-tribenzo[*b,d,f*]azepine (**18**)

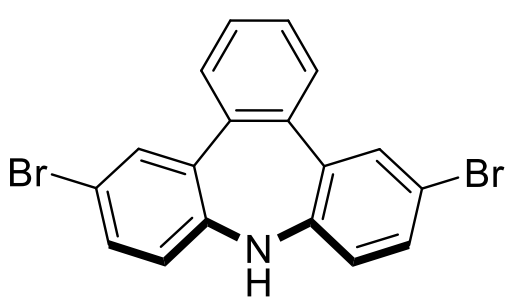

6,12-dibromo-9H-tribenzo[*b,d,f*]azepine **18** (132 mg, 0.542 mmol, , 1 eq.) was synthesized following a reported patent.[2] The starting material was dissolved in THF (2 ml, 0.27 M) using an ice bath, and N-bromosuccinamide (NBS) (202 mg, 1.138 mmol, 2.1 eq.) was added in portions. The mixture was stirred overnight at room temperature. After the complete consumption of the starting material, distilled water was added, and a liquid-liquid extraction was carried out using ethyl acetate. The combined organic layers were washed with water and dried over Na_2SO_4. After filtration and solvent evaporation, the target product was isolated by flash column chromatography (FC) over silica gel using Hexane/ethyl acetate (9:1). The compound was obtained in 80% of isolated yield (174.8 mg) as a brownish solid.**^{1}H NMR (400 MHz, $CDCl_3$)** δ 7.61 (d, *J* = 2.3 Hz, 2H), 7.47 (m, 5H), 7.35 (dd, *J* = 8.5, 2.3 Hz, 2H), 6.82 (d, *J* = 8.3 Hz, 2H).**^{13}C NMR (101 MHz, $CDCl_3$)** δ 149.6, 138.5, 134.6, 133.3, 131.4, 130.3, 128.3, 121.0, 117.1. **HRMS (ESI+)**: m/z $[M+H]^+$ calcd. for $C_{18}H_{12}Br_2N$: 399.9258; Found: 399.9250.

D. Synthesis of TBA-based photocatalysts.

General procedure A

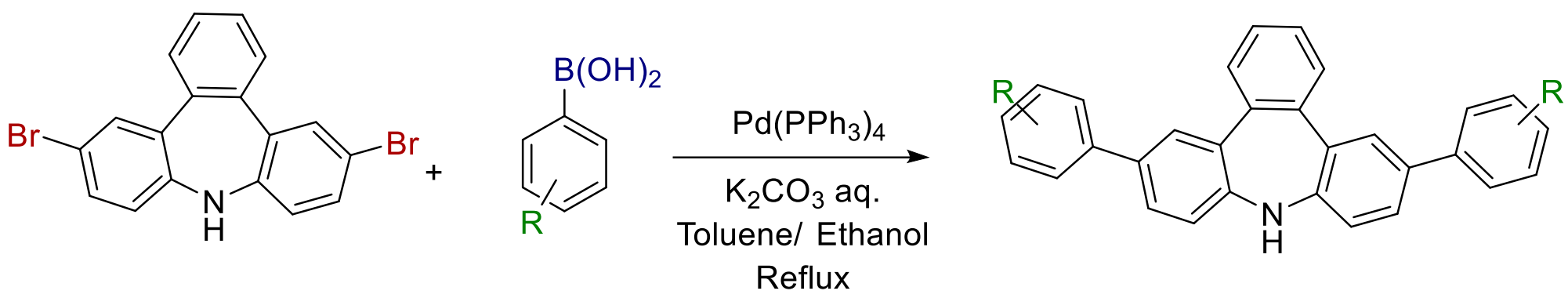

In a 100 ml two-neck flask with a condenser and a Teflon-coated magnetic stir bar was charged with 6,12-dibromo-9H-tribenzo[*b,d,f*]azepine (1 equiv.), the corresponding boronic acid (4 equiv.), $Pd(PPh_3)_4$ (20 mol%), in aqueous K_2CO_3 2.0 M (0.08 M), Ph-Me (0.05 M) and absolute EtOH (0.08M). The reaction mixture was purged with nitrogen for 15 minutes. The reaction was heated until reflux (120°C, approx.) for 12-16 h. Upon completion, the reaction mixture was cooled to room temperature, poured into water, and extracted with ethyl acetate. The combined organic layers were washed with water and dried over Na_2SO_4. After filtration and solvent evaporation, the crude was purified by flash column chromatography (FC) over silica gel to furnish the target product.

6,12-diphenyl-9H-tribenzo[*b,d,f*]azepine (**4**)

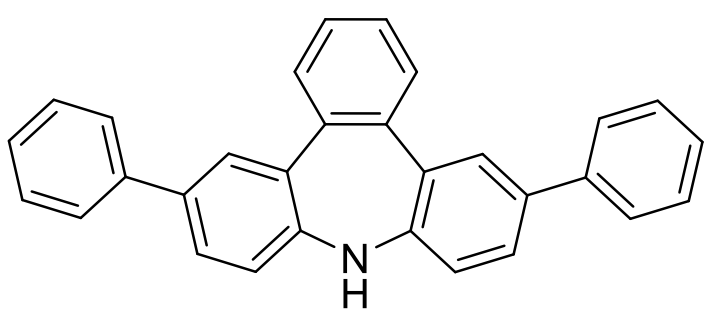

Synthesized following the general procedure starting from phenyl boronic acid (243.86 mg, 1.99 mmol, 4 equiv), 6,12-dibromo-9H-tribenzo[*b,d,f*]azepine (200.0 mg, 0.498 mmol, 1 equiv, $Pd(PPh_3)_4$ (115.12 mg ,0.099 mmol, 20 mol%), dissolved in aqueous K_2CO_3 2.0 M (6.22 ml, 0.08 M), Ph-Me (10 ml, 0.05 M) and absolute EtOH (6.22 ml, 0.08M). The reaction crude was purified by Flash column chromatography using Hexane/AcOEt 95:5 as eluent. Compound **3** was obtained in 36% yield (70.9 mg) as a brownish solid. **^{1}H NMR (400 MHz, $CDCl_3$)** δ 7.76 (d, *J* = 2.1 Hz, 2H), 7.61 (ddd, *J* = 9.3, 5.0, 2.5 Hz, 6H), 7.53 – 7.42 (m, 8H), 7.39 – 7.33 (m, 2H), 7.02 (d, *J* = 8.1 Hz, 2H), 5.35 (s, 1H). **^{13}C NMR (101 MHz, $CDCl_3$)** δ 150.2, 140.7, 139.4, 137.4, 132.9, 130.2, 129.1, 128.8, 128.0, 127.2, 127.0, 126.9, 120.2. **HRMS (ESI+)**: $[M+H]^+$ m/z Calcd. for $C_{30}H_{22}N$: 396.1747; Found: 396.1739.

6,12-bis(4-methoxyphenyl)-9H-tribenzo[*b,d,f*]azepine (**5**)

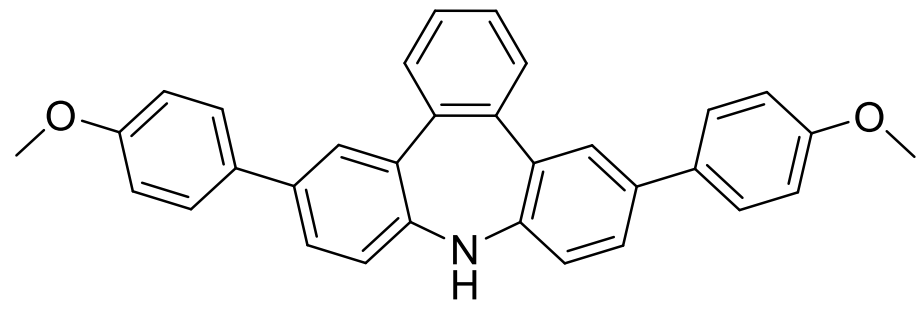

Synthesized following the general procedure starting from 4-methoxyphenylboronic acid (303.1 mg, 1.99 mmol, 4 equiv), 6,12-dibromo-9H-tribenzo[*b,d,f*]azepine (200.0 mg, 0.498 mmol, 1 equiv), $Pd(PPh_3)_4$ (115.12 mg ,0.099 mmol, 20 mol%), dissolved in aqueous K_2CO_3 2.0 M (6.22 ml, 0.08 M), Ph-Me (10 ml, 0.05 M) and absolute EtOH (6.22 ml, 0.08M). The reaction crude was purified by Flash column chromatography using Hexane/AcOEt 95:5 as eluent. Compound **4** was obtained in 39% yield (81.6 mg) as a white solid.**^{1}H NMR (400 MHz, $CDCl_3$)** δ 7.68 (d, *J* = 2.1 Hz, 2H), 7.56 (dt, *J* = 7.3, 3.6 Hz, 2H), 7.54 – 7.48 (m, 4H), 7.46 (dd, *J* = 5.8, 3.4 Hz, 2H), 7.41 (dd, *J* = 8.1, 2.1 Hz, 2H), 6.97 (dd, *J* = 8.5, 2.4 Hz, 6H), 5.29 (s, 1H), 3.85 (s, 6H). **^{13}C NMR (101 MHz, $CDCl_3$) δ** 159.0, 149.8, 139.5, 137.0, 133.3,

132.9, 130.2, 128.7, 128.0, 127.9, 126.8, 120.1, 114.2, 55.4. **HRMS (ESI+)**: $[M+H]^+$ m/z calcd. for $C_{32}H_{26}NO_2$: 456.1959; Found: 456.1948.

6,12-di(naphthalen-2-yl)-9H-tribenzo[*b,d,f*]azepine (**6**)

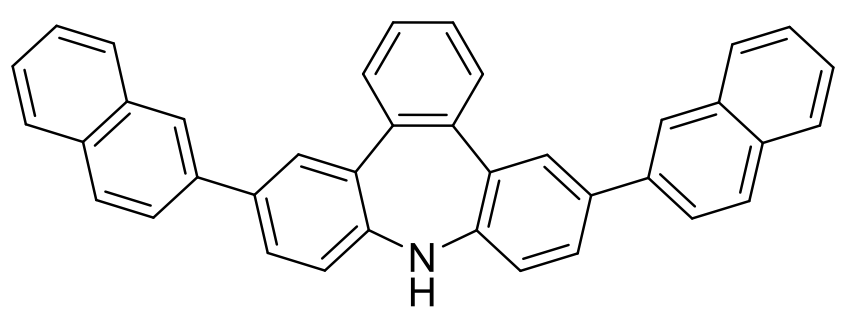

Synthesized following the general procedure starting from 2-Naphtylboronic acid (343 mg, 1.99 mmol, 4.0 equiv.), 6,12-dibromo-9H-tribenzo[*b,d,f*]azepine (200.0 mg, 0.498 mmol, 1 equiv), $Pd(PPh_3)_4$ (115.12 mg,0.099 mmol, 20 mol%), dissolved in aqueous K_2CO_3 2.0 M (6.22 ml, 0.08 M), Ph-Me (10 ml, 0.05 M) and absolute EtOH (6.22 ml, 0.08M). The reaction crude was purified by Flash column chromatography using Hexane/AcOEt 95:5 as eluent. Compound **5** was obtained in 52% yield (128.3 mg) as a yellowish solid. **^{1}H NMR (400 MHz, $CDCl_3$) δ** 8.03 (d, *J* = 1.8 Hz, 1H), 7.94 – 7.82 (m, 8H), 7.75 (dd, *J* = 8.5, 1.8 Hz, 2H), 7.61 (dt, *J* = 6.4, 3.9 Hz, 4H), 7.49 (pd, *J* = 6.7, 1.7 Hz, 7H), 7.06 (d, *J* = 8.1 Hz, 2H), 5.38 (s, 1H). **^{13}C NMR (101 MHz, $CDCl_3$)** δ 150.5, 139.5, 138.1, 137.4, 133.9, 133.1, 132.6, 130.4, 129.5, 128.6, 128.3, 127.8, 127.6, 126.5, 126.0, 125.5, 125.4, 120.4. **HRMS (ESI+)**: $[M+H]^+$ m/z calcd. for $C_{38}H_{26}N$: 496.2060; Found: 496.2052.

4,4'-(9H-tribenzo[*b,d,f*]azepine-6,12-diyl)dibenzonitrile (**7**)

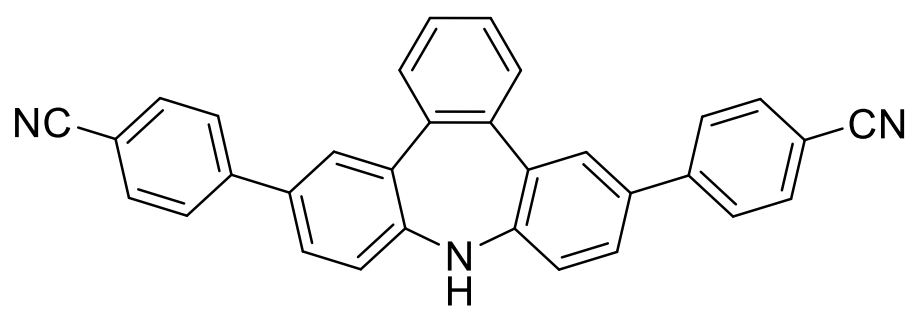

Synthesized following the general procedure starting from 4-cyanophenylboronic acid (293.07 mg, 1.99 mmol, 4.0 equiv.), 6,12-dibromo-9H-tribenzo[*b,d,f*]azepine (200.0 mg, 0.498 mmol, 1 equiv), $Pd(PPh_3)_4$ (115.12 mg ,0.099 mmol, 20 mol%), dissolved in aqueous K_2CO_3 2.0 M (6.22 ml, 0.08 M), Ph-Me (10 ml, 0.05 M) and absolute EtOH (6.22 ml, 0.08M). The reaction crude was purified by Flash column chromatography using Hexane/AcOEt 95:5 as eluent. Compound **6** was obtained in 62% yield (137.5 mg) as a yellowish solid. **^{1}H NMR (400 MHz, $CDCl_3$)** δ 7.74 – 7.69 (m, 6H), 7.67 (d, *J* = 8.5 Hz, 4H), 7.55 – 7.45 (m, 6H), 7.03 (d, *J* = 8.1 Hz, 2H), 5.45 (s, 1H). **^{13}C NMR (101 MHz, CDCl3**) δ 151.3, 145.0, 139.1, 135.9, 133.3, 132.8, 130.4, 129.3, 128.6, 127.5, 127.4, 120.7, 119.1, 109.9. **HRMS (ESI+)**: $[M+H]^+$ m/z calcd. for $C_{32}H_{20}N_3$: 446.1652; Found: 446.1643.

6,12-bis(4-(trifluoromethyl)phenyl)-9H-tribenzo[*b,d,f*]azepine (**8**)

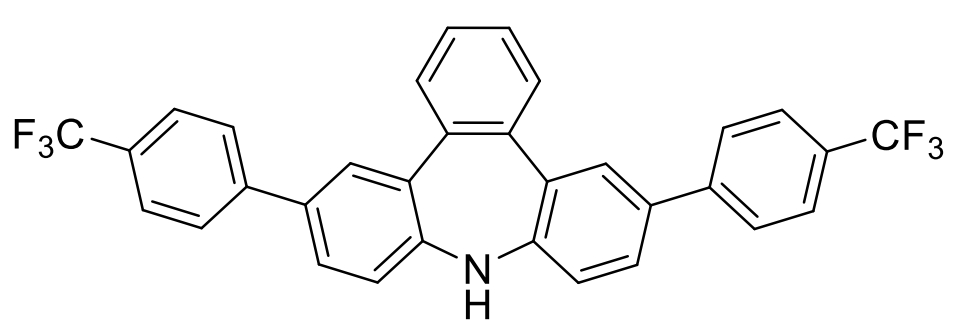

Synthesized following the general procedure starting from (4-(trifluoromethyl)phenyl)boronic acid (378.8 mg, 1.99 mmol, 4.0 equiv.), 6,12-dibromo-9H-tribenzo[*b,d,f*]azepine (200.0 mg, 0.498 mmol, 1 equiv), $Pd(PPh_3)_4$ (115.12 mg ,0.099 mmol, 20 mol%), dissolved in aqueous K_2CO_3 2.0 M (6.22 ml, 0.08 M), Ph-Me (10 ml, 0.05 M) and absolute EtOH (6.22 ml, 0.08M). The reaction crude was purified by Flash column chromatography using Hexane/AcOEt 95:5 as eluent. Compound **7** was obtained in 48% yield (127.0 mg) as a greenish solid. **^{1}H NMR (400 MHz, $CDCl_3$)** δ 7.75 (d, *J* = 2.1 Hz, 2H), 7.69 (s, 8H), 7.56 (dt, *J* = 7.2, 3.6 Hz, 2H), 7.49 (dt, *J* = 8.3, 3.1 Hz, 4H), 7.02 (d, *J* = 8.1 Hz, 2H), 5.39 (s, 1H). **^{13}C NMR (101 MHz, $CDCl_3$)** δ 150.8, 144.0, 139.1, 136.0, 133.1, 130.3, 129.3, 129.3, 129.0, 128.3, 127.4, 127.0, 126.5, 125.9, 125.8, 125.8, 125.7, 125.7, 123.0, 120.4**. HRMS (ESI+)**: $[M+H]^+$ m/z calcd. for $C_{32}H_{20}F_6N$: 532.1495; Found: 532.1507.

E. Photoredox properties

Luminescence quantum yield measurements.

The luminescence quantum yield measurements were performed with Quinine sulfate $1x10^{-5}$ M in H_2SO_4 0.5M (Φ = 0.54). Diverse substrate solutions were used:

4($1x10^{-5}$ M), **5** ($5x10^{-6}$M), **6** ($2.5x10^{-6}$ M), **7** and **8** ($5x10^{-6}$ M).

$\Phi_x = \Phi_{st} \times (I_x/ I_{st}) \times (f_{st}/f_x) \times (\eta^2{}_x /\eta^2{}_s)$

I is the measured integrated fluorescence emission intensity, f is the absorption factor, η is the refractive index of the solvent and Φ is the quantum yield. The index x denotes the sample and the index *st* denotes the standard.[3]

Redox potential of the excited state

The oxidation potential of the excited state photocatalysts $E^*{}_{ox}$ was estimated by means of the Rehm- Weller formula:

$E^*{}_{ox} = E_{ox} - E_{0,0}$

$E^*{}_{red} = E_{0,0} + E_{red}$

The oxidation potential E_{ox} and E_{red} were determined by cyclic voltammetry measurements, and the excitation energy $E_{0,0}$ was determined, when possible, from the intersection point between the absorption and the emission profiles,[4] or using the tangent method.[5]

Photoredox properties summary

PC	**λ_{max} abs (nm)**	**λ_{max} em (nm)**	**$E_{0,0}$ Exp (eV)**	**τ S (ns)**	**τ T (μs)**	**Stokes shift (nm)**	**QY (%)**	**E_{ox} (V)**	**E^*_{ox} (V)**	**E_{red} (V)**	**E^*_{red} (V)**	**E_T Exp (eV)**
**4**	264	527	3.8[a]	6.9	654	263	2	0.68	-2.62	----[c]	----	3.3
**5**	270	534	3.8[a]	6.1	786	264	2	0.89	-2.40	----[c]	----	3.2
**6**	304	527	2.9	6.9	736	223	5	0.66	-2.24	-2.33	0.57	3.3
**7**	315	512	3.0	7.4	---[b]	197	14	1.10	-1.90	-2.00	1.0	3.3
**8**	303	520	3.5[a]	7.8	805	218	5	0.73	-2.77	-2.19	1.31	3.3

Table S1: *All potentials are reported in V vs SCE.*
Measurements were performed in MeCN at room temperature.
Measurements were performed in MeTHF at 77 K.
a. Values calculated using the tangent method
b. Not determined under the established conditions.
c. No presence of a reduction peak.

9-(4-(phenylsulfonyl)phenyl)-9*H*-tribenzo[*b*,*d*,*f*]azepine (**4**)

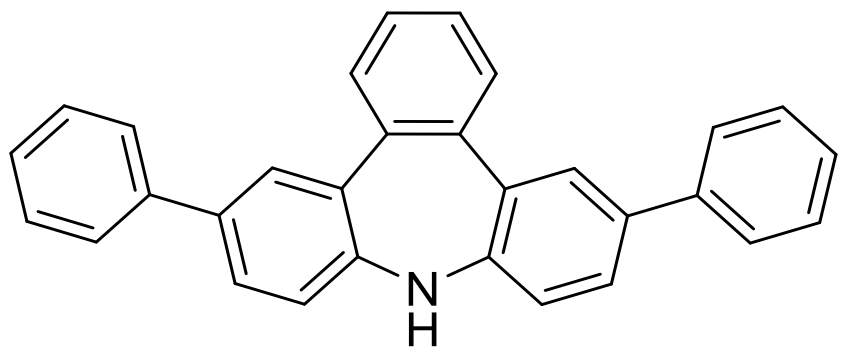

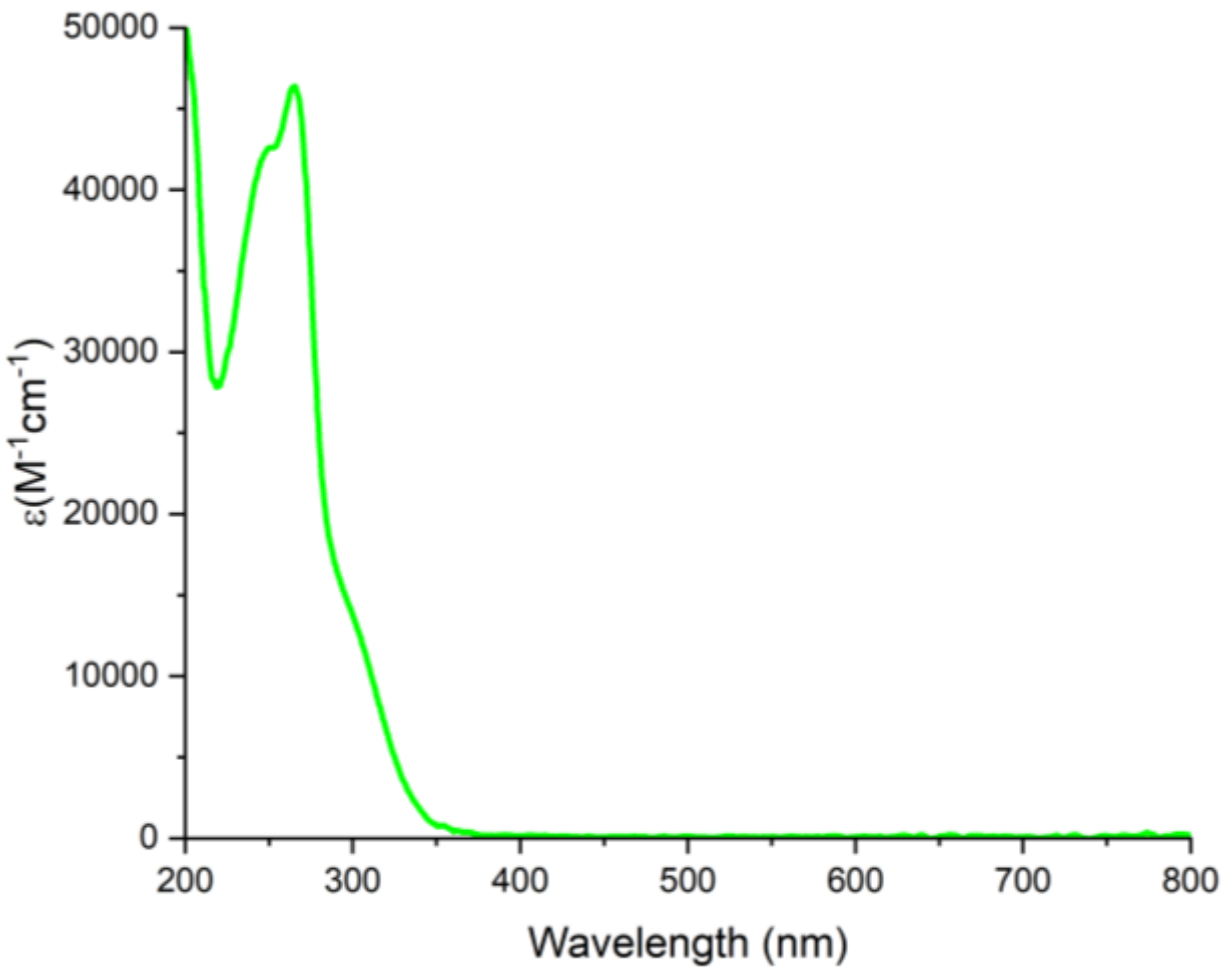

Concentration (mmol/mL)	
	1·10⁻⁵
λ peak	264 nm

Figure S2: *UV-Vis absorption of* ***4*** *at 1·10⁻⁵ M (green trace). Recorded in MeCN in quartz cuvettes (1 cm optical path).*

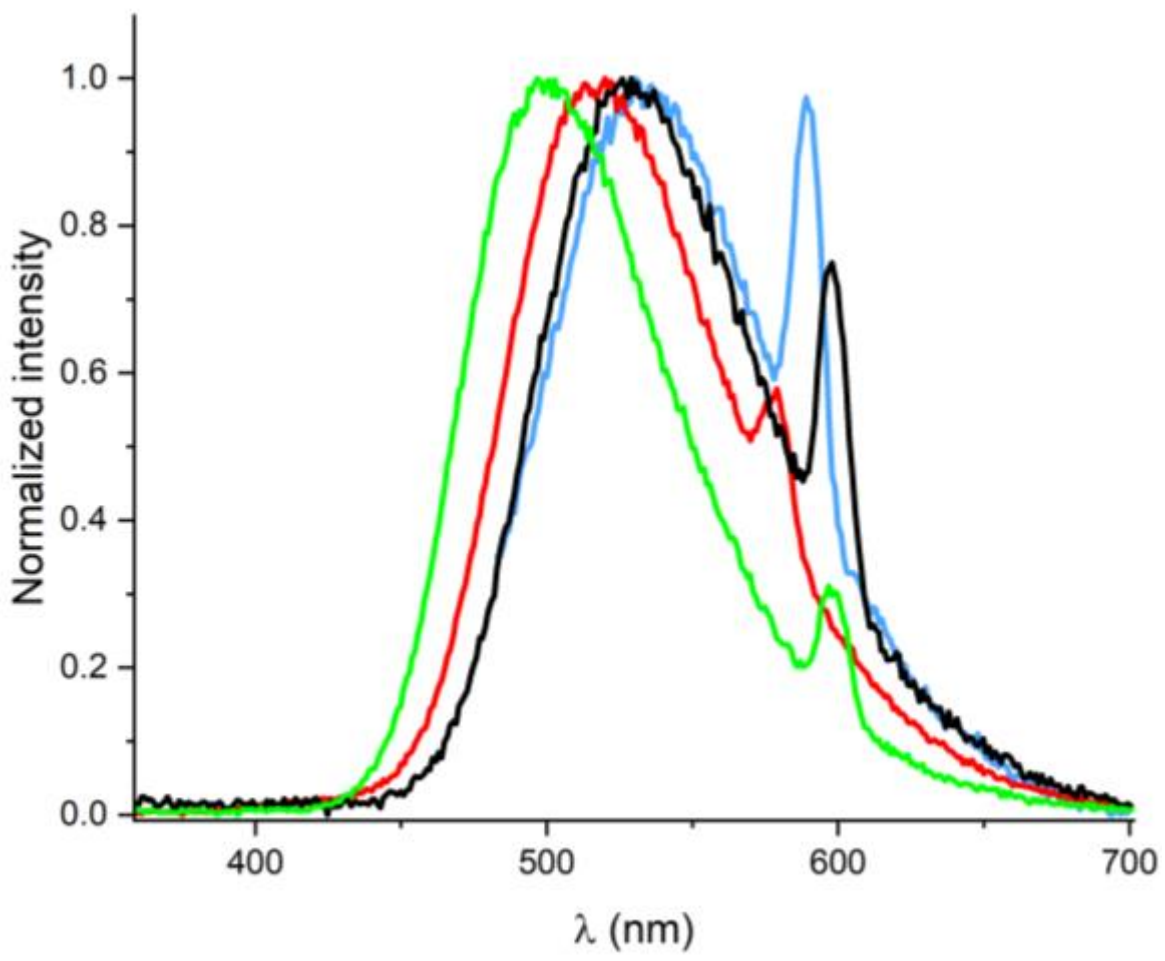

	Solvent	Peak (nm)
	DMF	530
	MeCN	527
	DCM	514
	Cyclohexane	498

Figure S3: *Normalized fluorescence emission of* ***4*** *at 1·10⁻⁵ M in different solvents. MeCN (black trace); DMF (blue trace); DCM (red trace), Cyclohexane (green trace).* Second harmonic is visualized.

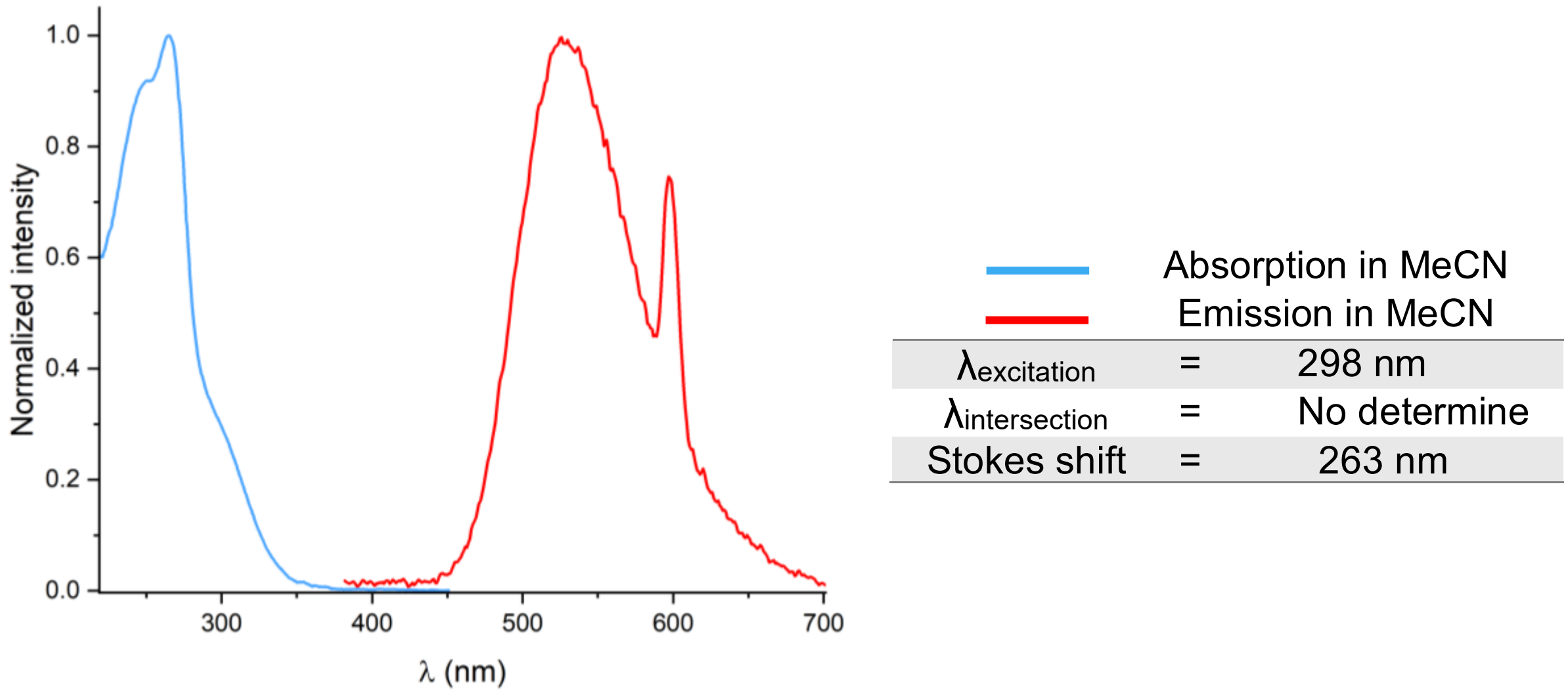

Figure S4: *Normalized optical absorption spectrum (blue trace) and emission spectra (red trace) of **4**.* $\lambda_{excitation}$ = 298 nm

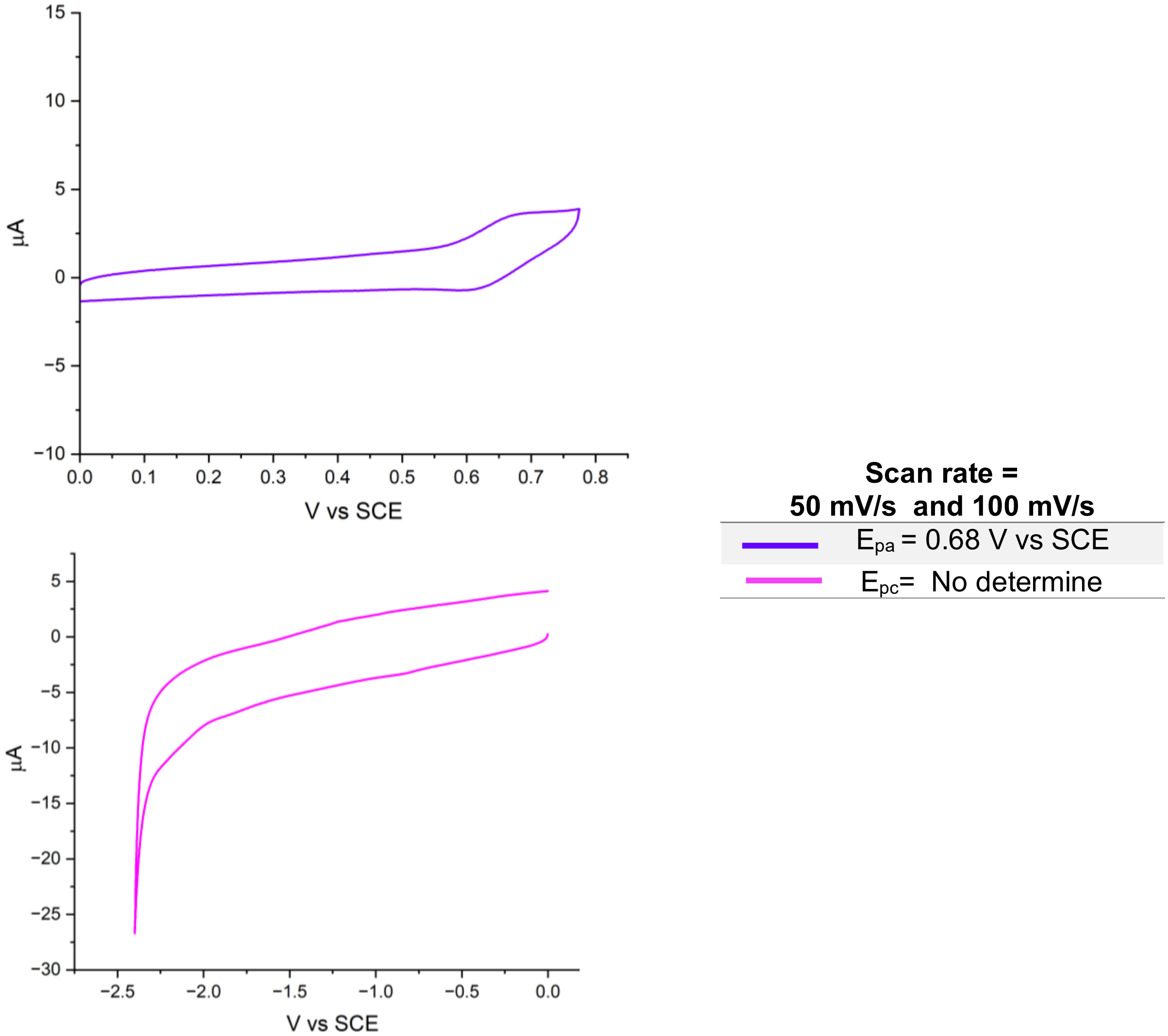

Figure S5*: Cyclic voltammograms of **4** (1mM) in a [Bu_4N][PF_6] (0.1 M) solution in MeCN. The plot follows the IUPAC convention. Glassy carbon electrode; Ag/AgCl (3 M NaCl) reference electrode; Pt counter electrode. For oxidation: Scan rate= 50 mV/s; initial potential= 0.0 V; switching potential= 0.77 V; final potential; 0.0 V. For the reduction: Scan rate= 100 mV/s. initial potential= 0.0 V; switching potential= -2.4 V; final potential; 0.0 V. Reversibility and irreversibility were determined using different scan rates.*

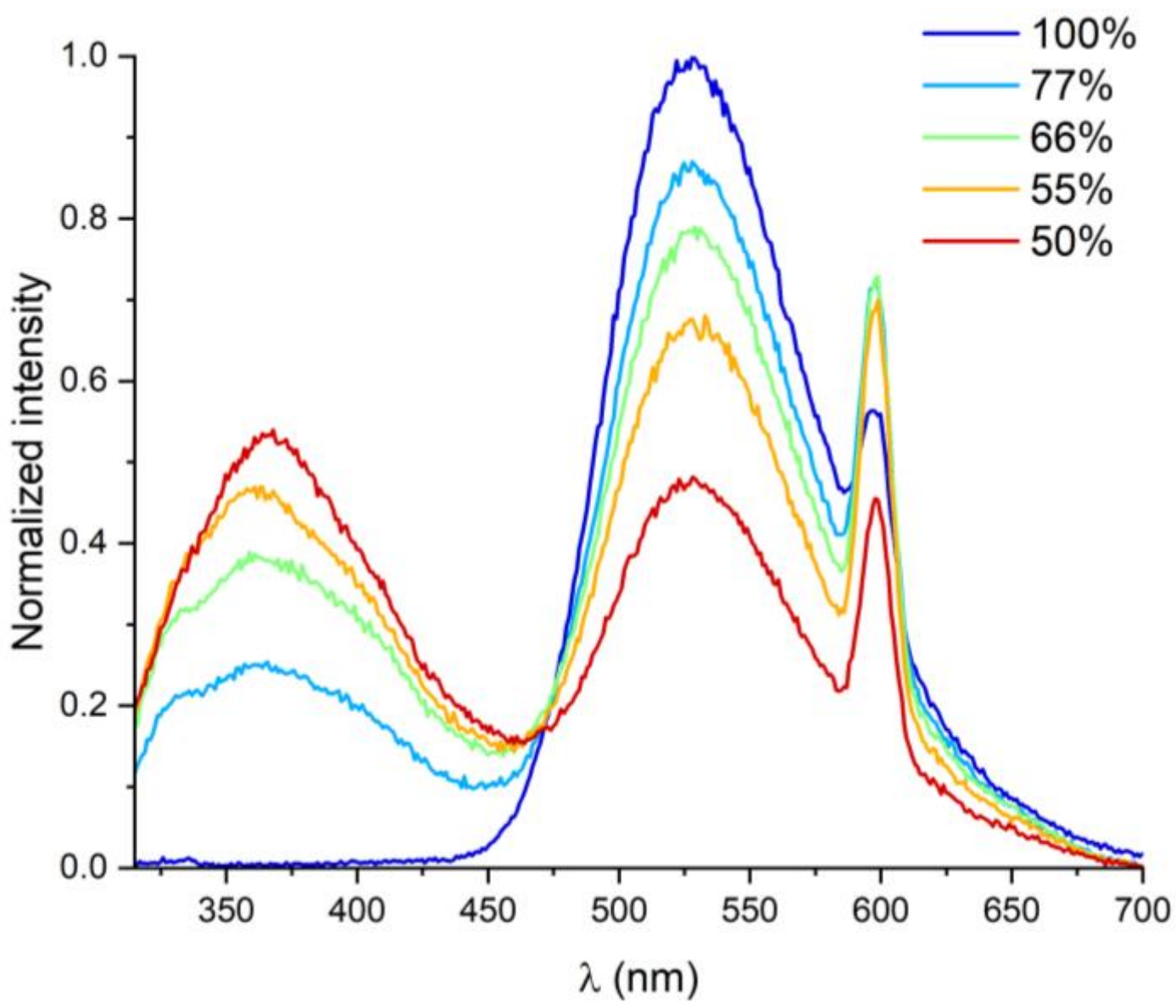

***Figure S6**: Fluorescence measurements of molecule **4** at 1·10^{-5} M in different volume ratios of methanol/glycerol as a function of viscosity at room temperature. Slit excitation:5. Slit emission:10. $\lambda_{excitation}$ = 300 nm. Second harmonic is visualized.*

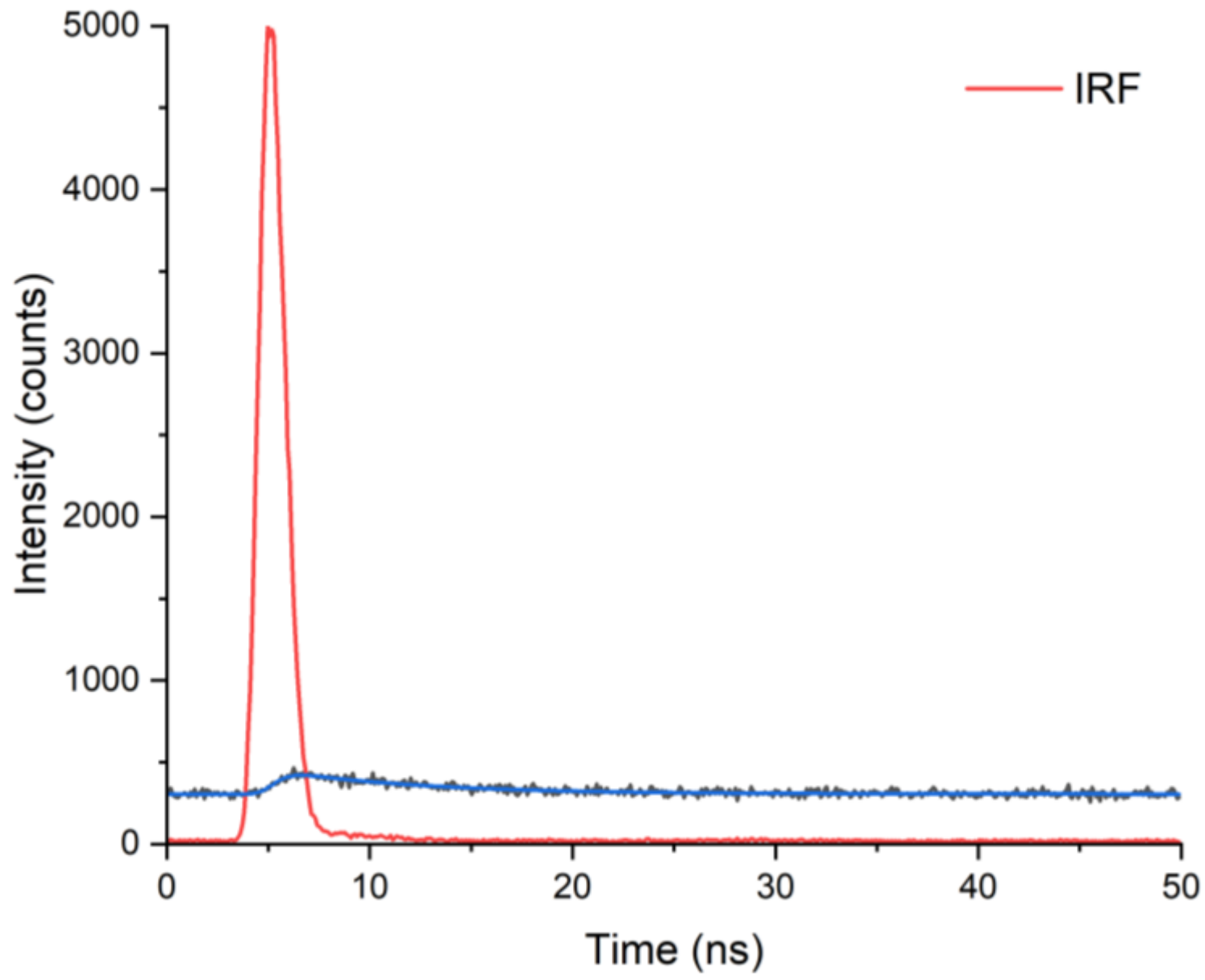

***Figure S7**: Time-resolved emission decay at room temperature(excitation at 286.2 nm) of **4** 1·10^{-5} M in MeCN solution measured by TC-SPC (τ = 6.9 ns), from deconvolution and single-exponential fitting, X2 = 1.02).*

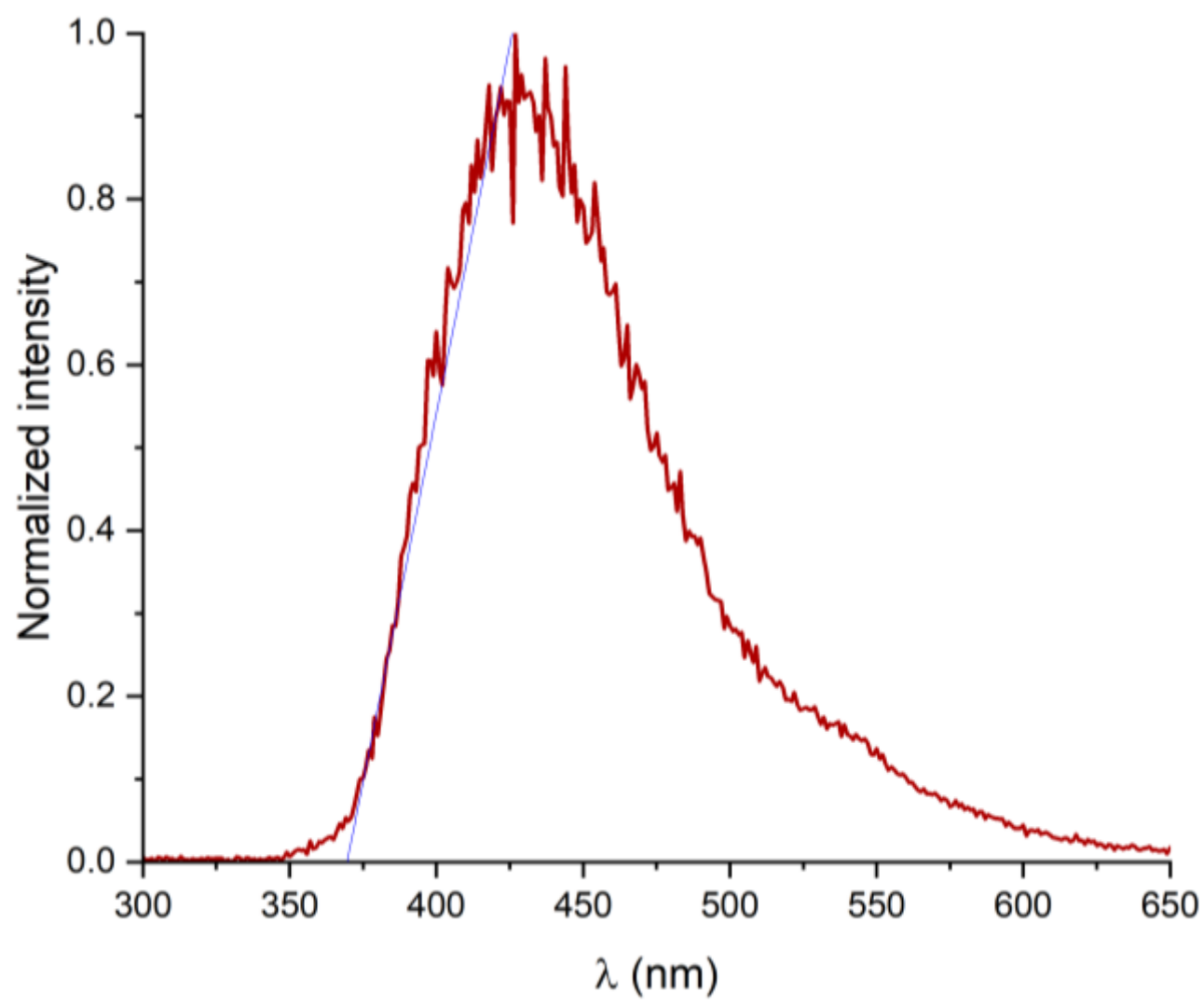

Figure S8: *Phosphorescence spectrum at 77 K of **4** at 1·10^{-5} M in 2-MeTHF. Excitation at 270 nm, slits= 10.*

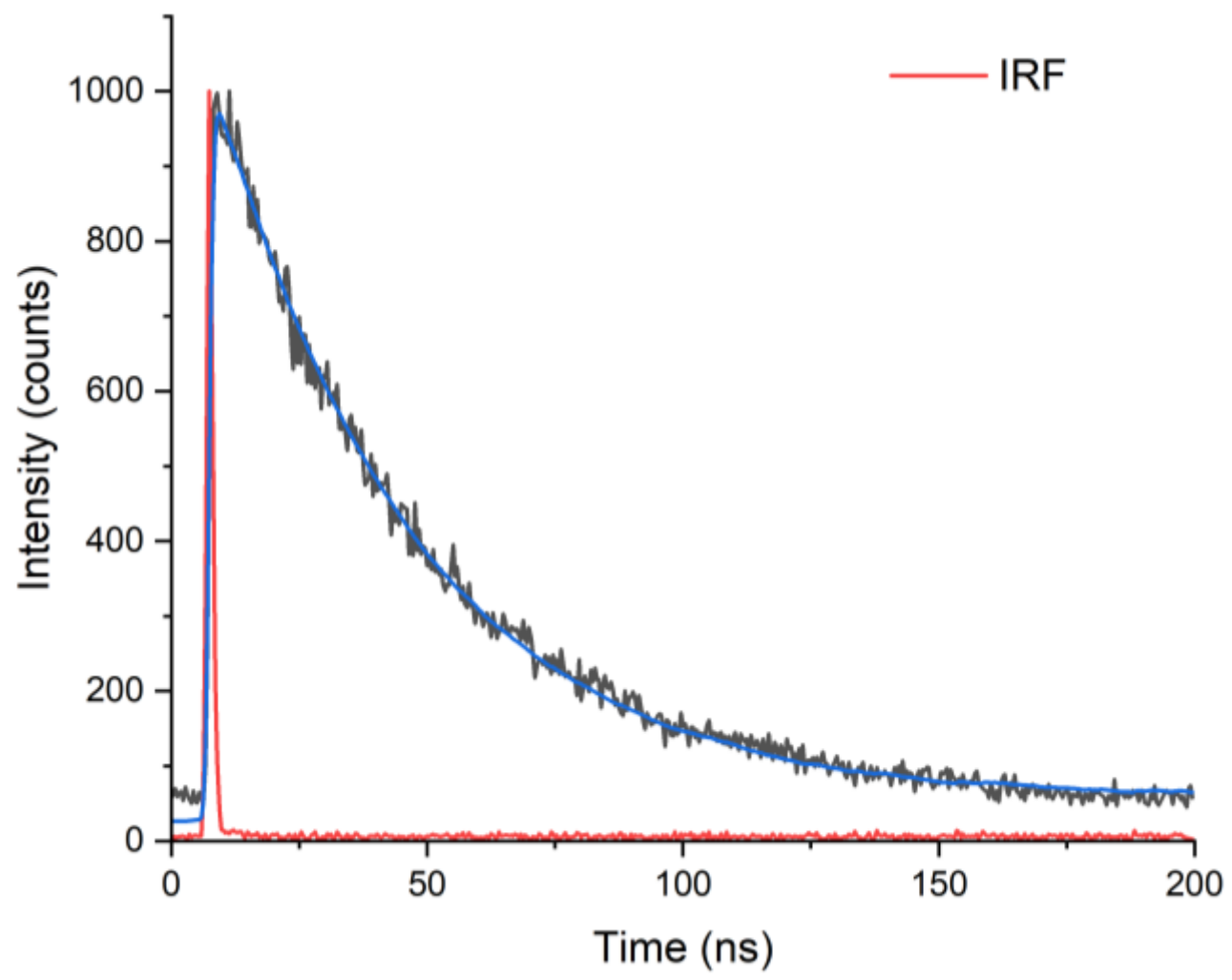

Figure S9: *Time-resolved emission decay at 77K of **4** at 1·10^{-5} M in 2-MeTHF solution measured by TC-SPC (τ= 38.8 ns), from deconvolution and single-exponential fitting, X2 = 1.78). Excitation: 286.2 nm. Emission: 510 nm.*

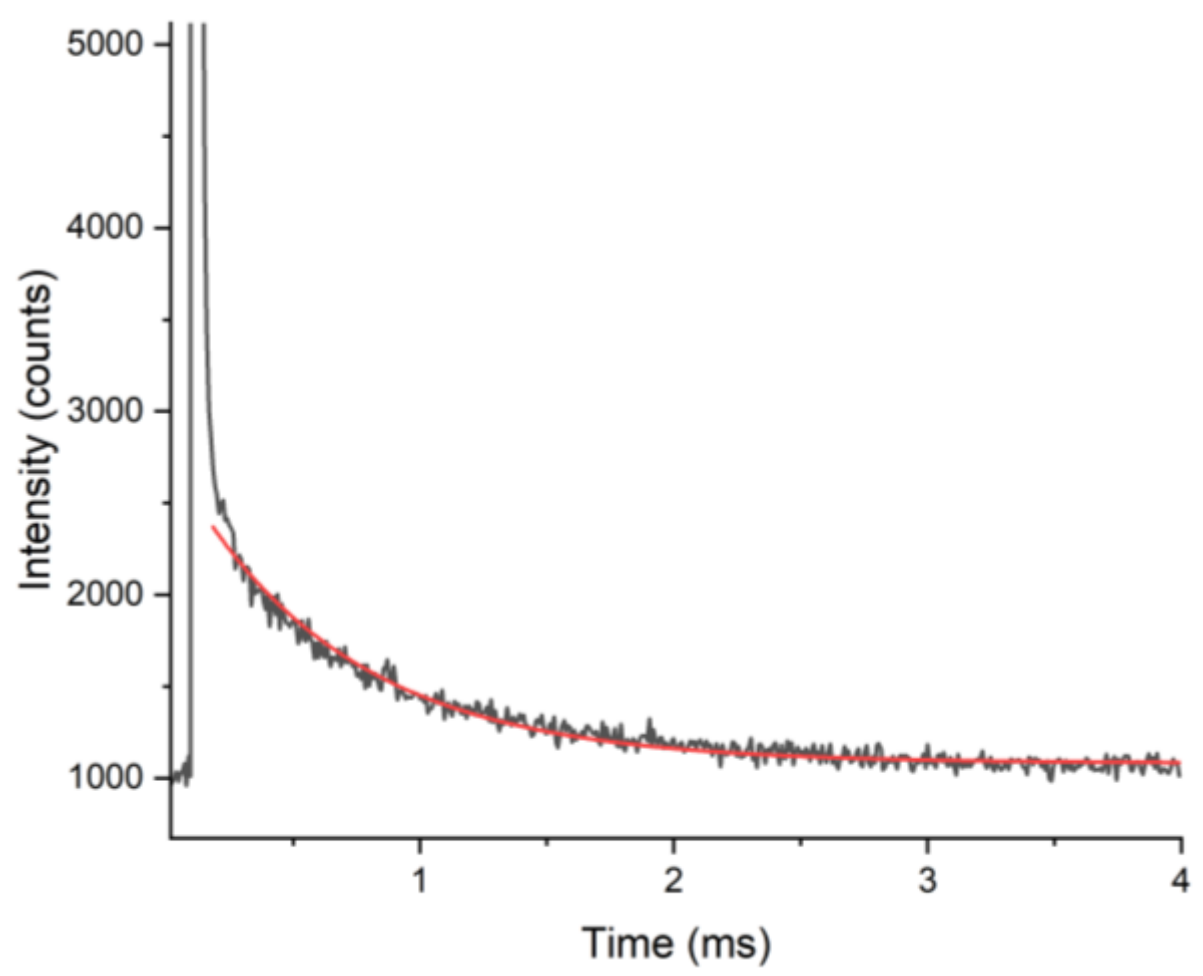

Figure S10: *Triplet-state lifetime measurement of **4** at 1·10^{-5} M in a frozen MeTHF glass at 77 K, giving τ= 654 μs. X2 = 1.79. Excitation: 264 nm. Emission: 440 nm.*

6,12-bis(4-methoxyphenyl)-9H-tribenzo[*b,d,f*]azepine (**5**)

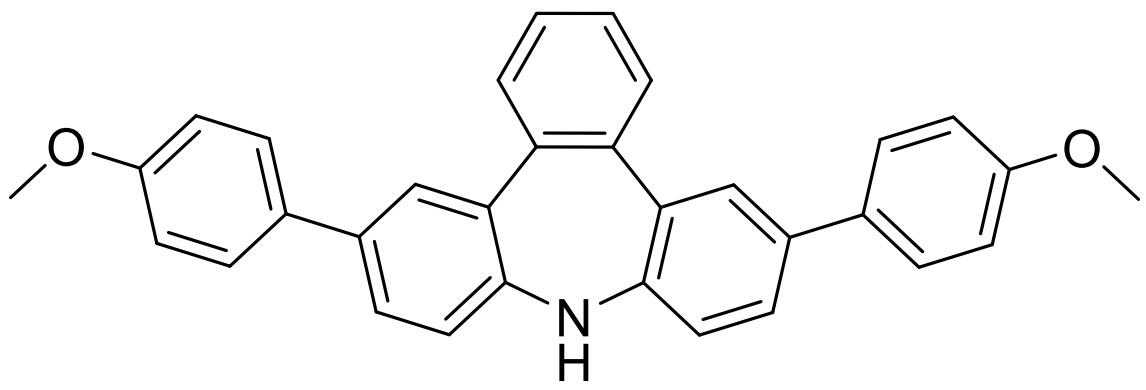

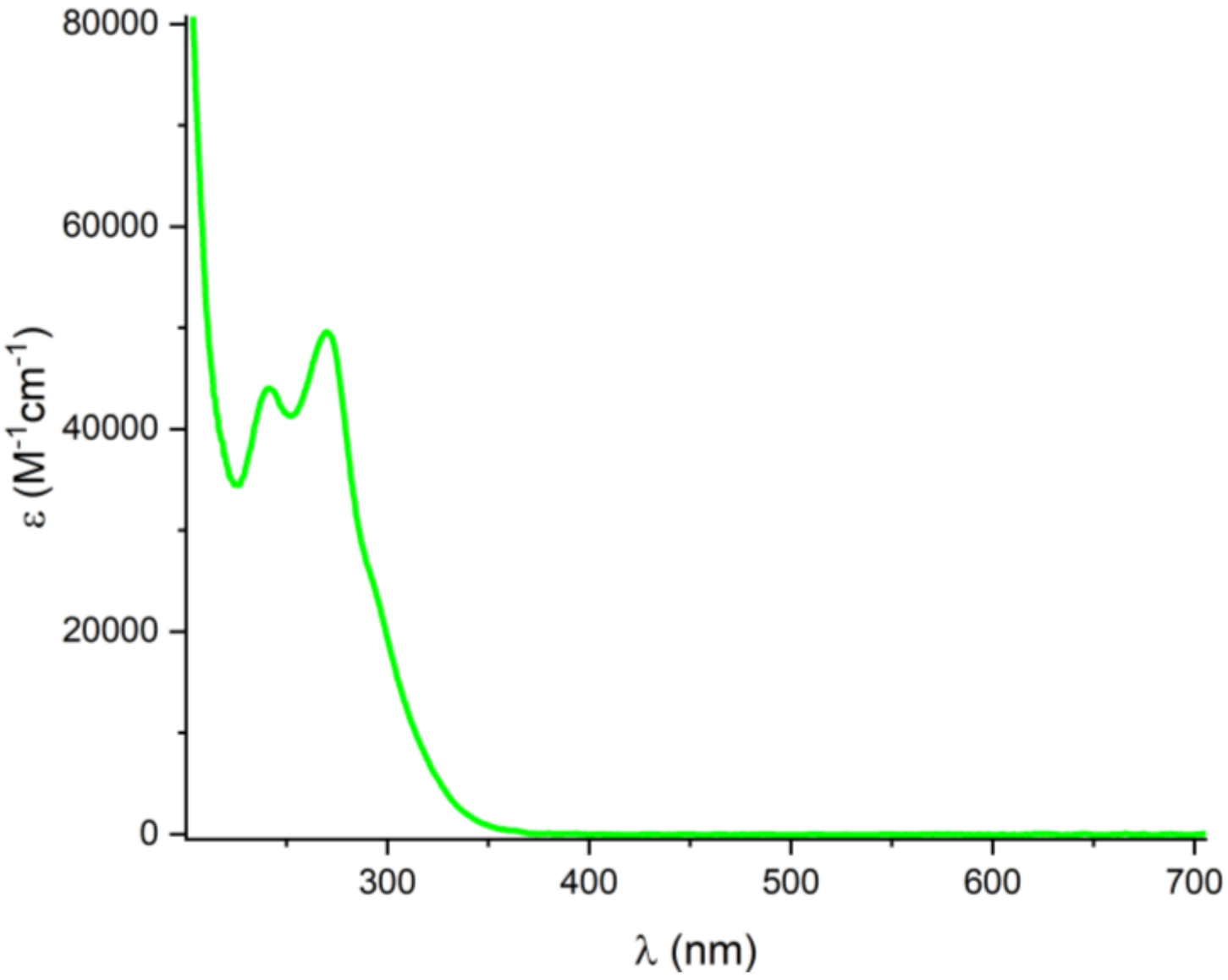

Concentration (mmol/mL)	
(green line)	*1·10⁻⁵*
λ peak	270 nm

Figure S11: *UV-Vis absorption of **5** at 1·10^{-5} M (green trace). Recorded in MeCN in quartz cuvettes (1 cm optical path).*

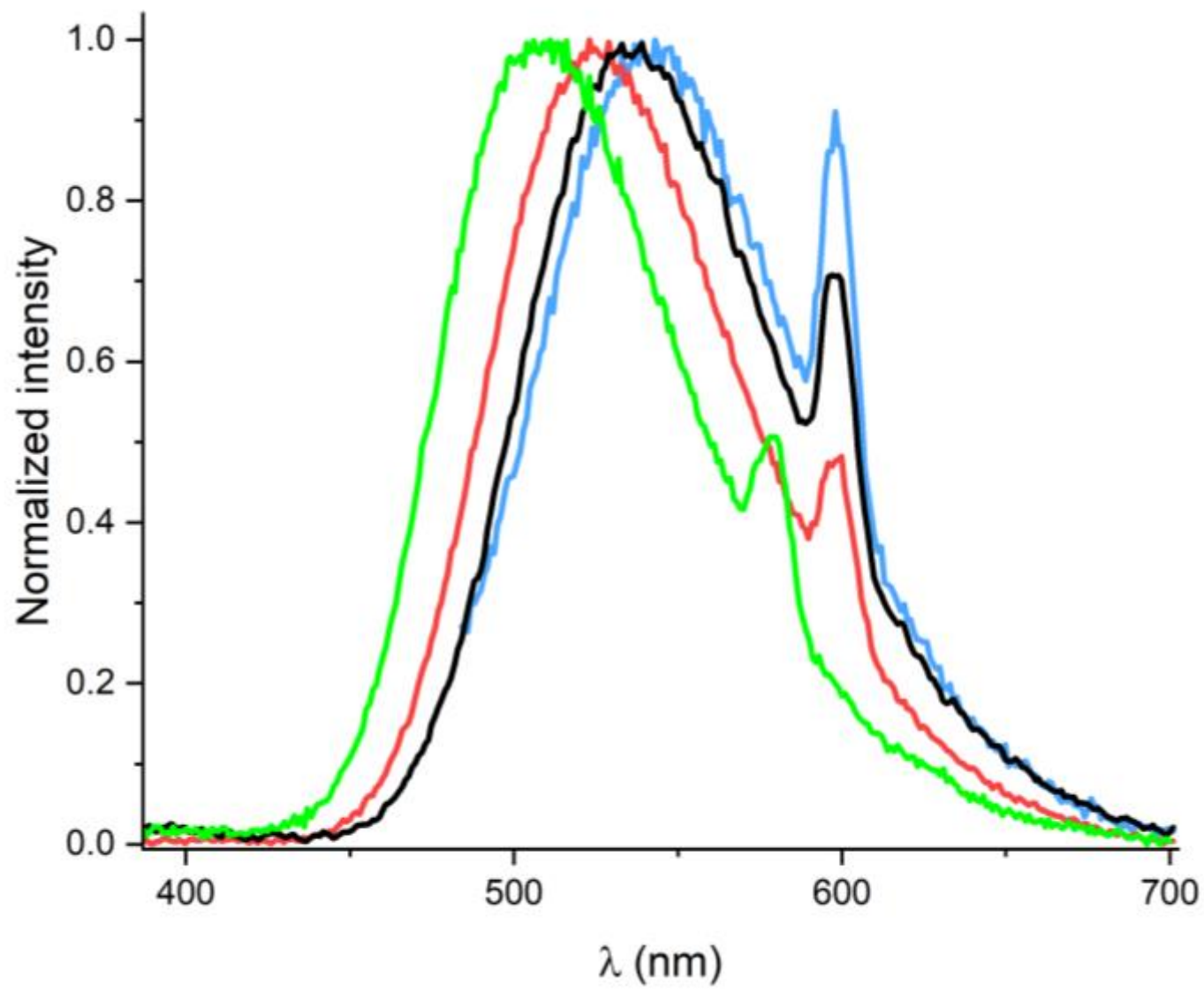

	Solvent	Peak (nm)
(blue line)	DMF	541
(black line)	MeCN	534
(red line)	DCM	523
(green line)	Cyclohexane	509

Figure S12: *Normalized fluorescence emission of **5** at 1·10^{-5} M in different solvents. DMF (blue trace); MeCN (black trace); DCM (red trace); Cyclohexane (green trace). Second harmonic is visualized.*

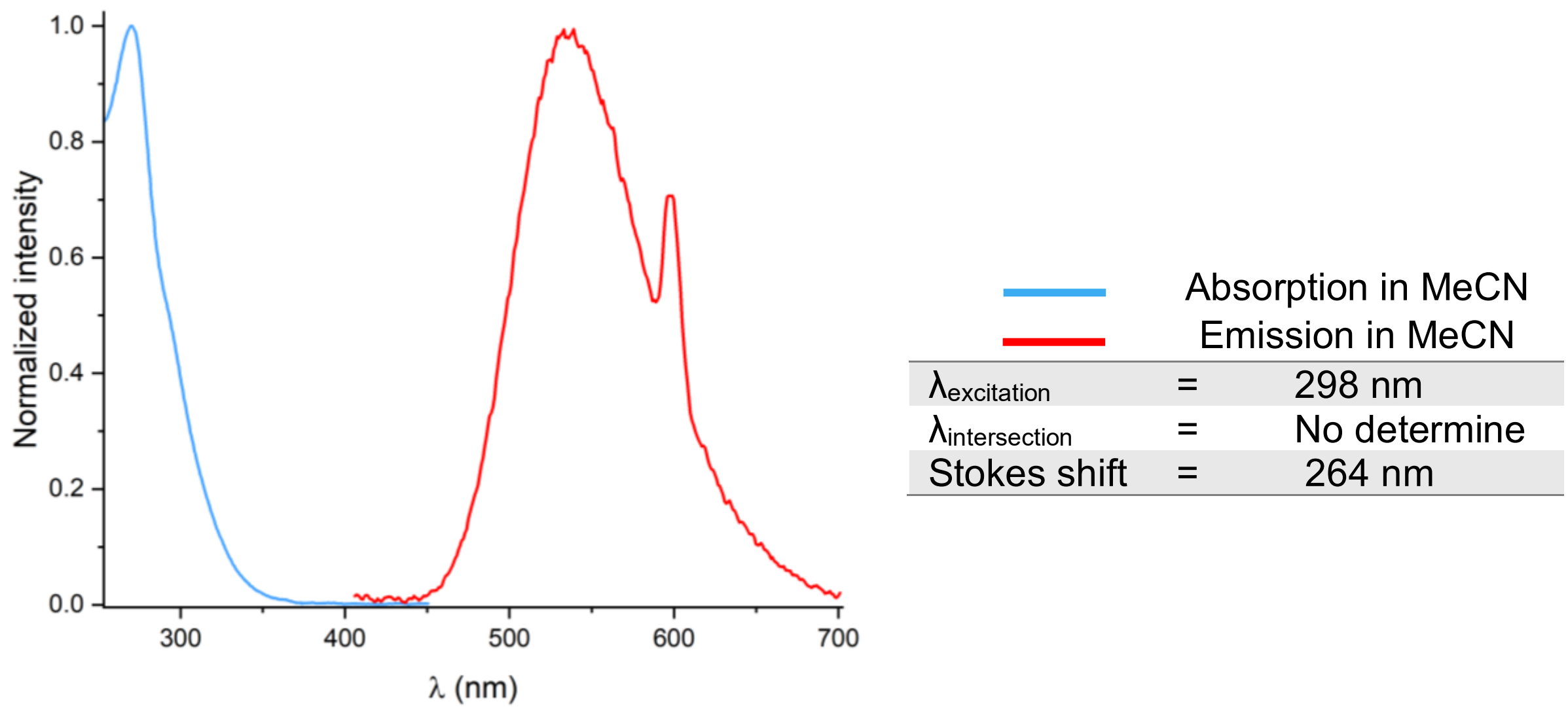

Figure S13: *Normalized optical absorption spectrum (blue trace) and emission spectra (red trace) of **5**.* $\lambda_{excitation}$ = 298 nm.

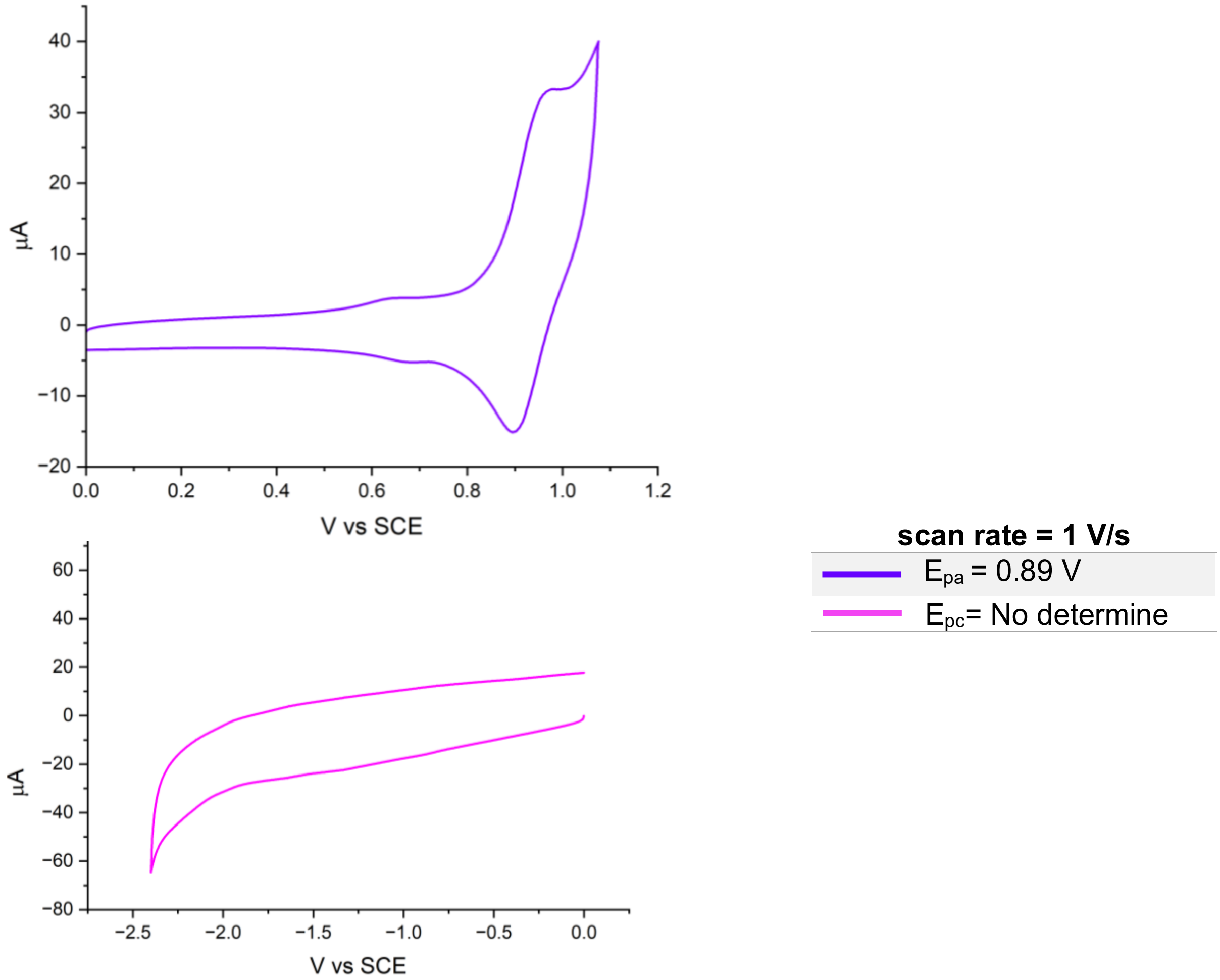

Figure S14*: Cyclic voltammograms of **5** (1mM) in a [Bu_4N][PF_6] (0.1 M) solution in MeCN. The plot follows the IUPAC convention. Glassy carbon electrode; Ag/AgCl (3 M NaCl) reference electrode; Pt counter electrode. For oxidation: Scan rate= 1 V/s; initial potential= 0.0 V; switching potential= 1.07 V; final potential; 0.0 V. For the reduction: Scan rate= 1 V/s. initial potential= 0.0 V; switching potential= -2.4 V; final potential; 0.0 V. Reversibility and irreversibility were determined using different scan rates.*

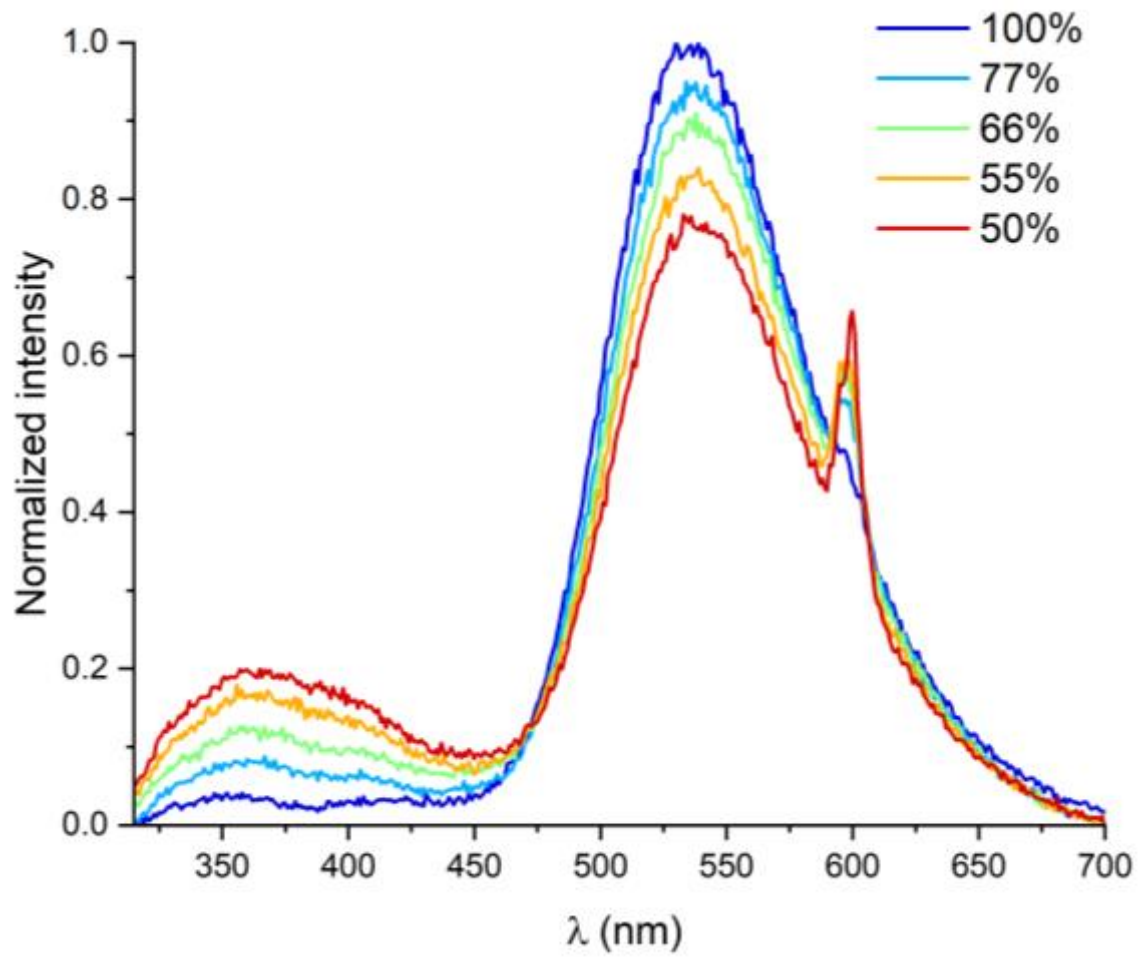

Figure S15: Fluorescence measurements of molecule **5** at 1·10^{-5} M in different volume ratios of methanol/glycerol as a function of viscosity at room temperature. *Slit excitation: 5. Slit emission: 5.* $\lambda_{excitation}$ = 300 nm. Second harmonic is visualized.

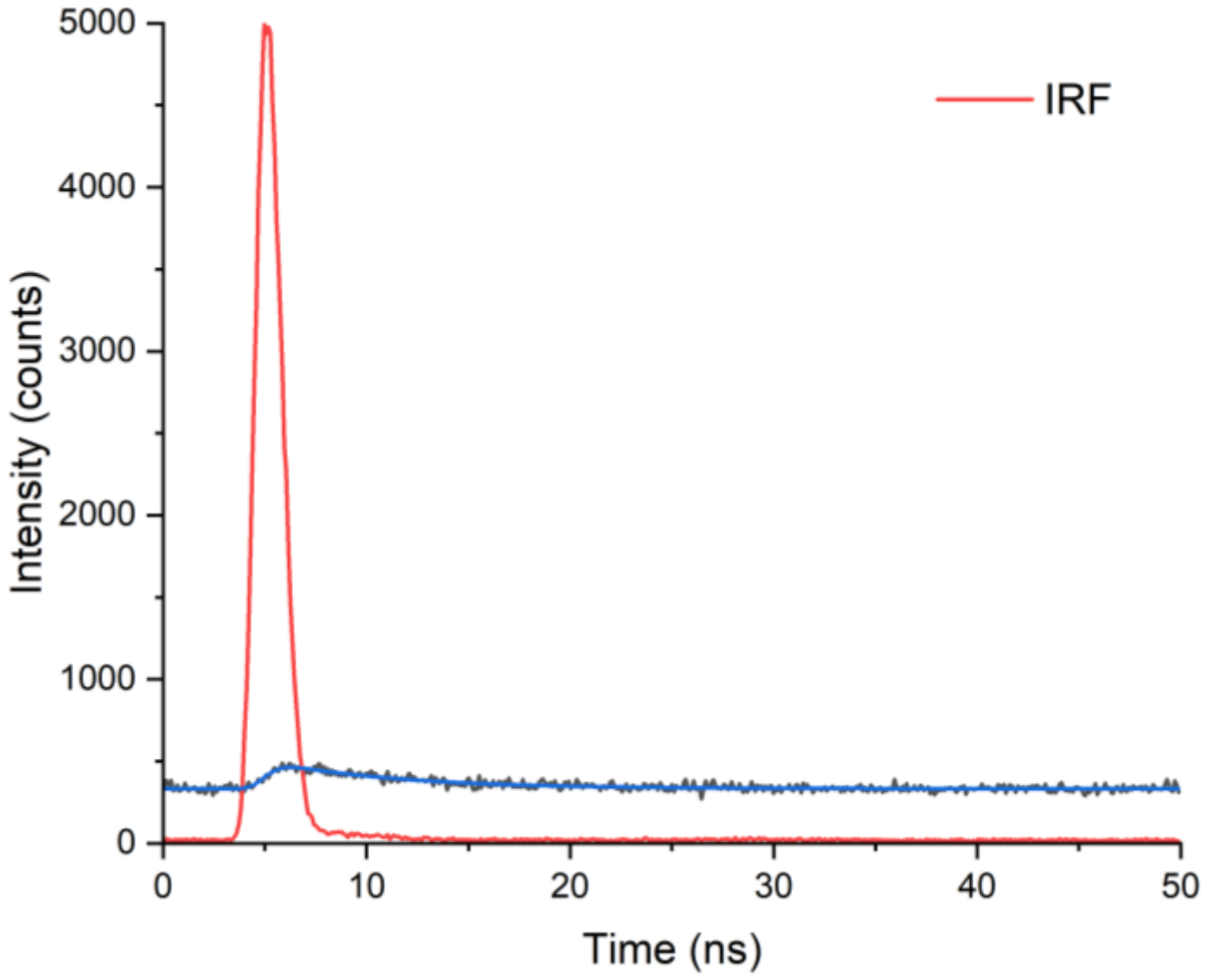

Figure S16*: Time-resolved emission decay at room temperature (excitation at 286.2 nm) of* ***5*** *at 1·10^{-5} M in MeCN solution measured by TC-SPC (τ = 6.1 ns), from deconvolution and single-exponential fitting, X2 = 1.01).*

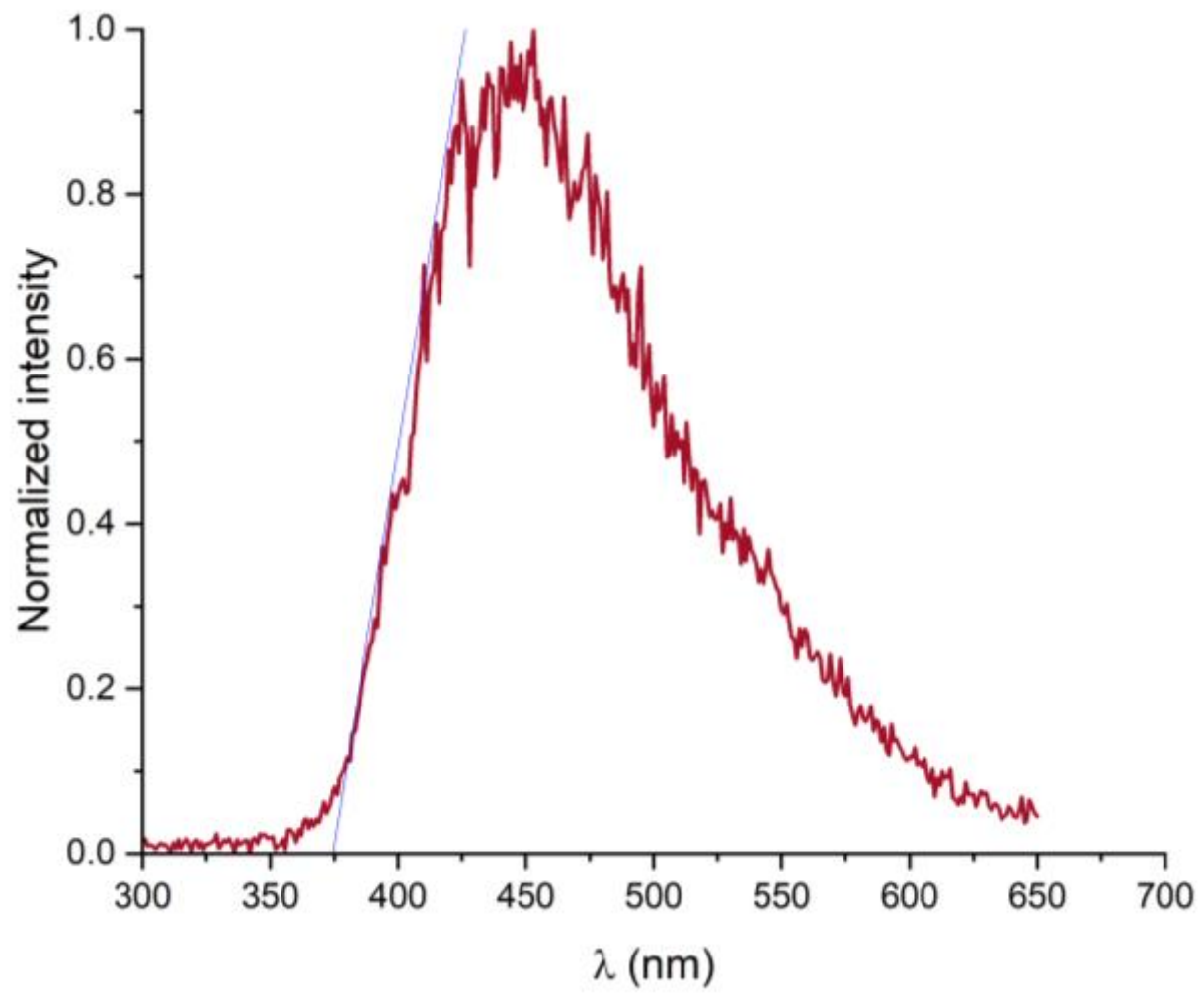

***Figure S17**: Phosphorescence spectrum at 77 K of **5** at 1·10^{-5} M in 2-MeTHF. Excitation at 270 nm, slits=10.*

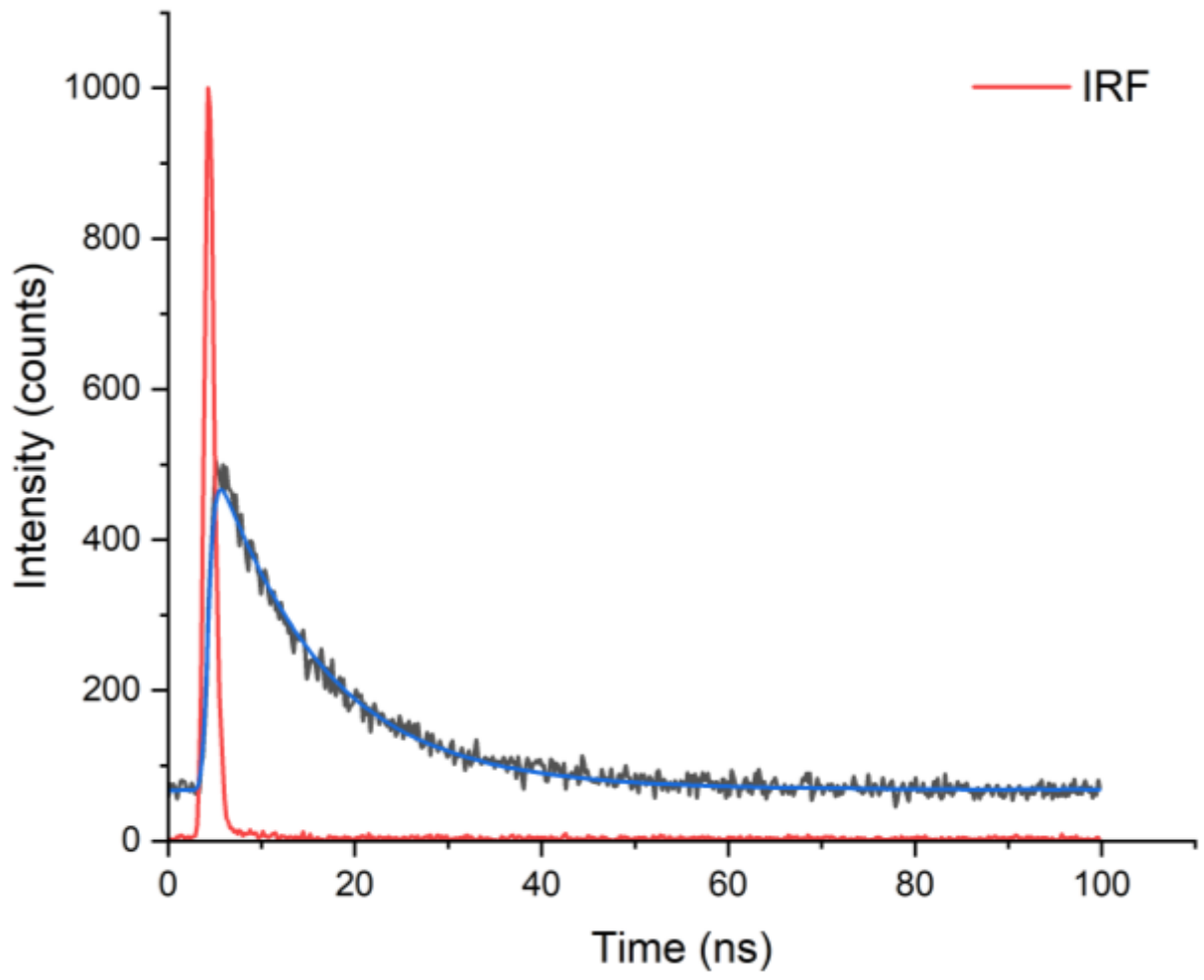

***Figure S18**: Time-resolved emission decay at 77K of **5** at 1·10^{-5} M in 2-MeTHF solution measured by TC-SPC (τ = 11.4 ns), from deconvolution and single-exponential fitting, X2 = 1.01). Excitation: 286.2 nm. Emission: 510 nm.*

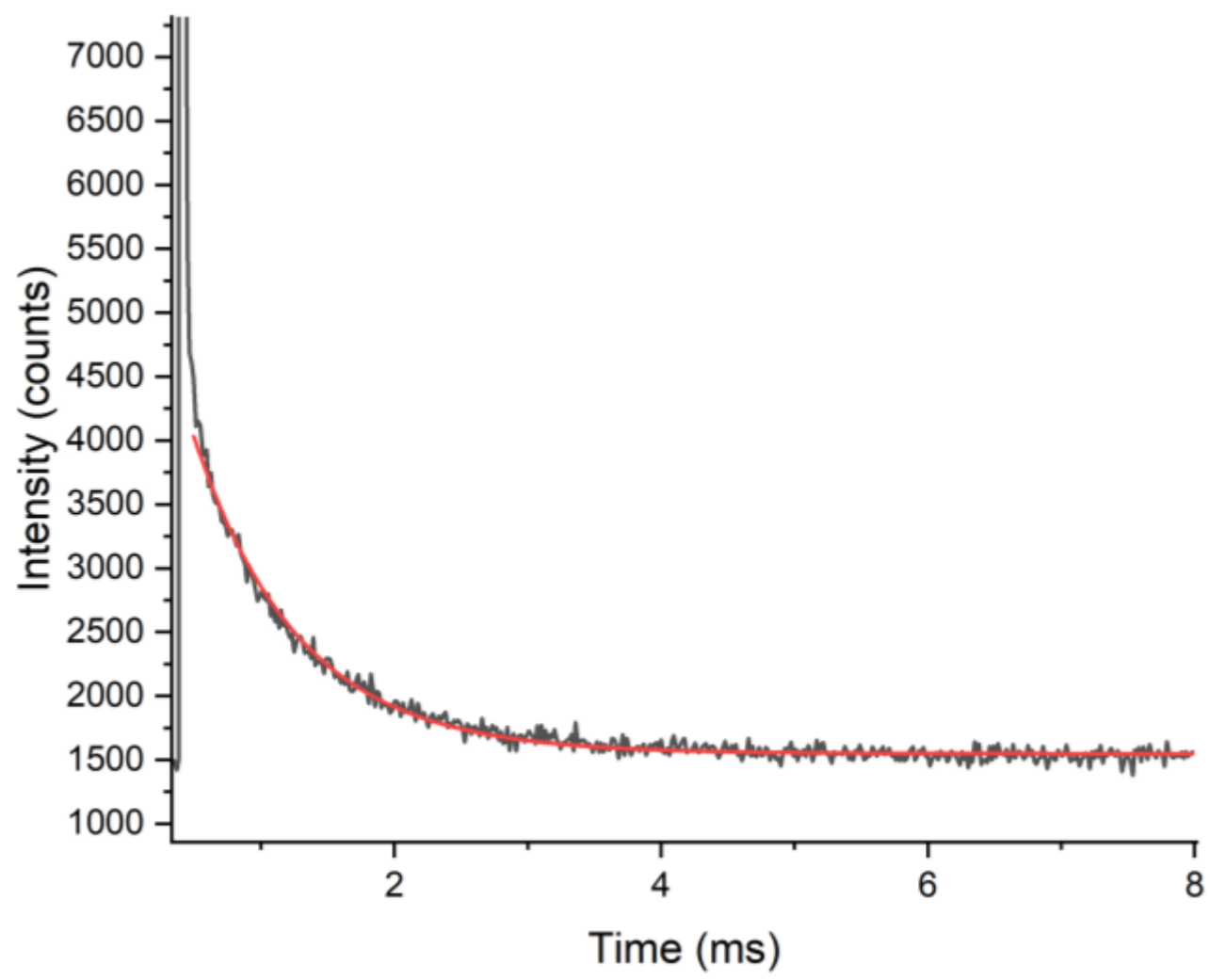

***Figure S19**:* Triplet-state lifetime measurement of **5** *at 1·10^{-5} M* in a frozen MeTHF glass at 77 K, giving τ= 786 μs. *X2 = 1.73. Excitation= 270 nm. Emission: 440 nm.*

6,12-di(naphthalen-2-yl)-9H-tribenzo[*b,d,f*]azepine (**6**)

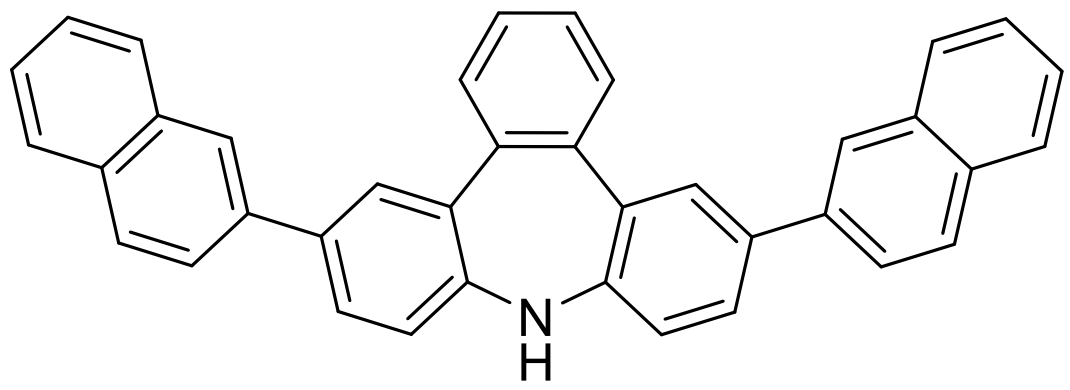

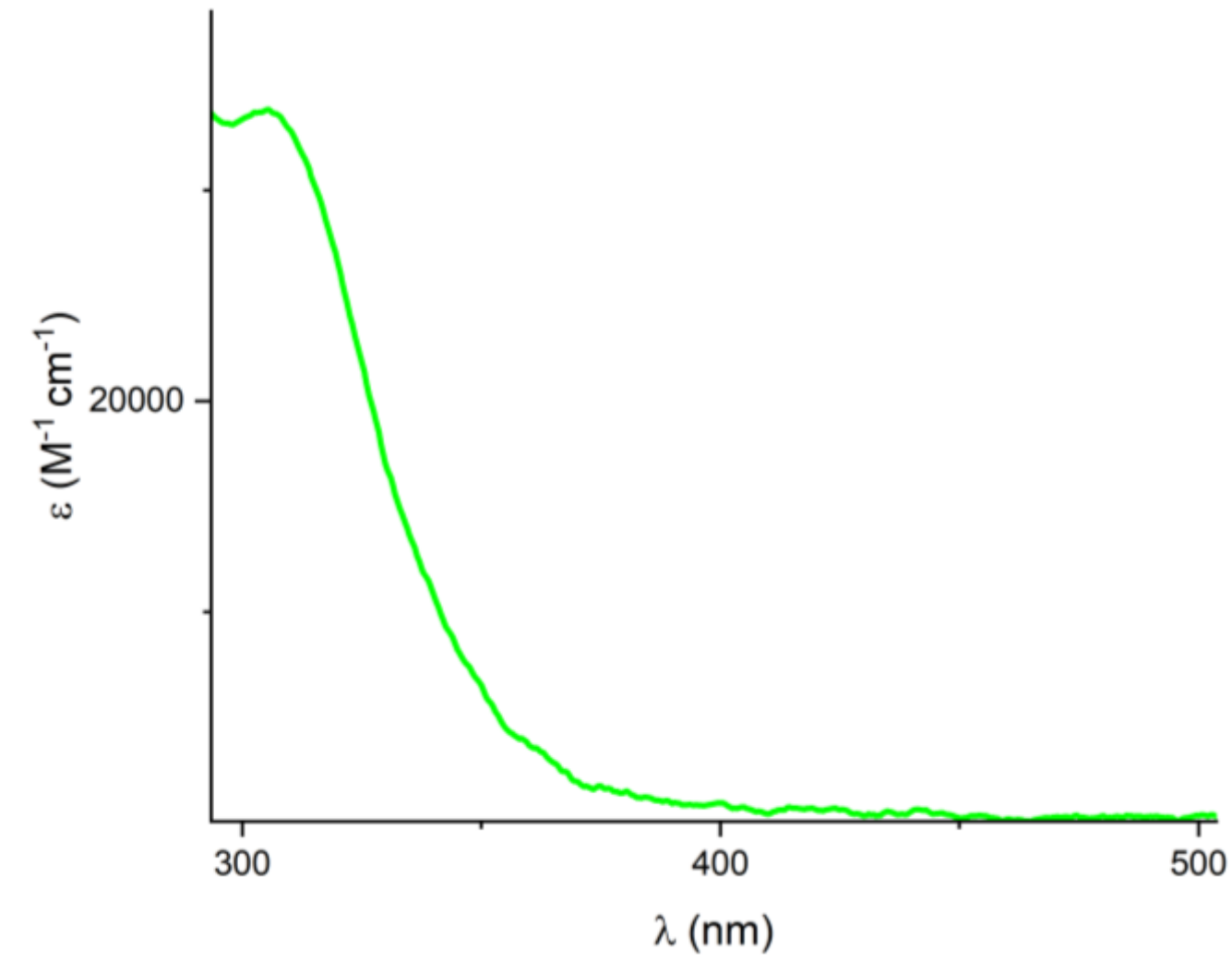

Concentration (mmol/mL)	
	5·10⁻⁶
λ peak	304 nm

Figure S20: *UV-Vis absorption of **6** at 5·10^{-6} M (green trace). Recorded in MeCN in quartz cuvettes (1 cm optical path).*

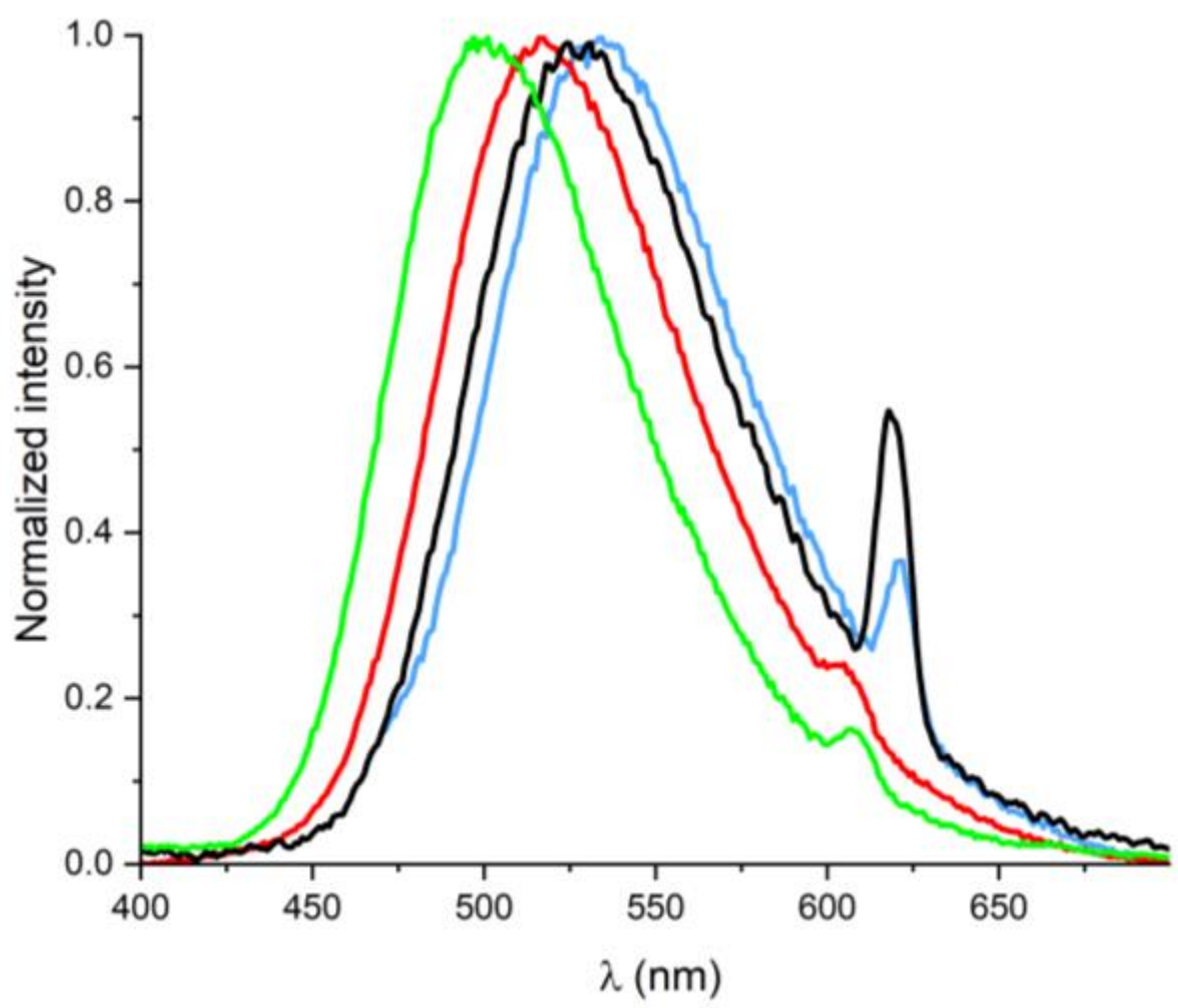

	Solvent	Peak (nm)
	DMF	534
	MeCN	527
	DCM	517
	Cyclohexane	499

Figure S21: *Normalized fluorescence emission of **6** at 1·10-5 M in different solvents. MeCN (black trace); DMF (blue trace); DCM (red trace), Cyclohexane (green trace).*

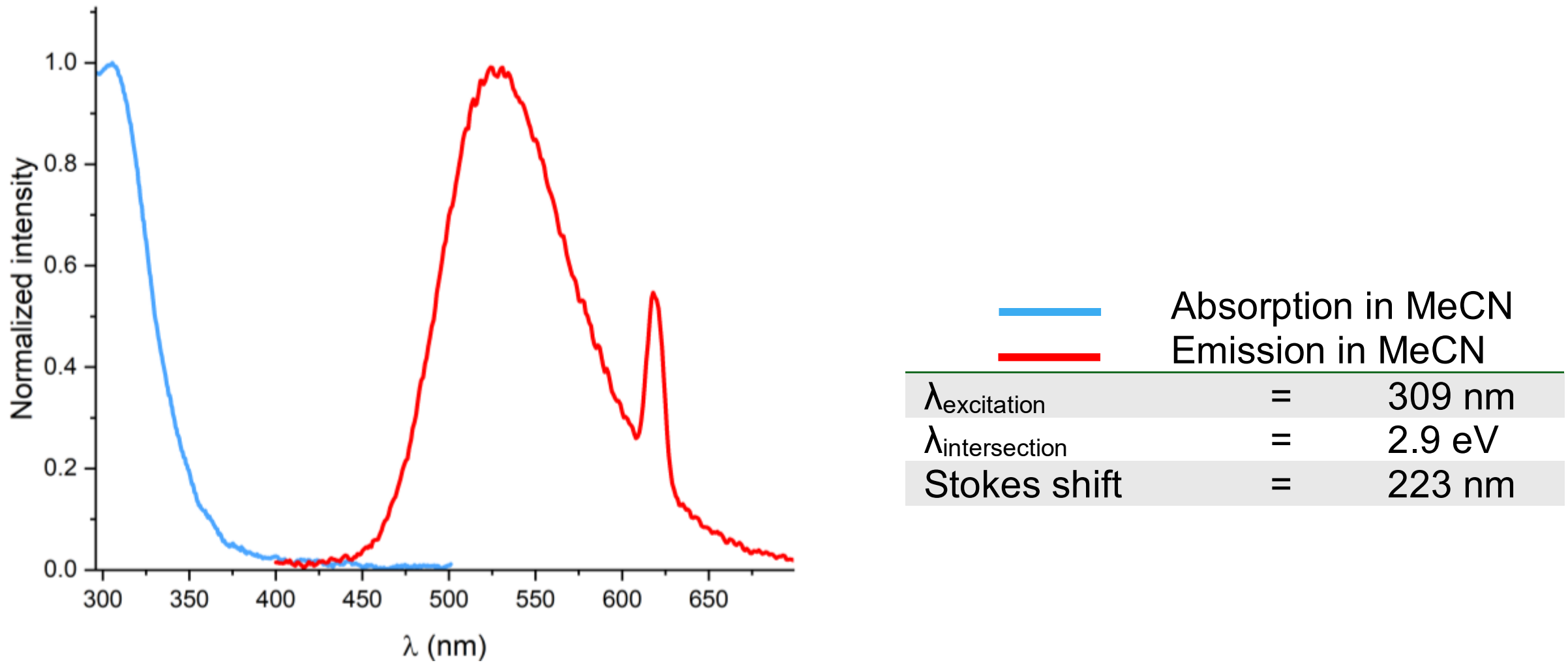

Figure S22*: Normalized optical absorption spectrum (blue trace) and emission spectra (red trace) of* ***6****.* $\lambda_{excitation}$ = 309 nm.

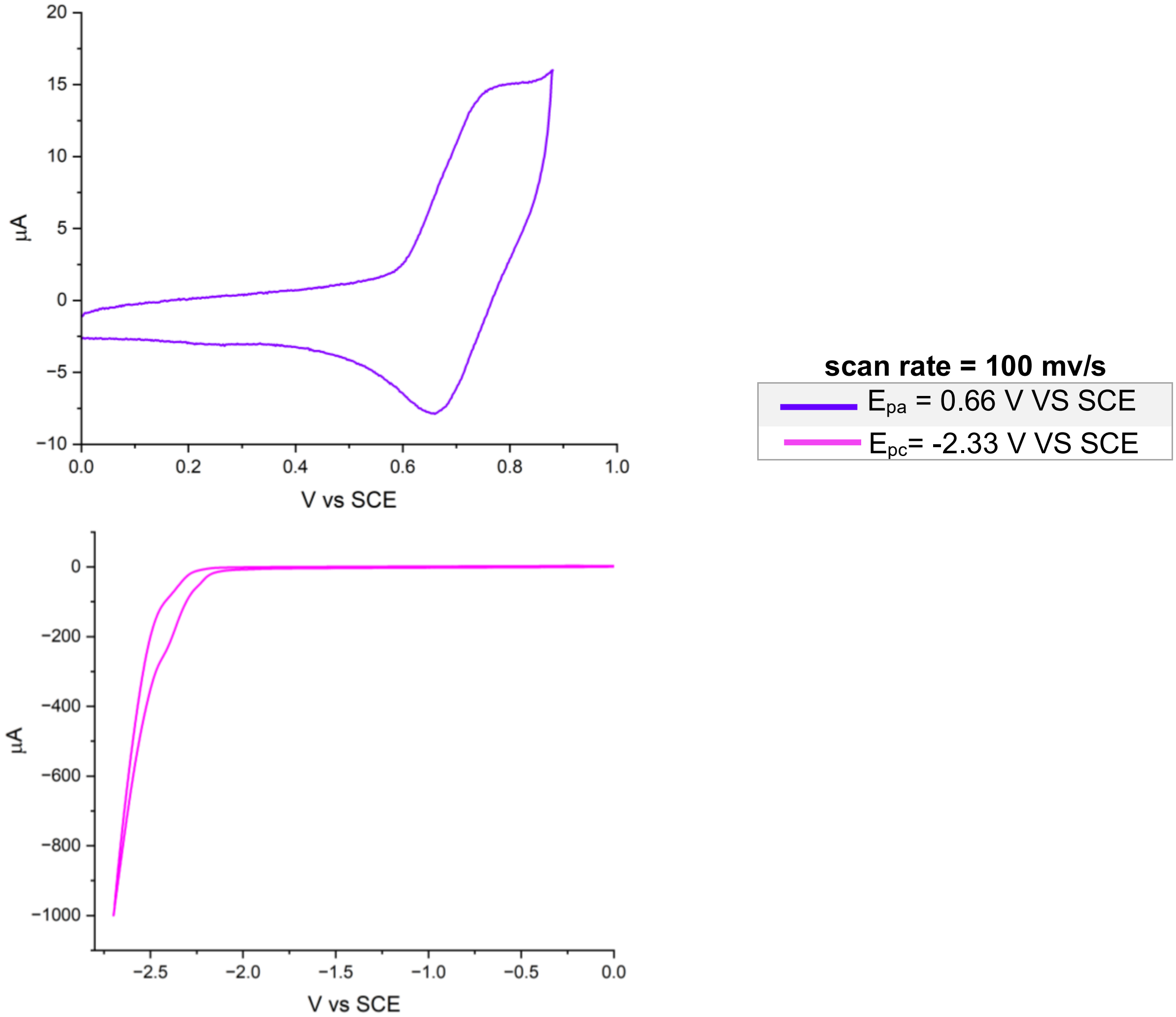

Figure S23*: Cyclic voltammograms of* ***6*** *(1mM) in a [Bu_4N][PF_6] (0.1 M) solution in MeCN. The plot follows the IUPAC convention. Glassy carbon electrode; Ag/AgCl (3 M NaCl) reference electrode; Pt counter electrode. For oxidation: Scan rate= 100 mV/s; initial potential= 0.0 V; switching potential= 0.88 V; final potential; 0.0 V. For the reduction: Scan rate= 100 mV/s. initial potential= 0.0 V; switching potential= -2.7 V; final potential; 0.0 V. Reversibility and irreversibility were determined using different scan rates.*

.

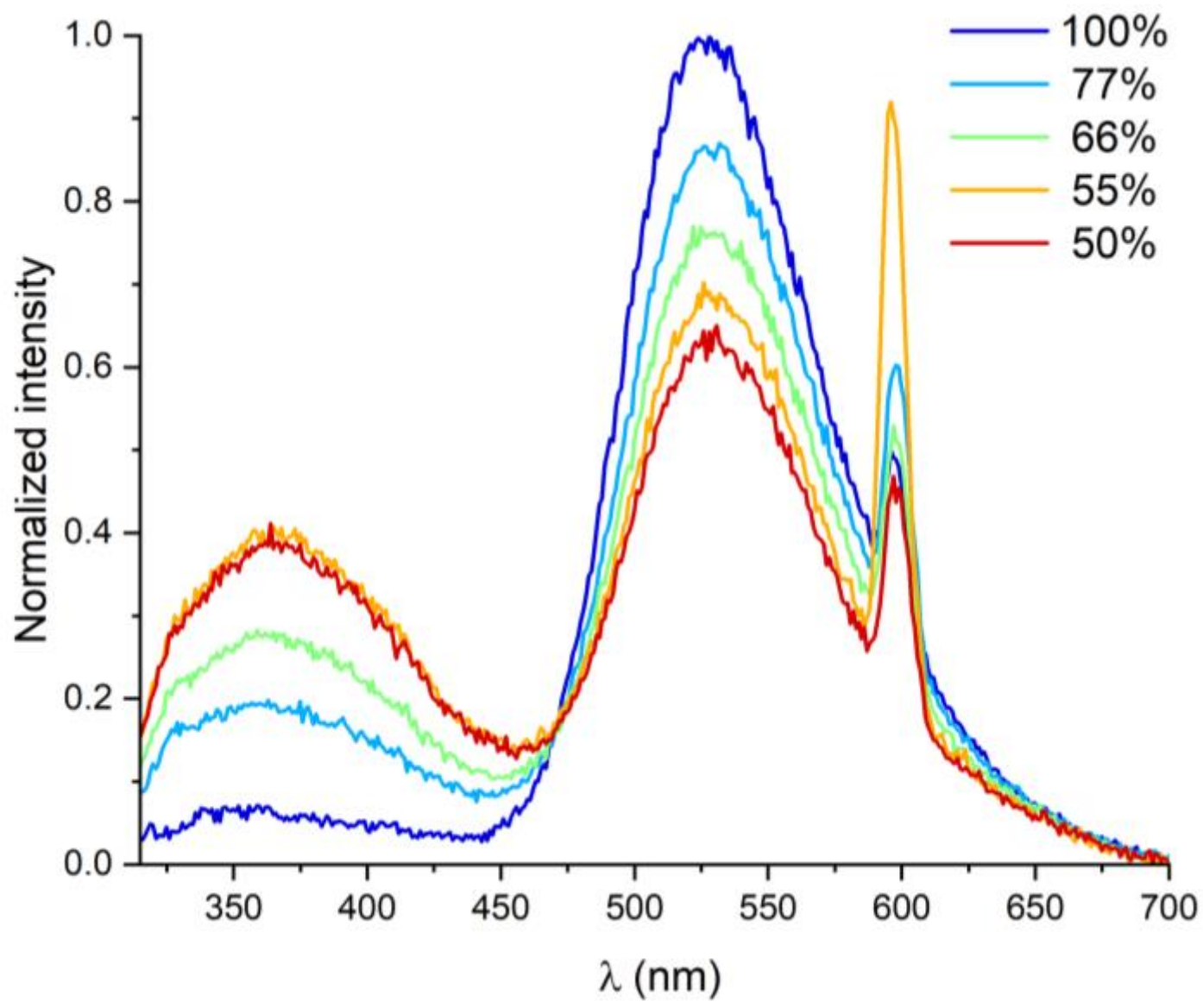

Figure S24: *Fluorescence measurements of molecule **6** at $1{\cdot}10^{-5}$ M in different volume ratios of methanol/glycerol as a function of viscosity at room temperature. Slit excitation: 5. Slit emission: 5. $\lambda_{excitation}$ = 300 nm. Second harmonic is visualized.*

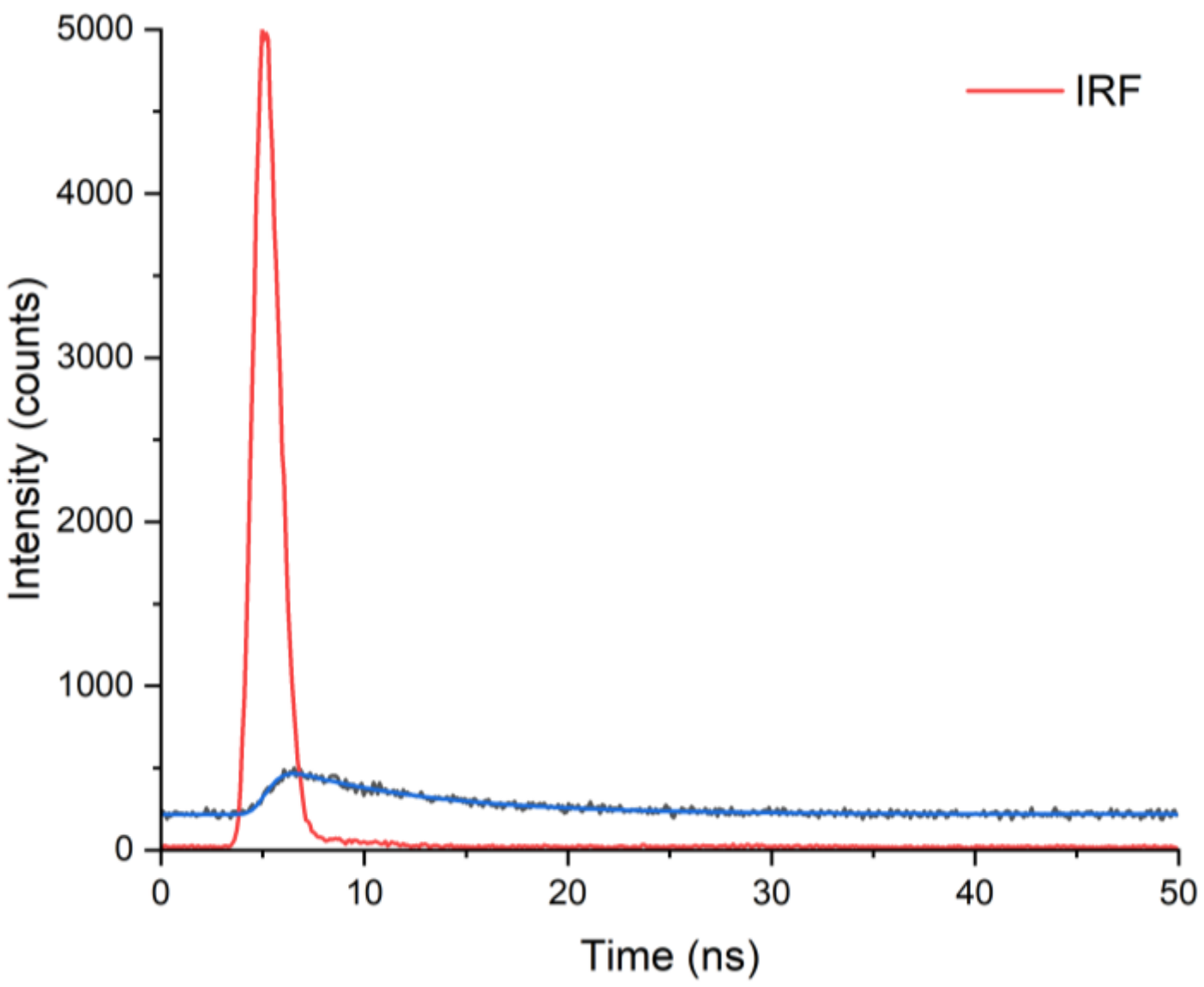

Figure S25: *Time-resolved emission decay at room temperature (excitation at 286.2 nm) of **6** at $1{\cdot}10^{-5}$ M in MeCN solution measured by TC-SPC (τ = 6.9 ns), from deconvolution and single-exponential fitting, X2 = 0.94).*

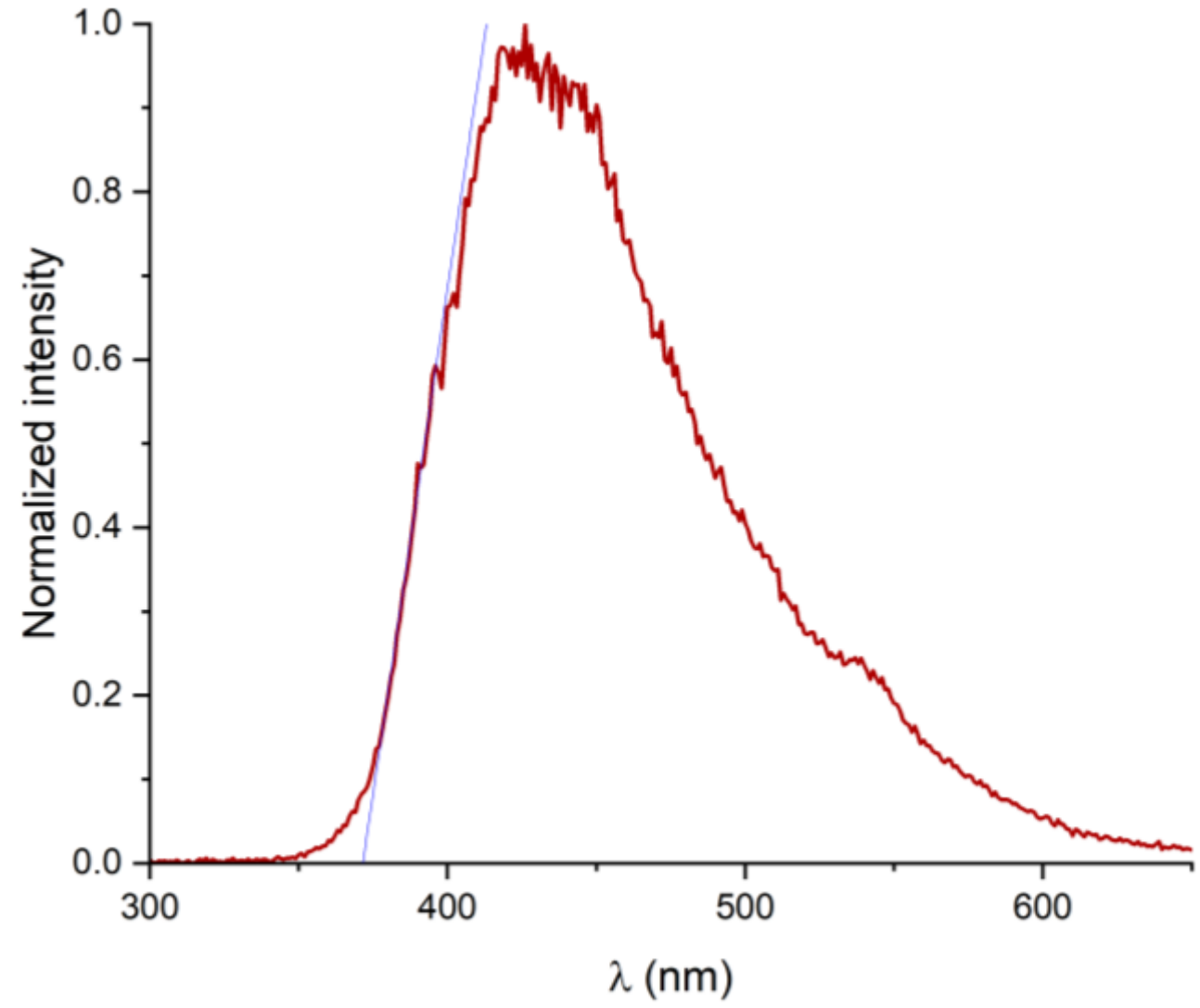

Figure S26: *Phosphorescence spectrum at 77 K of* ***6*** *at 1·10^{-5} M in 2-MeTHF. Excitation at 270 nm, slits=10.*

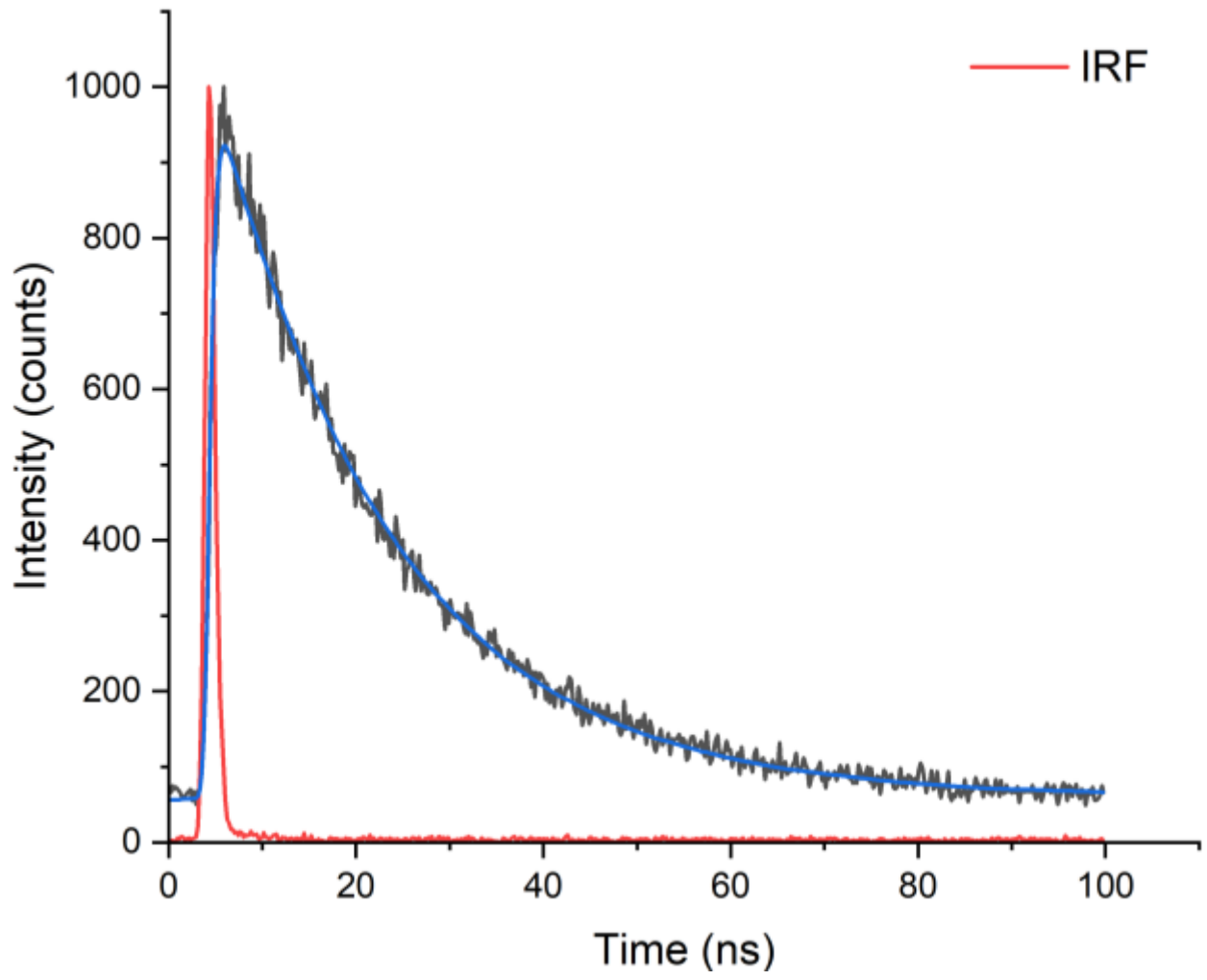

Figure S27: *Time-resolved emission decay at 77K of* ***6*** *at 1·10^{-5} M in 2-MeTHF solution measured by TC-SPC (τ = 18.5 ns), from deconvolution and single-exponential fitting, X2 = 1.08). Excitation at 286.2 nm. Emission: 520 nm.*

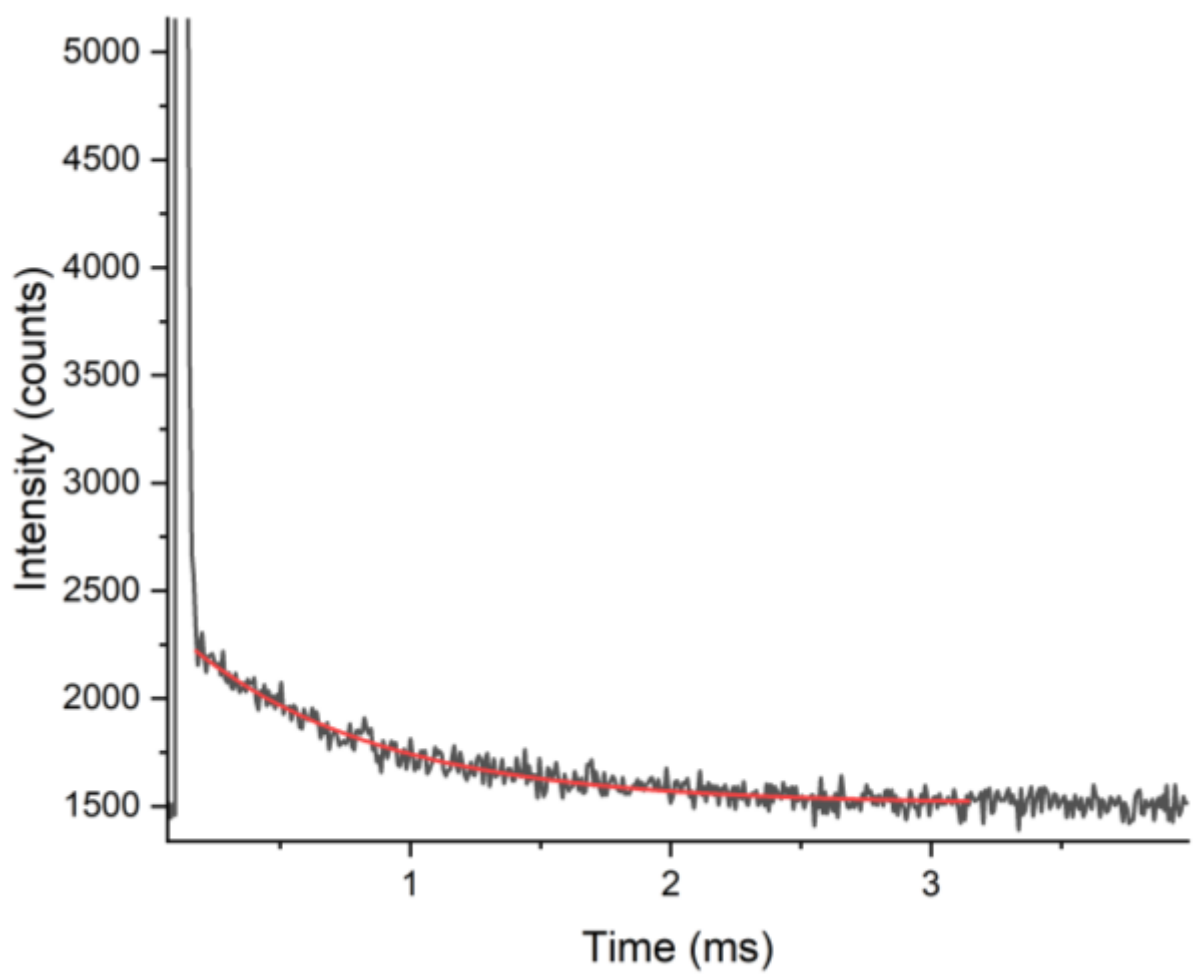

Figure S28: *Triplet-state lifetime measurement of* ***6*** *at 1·10^{-5} M in a frozen MeTHF glass at 77 K, giving τ= 736 μs. X2 = 1.19. Excitation: 303 nm. Emission: 440 nm.*

4,4'-(9H-tribenzo[*b,d,f*]azepine-6,12-diyl)dibenzonitrile (**7**)

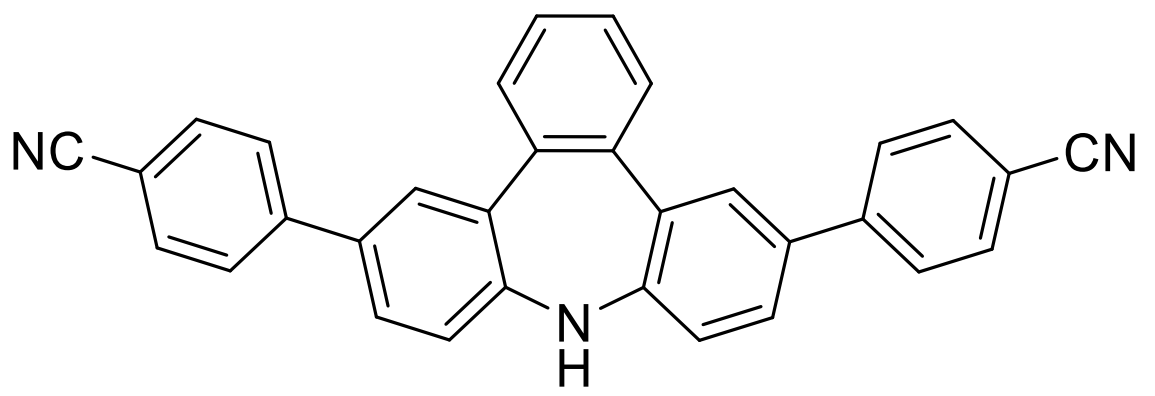

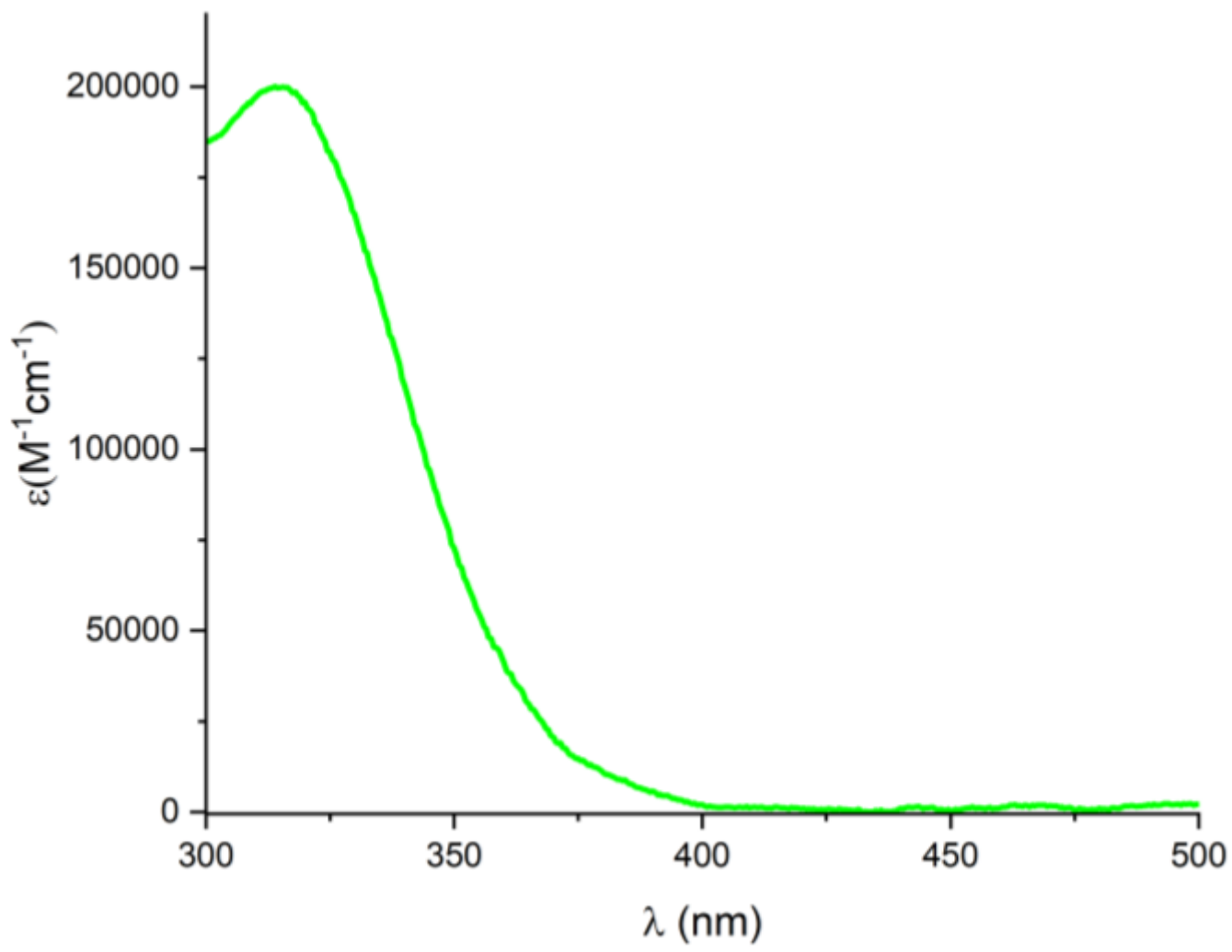

Concentration (mmol/mL)	
	5·10⁻⁶
λ peak	315 nm

Figure S29: *UV-Vis absorption of **7** at 5·10^{-6} M. Recorded in MeCN in quartz cuvettes (1 cm optical path).*

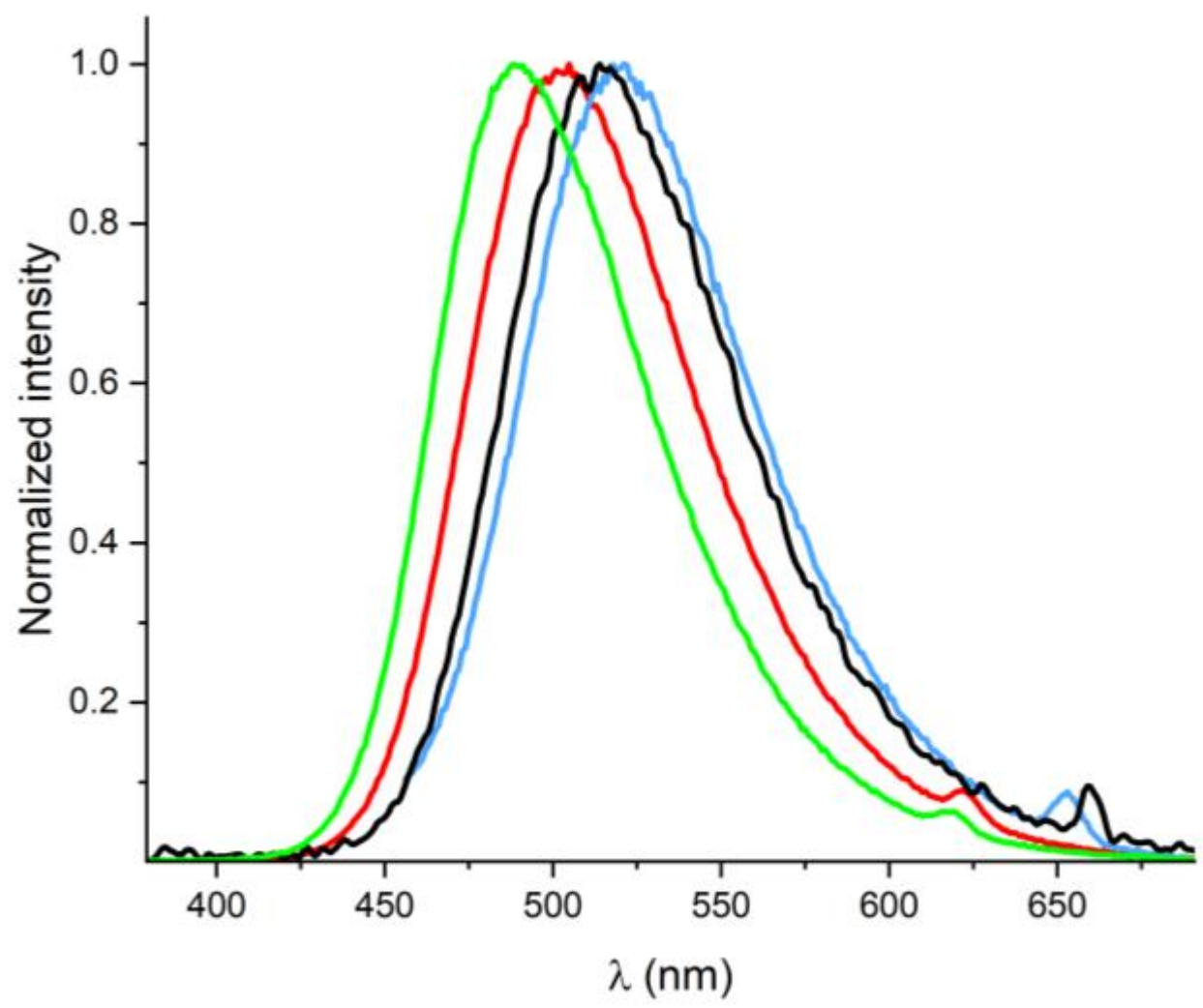

	**Solvent**	**Peak (nm)**
	DMF	519
	MeCN	512
	DCM	502
	Cyclohexane	488

Figure S30: *Normalized fluorescence emission of **7** at 5·10^{-6} M in different solvents. DMF (blue trace); MeCN (black trace); DCM (red trace); Cyclohexane (green trace).*

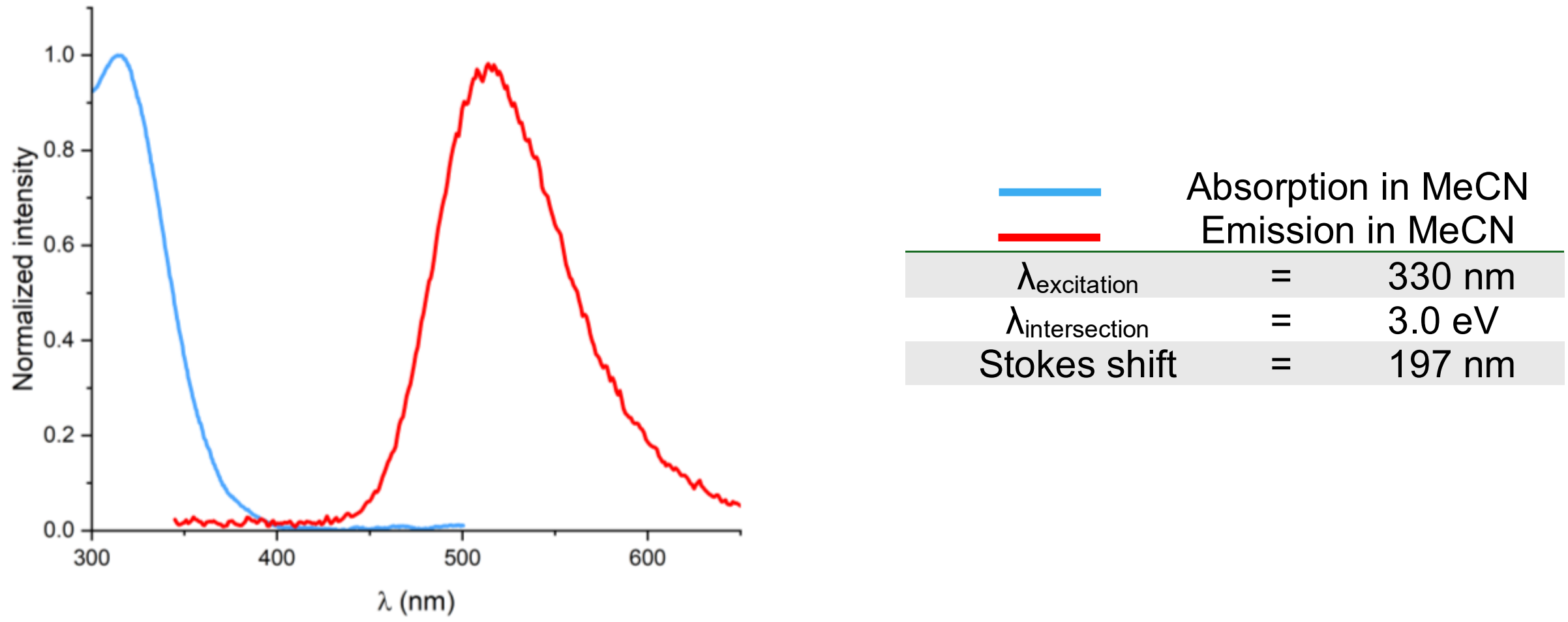

Figure S31: *Normalized optical absorption spectrum (blue trace) and emission spectra (red trace) of **7**.* $\lambda_{excitation}$ = 330 nm.

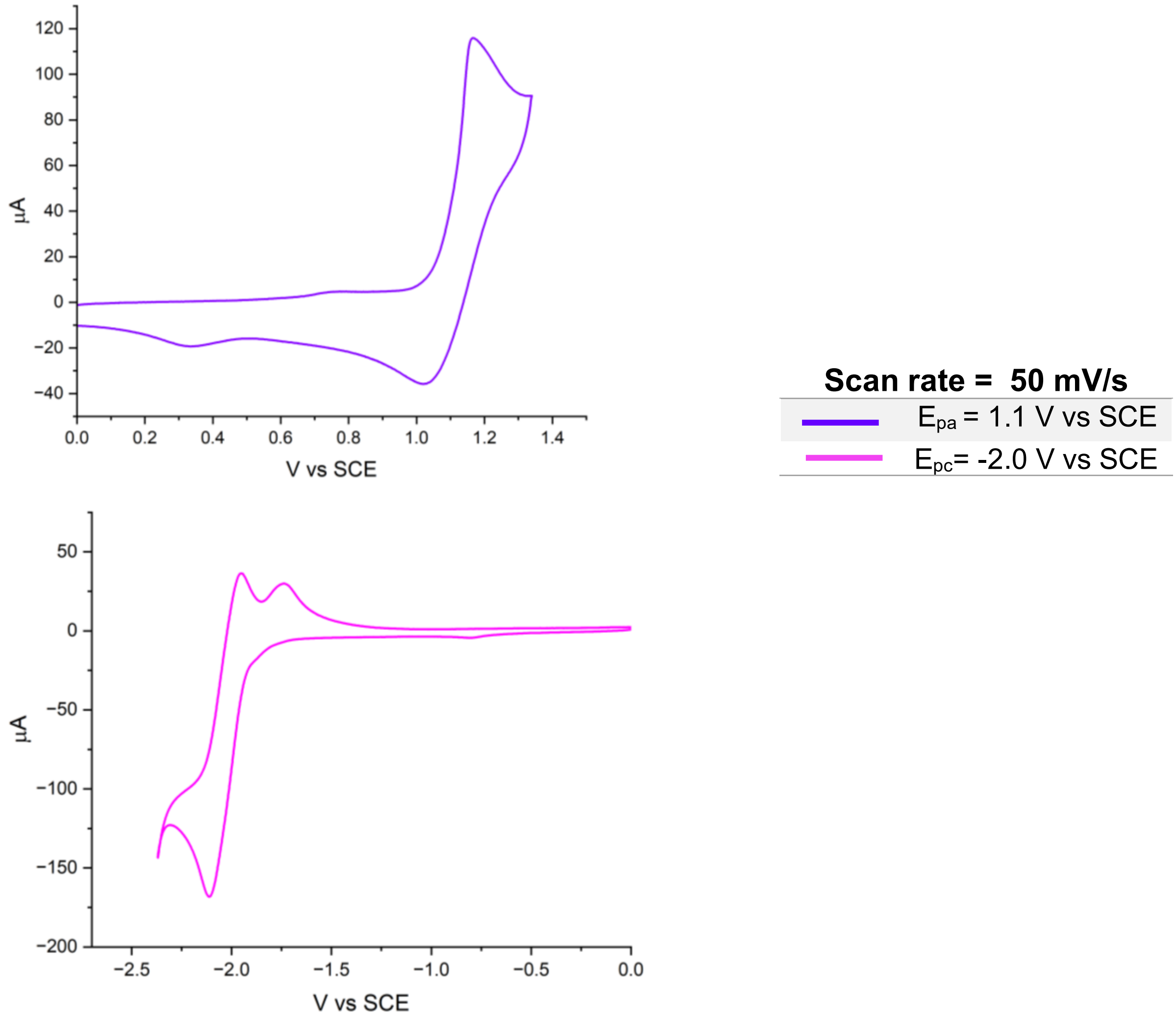

Figure S32: *Cyclic voltammograms of **7** (1mM) in a [Bu_4N][PF_6] (0.1 M) solution in MeCN. The plot follows the IUPAC convention. Glassy carbon electrode; Ag/AgCl (3 M NaCl) reference electrode; Pt counter electrode. For oxidation: Scan rate= 50 mV/s; initial potential= 0.0 V; switching potential= 1.34 V; final potential; 0.0 V. For the reduction: Scan rate= 50 mV/s. initial potential= 0.0 V; switching potential= -2.4 V; final potential; 0.0 V. Reversibility and irreversibility were determined using different scan rates.*

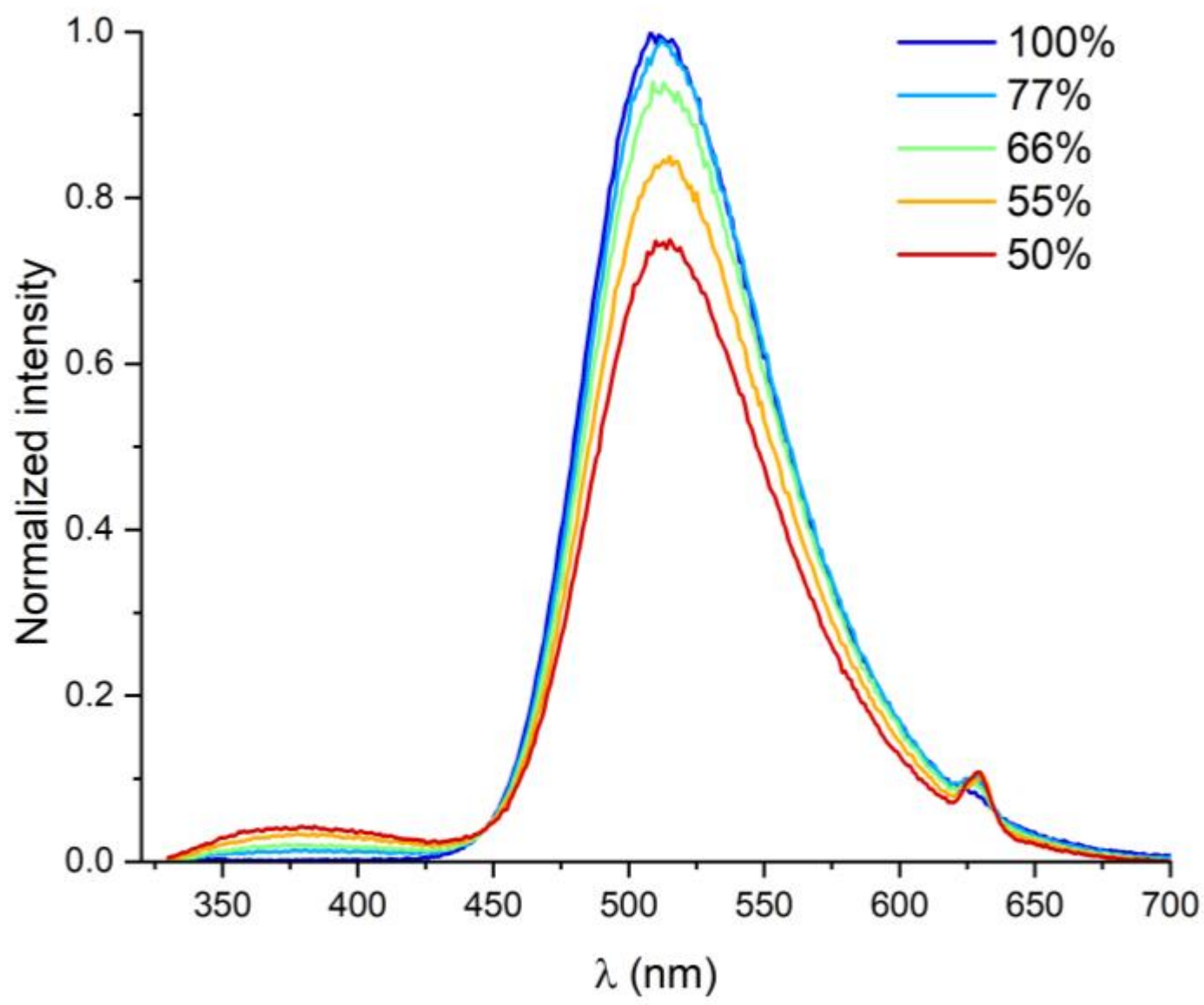

Figure S33: *Fluorescence measurements of molecule **7** at $1{\cdot}10^{-5}$ M in different volume ratios of methanol/glycerol as a function of viscosity at room temperature. Slit excitation: 5. Slit emission: 5. $\lambda_{excitation}$ = 315 nm*

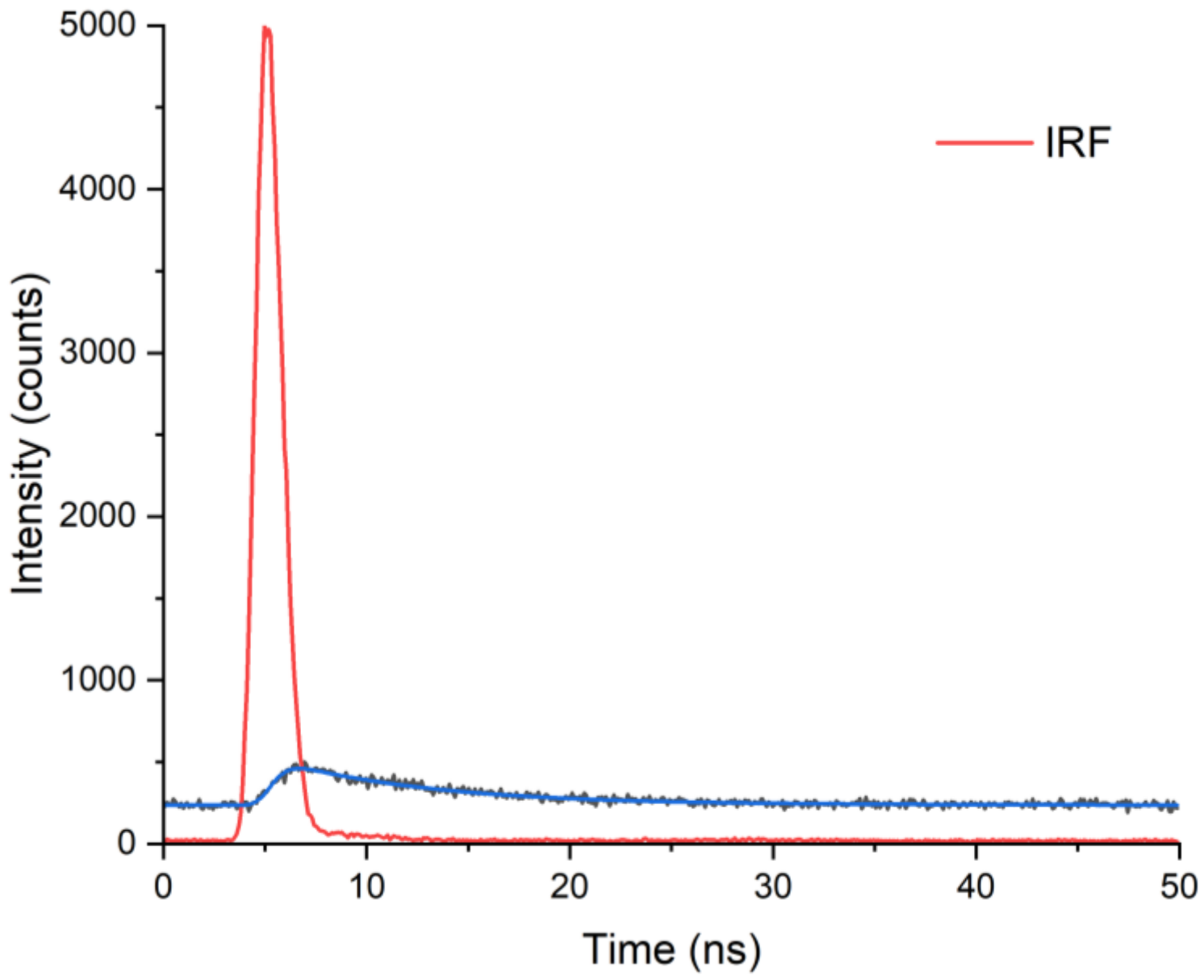

Figure S34: *Time-resolved emission decay at room temperature (excitation at 286.2 nm) of **7** $1{\cdot}10^{-5}$ M in MeCN solution measured by TC-SPC (τ =7.37 ns), from deconvolution and single-exponential fitting, X2 = 1.07).*

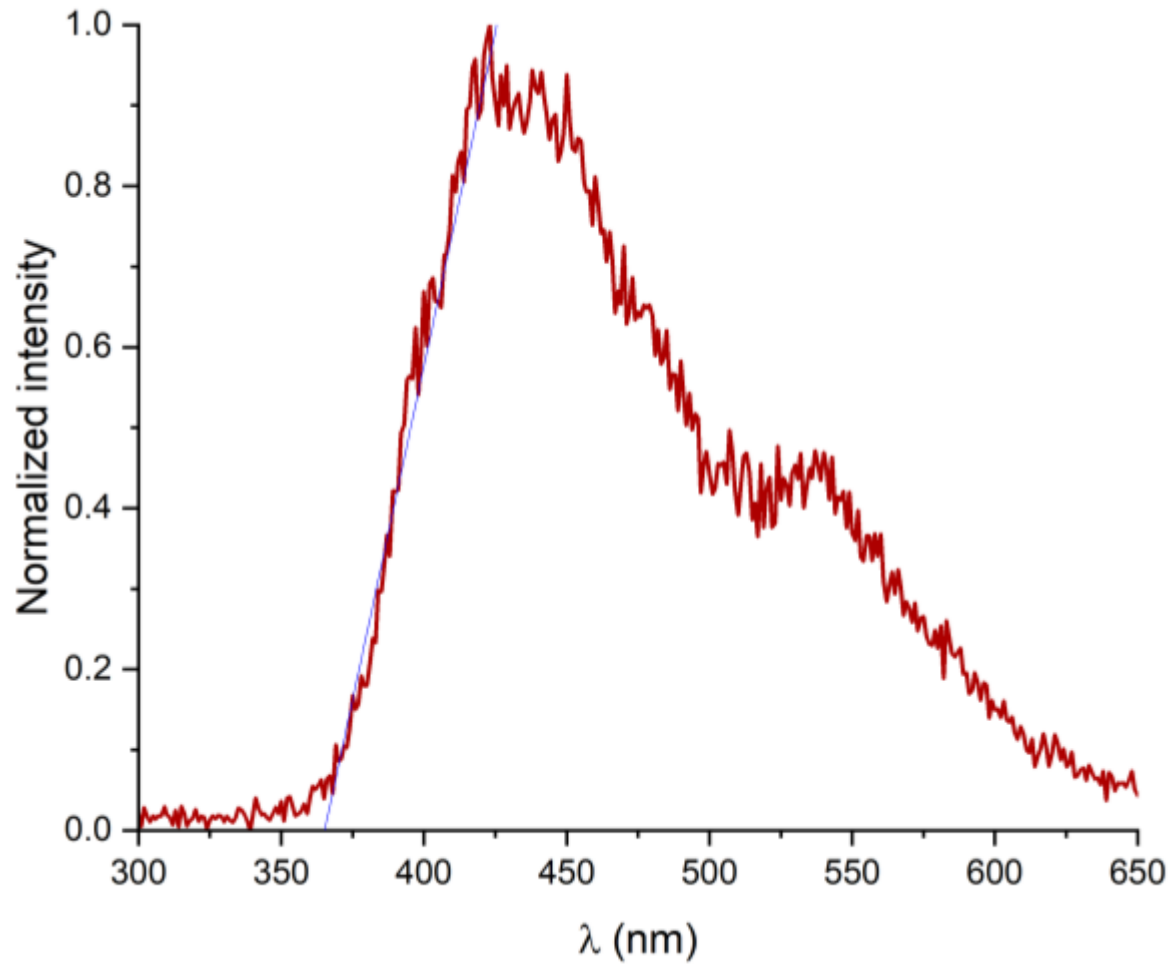

Figure S35: *Phosphorescence spectrum at 77 K of **7** at 1·10^{-5} M in 2-MeTHF. Excitation at 270 nm, slits=10.*

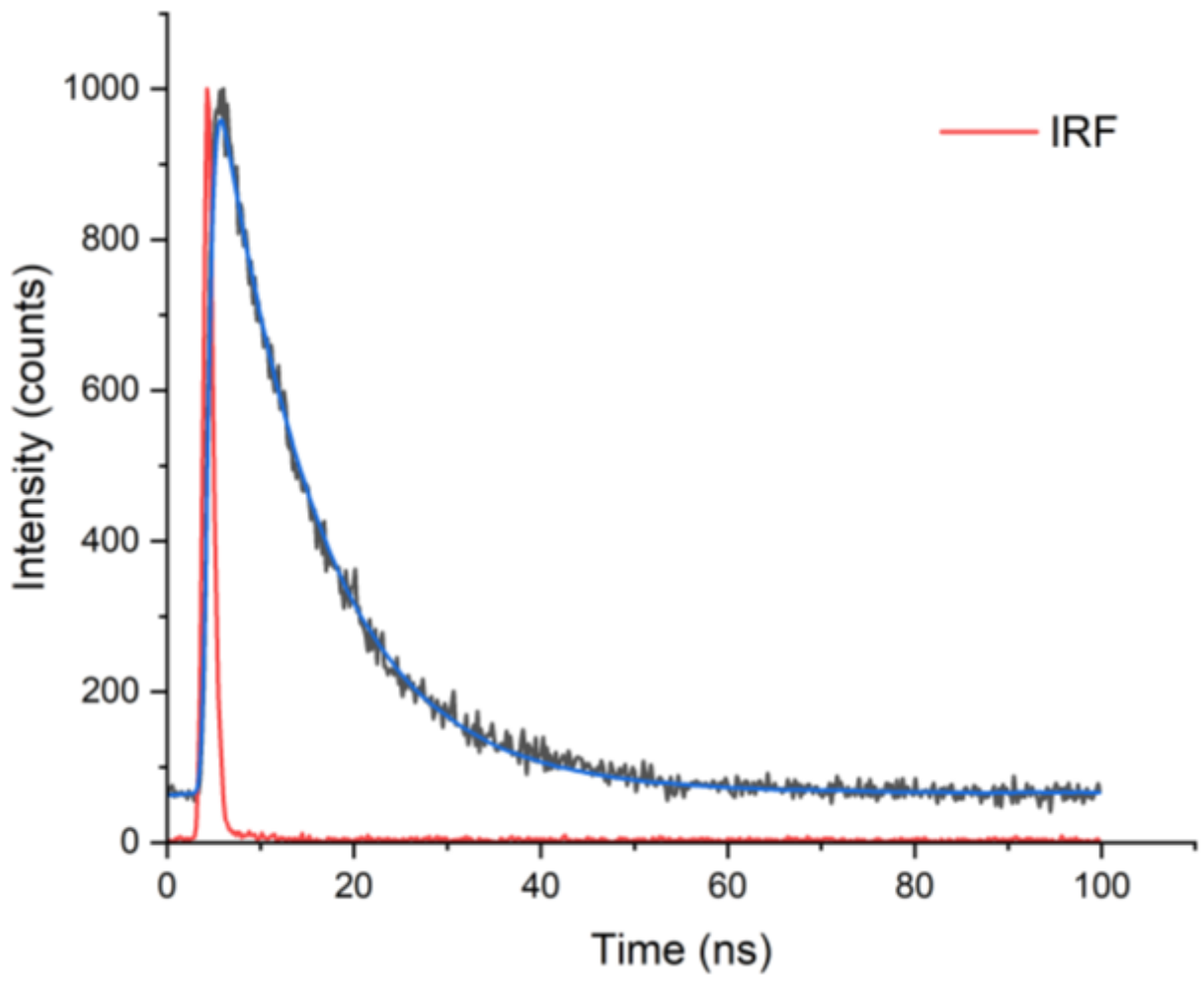

Figure S36: *Time-resolved emission decay at 77K of **7** at 1·10^{-5} M in 2-MeTHF solution measured by TC-SPC (τ=10.7 ns), from deconvolution and single-exponential fitting, X2 = 1.1). Excitation at 286.2 nm. Emission:550 nm.*

6,12-bis(4-(trifluoromethyl)phenyl)-9H-tribenzo[*b*,*d*,*f*]azepine (**8**)

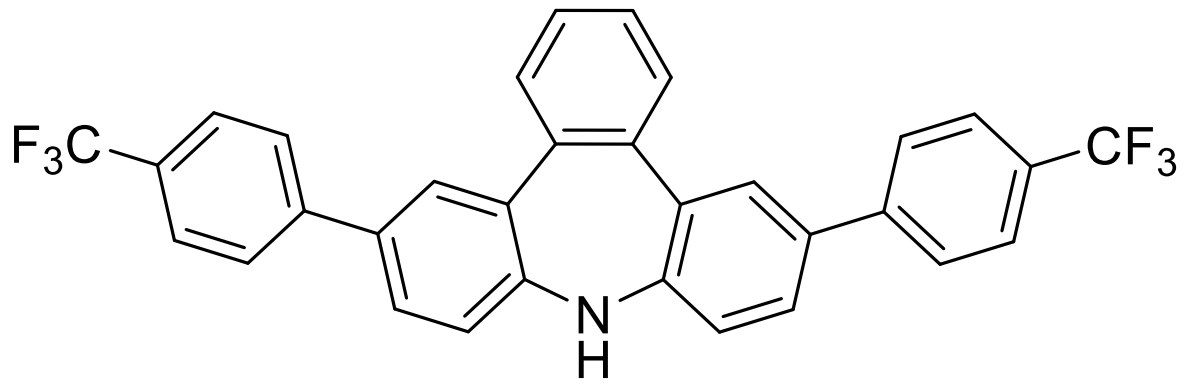

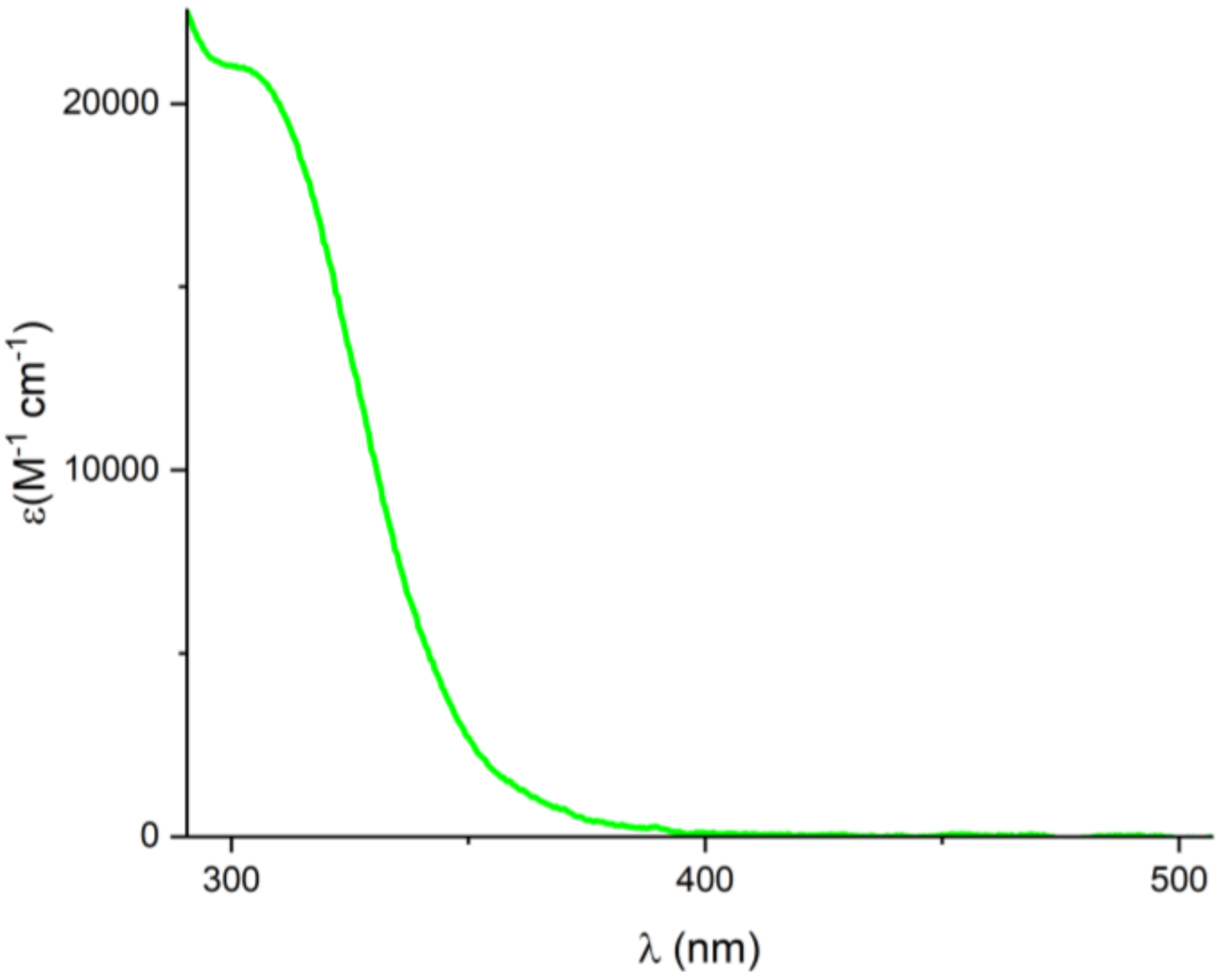

Concentration (mmol/mL)	
(green line)	*1·10⁻⁵*
**λ peak**	**303 nm**

Figure S37*: UV-Vis absorption of* ***8*** *at 1·10^{-5} M (green trace). Recorded in MeCN in quartz cuvettes (1 cm optical path).*

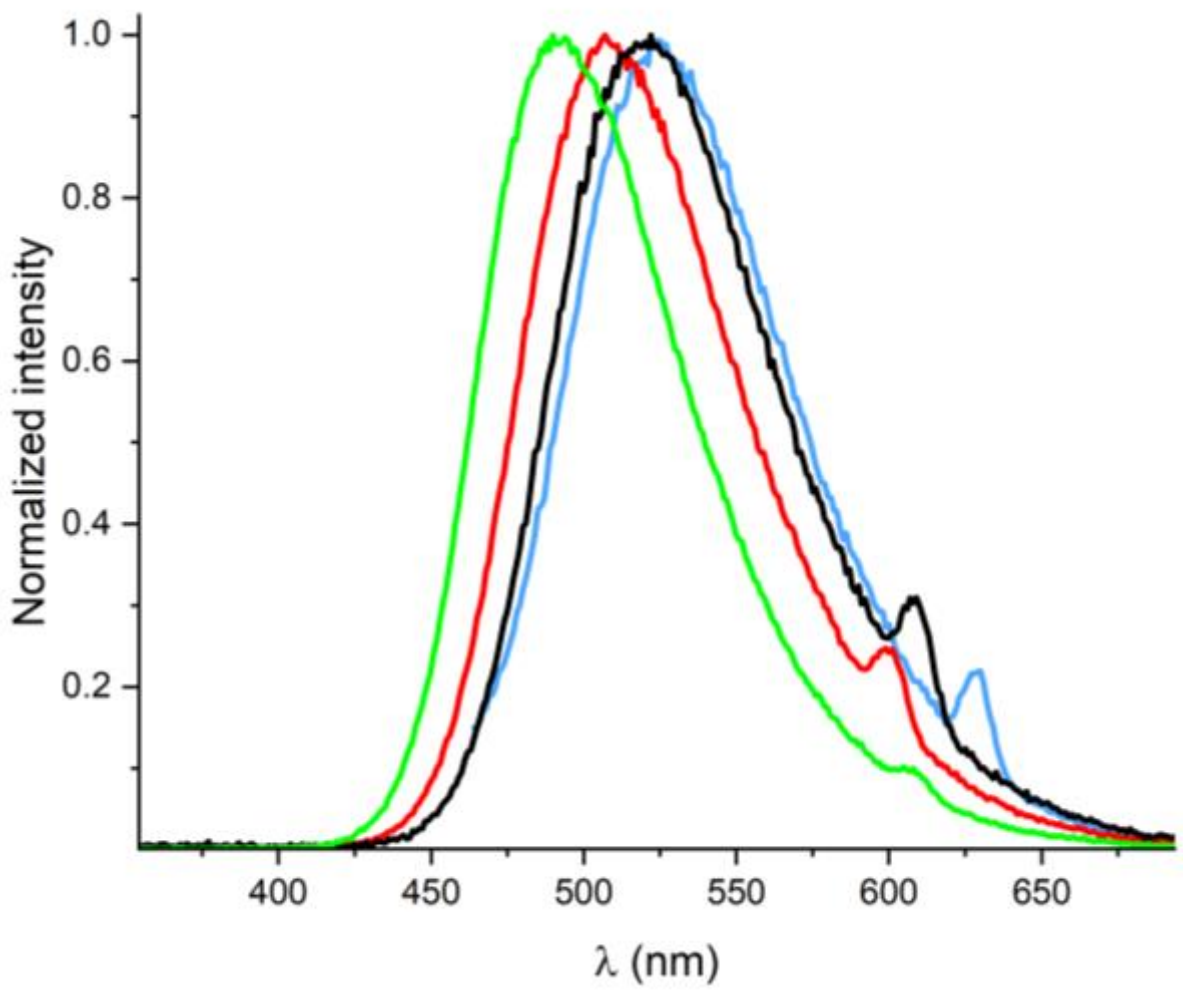

	Solvent	Peak (nm)
(blue line)	DMF	524
(black line)	MeCN	520
(red line)	DCM	506
(green line)	Cyclohexane	492

Figure S38*: Normalized fluorescence emission of* ***8*** *at 1·10^{-5} M in different solvents. DMF (blue trace); MeCN (black trace); DCM (red trace), Cyclohexane (green trace).*

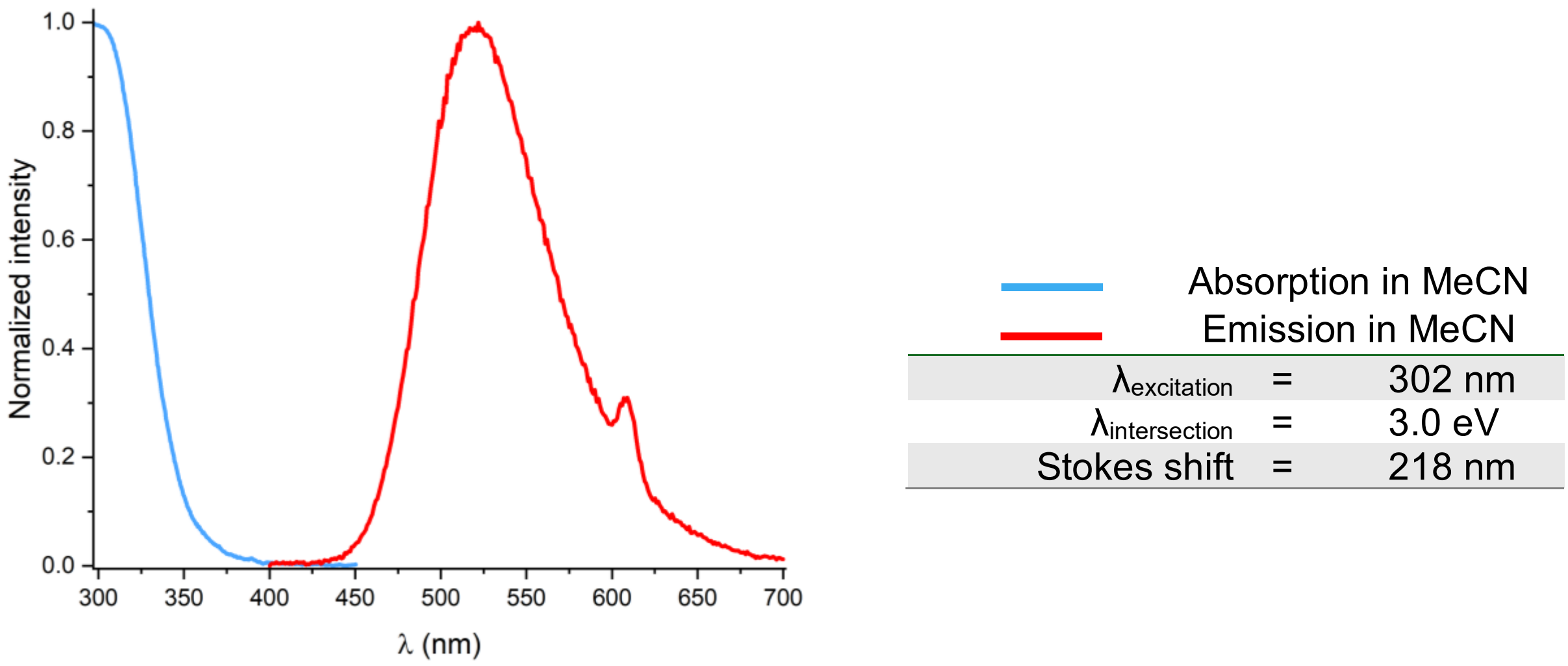

Figure S39: *Normalized optical absorption spectrum (blue trace) and emission spectra (red trace) of* ***8****. λexcitation* = 302 nm.

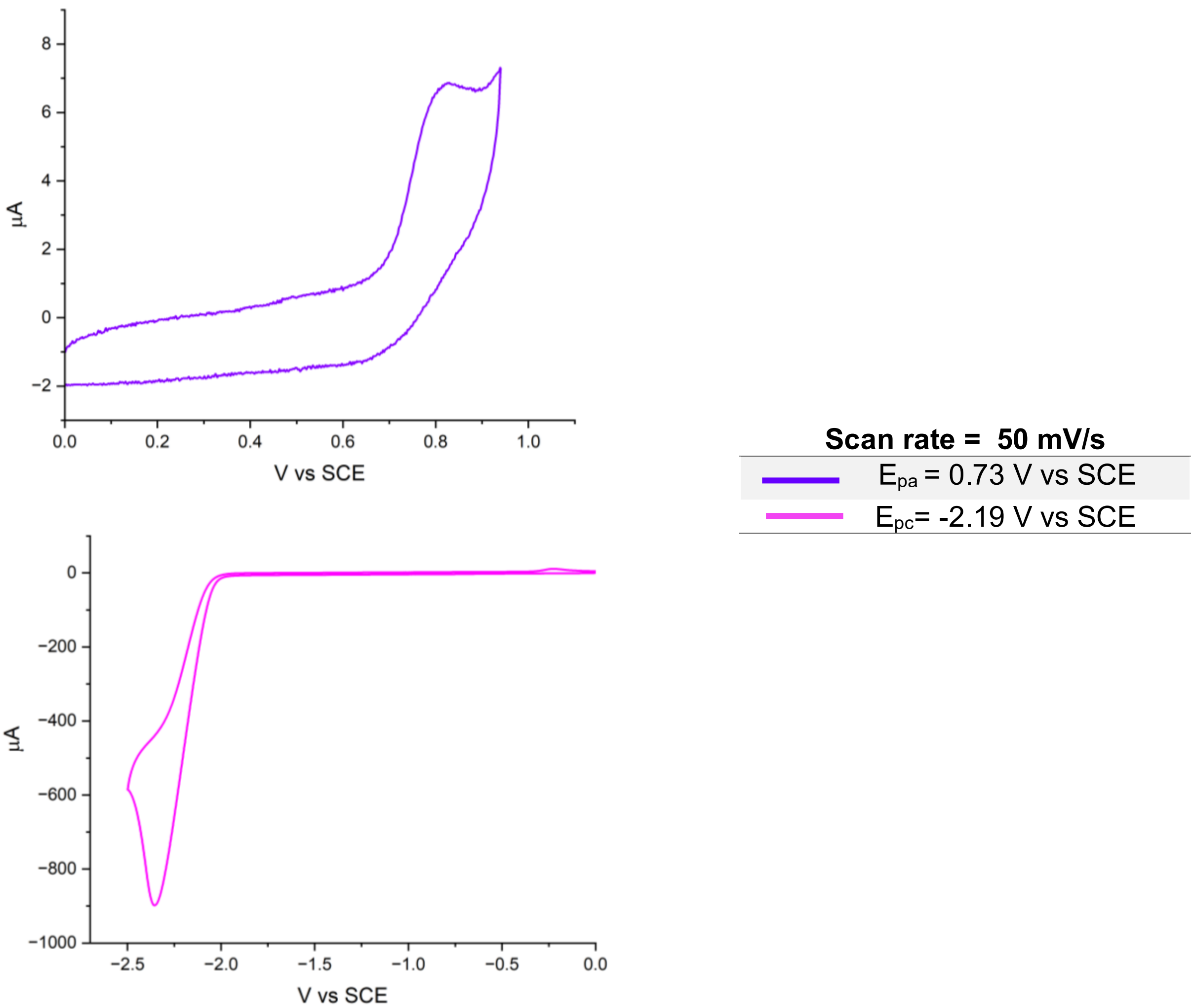

Figure S40*: Cyclic voltammograms of* ***8*** *(1mM) in a [Bu$_4$N][PF$_6$] (0.1 M) solution in MeCN. The plot follows the IUPAC convention. Glassy carbon electrode; Ag/AgCl (3 M NaCl) reference electrode; Pt counter electrode. For oxidation: Scan rate= 50 mV/s; initial potential= 0.0 V; switching potential= 0.94 V; final potential; 0.0 V. For the reduction: Scan rate= 50 mV/s. initial potential= 0.0 V; switching potential= -2.5 V; final potential; 0.0 V. Reversibility and irreversibility were determined using different scan rates.*

.

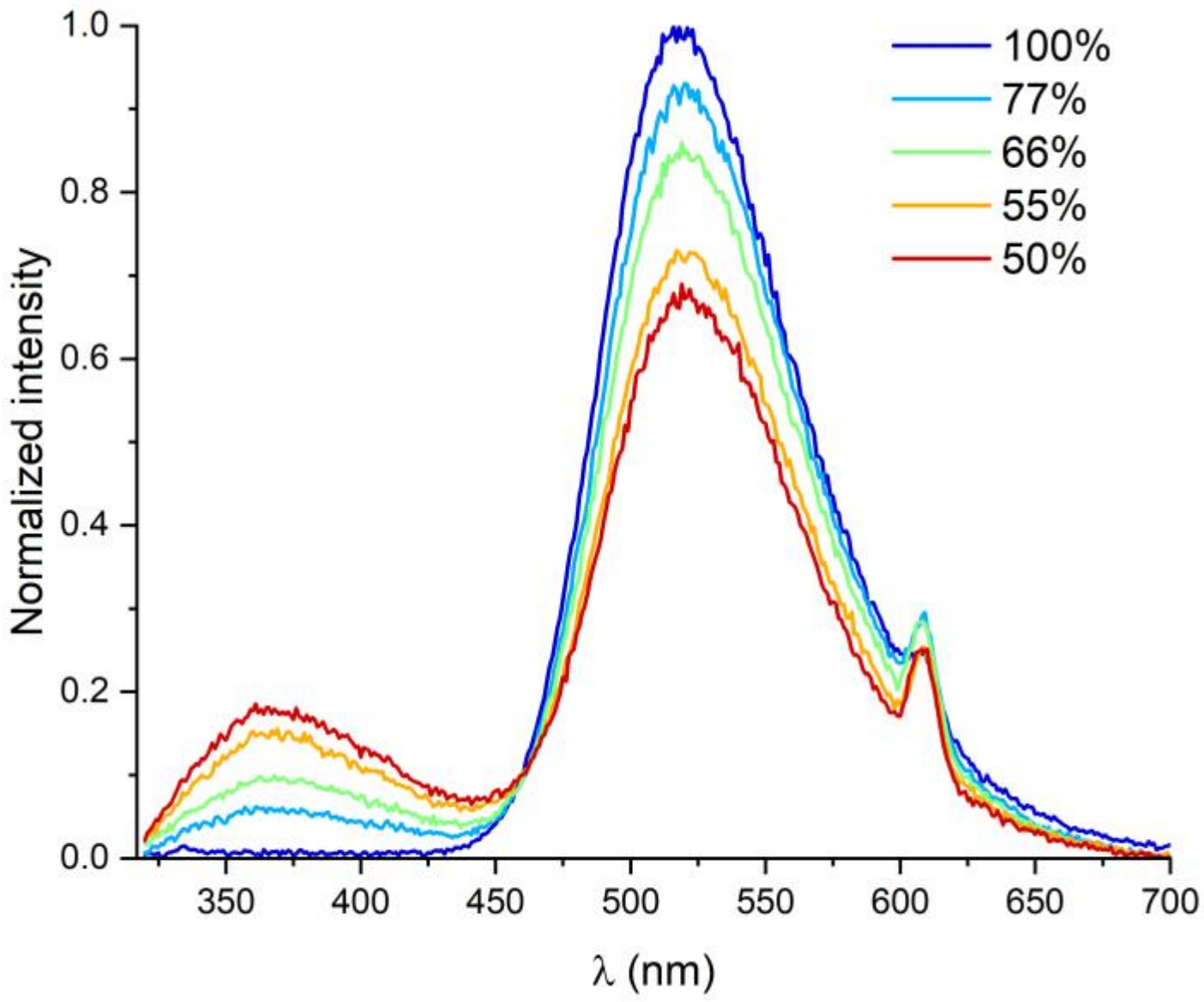

Figure S41: *Fluorescence measurements of molecule **8** at 1·10^{-5} M in different volume ratios of methanol/glycerol as a function of viscosity at room temperature. Slit excitation: 5. Slit emission: 5. $\lambda_{excitation}$ = 305 nm. Second harmonic is visualized.*

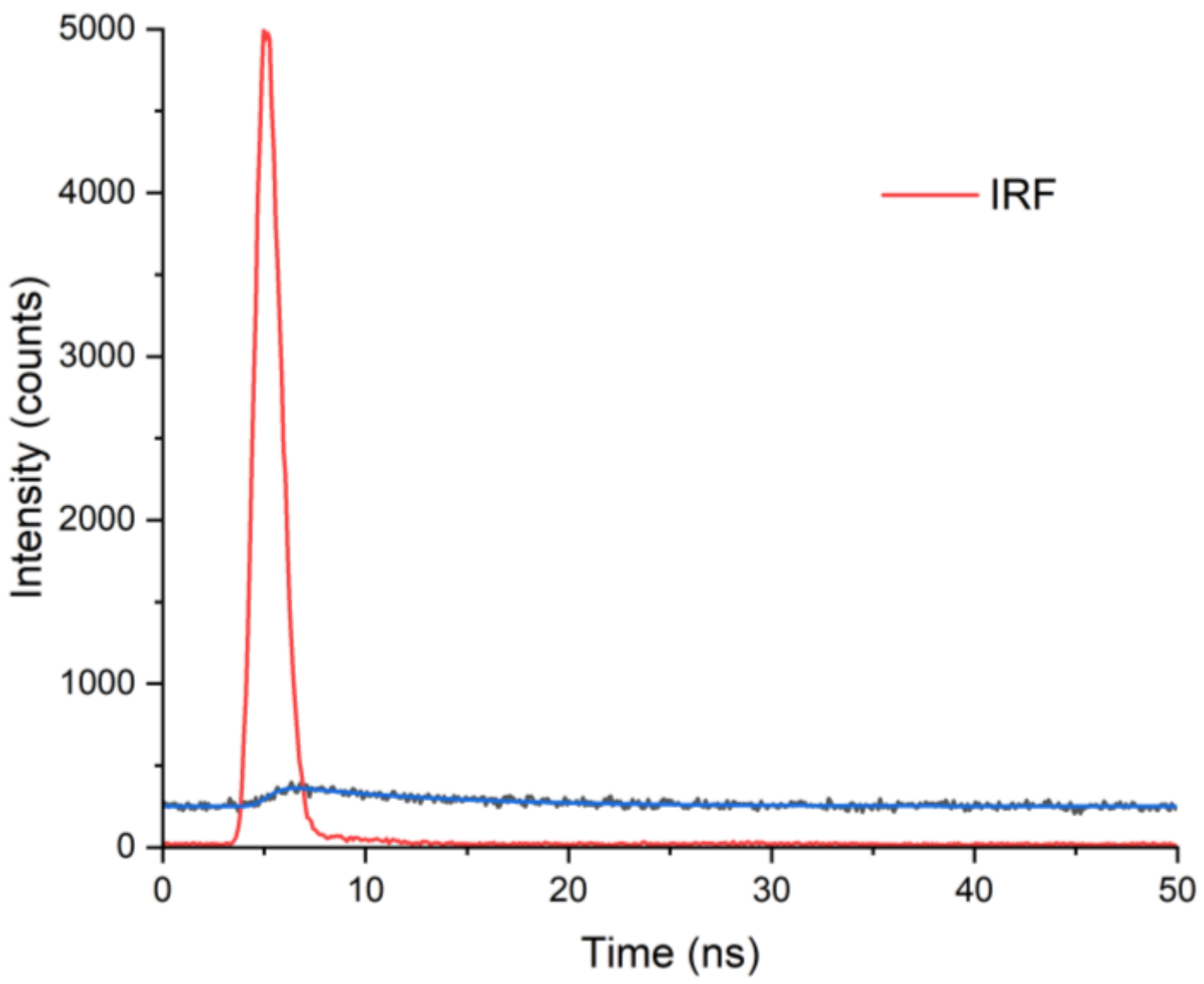

Figure S42: *Time-resolved emission decay at room temperature (excitation at 286.2 nm) of **8** at 1·10^{-5} M in MeCN solution measured by TC-SPC (τ = 7.8 ns), from deconvolution and single-exponential fitting, X2 = 1.09).*

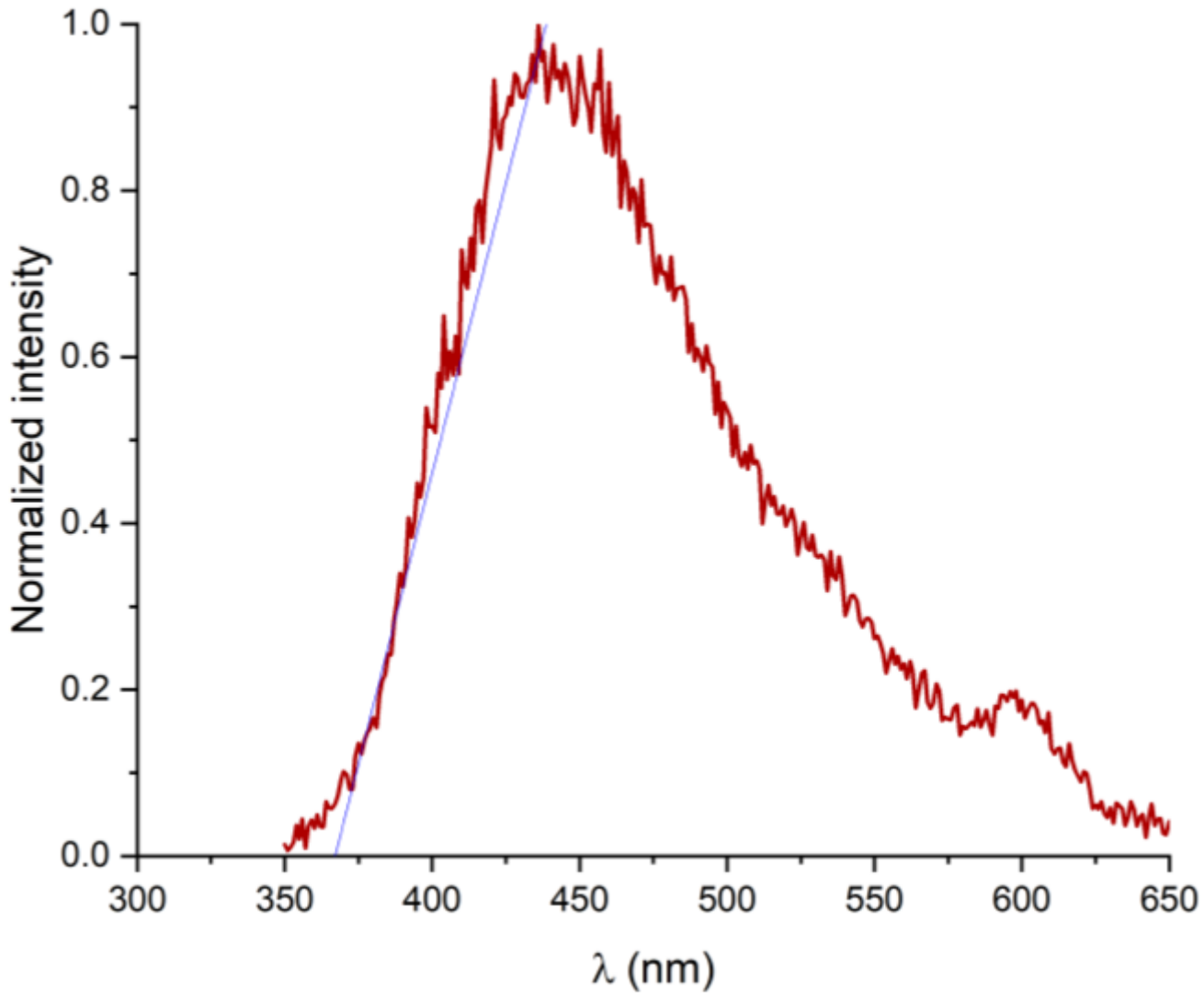

Figure S43: *Phosphorescence spectrum at 77 K of **8** at 1·10^{-5} M in 2-MeTHF. Excitation at 302 nm, slits=10.*

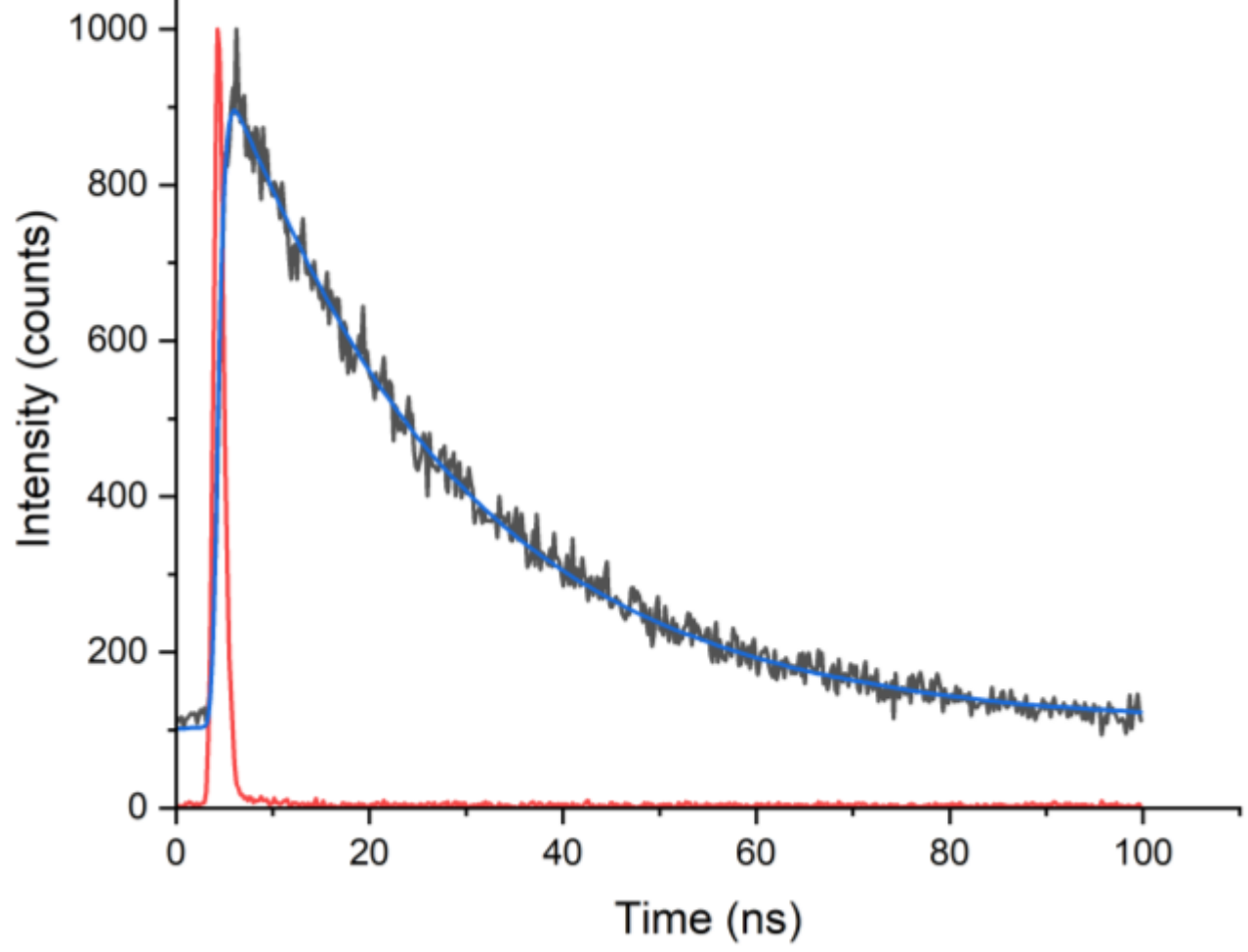

Figure S44: *Time-resolved emission decay at 77K of **8** at 1·10^{-5} M in 2-MeTHF solution measured by TC-SPC (τ = 23.6 ns), from deconvolution and single-exponential fitting, X2 = 1.1). Excitation: 286.2 nm. Emission 550 nm.*

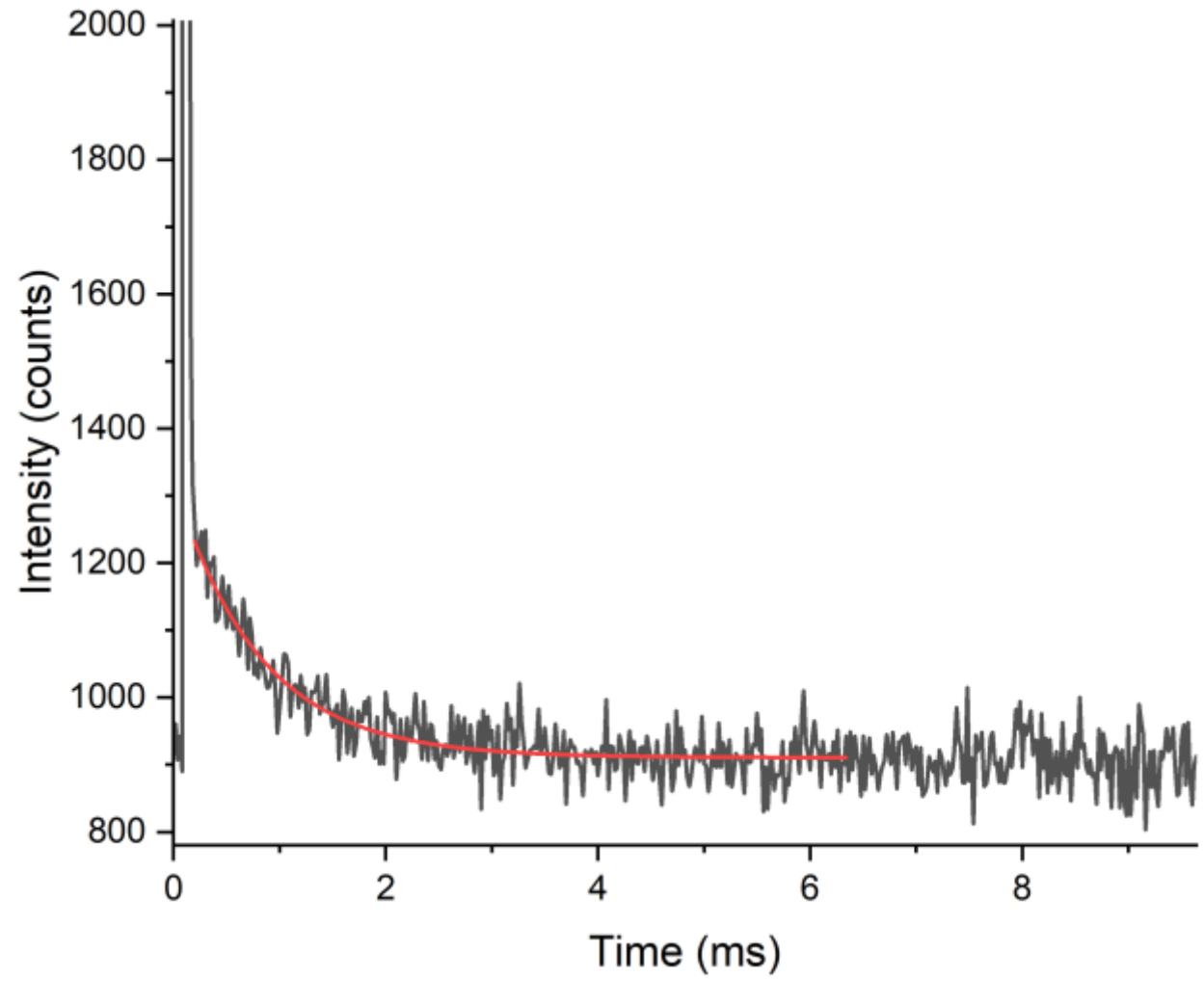

Figure S45: Triplet-state lifetime measurement of **8** *at 1·10^{-5} M* in a frozen MeTHF glass at 77 K, giving τ= 805 μs. *X2 = 1.17. Excitation: 302 nm. Emission :440 nm.*

Glycerol analysis

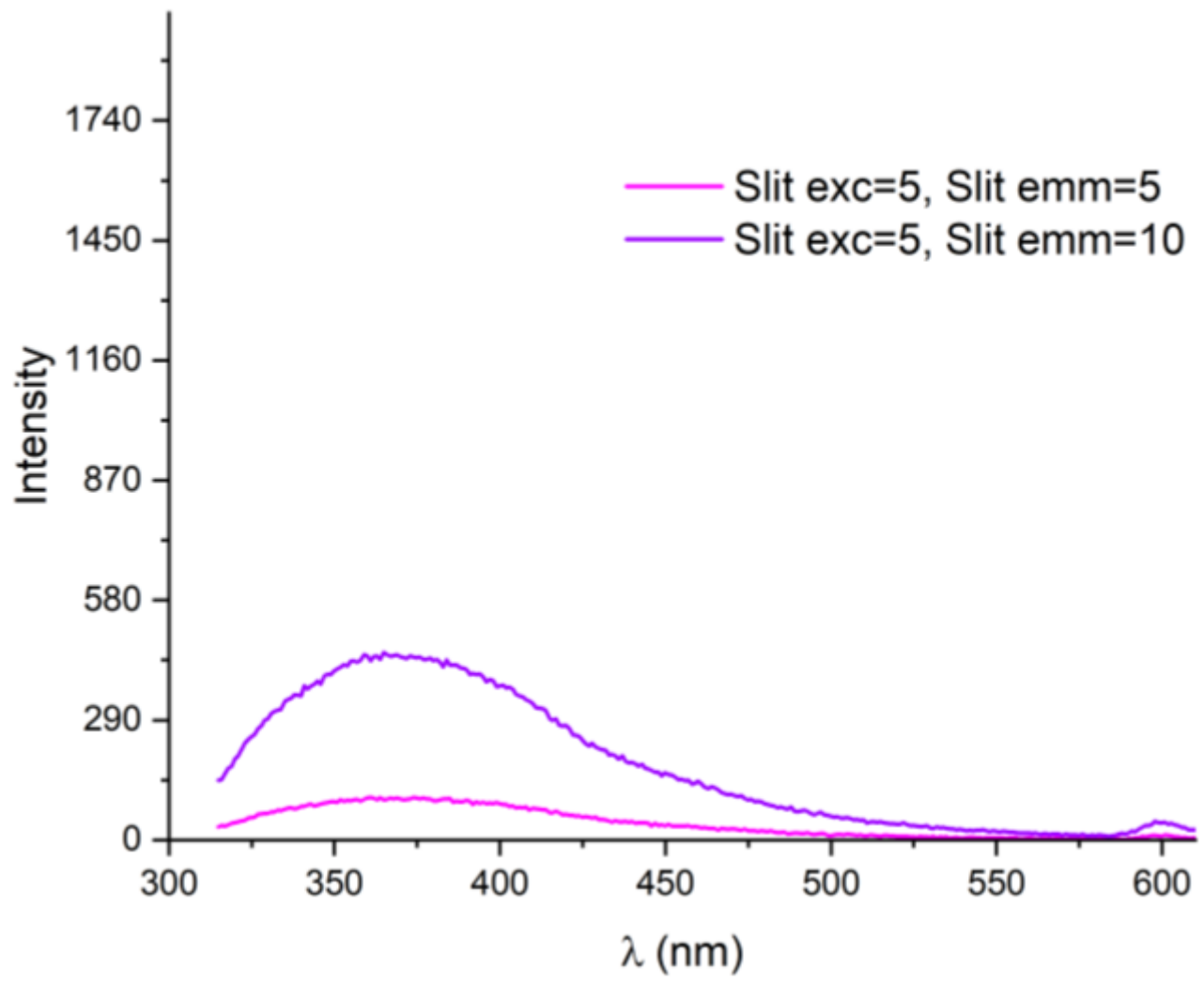

Figure S46*: Fluorescence measurements of the glycerol used in viscosity studies, modifying the slit according to the parameters used for molecules* ***4-8****.*

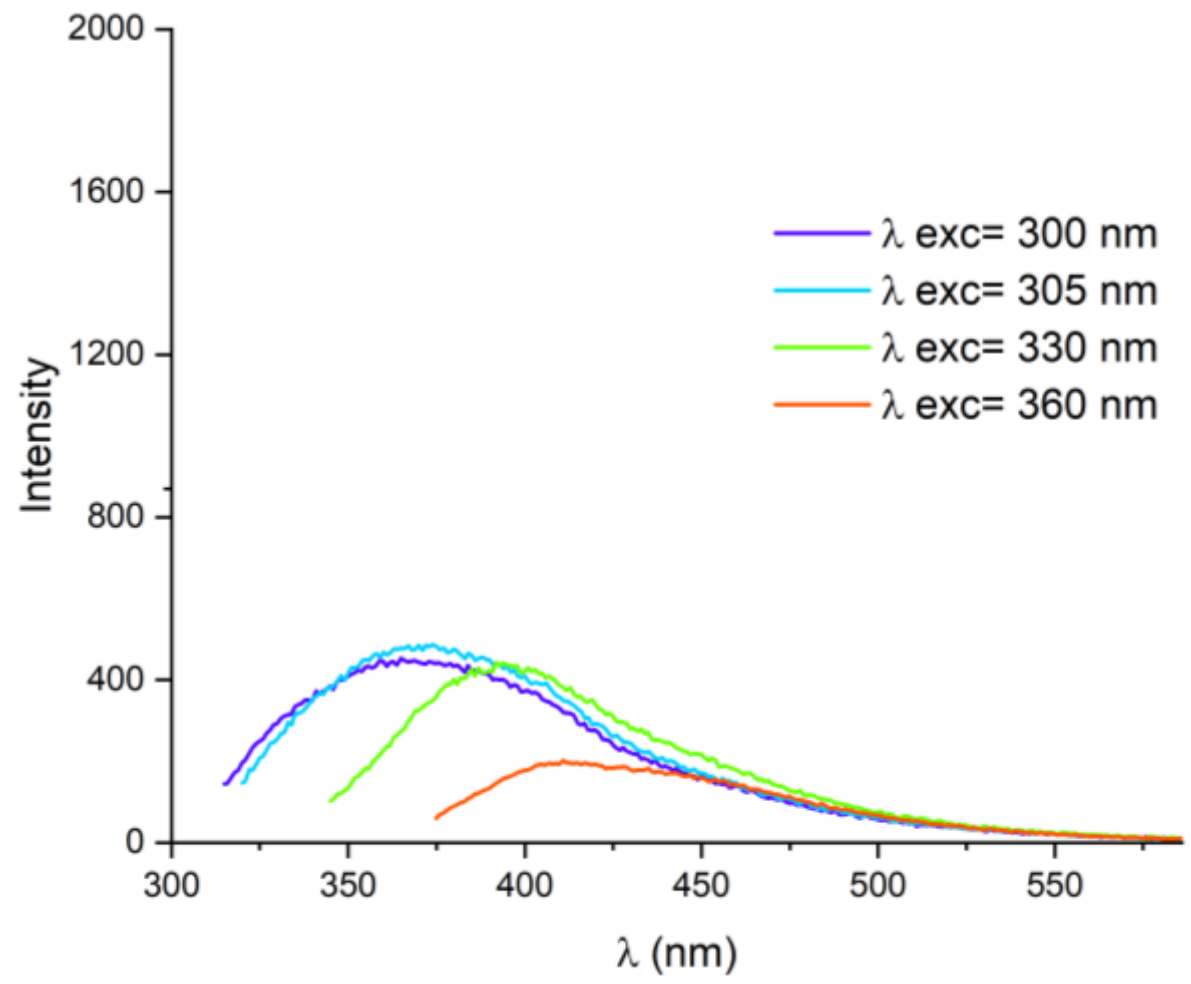

Figure S47*: Fluorescence measurements of the glycerol used in viscosity studies, modifying the* λ_{exc} *value.*

F. X-ray characterization.

The crystal was obtained by slow evaporation using dichloromethane as the solvent. A translucent colourless, Fragment (Prism)-shaped crystal was mounted on the goniometer. Data for compound **16** were collected from a single crystal at 298.00 K on a Bruker D8 KAPPA diffractometer with a microfocus sealed tube using a multilayer mirror as monochromator and a PhotonIII_C14 CPAD detector. The diffractometer used CuK_α radiation (λ = 1.54178 Å). All data were integrated with SAINT V8.42, yielding 48488 reflections of which 5410 where independent (average redundancy 8.96) and 76.9% were greater than 2σ(F^2).[6] A Multi-Scan absorption correction using SADABS 2016/2 was applied.[7] The structure was solved by dual methods with SHELXT 2018/2 and refined by full-matrix least-squares methods against F^2 using SHELXL 2018/3.[8,9] All non-hydrogen atoms were refined with anisotropic displacement parameters. All C-bound hydrogen atoms were refined isotropic on calculated positions using a riding model with their U_{iso} values constrained to 1.5 times the U_{eq} of their pivot atoms for terminal sp^3 carbon atoms and 1.2 times for all other carbon atoms. Crystallographic data for the structures reported in this paper have been deposited with the Cambridge Crystallographic Data Centre.[10] CCDC 2580408 contain the supplementary crystallographic data for this paper. These data can be obtained free of charge from The Cambridge Crystallographic Data Centre via www.ccdc.cam.ac.uk/structures.

CCDC number	2580408
Empirical formula	$C_{30}H_{19}N$
Formula weight	393.48
Temperature [K]	298.00
Crystal system	monoclinic
Space group (number)	$P2_1/c$ (14)
a [Å]	14.4471(3)
b [Å]	10.1946(2)
c [Å]	18.4517(4)
α [°]	90
β [°]	112.8080(10)
γ [°]	90
Volume [Å^3]	2505.12(9)
Z	4.0
ρ_{calc} [gcm^{-3}]	1.290
μ [mm^{-1}]	0.575
F(000)	1024
Crystal size [mm^3]	0.04×0.055×0.09
Crystal colour	translucent colourless
Crystal shape	Fragment (Prism)
Radiation	CuK_α (λ=1.54178 Å)
2θ range [°]	6.64 to 161.31 (0.78 Å)
Index ranges	$-18 \le h \le 18$<br>$-12 \le k \le 11$<br>$-23 \le l \le 23$
Reflections collected	48488
Independent reflections	5410<br>R_{int} = 0.0744<br>R_{sigma} = 0.0553
Completeness to θ = 67.679°	99.8
Data / Restraints / Parameters	5410 / 0 / 343
Goodness-of-fit on F^2	1.029
Final *R* indexes [$I \ge 2\sigma(I)$]	R_1 = 0.0476<br>wR_2 = 0.1170
Final *R* indexes [all data]	R_1 = 0.0656<br>wR_2 = 0.1299
Largest peak/hole [eÅ^{-3}]	0.41/−0.47

Table S2*. Crystal data and structure refinement for compound* ***16****.*

The crystal was obtained by slow evaporation using dichloromethane as the solvent. A translucent colourless, Fragment (Prism)-shaped crystal was mounted on the goniometer. Data for compound **5** were collected from a single crystal in 2.08 hours at 298.00 K on a Bruker D8 KAPPA diffractometer with a microfocus sealed tube using a multilayer mirror as monochromator and a PhotonIII_C14 CPAD detector. The diffractometer used CuK_α radiation (λ = 1.54178 Å). All data were integrated with SAINT V8.42, yielding 38850 reflections of which 5183 where independent (average redundancy 7.50) and 71.9% were greater than $2\sigma(F^2)$.[6] A Multi-Scan absorption correction using SADABS 2016/2 was applied.[7] The structure was solved by dual methods with SHELXT 2018/2 and refined by full-matrix least-squares methods against F^2 using SHELXL 2018/3.[8,9] All non-hydrogen atoms were refined with anisotropic displacement parameters. All C-bound hydrogen atoms were refined isotropic on calculated positions using a riding model with their U_{iso} values constrained to 1.5 times the U_{eq} of their pivot atoms for terminal sp^3 carbon atoms and 1.2 times for all other carbon atoms. Crystallographic data for the structures reported in this paper have been deposited with the Cambridge Crystallographic Data Centre.[10] CCDC 2580327 contain the supplementary crystallographic data for this paper. These data can be obtained free of charge from The Cambridge Crystallographic Data Centre via www.ccdc.cam.ac.uk/structures. This report and the CIF file were generated using FinalCif.[6]

CCDC number	2580327
Empirical formula	$C_{21}H_{12}NO_2$
Formula weight	310.33
Temperature [K]	298.00
Crystal system	monoclinic
Space group (number)	$P2_1/c$ (14)
a [Å]	21.9694(4)
b [Å]	9.8737(2)
c [Å]	11.4027(2)
α [°]	90
β [°]	104.5480(10)
γ [°]	90
Volume [Å^3]	2394.16(8)
Z	6.0
ρ_{calc} [gcm^{-3}]	1.264
μ [mm^{-1}]	0.614
F(000)	960
Crystal size [mm^3]	0.035×0.055×0.085
Crystal colour	translucent colourless
Crystal shape	Fragment (Prism)
Radiation	CuK_α (λ=1.54178 Å)
2θ range [°]	8.32 to 160.94 (0.78 Å)
Index ranges	$-26 \le h \le 27$<br>$-12 \le k \le 12$<br>$-14 \le l \le 12$
Reflections collected	38850
Independent reflections	5183<br>R_{int} = 0.0889<br>R_{sigma} = 0.0487
Completeness to θ = 67.679°	99.9
Data / Restraints / Parameters	5183 / 0 / 319
Goodness-of-fit on F^2	0.994
Final *R* indexes [$I \ge 2\sigma(I)$]	R_1 = 0.0461<br>wR_2 = 0.1290
Final *R* indexes [all data]	R_1 = 0.0677<br>wR_2 = 0.1457
Largest peak/hole [eÅ^{-3}]	0.20/−0.18
Extinction coefficient	0.0031(4)

***Table S3**. Crystal data and structure refinement for compound **5**.*

The crystal was obtained by slow evaporation using dichloromethane as the solvent. A translucent orange-yellow, prism-shaped crystal was mounted on the goniometer. Data for compound **6** were collected from a single crystal in 2.38 hours at 298.00 K on a Bruker D8 KAPPA diffractometer with a microfocus sealed tube using a multilayer mirror as monochromator and a PhotonIII_C14 CPAD detector. The diffractometer used CuK_α radiation (λ = 1.54178 Å). All data were integrated with SAINT V8.42, yielding 24690 reflections of which 5304 where independent (average redundancy 4.65) and 76.7% were greater than 2σ(F^2).[6] A Multi-Scan absorption correction using SADABS 2016/2 was applied.[7] The structure was solved by dual methods with SHELXT 2018/2 and refined by full-matrix least-squares methods against F^2 using SHELXL 2018/3.[8,9] All non-hydrogen atoms were refined with anisotropic displacement parameters. All C-bound hydrogen atoms were refined isotropic on calculated positions using a riding model with their U_{iso} values constrained to 1.5 times the U_{eq} of their pivot atoms for terminal sp^3 carbon atoms and 1.2 times for all other carbon atoms. Crystallographic data for the structures reported in this paper have been deposited with the Cambridge Crystallographic Data Centre.[10] CCDC 2580329 contain the supplementary crystallographic data for this paper. These data can be obtained free of charge from The Cambridge Crystallographic Data Centre via www.ccdc.cam.ac.uk/structures.

CCDC number	2580329
Empirical formula	$C_{38}H_{25}N$
Formula weight	495.61
Temperature [K]	298.00
Crystal system	monoclinic
Space group (number)	$P2_1/c$ (14)
a [Å]	16.8053(3)
b [Å]	7.89090(10)
c [Å]	20.5585(4)
α [°]	90
β [°]	113.4020(10)
γ [°]	90
Volume [Å^3]	2501.98(8)
Z	4.0
ρ_{calc} [gcm^{-3}]	1.316
μ [mm^{-1}]	0.576
F(000)	1040
Crystal size [mm^3]	0.045×0.085×0.14
Crystal colour	translucent orange-yellow
Crystal shape	prism
Radiation	CuK_α (λ=1.54178 Å)
2θ range [°]	8.83 to 160.74 (0.78 Å)
Index ranges	−21 ≤ h ≤ 21<br>−9 ≤ k ≤ 9<br>−26 ≤ l ≤ 26
Reflections collected	24690
Independent reflections	5304<br>R_{int} = 0.0687<br>R_{sigma} = 0.0498
Completeness to θ = 67.679°	99.8
Data / Restraints / Parameters	5304 / 0 / 352
Goodness-of-fit on F^2	1.037
Final *R* indexes [I≥2σ(I)]	R_1 = 0.0466<br>wR_2 = 0.1246
Final *R* indexes [all data]	R_1 = 0.0622<br>wR_2 = 0.1356
Largest peak/hole [eÅ^{-3}]	0.47/−0.41

***Table S4**. Crystal data and structure refinement for compound **6**.*

A translucent green, Fragment (Prism)-shaped crystal was mounted on the goniometer. Data for compound **8** were collected from a single crystal at 298.00 K on a Bruker D8 KAPPA diffractometer with a microfocus sealed tube using a multilayer mirror as monochromator and a PhotonIII_C14 CPAD detector. The diffractometer used CuK_α radiation (λ = 1.54178 Å). All data were integrated with SAINT V8.42, yielding 30245 reflections of which 5202 where independent (average redundancy 5.81) and 84.9% were greater than $2\sigma(F^2)$.[6] A Multi-Scan absorption correction using SADABS 2016/2 was applied.[7] The structure was solved by dual methods with SHELXT 2018/2 and refined by full-matrix least-squares methods against F^2 using SHELXL 2018/3.[8,9] All non-hydrogen atoms were refined with anisotropic displacement parameters. All C-bound hydrogen atoms were refined isotropic on calculated positions using a riding model with their U_{iso} values constrained to 1.5 times the U_{eq} of their pivot atoms for terminal sp^3 carbon atoms and 1.2 times for all other carbon atoms. Crystallographic data for the structures reported in this paper have been deposited with the Cambridge Crystallographic Data Centre.[10] CCDC 2580330 contain the supplementary crystallographic data for this paper. These data can be obtained free of charge from The Cambridge Crystallographic Data Centre via www.ccdc.cam.ac.uk/structures.

CCDC number	2580330
Empirical formula	$C_{32}H_{19}F_6N$
Formula weight	531.49
Temperature [K]	298.00
Crystal system	monoclinic
Space group (number)	$P2_1/c$ (14)
a [Å]	15.842(2)
b [Å]	7.8010(12)
c [Å]	20.747(3)
α [°]	90
β [°]	106.459(6)
γ [°]	90
Volume [Å^3]	2459.0(6)
Z	4.0
ρ_{calc} [gcm^{-3}]	1.436
μ [mm^{-1}]	0.984
F(000)	1088
Crystal size [mm^3]	0.03×0.1×0.17
Crystal colour	translucent green
Crystal shape	Fragment (Prism)
Radiation	CuK_α (λ=1.54178 Å)
2θ range [°]	5.82 to 160.84 (0.78 Å)
Index ranges	$-20 \le h \le 20$<br>$-8 \le k \le 9$<br>$-25 \le l \le 26$
Reflections collected	30245
Independent reflections	5202<br>R_{int} = 0.0506<br>R_{sigma} = 0.0368
Completeness to θ = 67.679°	99.9
Data / Restraints / Parameters	5202 / 27 / 356
Goodness-of-fit on F^2	1.071
Final *R* indexes [$I \ge 2\sigma(I)$]	R_1 = 0.0821<br>wR_2 = 0.2389
Final *R* indexes [all data]	R_1 = 0.0911<br>wR_2 = 0.2503
Largest peak/hole [eÅ^{-3}]	0.90/−0.88
Extinction coefficient	0.0025(6)

***Table S5**. Crystal data and structure refinement for compound **8**.*

Top view

16 **5** **6**

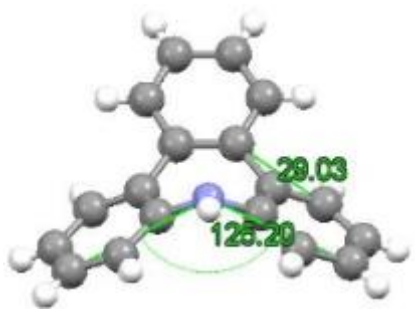

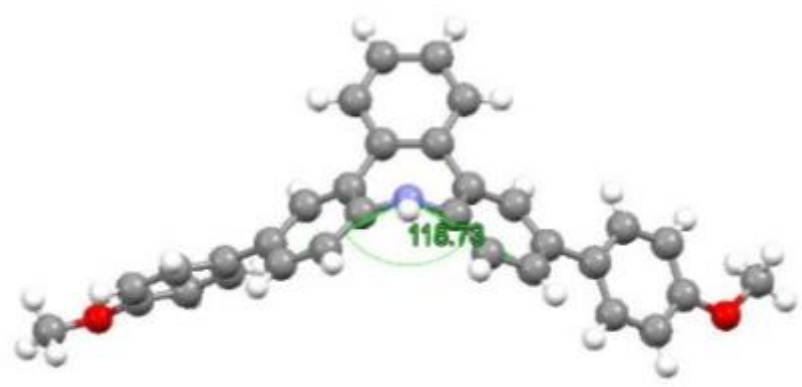

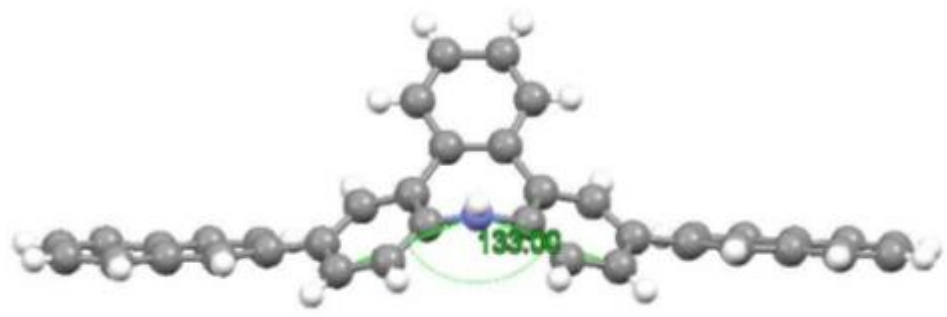

Side view

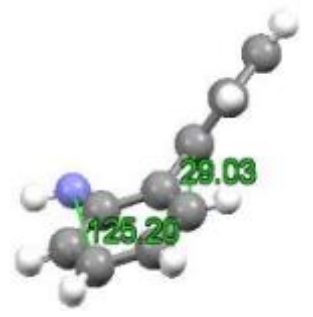

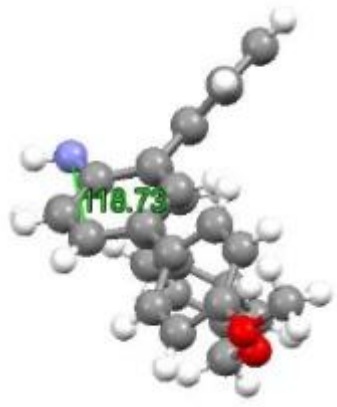

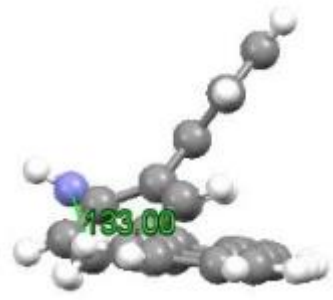

Top view

8

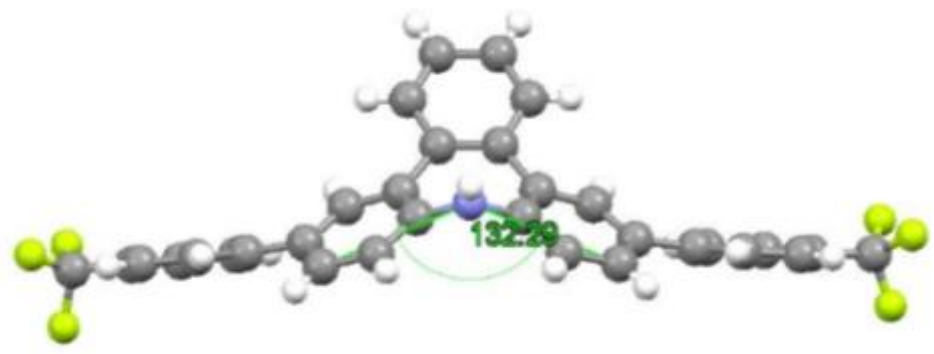

Side view

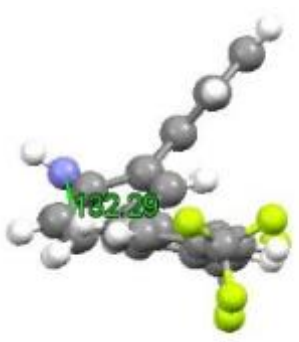

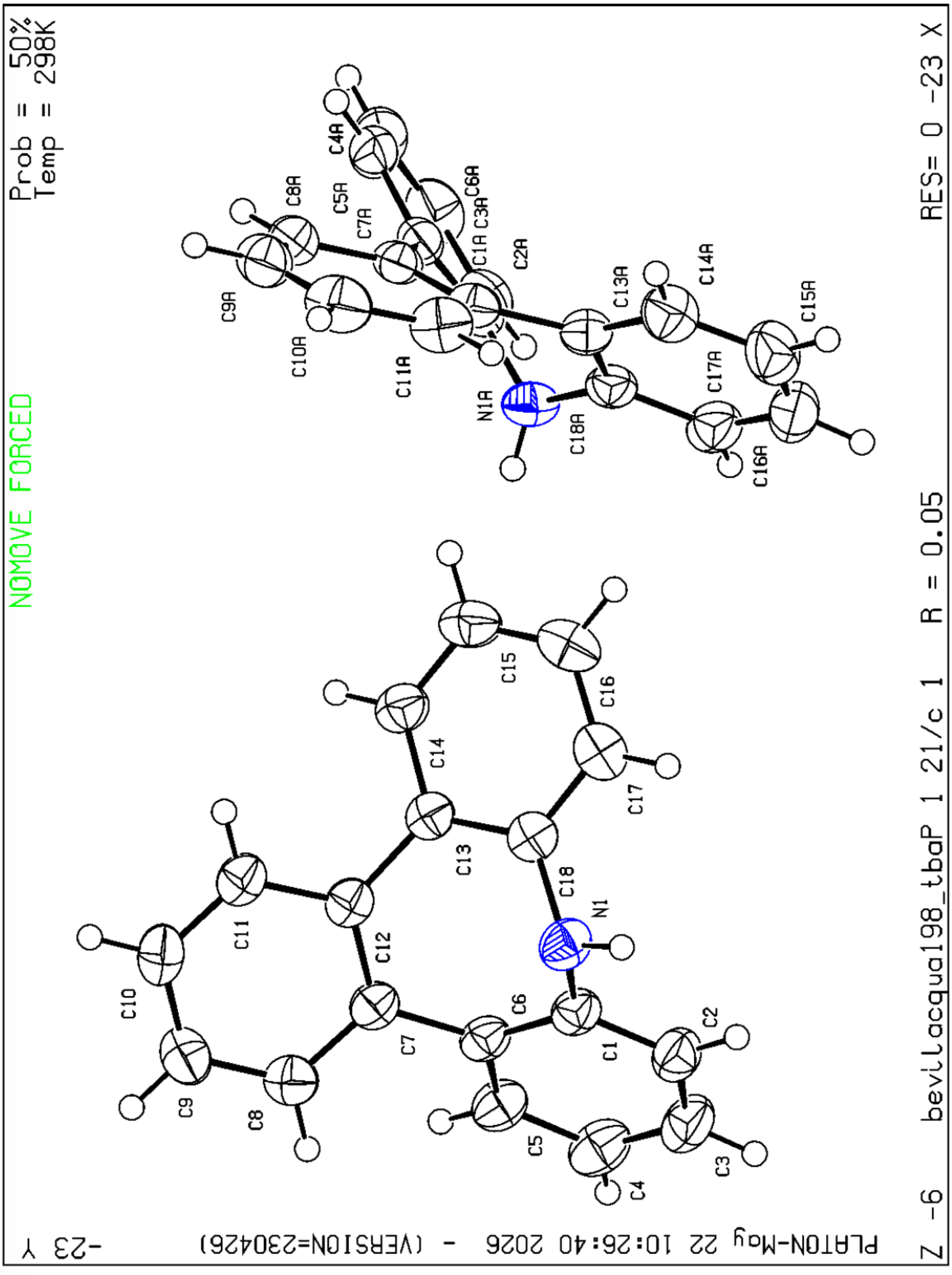

***Figure S48**:* Thermal ellipsoid plot for **16** crystal structure (50% probability).

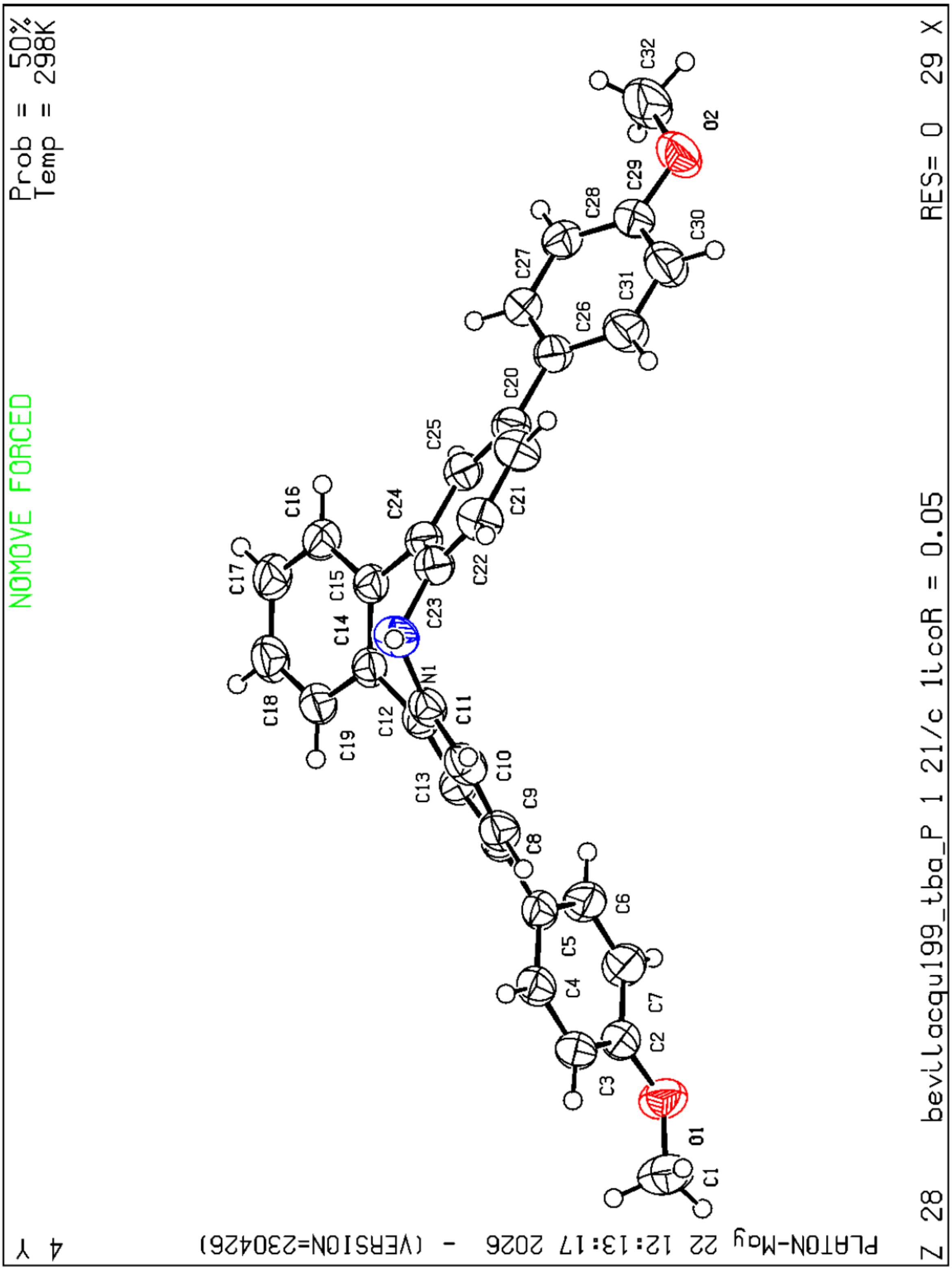

***Figure S49**:* Thermal ellipsoid plot for **5** crystal structure (50% probability).

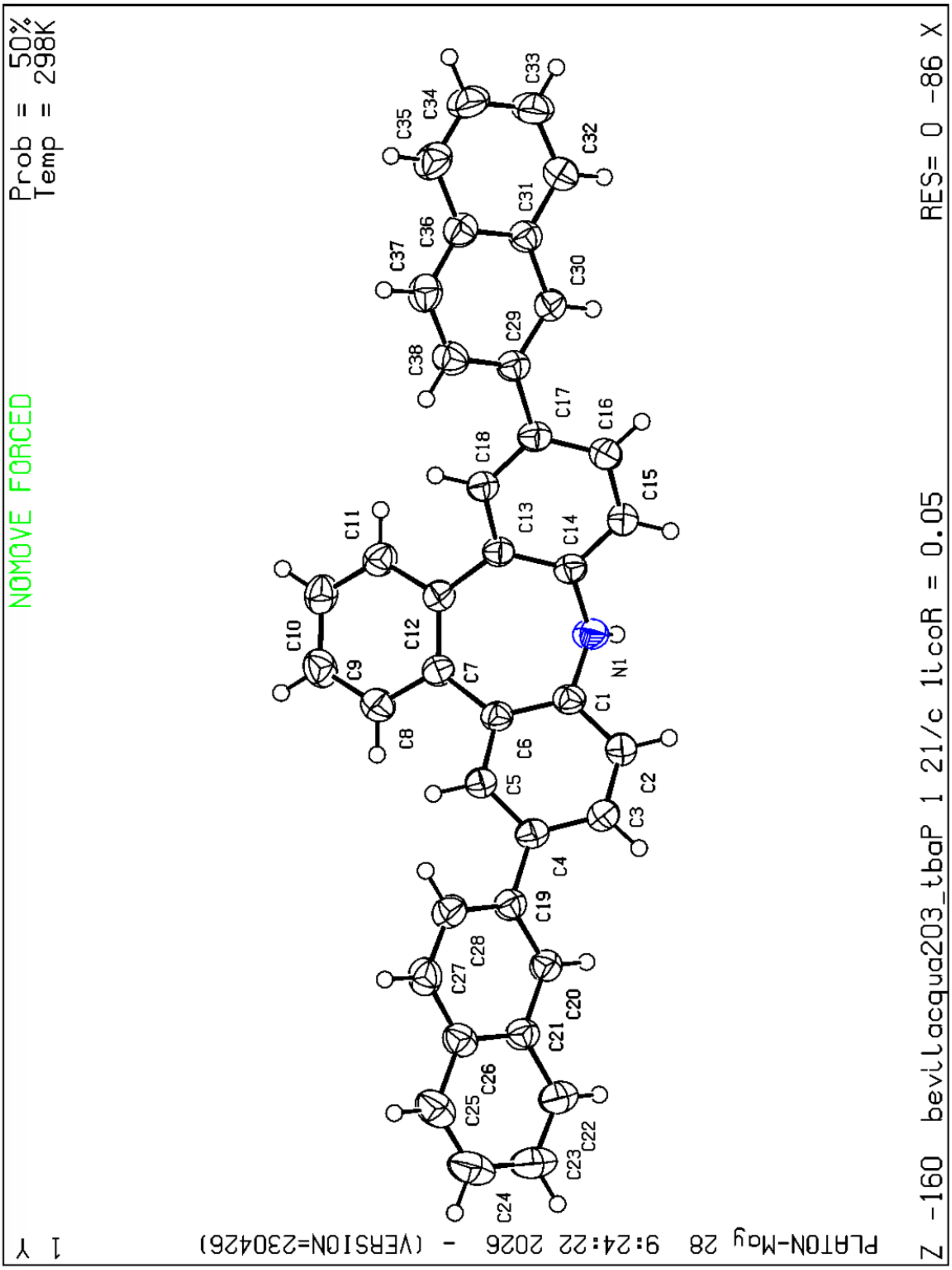

***Figure S50**:* Thermal ellipsoid plot for **6** crystal structure (50% probability).

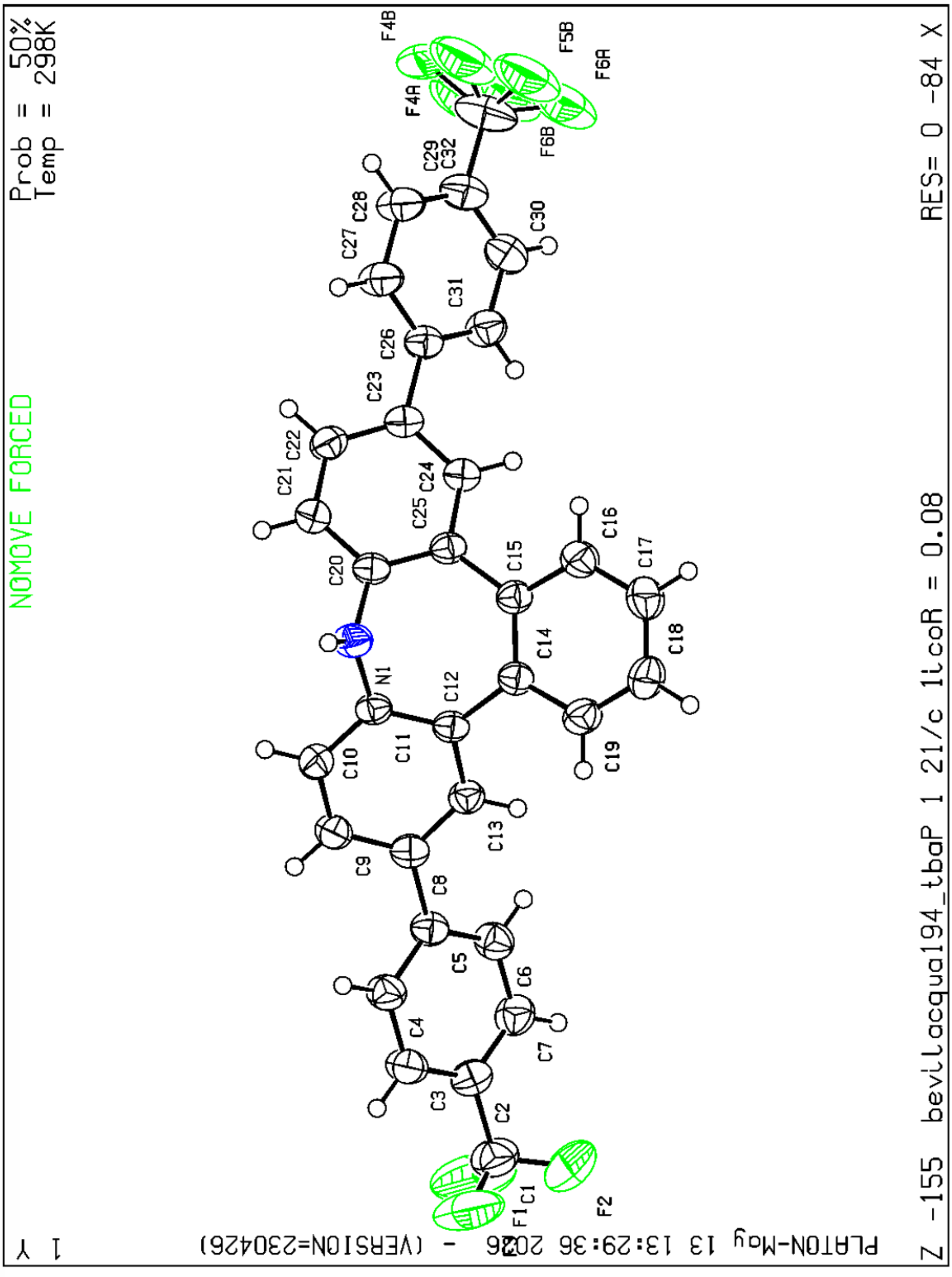

***Figure S51**:* Thermal ellipsoid plot for **8** crystal structure (50% probability).

G. Evaluation of photocatalytic activity

Oxidative quenching cycle

General Procedure B

Dehalogenation of 4-(methoxycarbonyl)-N,N,N-trimethylbenzenaminium trifluoromethanesulfotate.

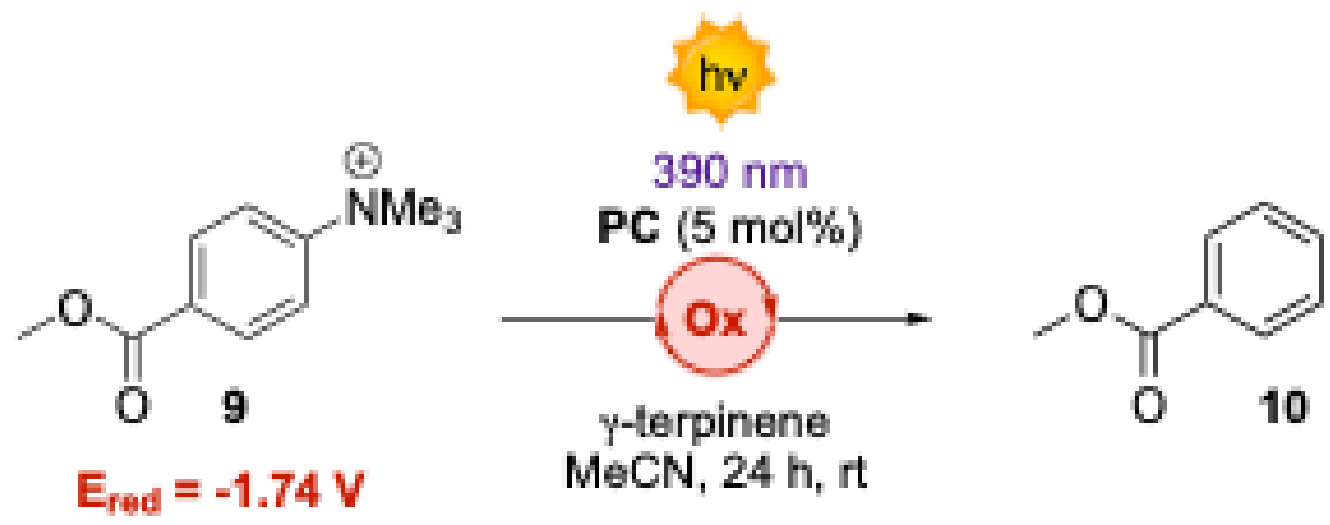

The procedure was slightly modified from the one reported in the literature.[11] A 4 mL vial equipped with a septum was charged with a magnetic stir bar, ammonium salt **9** (34.3 mg 0.1 mmol, 1 eq.) and PC (5 mol%) and degassed with Ar. Then degassed MeCN (1 mL, 0.1 M) was added, followed by γ-terpinene (80 μl, 0.5 mmol, 5 equiv). The reaction mixture was purged with Ar for 1 minute. The vial was irradiated with a 390 nm Kessil lamp set at 100% of its maximum output power for the indicated amount of time. Yield of methyl benzoate (**10**) was determined by 1H NMR using CH_2Br_2 as the internal standard. **^{1}H NMR (400 MHz, $CDCl_3$):** δ 8.04 (d, 2H, J=8.5 Hz), 7.54 (t, 1H, *J*=7.3 Hz), 7.42 (t, 2H, *J*=7.7 Hz), 3.90 (s, 3H), ppm. These data match those previously reported in the literature.[11]

PC	NMR Yield (%)
**4**	49
**5**	34
**6**	43
**7**	16
**9**	32

Table S6: *Results of the screening of different PCs in the reduction of ammonium salt **9** according to General procedure B. CH_2Br_2 was used as an internal standard for the NMR yield calculation.*

Reductive quenching cycle

General Procedure C

Giese addition using Cbz-Pro-OH and methyl maleate.

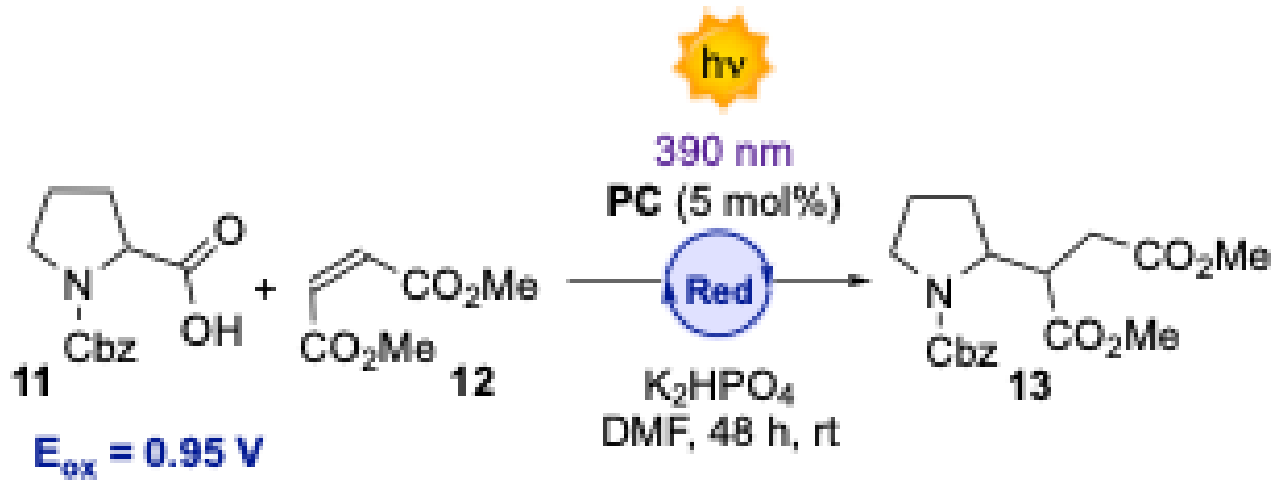

The procedure was followed according to the literature.[12a] To an oven dried vial a magnetic stir bar was charged with the selected photocatalyst (5 mol%), Cbz-Pro-OH **11** (50 mg, 0.2 mmol, 1.0 equiv), dimethyl maleate **12** (28.8 mg, 0.2 mmol, 1.0 equiv), K_2HPO_4 (41.4 mg, 0.24 mmol, 1.2 equiv), and DMF (0.5 ml, 0.4 M). The reaction mixture was degassed using the Freeze-Pump-Thaw technique, then irradiated using a 390 nm Kessil lamp set at 100% of its maximum output power for 48 h. The reaction mixture was diluted with saturated aqueous $NaHCO_3$ solution and extracted with Et_2O (3 × 5 mL). The combined organic extracts were washed with water and brine, dried over $MgSO_4$, and concentrated in vacuo. Purification of the crude product by flash chromatography (8:2 Hexane/AcOEt) on silica gel afforded the desired product **13.** **^{1}H NMR (400 MHz, $CDCl_3$)** δ 7.47 – 7.27 (m, 5H), 5.27 – 5.04 (m, 2H), 4.34 – 4.29 (m, 0.5H), 4.19 – 4.06 (m, 0.5H), 3.74 – 3.43 (m, 8H), 3.40 – 3.30 (m, 1H), 3.29 – 3.16 (m, 1H), 2.88 – 2.65 (m, 1H), 2.58 – 2.23 (m, 1H), 2.02 – 1.67 (m, 4H). The ^{1}H NMR spectrum is consistent with that in the literature.[13]

PC	Yield (%)
**4**	N.R.
**5**	N.R
**6**	Traces
**7**	63
**8**	70 (61^a)

Table S7: *Results of the screening of different PCs in the Giese reaction to obtain* ***13*** *according to General procedure C. Isolated yield in parentheses. CH_2Br_2 was used as an internal standard for the NMR yield calculation.*
^{a} 42.6 mg

Energy transfer

General Procedure D

Photoisomerization of *trans*-Stilbene

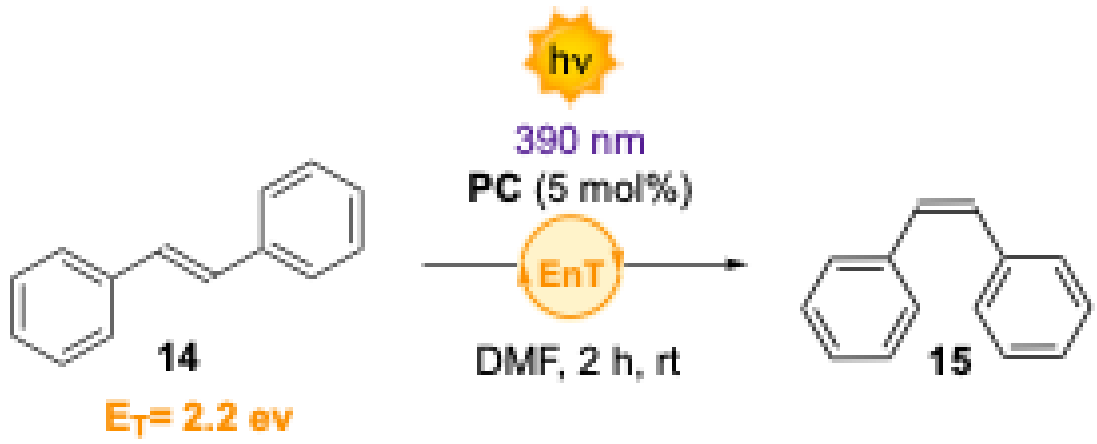

The procedure was followed according to the literature.[12b] To an oven-dried vial was added *trans*-Stilbene **14** (36 mg, 0.2 mmol, 1 eq) and photocatalyst (5 mol %). The vial was purged with N_2 for 5 minutes, and dry DMF (2 mL, 0.1 M) was added before further N_2 purging for 10 more minutes. The solution was stirred at room temperature while being irradiated by a Kessil lamp (390 nm, 100% intensity) for 2 h. After removing the solvent, the crude product was purified by flash column chromatography using hexane as the eluent to afford product **15**. **^{1}H NMR (400 MHz, $CDCl_3$)** 7.29-7.16 (m, 10H), 6.61 (s, 2H).The ^{1}H NMR spectrum is consistent with that in the literature.[12b]

PC	Yield (%)
**4**	81 (74^a)
**5**	77
**6**	78
**7**	85
**8**	85

Table S8: *Results of the screening of different PCs in the photoisomerization to obtain **15** according to General procedure D. Isolated yield in parentheses. CH_2Br_2 was used as an internal standard for the NMR yield calculation.*
^{a} 26.6 mg

Mechanistic studies

Photostationary analysis

Time (H)	% Z-Stilbene (**15**)	% E-Stilbene (**14**)
**0**	0	100
**2**	83	17
**4**	82	18
**6**	78	22
**8**	80	20
**14**	78	22
**18**	75	25
**24**	68	32

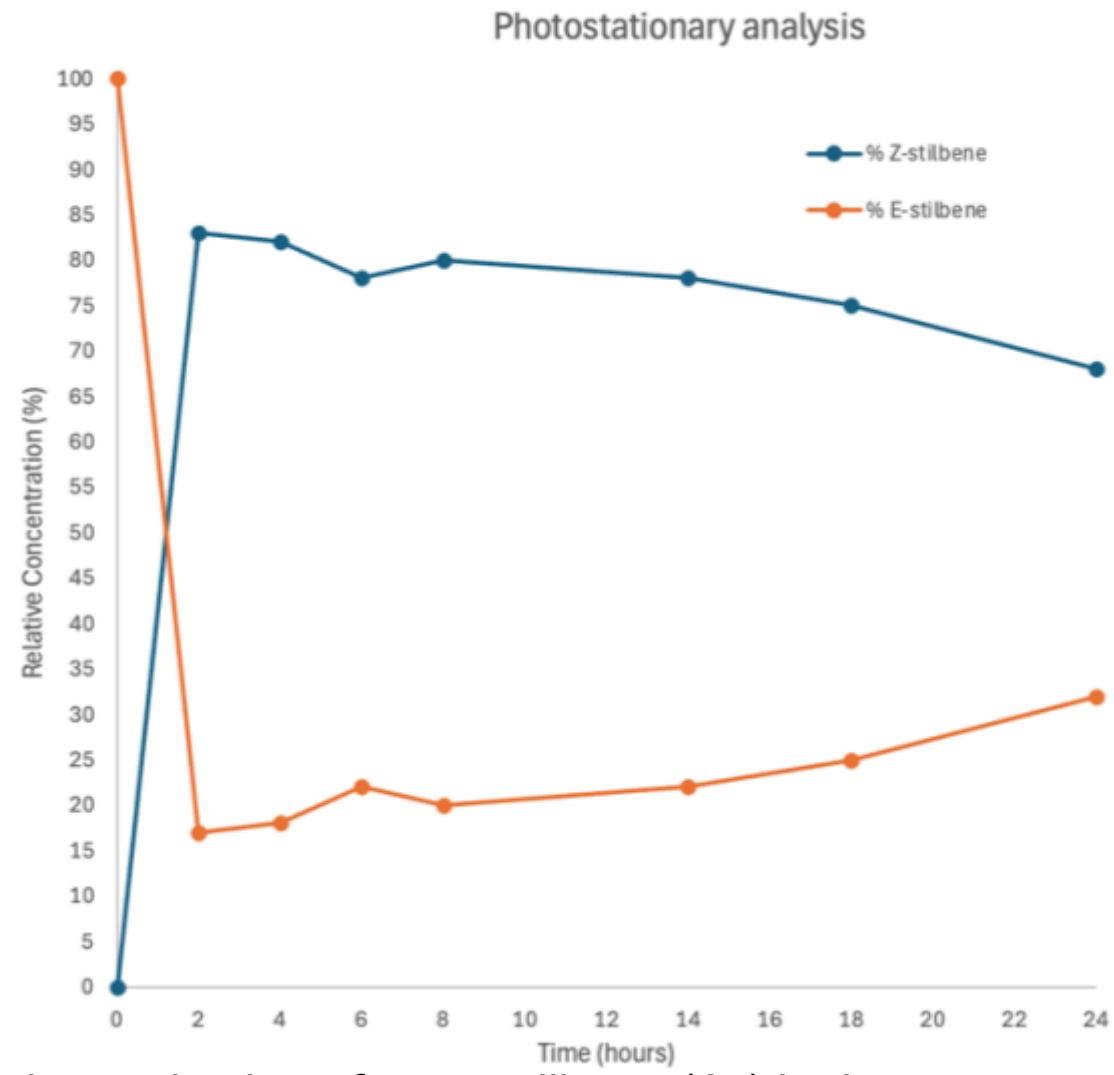

Figure S52. Photostationary analysis for the photoisomerization of trans-stilbene (**14**) in the presence of **7**.

Stern-Volmer analysis.

For the Stern-Volmer analysis, two stock solutions of **7** (1×10^{-4} M and $1\text{x}10^{-3}$ M) were prepared in 10 mL of DMF. A 0.1 M stock solution of the quenchers (trans-stilbene and dimethyl fumarate) was also prepared using 20 mL of DMF. The final concentration of the solutions utilized during the study is shown in the plot.

Before the measurements, the cuvette was degassed for 3 minutes with argon. We did not observe the fluorescence quenching of **7** in the presence of the substrates. This indirectly confirms that the isomerization of **14** does not occur via a SET process; instead, it involves the triplet. Dimethyl fumarate is not able to interact with **7** via SET nor by EnT.

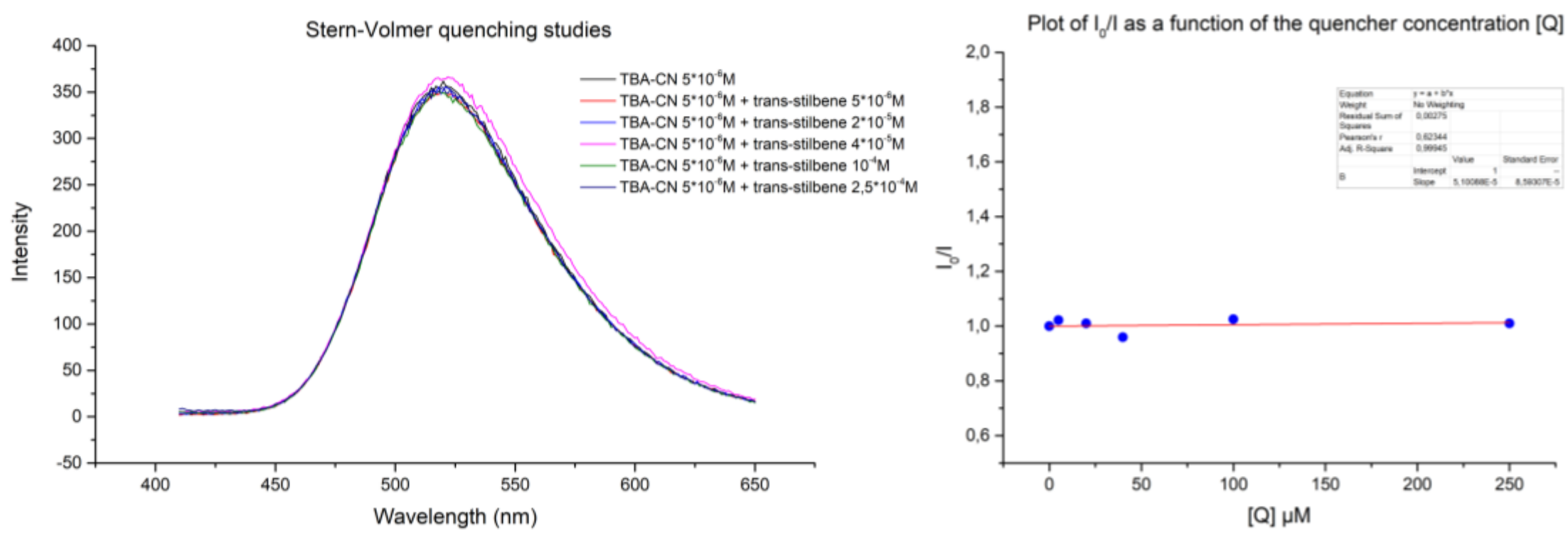

Figure S53. Stern-Volmer analysis of molecule **7** in the presence of **14.**

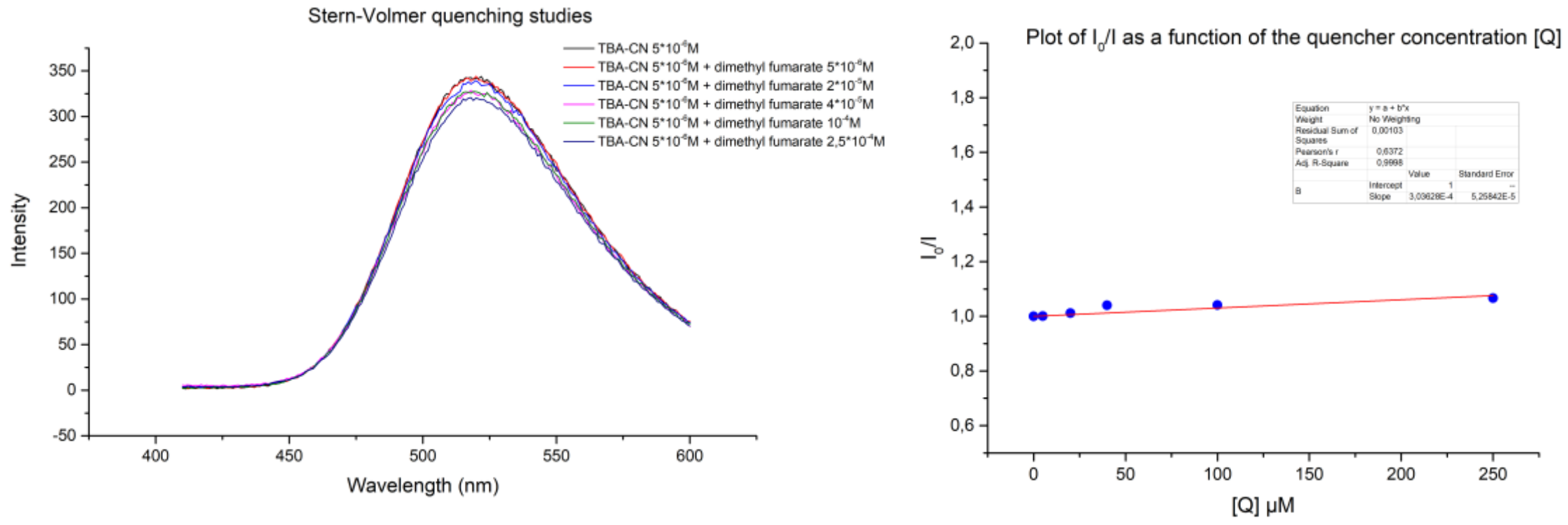

Figure S54. Stern-Volmer analysis of molecule **7** in the presence of dimethyl fumarate**.**

H. Theoretical studies

DFT calculations

Computational details
All the calculations were performed with the Gaussian 16 program package (Revision A.03).[14] Each geometry was optimized with default convergence thresholds (RMS values of 3x10-4 hartree/bohr and 1.2x10-3 bohr and maximum values of 4.5x10-4 hartree/bohr and 1.8x10-3 bohr on forces and displacements, respectively) at ωB97X-D/Def2-TZVP level of theory. Polarization effects were taken into account with the polarizable continuum model (PCM).

The energy gap between HOMO-LUMO was calculated using the ΔSCF method (Delta Self-Consistent Field).[15] This method estimates the HOMO and LUMO energies (and gap) by means of the energy of the potential of ionization (IP) and electron affinity (EA). These last values are calculated by the difference between the neutral form and the cation and anion species. All these energies are summarized in Table S13.

IP = energy (cation) - energy (neutral)
EA = energy (neutral) – energy (anion)
HOMO-LUMO gap = IP-EA

The excited state optimization for each molecule was performed using TDDFT at ωB97X-D/Def2-TZVP level of theory, requesting 10 roots per molecule and setting singlet states 1, 2, and 3 as optimization roots. Implicit solvation was considered by the conductor-like polarized continuum model (C-PCM) using acetonitrile as dielectric field. $E_{0,0}$ energies were calculated by Franck-Condon-Herzberg-Teller using the optimized ground state geometry and its corresponding frequencies as initial guess and the S_1 optimized excited state geometry and its corresponding frequencies as the final guess.[16] Further analysis of the charge-transfer character and Δr were calculated by transition density matrix analysis using TheoDORE[17] version 3.2.1. The major contributions and pair of orbitals involved for the first excited state, Δr, and excitation energies for the transition are summarized in table S14.

The TD-DFT calculation of the triplet states was performed by using the Tamm-Dancoff approximation (TDA) at the ωB97X-D/Def2-TZVP level of theory requesting 10 roots per geometry over the optimized ground state geometries and optimized excited state geometries available in the SI.

Energy transfer rates were calculated energy transfer rates from electronic coupling using the formulation of Iozzi et al.[18] available in Gaussian 16. We optimized the geometries of cis-stilbene (**15**) and trans-stilbene (**14**) in acetonitrile using the methods described above, and used the optimized S_2-state geometry as the reactive geometry for EnT. Two different pairs, **7** and cis-stilbene, and **7** and trans-stilbene were calculated. We conducted a distance scan between the donor and the acceptor. The intermolecular distance between each pair was varied from 5 Å to 15 Å, with 1.0 Å increments. The center of mass of each molecule was taken as reference for the intermolecular distance. The energy transfer rates were calculated by the Fermi's golden rule:

$$k_{i\to j} = \frac{2\pi}{\hbar} V^2 \delta(E_i - E_j),$$

where V is the electronic coupling between the electronic excited state of **7** and the acceptor (cis-stilbene and trans-stilbene), and

$$\delta\left(E_i - E_j\right) = \frac{1}{|E_i - E_j|}.$$

We estimated the photoactivity of energy transfer between **7** and trans-stilbene compared to **7** and cis-stilbene by a photoreactivity ratio:

$$p_{ratio} = \frac{k_{trans-stilbene}}{k_{cis-stilbene}}.$$

The calculation of spin-orbit coupling constants was performed at multiconfigurational pair density functional theory (MC-PDFT).[19] MC-PDFT is a post-complete active space self-consistent field (CASSCF) method with similar accuracy to complete active space second-order perturbation theory (CASPT2). Five singlet roots and five triplet roots were requested. We used atomic Cholesky decomposition[20] to treat the two-electron integrals, and requesting the computation of atomic mean-field integrals. The translated-PBE (T:PBE) on-top functional was used for dynamic correlation energy correction, and the ANO-XS-VDZP basis set was used.[21] MC-PDFT calculations were performed with an (8,11) active space. OpenMolcas 23.02 version was used.

Electronic couplings and energy transfer rates.

In agreement with experimental results, we considered a triplet-triplet Dexter energy transfer mechanism. We calculated the T_1 of **7** to T_1 of cis/trans-stilbene, as well as the T_2 of **7** to T_1 cis/trans-stilbene electronic couplings and energy transfer rates by means of Fermi's golden rule.

Tables S9 and S10 show the electronic couplings. The highest electronic coupling between the donor and the acceptors is at 5.0 Å, except when T_2-**7** is playing a role with cis-stilbene, which highest electronic coupling is at 6.0 Å. However, it is notorious that the electronic coupling at short distances can be from two to five times bigger for trans-stilbene than for cis-stilbene. A clear example is at 5.0 Å, where the electronic coupling between the T_1 of **7** and the T_1 of cis-stilbene is 18.74 meV, while the electronic coupling between the T_1 of **7** and the T_1 of trans-stilbene is 43.29 meV. The electronic coupling order of magnitude changes when compared with the T_2 state of **7**: the electronic coupling between **7** and cis-stilbene is 7.30 meV, while the electronic coupling between **7** and trans-stilbene is -34.81 meV.

The computed EnT rates estimate that the process will be up to 15 times faster when EnT involves trans-stilbene than when EnT involves cis-stilbene and T_1 of **7**, or up to 167 times faster if the EnT starts from T_2 of **7** to T_1 of trans-stilbene over T_1 of cis-stilbene. These data support experimental evidence that **7** preferentially photosensitizes **14** over **15**, even though the calculated ET value for our PCs could activate molecule **15**.

**COUPLINGS**	**Cis-stilbene (15)**	**Trans-stilbene (14)**	
**Intermolecular distance**	V (meV)	V (meV)	p_{ratio}
**5.0**	18.74	43.29	15.3
**6.0**	17.53	34.51	11.1
**7.0**	15.44	-27.76	9.2
**8.0**	13.25	-22.41	8.2
**9.0**	-11.22	-18.18	7.5
**10.0**	-9.47	-14.84	7.0

**11.0**	7.98	-12.20	6.7
**12.0**	6.73	-10.10	6.4
**13.0**	5.70	-8.42	6.2
**14.0**	4.85	7.07	6.1
**15.0**	4.15	5.98	6.0

Table S9: Calculated electronic couplings of the T_1 electronic transition of **7** and cis/trans-stilbene over intermolecular distance and the photoreactivity ratio at the specified distance.

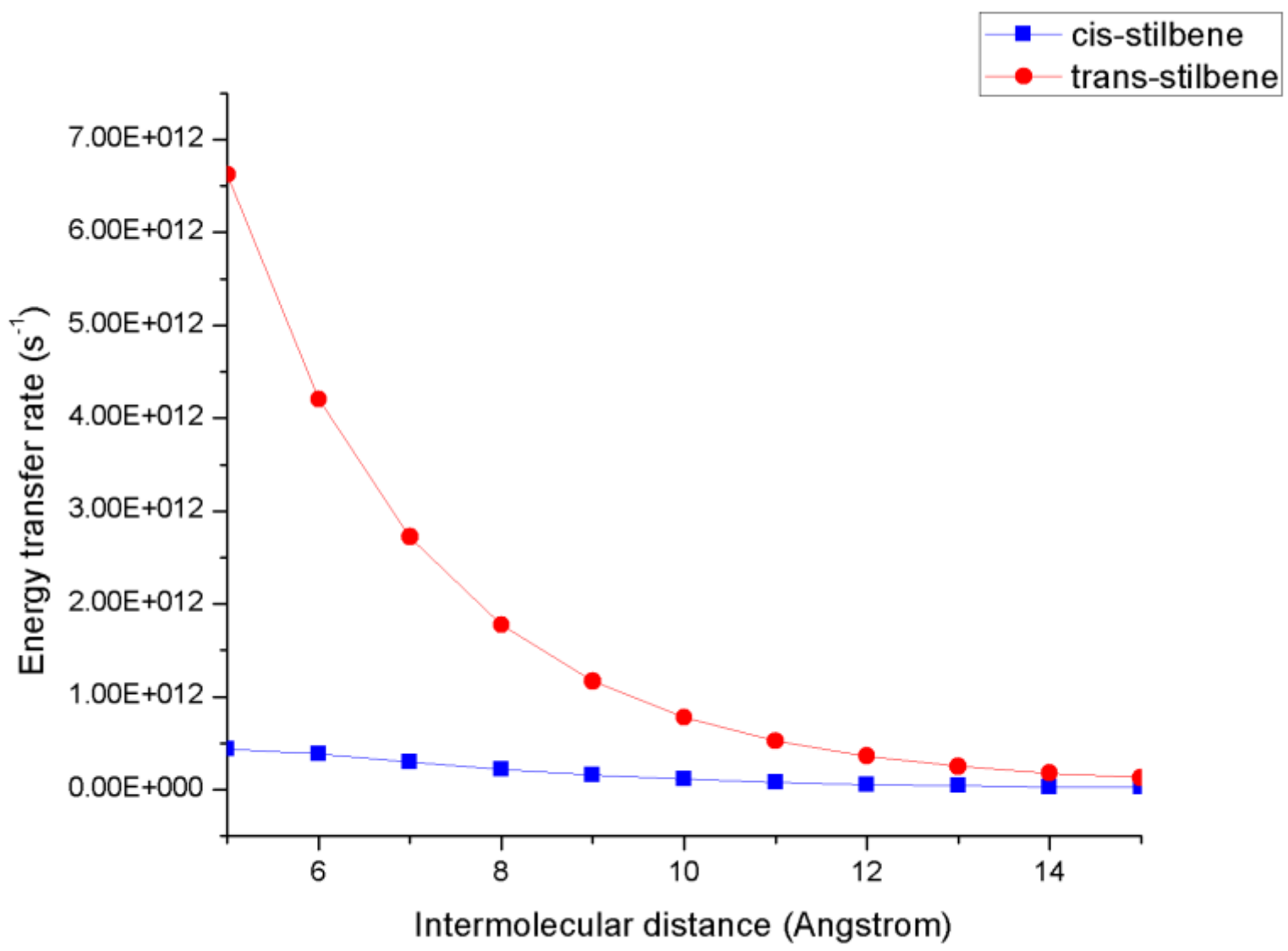

Figure S55: Energy transfer rates of **7** and cis/trans-stilbene. The initial state T_1 of **7** is considered as the donor and the final T_1 state of cis/trans-stilbene is considered as the acceptor.

**COUPLINGS**	**Cis-stilbene (15)**	**Trans-stilbene (14)**	
**Intermolecular distance**	V (meV)	V (meV)	p_{ratio}
**5.0**	7.30	-34.81	167.8
**6.0**	8.61	-27.94	78.8
**7.0**	8.12	22.16	55.7
**8.0**	7.28	17.66	43.9
**9.0**	6.39	14.18	36.7
**10.0**	-5.53	11.48	32.2
**11.0**	4.76	-9.38	29.0
**12.0**	4.09	-7.73	26.6
**13.0**	3.52	6.42	24.8
**14.0**	3.04	-5.38	23.3
**15.0**	-2.64	-4.54	22.1

Table S10: Calculated electronic couplings of the T_2 electronic transition of **7** and cis/trans-stilbene over intermolecular distance and the photoreactivity ratio at the specified distance.

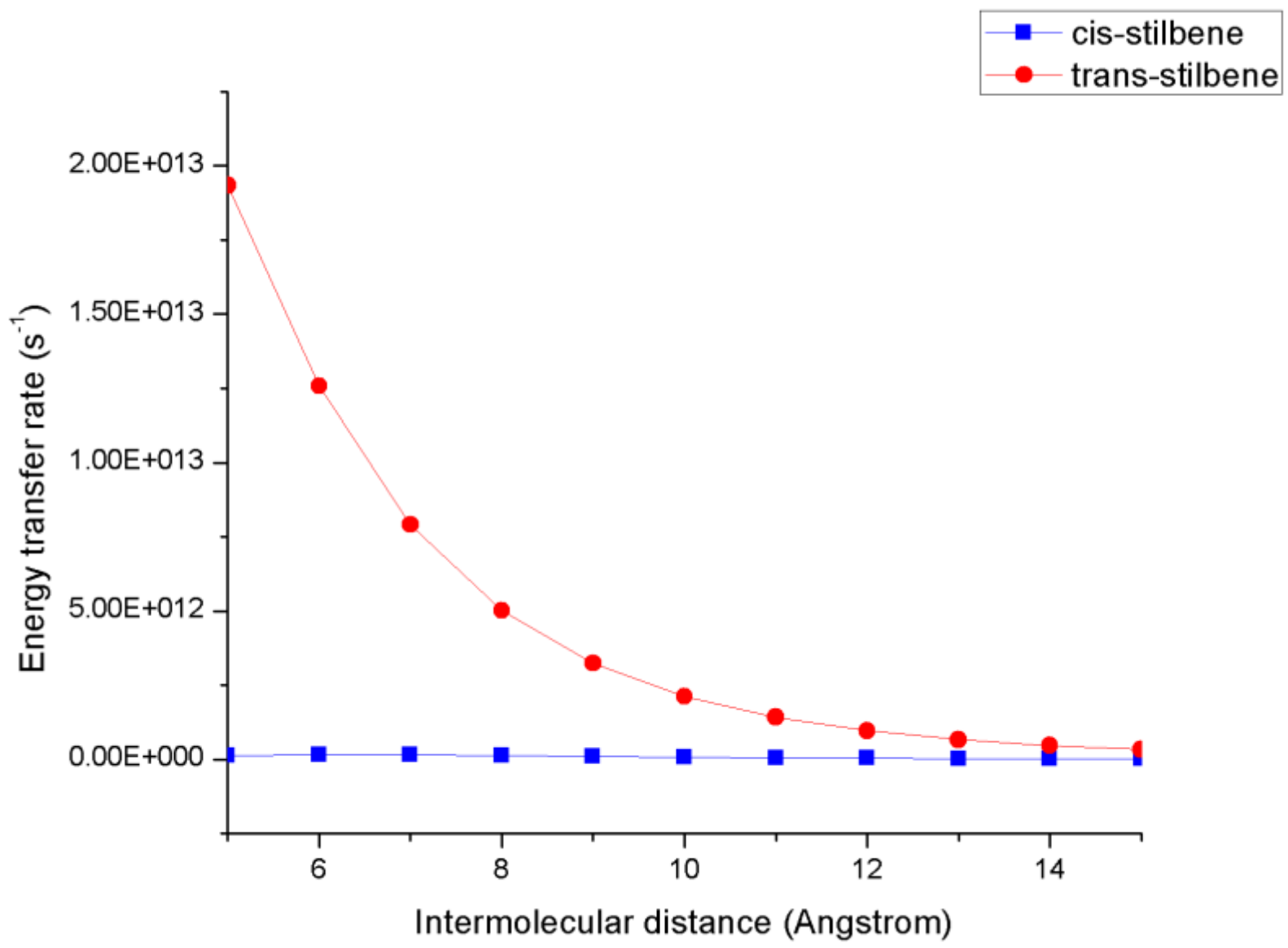

Figure S56: Energy transfer rates of **7** and cis/trans-stilbene. The initial state T_2 of **7** is considered as the donor and the final T_1 state of cis/trans-stilbene is considered as the acceptor.

Spin-orbit coupling constants

CAS(8,11)-PDFT/ANO-XS-VDZP calculations over the optimized ground state geometry of **7** are shown in table S11. The results show the brightest singlet state at 4.26 eV, lower in energy than the S_2 calculated with TD-DFT at 4.59 eV. We used this bright excited state as a reference for the spin-orbit coupling constants, as it has the highest probability of absorbing a photon, and we considered all triplet states.

Energy (eV)	Wavelength (nm)	Multiplicity	f
**3.77**	329	Singlet	0.235052
**3.94**	315	Triplet	0
**3.96**	313	Triplet	0
**4.18**	297	Singlet	0.365191
**4.21**	295	Triplet	0
**4.26**	291	Singlet	0.441672
**4.30**	289	Triplet	0
**4.31**	287	Triplet	0
**4.84**	256	Singlet	0.010141

Table S11: Excitation energies, wavelengths, state multiplicities and oscillator strengths calculated at CAS(8,11)-PDFT/ANO-XS-VDZP level of theory.

Triplet	SOC constant (cm^{-1})	S-T gap (eV)
T_1	1.127	0.32
T_2	0.284	0.30
T_3	0.192	0.06
T_4	0.059	0.03
T_5	0.399	0.05

Table S12: Spin-orbit coupling constants and singlet-triplet gaps in **7**.

Spin-orbit coupling constants are within 0.192 and 1.127 cm^{-1}. Small SOC values are common in organic molecules due the absence of heavy atoms. However, the presence of nitrogens enhance nonzero SOC values.[22] The SOC values presented in Table S12 are related to molecules in which phosphorescence decay competes with fluorescence. Experiments show that phosphorescence is promoted at 77 K, which is consistent, as low temperatures affect the vibrational modes and allow singlet-triplet electronic changes to occur. Hence, ISC occurs in **7**, slowed by the effect of a small SOC between the singlet state of interest and the triplet states.

Ground state optimized geometries:

Optimized ground state coordinates of 4.

Energy = -1210.63634 Ha

N	-0.00000000	0.36236700	2.46432400
H	-0.00000000	-0.07386100	3.37490500
C	-1.19581500	0.07058300	1.76867200
C	-2.07022600	-0.89124800	2.26312300
H	-1.82143300	-1.41245800	3.17994400
C	-3.23734800	-1.20171200	1.59148700
H	-3.89047800	-1.96712300	1.99149400
C	-3.54881600	-0.57783600	0.38559000
C	-2.67298700	0.39212800	-0.09004400
H	-2.91157200	0.90759800	-1.01218500
C	-1.51231300	0.75235800	0.58975700
C	-0.70117400	1.89903200	0.11362500
C	-1.37377300	3.02933300	-0.35714100
H	-2.45607200	3.04216300	-0.33731000
C	-0.69323300	4.13747800	-0.82650700
H	-1.24339200	5.00113000	-1.17734800
C	0.69323400	4.13747900	-0.82650700
H	1.24339200	5.00113000	-1.17734800
C	1.37377300	3.02933300	-0.35714100
H	2.45607200	3.04216300	-0.33730900
C	0.70117400	1.89903200	0.11362600
C	1.51231300	0.75235800	0.58975700
C	2.67298700	0.39212800	-0.09004300
H	2.91157200	0.90759800	-1.01218500
C	3.54881600	-0.57783600	0.38559000
C	3.23734800	-1.20171200	1.59148700
H	3.89047800	-1.96712300	1.99149400
C	2.07022500	-0.89124800	2.26312300
H	1.82143300	-1.41245800	3.17994400
C	1.19581500	0.07058300	1.76867200
C	-4.77549700	-0.93045200	-0.36736200
C	-4.76209900	-1.01036600	-1.76008200
C	-5.97292800	-1.19142700	0.29920300
C	-5.90990700	-1.33908700	-2.46456400
H	-3.83911000	-0.83155800	-2.29808700
C	-7.12083900	-1.52232300	-0.40407000
H	-6.01236700	-1.11688700	1.37909800
C	-7.09446300	-1.59699100	-1.78946000
H	-5.87606900	-1.40141900	-3.54506600
H	-8.04132500	-1.71503500	0.13268200

H	-7.99078000	-1.85458300	-2.33917700
C	4.77549700	-0.93045200	-0.36736200
C	4.76209900	-1.01036500	-1.76008200
C	5.97292800	-1.19142800	0.29920300
C	5.90990700	-1.33908600	-2.46456400
H	3.83911100	-0.83155700	-2.29808700
C	7.12083900	-1.52232300	-0.40407000
H	6.01236700	-1.11688800	1.37909800
C	7.09446300	-1.59699000	-1.78946000
H	5.87606900	-1.40141800	-3.54506600
H	8.04132400	-1.71503700	0.13268200
H	7.99078000	-1.85458300	-2.33917700

Optimized ground state geometry of 5.

Energy = -1439.70842 Ha

N	0.00000000	1.84816500	2.14200400
H	0.00000000	1.88862700	3.15089700
C	-1.19573800	1.26369000	1.66333500
C	-2.06979500	0.64172500	2.54826700
H	-1.82097000	0.60686100	3.60239900
C	-3.23670200	0.05308000	2.09859900
H	-3.88832600	-0.43958700	2.80947300
C	-3.55154600	0.04518800	0.74133600
C	-2.67487100	0.68279100	-0.13038200
H	-2.91393500	0.71135600	-1.18638800
C	-1.51277200	1.31661800	0.30273600
C	-0.70127200	2.10825900	-0.65378200
C	-1.37363100	2.88809800	-1.59807200
H	-2.45589700	2.90912500	-1.58637000
C	-0.69323500	3.64853000	-2.53092500
H	-1.24345100	4.24817000	-3.24462900
C	0.69323300	3.64853100	-2.53092500
H	1.24344900	4.24817000	-3.24462900
C	1.37363000	2.88809800	-1.59807200
H	2.45589600	2.90912500	-1.58637100
C	0.70127100	2.10825900	-0.65378300
C	1.51277200	1.31661800	0.30273600
C	2.67487100	0.68279200	-0.13038200
H	2.91393500	0.71135700	-1.18638900
C	3.55154600	0.04518900	0.74133500
C	3.23670300	0.05308100	2.09859900
H	3.88832700	-0.43958500	2.80947200
C	2.06979600	0.64172600	2.54826700
H	1.82097100	0.60686200	3.60239900
C	1.19573800	1.26369000	1.66333500
C	-4.77994700	-0.61260800	0.23968000
C	-4.78107200	-1.32165600	-0.96480800
C	-5.97451000	-0.54610000	0.94764300
C	-5.92379700	-1.93339900	-1.43726300
H	-3.86480000	-1.41202100	-1.53548800
C	-7.13423200	-1.15627000	0.48779100
H	-6.01758500	0.01070800	1.87587200
C	-7.11349400	-1.85573000	-0.71386500
H	-5.91152600	-2.48704100	-2.36744500
H	-8.04049400	-1.06989600	1.06964500

C	4.77994800	-0.61260700	0.23967900
C	4.78107300	-1.32165400	-0.96481000
C	5.97451100	-0.54609800	0.94764300
C	5.92379800	-1.93339700	-1.43726400
H	3.86480000	-1.41201900	-1.53548900
C	7.13423300	-1.15626800	0.48779000
H	6.01758500	0.01070900	1.87587100
C	7.11349400	-1.85572900	-0.71386600
H	5.91152600	-2.48703900	-2.36744700
H	8.04049500	-1.06989500	1.06964400
O	8.18279000	-2.48459700	-1.25367300
O	-8.18279000	-2.48459900	-1.25367200
C	-9.41074700	-2.43877500	-0.55344100
H	-9.76692900	-1.41109800	-0.44264600
H	-10.12148000	-3.00267600	-1.15211300
H	-9.32281100	-2.90068100	0.43352900
C	9.41074500	-2.43878100	-0.55343800
H	10.12147800	-3.00268500	-1.15210800
H	9.76693200	-1.41110600	-0.44264100
H	9.32280300	-2.90068600	0.43353100

Optimized ground state geometry of 6.

Energy = -1517.91861 Ha

N	-0.00000000	-1.93492900	2.40320800
H	-0.00000000	-1.81586800	3.40578600
C	1.19522800	-1.42990700	1.84012500
C	2.06645900	-0.67592800	2.61928700
H	1.82392800	-0.49206500	3.65937200
C	3.23304200	-0.16269900	2.08532700
H	3.90314400	0.40341300	2.72001900
C	3.54757200	-0.36509500	0.74338000
C	2.67624400	-1.13458600	-0.01976500
H	2.89745500	-1.29744100	-1.06735500
C	1.51477500	-1.69585000	0.50557200
C	0.70118700	-2.61915800	-0.32160800
C	1.37366400	-3.53034900	-1.13990100
H	2.45602100	-3.54885600	-1.12533000
C	0.69326800	-4.42080100	-1.94941500
H	1.24347200	-5.11968000	-2.56624600
C	-0.69326700	-4.42080000	-1.94941600
H	-1.24347100	-5.11967900	-2.56624700
C	-1.37366400	-3.53034900	-1.13990200
H	-2.45602100	-3.54885500	-1.12533100
C	-0.70118700	-2.61915700	-0.32160800
C	-1.51477500	-1.69585000	0.50557100
C	-2.67624400	-1.13458600	-0.01976600
H	-2.89745400	-1.29744000	-1.06735600
C	-3.54757200	-0.36509500	0.74338000
C	-3.23304300	-0.16270000	2.08532700
H	-3.90314600	0.40341200	2.72001900
C	-2.06646000	-0.67592900	2.61928700
H	-1.82392900	-0.49206700	3.65937200
C	-1.19522800	-1.42990700	1.84012600
C	-4.77013200	0.21891900	0.14408900
C	-5.19483700	1.47792900	0.48512000

C	-5.52743800	-0.52085500	-0.80115100
C	-6.36032500	2.04576900	-0.07923900
H	-4.62332600	2.07155600	1.18952200
C	-6.65525900	-0.00077100	-1.36366100
H	-5.21258700	-1.52328900	-1.06343400
C	-6.80210000	3.34681000	0.26557400
C	-7.10663700	1.29666300	-1.02236300
H	-7.22645700	-0.58343500	-2.07683600
C	-7.93075400	3.87142000	-0.29695300
H	-6.22932700	3.91976900	0.98536800
C	-8.27233700	1.86631200	-1.58767300
C	-8.67520400	3.12288600	-1.23412000
H	-8.26020500	4.86643500	-0.02558300
H	-8.84045700	1.28848300	-2.30728800
H	-9.56793400	3.55103200	-1.67207500
C	4.77013200	0.21891900	0.14409000
C	5.19483700	1.47792900	0.48512000
C	5.52743800	-0.52085500	-0.80115100
C	6.36032500	2.04576900	-0.07923900
H	4.62332500	2.07155600	1.18952200
C	6.65526000	-0.00077100	-1.36366000
H	5.21258800	-1.52328900	-1.06343300
C	6.80210000	3.34681000	0.26557400
C	7.10663800	1.29666300	-1.02236300
H	7.22645700	-0.58343600	-2.07683500
C	7.93075400	3.87142000	-0.29695300
H	6.22932700	3.91976900	0.98536700
C	8.27233800	1.86631200	-1.58767300
C	8.67520500	3.12288600	-1.23412000
H	8.26020500	4.86643500	-0.02558300
H	8.84045700	1.28848200	-2.30728800
H	9.56793400	3.55103200	-1.67207500

Optimized ground state geometry of 7.

Energy = -1395.12921 Ha

N	0.00000000	2.01101800	1.95964600
H	0.00000100	2.16464300	2.95749300
C	-1.19701400	1.38648300	1.54564900
C	-2.07573900	0.87613500	2.49586400
H	-1.82783200	0.96050700	3.54711300
C	-3.24570100	0.24869900	2.11527700
H	-3.90176800	-0.15395600	2.87657000
C	-3.55167900	0.08413000	0.76637900
C	-2.67275500	0.61053600	-0.17388200
H	-2.90972800	0.51794000	-1.22645900
C	-1.51190400	1.28833400	0.18650600
C	-0.70092600	1.96869000	-0.85203000
C	-1.37393100	2.63904900	-1.87611300
H	-2.45616800	2.66143800	-1.86681100
C	-0.69318300	3.29181900	-2.88697300
H	-1.24319000	3.80904500	-3.66251100
C	0.69318000	3.29181800	-2.88697400
H	1.24318700	3.80904500	-3.66251200
C	1.37392900	2.63904900	-1.87611400
H	2.45616600	2.66143700	-1.86681300

C	0.70092400	1.96869000	-0.85203100
C	1.51190400	1.28833300	0.18650500
C	2.67275400	0.61053500	-0.17388500
H	2.90972600	0.51793900	-1.22646100
C	3.55167800	0.08412800	0.76637600
C	3.24570100	0.24869700	2.11527400
H	3.90176900	-0.15395800	2.87656600
C	2.07574000	0.87613400	2.49586200
H	1.82783400	0.96050600	3.54711100
C	1.19701400	1.38648200	1.54564800
C	-4.77827900	-0.62564100	0.34192200
C	-4.76667500	-1.46800100	-0.77122100
C	-5.97298500	-0.46803700	1.04658000
C	-5.90841000	-2.13277000	-1.17322700
H	-3.84695200	-1.62197000	-1.32017300
C	-7.12163500	-1.12847900	0.65757300
H	-6.01093800	0.19559900	1.90038300
C	-7.09216900	-1.96457400	-0.45713400
H	-5.88452700	-2.78772700	-2.03359800
H	-8.04246000	-0.99264000	1.20836600
C	4.77827800	-0.62564300	0.34191700
C	4.76667200	-1.46800300	-0.77122500
C	5.97298400	-0.46803900	1.04657400
C	5.90840600	-2.13277300	-1.17323400
H	3.84694800	-1.62197200	-1.32017600
C	7.12163300	-1.12848200	0.65756600
H	6.01093800	0.19559700	1.90037700
C	7.09216600	-1.96457700	-0.45714200
H	5.88452200	-2.78773000	-2.03360500
H	8.04245900	-0.99264300	1.20835700
C	-8.27877200	-2.65205400	-0.86592200
N	-9.23197200	-3.20500200	-1.19368400
C	8.27876500	-2.65206000	-0.86593500
N	9.23199800	-3.20498000	-1.19364700

Optimized ground state geometry of 8.

Energy = -1884.84953 Ha

N	-0.00000200	-2.72699400	-1.68671300
H	-0.00000200	-3.03201600	-2.64900900
C	-1.19724000	-2.04454900	-1.37639100
C	-2.07651100	-1.69163800	-2.39501800
H	-1.82910000	-1.94299200	-3.41953200
C	-3.24653100	-1.01132300	-2.11837600
H	-3.90308600	-0.73482400	-2.93373400
C	-3.55315100	-0.63540500	-0.81280800
C	-2.67424900	-1.00736500	0.19832600
H	-2.91161600	-0.74907100	1.22284900
C	-1.51236900	-1.73266100	-0.05014400
C	-0.70097600	-2.23928400	1.08310000
C	-1.37380200	-2.73835300	2.20092300
H	-2.45606200	-2.76183100	2.19528600
C	-0.69315900	-3.22195000	3.30283800
H	-1.24327300	-3.60906900	4.15082100
C	0.69323100	-3.22193300	3.30282900
H	1.24336500	-3.60904000	4.15080500

C	1.37384900	-2.73832100	2.20090600
H	2.45610900	-2.76177300	2.19525600
C	0.70099700	-2.23926700	1.08309100
C	1.51236100	-1.73262000	-0.05016300
C	2.67422900	-1.00730000	0.19829400
H	2.91160800	-0.74901100	1.22281500
C	3.55310400	-0.63530800	-0.81285200
C	3.24646400	-1.01121400	-2.11841900
H	3.90299300	-0.73468700	-2.93378800
C	2.07645800	-1.69155800	-2.39504700
H	1.82903500	-1.94290800	-3.41955800
C	1.19721800	-2.04450800	-1.37640600
C	-4.77996100	0.13393900	-0.50500300
C	-4.76785500	1.14567900	0.45250300
C	-5.97856700	-0.13719300	-1.16801600
C	-5.91409800	1.86611800	0.74547700
H	-3.84693800	1.38970100	0.96618600
C	-7.12479500	0.57898200	-0.88304800
H	-6.02006900	-0.93116200	-1.90240100
C	-7.09431100	1.58311900	0.07734300
H	-5.87986300	2.65047200	1.48863800
H	-8.04596800	0.34920200	-1.40325900
C	4.77991400	0.13404200	-0.50506100
C	4.76784000	1.14573700	0.45249200
C	5.97849500	-0.13704700	-1.16814000
C	5.91409000	1.86617600	0.74545100
H	3.84694300	1.38973100	0.96622400
C	7.12472300	0.57913300	-0.88319600
H	6.01997400	-0.93097900	-1.90256600
C	7.09426900	1.58323500	0.07723400
H	5.87987800	2.65049800	1.48864500
H	8.04587500	0.34939700	-1.40346200
C	-8.35400000	2.34797200	0.36329100
C	8.35403500	2.34791100	0.36330300
F	-9.36130800	1.53528000	0.73174500
F	-8.20507500	3.24938200	1.34119300
F	-8.78999700	3.01778700	-0.72022600
F	9.36071000	1.53520600	0.73351200
F	8.20470700	3.25049100	1.34006000
F	8.79118700	3.01628000	-0.72062800

Excited state geometries

Optimized S_1 state coordinates of 4.

Energy = -1210.60669 Ha

N	-0.00002600	-1.79258700	0.92571900
H	-0.00003200	-2.77916200	1.14565600
C	-1.25224500	-1.31169400	0.70226000
C	-2.26678300	-2.29448700	0.71038600
H	-1.99094900	-3.31633400	0.94597900
C	-3.55576200	-2.00663500	0.38153300
H	-4.29551800	-2.79486700	0.35865700
C	-3.88985600	-0.68593300	-0.01840200
C	-2.91997700	0.28318100	0.03891600
H	-3.19455500	1.28118600	-0.26767200

C	-1.58210800	0.06793300	0.47295400
C	-0.72823600	1.18601300	0.72959500
C	-1.37047700	2.43419000	0.99304500
H	-2.44734100	2.44959400	1.07532300
C	-0.70592100	3.59944900	1.22281800
H	-1.25751500	4.50233400	1.45147000
C	0.70607800	3.59940900	1.22285200
H	1.25771200	4.50226500	1.45152100
C	1.37058200	2.43410900	0.99313800
H	2.44744200	2.44946900	1.07545900
C	0.72828800	1.18597000	0.72962800
C	1.58209000	0.06787600	0.47282000
C	2.91995700	0.28309700	0.03876000
H	3.19458700	1.28111200	-0.26774500
C	3.88978000	-0.68606300	-0.01868200
C	3.55565600	-2.00676900	0.38121300
H	4.29538800	-2.79502200	0.35829800
C	2.26668700	-2.29457900	0.71014100
H	1.99083000	-3.31642000	0.94572800
C	1.25218300	-1.31175000	0.70207400
C	-5.25495600	-0.36805600	-0.48865400
C	-5.46831700	0.54697500	-1.52179900
C	-6.36812200	-0.97742300	0.09407500
C	-6.75066100	0.84875200	-1.95110100
H	-4.62066300	1.01187900	-2.00973400
C	-7.65093400	-0.67408900	-0.33359400
H	-6.23191400	-1.68091900	0.90608700
C	-7.84841400	0.24069600	-1.35816600
H	-6.89228600	1.55599400	-2.75869400
H	-8.49999100	-1.15148100	0.13928200
H	-8.85020300	0.47565800	-1.69446700
C	5.25492100	-0.36817000	-0.48880300
C	5.46838400	0.54699100	-1.52180900
C	6.36802800	-0.97757600	0.09399400
C	6.75077600	0.84886800	-1.95090200
H	4.62077900	1.01194400	-2.00978200
C	7.65088700	-0.67414200	-0.33346300
H	6.23173700	-1.68116000	0.90591600
C	7.84847100	0.24077900	-1.35789300
H	6.89248500	1.55622400	-2.75838000
H	8.49989700	-1.15155800	0.13947300
H	8.85029800	0.47582900	-1.69402100

Optimized S_1 state coordinates of 5.

Energy = -1439.67879 Ha

N	0.00003900	1.86721400	1.06593800
H	0.00003900	2.86170700	1.24649400
C	1.25234900	1.37789900	0.85897400
C	2.26701600	2.35945700	0.82486000
H	1.99189700	3.39029400	1.01872700
C	3.55549500	2.05885900	0.50424600
H	4.29297500	2.84737200	0.44556600
C	3.89147800	0.72315700	0.15835400
C	2.91952300	-0.24206900	0.25878100
H	3.19250300	-1.25296100	-0.00377400

C	1.58259400	-0.00914800	0.68435700
C	0.72805300	-1.11582600	0.98863000
C	1.37005700	-2.35148800	1.30524200
H	2.44704900	-2.36297200	1.38799700
C	0.70580200	-3.50654300	1.58401000
H	1.25760700	-4.39874500	1.85102500
C	-0.70614900	-3.50656100	1.58356500
H	-1.25809800	-4.39879400	1.85018200
C	-1.37027700	-2.35150500	1.30452000
H	-2.44731300	-2.36305900	1.38665800
C	-0.72813000	-1.11579000	0.98833100
C	-1.58249500	-0.00907100	0.68378100
C	-2.91938300	-0.24187000	0.25795000
H	-3.19238400	-1.25268600	-0.00487300
C	-3.89134000	0.72337500	0.15779700
C	-3.55536300	2.05903100	0.50385700
H	-4.29283600	2.84755400	0.44524100
C	-2.26688200	2.35955400	0.82458400
H	-1.99174800	3.39035500	1.01861800
C	-1.25226200	1.37796000	0.85869400
C	5.25380000	0.38825300	-0.30214800
C	5.47680100	-0.59990400	-1.26792400
C	6.36880800	1.04310300	0.21320600
C	6.74992700	-0.91960900	-1.68860200
H	4.63536900	-1.11381300	-1.71579600
C	7.65787600	0.73107500	-0.19606500
H	6.24229400	1.80444300	0.97290600
C	7.85562800	-0.25765600	-1.15395000
H	6.90901100	-1.67853800	-2.44393900
H	8.49142500	1.26047200	0.24239000
C	-5.25371400	0.38851100	-0.30261500
C	-5.47680100	-0.59930600	-1.26871400
C	-6.36867800	1.04298800	0.21330300
C	-6.74999500	-0.91906100	-1.68916500
H	-4.63540000	-1.11292600	-1.71697800
C	-7.65781000	0.73089700	-0.19572900
H	-6.24207400	1.80406000	0.97325700
C	-7.85566000	-0.25751100	-1.15393200
H	-6.90916000	-1.67773200	-2.44474200
H	-8.49132000	1.25998500	0.24317000
O	-9.06399200	-0.63760000	-1.62519500
O	9.06388700	-0.63768700	-1.62542800
C	10.21556500	0.01006200	-1.11988200
H	10.31681300	-0.14408700	-0.04240900
H	11.06360000	-0.44071200	-1.62882000
H	10.19370200	1.08203000	-1.33302900
C	-10.21566200	0.00959700	-1.11892200
H	-11.06376500	-0.44121200	-1.62771700
H	-10.31643600	-0.14504300	-0.04147500
H	-10.19420500	1.08166100	-1.33161900

Optimized S_1 state coordinates of 6.

Energy = -1517.88879 Ha

N	0.00002700	-0.77297300	-2.01351800
H	0.00002700	-1.55285100	-2.65636600

C	1.25274700	-0.44543600	-1.59872100
C	2.26753100	-1.31152800	-2.06405000
H	1.99467000	-2.09430000	-2.76324500
C	3.55611300	-1.21041900	-1.63943900
H	4.30585400	-1.88963500	-2.02085000
C	3.89473700	-0.22068800	-0.67861300
C	2.92469400	0.66538200	-0.28163900
H	3.18387700	1.37986200	0.48515600
C	1.58444000	0.67279400	-0.76042800
C	0.72782600	1.77700500	-0.45629100
C	1.37012000	3.00313700	-0.10443000
H	2.44688700	3.05574300	-0.17162500
C	0.70589600	4.13959700	0.24150100
H	1.25763300	5.04391300	0.46401600
C	-0.70579600	4.13959600	0.24157500
H	-1.25750600	5.04390600	0.46417900
C	-1.37007900	3.00312900	-0.10422700
H	-2.44684700	3.05579000	-0.17130100
C	-0.72785200	1.77699900	-0.45622800
C	-1.58447900	0.67282200	-0.76051400
C	-2.92475900	0.66540000	-0.28179400
H	-3.18406200	1.38002900	0.48481300
C	-3.89472200	-0.22084000	-0.67858900
C	-3.55595300	-1.21087900	-1.63903800
H	-4.30558600	-1.89035100	-2.02020200
C	-2.26737400	-1.31189900	-2.06366300
H	-1.99441000	-2.09485500	-2.76261600
C	-1.25270000	-0.44551500	-1.59863300
C	-5.25552000	-0.16553000	-0.10577500
C	-5.98956800	-1.31088800	0.08598100
C	-5.83686100	1.07843400	0.25971100
C	-7.28886000	-1.27727000	0.64119500
H	-5.56791900	-2.27648100	-0.16764000
C	-7.08849800	1.14347600	0.79410300
H	-5.28455200	1.99321700	0.08641300
C	-8.04447200	-2.45734400	0.84978000
C	-7.85321200	-0.02957400	1.00474300
H	-7.51857300	2.10307600	1.05592500
C	-9.29823700	-2.39569700	1.38824100
H	-7.61126000	-3.41153400	0.57356600
C	-9.15458200	0.00320300	1.55824300
C	-9.86059400	-1.15158200	1.74613400
H	-9.86767900	-3.30343900	1.54282800
H	-9.58266400	0.96035400	1.83219500
H	-10.85554600	-1.11728500	2.17149400
C	5.25549800	-0.16544500	-0.10571100
C	5.98963900	-1.31079700	0.08570100
C	5.83670600	1.07845400	0.26020700
C	7.28888700	-1.27724600	0.64102100
H	5.56809500	-2.27634200	-0.16828300
C	7.08829800	1.14343200	0.79471400
H	5.28432600	1.99324400	0.08717500
C	8.04458500	-2.45732200	0.84928500
C	7.85309900	-0.02962000	1.00502700
H	7.51827300	2.10298400	1.05687600

C	9.29829800	-2.39574200	1.38787400
H	7.61147900	-3.41146000	0.57272600
C	9.15442600	0.00309100	1.55863200
C	9.86051900	-1.15169400	1.74621200
H	9.86780400	-3.30348500	1.54222100
H	9.58240600	0.96019200	1.83292100
H	10.85543600	-1.11744700	2.17165900

Optimized S_1 state coordinates of 7.

Energy = -1395.10053 Ha

N	-0.00494100	-1.82582900	-1.21331200
H	-0.00691800	-2.80484900	-1.46563000
C	1.24806200	-1.35320100	-0.98265600
C	2.26104500	-2.33827600	-1.01994000
H	1.98632900	-3.34840300	-1.30228300
C	3.54896600	-2.06394400	-0.68083800
H	4.29630100	-2.84393300	-0.72295600
C	3.88831600	-0.75106400	-0.25744900
C	2.92222600	0.22278900	-0.29055100
H	3.18481300	1.20132700	0.08275500
C	1.58165300	0.01968400	-0.71832300
C	0.72517700	1.14734900	-0.92197200
C	1.36705000	2.40178500	-1.15104700
H	2.44248900	2.41840200	-1.24917600
C	0.70083300	3.57510400	-1.32982600
H	1.25039700	4.48512700	-1.53319500
C	-0.70988300	3.57652500	-1.31062800
H	-1.26293500	4.48769400	-1.49882200
C	-1.37365500	2.40432500	-1.11498900
H	-2.45121900	2.42370800	-1.18472800
C	-0.72865200	1.14858300	-0.90451700
C	-1.58010100	0.01981100	-0.68323700
C	-2.91002400	0.21943000	-0.22643600
H	-3.17779200	1.20535700	0.12181500
C	-3.88179500	-0.75003400	-0.20286000
C	-3.55732000	-2.05369000	-0.66420900
H	-4.29792000	-2.84116600	-0.66926700
C	-2.27265200	-2.33007300	-1.01518100
H	-2.00195700	-3.34160100	-1.29616300
C	-1.25451400	-1.35090000	-0.97169000
C	5.24793700	-0.44945300	0.23029500
C	5.83700000	0.79776800	0.00254200
C	5.98124600	-1.41154100	0.93063700
C	7.10580400	1.08235400	0.46458600
H	5.30505400	1.54969800	-0.56486000
C	7.25128500	-1.13995400	1.39860600
H	5.54459600	-2.38036600	1.13339900
C	7.81904500	0.11172700	1.16706200
H	7.55099400	2.04960900	0.27498000
H	7.80238700	-1.89123000	1.94780900
C	-5.23926600	-0.44421000	0.28615500
C	-5.44339200	0.45297500	1.33871300
C	-6.35680100	-1.04879100	-0.29664500
C	-6.71438300	0.74635400	1.78917500
H	-4.59479800	0.91135900	1.82879700

C	-7.63346200	-0.76331800	0.14367600
H	-6.22964200	-1.73631900	-1.12217800
C	-7.81733000	0.13802200	1.19102300
H	-6.85588300	1.43727100	2.60921900
H	-8.48880700	-1.23170700	-0.32409500
C	9.13593300	0.39902700	1.64623600
N	10.19425600	0.63003000	2.03177200
C	-9.13717300	0.43509700	1.65541400
N	-10.19825200	0.67415100	2.02837400

Optimized S_1 state coordinates of 8.

Energy = -1884.84307 Ha

N	0.00000800	-2.61794600	-1.37854300
H	-0.00003300	-3.07802900	-2.27690700
C	-1.21092700	-1.94319600	-1.15076500
C	-2.12722100	-1.81340300	-2.18496400
H	-1.86900100	-2.20397800	-3.16256100
C	-3.36012800	-1.19502000	-2.00893900
H	-4.02677400	-1.09936600	-2.85531700
C	-3.70011400	-0.65223300	-0.76434300
C	-2.78674700	-0.80647400	0.27481200
H	-3.04477900	-0.41391600	1.24889000
C	-1.53873300	-1.46128800	0.13752100
C	-0.71500500	-1.67621800	1.31573900
C	-1.37441300	-1.85820000	2.55186800
H	-2.45537900	-1.89016200	2.56880400
C	-0.69203100	-2.04372500	3.75254200
H	-1.24898500	-2.19356300	4.66867500
C	0.69179100	-2.04286500	3.75260100
H	1.24885500	-2.19193200	4.66879100
C	1.37407100	-1.85655000	2.55202700
H	2.45506600	-1.88743400	2.56912700
C	0.71460800	-1.67542800	1.31577400
C	1.53842800	-1.46086200	0.13750600
C	2.78634500	-0.80579800	0.27467100
H	3.04358000	-0.41161200	1.24829800
C	3.70050000	-0.65313900	-0.76403900
C	3.36146200	-1.19790600	-2.00800300
H	4.02883800	-1.10375200	-2.85397700
C	2.12843600	-1.81613700	-2.18413000
H	1.87082000	-2.20793400	-3.16138700
C	1.21119100	-1.94393300	-1.15048400
C	-4.98181300	0.03104400	-0.53449800
C	-5.09498100	1.07861000	0.38662400
C	-6.13138200	-0.34533500	-1.24461100
C	-6.30136800	1.71995500	0.59867500
H	-4.22291500	1.41815100	0.92993200
C	-7.33612500	0.29209400	-1.03686600
H	-6.08485700	-1.16406900	-1.95037700
C	-7.42752100	1.32961900	-0.11294600
H	-6.35849400	2.53091400	1.31128800
H	-8.21192700	-0.02266300	-1.59014900
C	4.98204600	0.03044100	-0.53423900
C	5.09453500	1.07917600	0.38560300
C	6.13211200	-0.34679100	-1.24305500

C	6.30086700	1.72052800	0.59808200
H	4.22205300	1.41946400	0.92777300
C	7.33669600	0.29090100	-1.03519100
H	6.08614800	-1.16634000	-1.94790800
C	7.42752800	1.32931500	-0.11222100
H	6.35747100	2.53222500	1.30988800
H	8.21288300	-0.02448900	-1.58752500
C	-8.75301800	1.99728100	0.09623400
C	8.75267200	1.99786500	0.09624300
F	-9.67668000	1.14275100	0.57933800
F	-8.69037500	3.02267200	0.95450600
F	-9.26123600	2.48141600	-1.05314700
F	9.67943400	1.14201400	0.57080900
F	8.69144200	3.01807300	0.96074100
F	9.25594800	2.48975600	-1.05212900

Optimized S_3 state coordinates of 4.

Energy = -1210.62601 Ha

N	0.00061000	0.86665500	2.16668100
H	-0.00229000	1.64791700	2.80493600
C	1.22437700	0.76508800	1.49252400
C	2.16256600	1.78086100	1.64996300
H	1.90923600	2.62124400	2.28722700
C	3.41167800	1.75918200	1.03700100
H	4.10244400	2.56913800	1.22087500
C	3.75499000	0.68186500	0.20555100
C	2.80984100	-0.35231300	0.08065600
H	3.03587900	-1.16610800	-0.59359200
C	1.55325400	-0.37589800	0.72123600
C	0.72342300	-1.55549900	0.59762700
C	1.37425600	-2.80268700	0.44775200
H	2.45420500	-2.83508700	0.50242400
C	0.69670100	-3.98317600	0.29345400
H	1.24368600	-4.91283000	0.20329900
C	-0.70968800	-3.98221300	0.28377500
H	-1.25970700	-4.90871700	0.18486900
C	-1.37974300	-2.79433400	0.42897700
H	-2.46055200	-2.81655300	0.46257600
C	-0.72390900	-1.55510300	0.59030500
C	-1.55605200	-0.37132200	0.71237500
C	-2.81212700	-0.34689100	0.06434400
H	-3.04678500	-1.15604400	-0.61380900
C	-3.74140900	0.68273100	0.19952700
C	-3.40347800	1.74923200	1.03345500
H	-4.09916600	2.55950000	1.20622900
C	-2.15790800	1.77653000	1.65496000
H	-1.90857500	2.61626200	2.29378300
C	-1.22457400	0.76184200	1.49047400
C	5.01541700	0.62614600	-0.53054600
C	5.56868500	-0.59230500	-0.96741100
C	5.73827500	1.80216500	-0.80933600
C	6.76936900	-0.63127900	-1.64825500
H	5.06754400	-1.52571800	-0.74660500
C	6.93808000	1.75806200	-1.49424900
H	5.33794200	2.76221400	-0.51185100

C	7.46491200	0.54404300	-1.91954500
H	7.17283100	-1.58516500	-1.96386900
H	7.46602000	2.67981800	-1.70515600
H	8.40586900	0.51243300	-2.45362200
C	-5.02414900	0.64050400	-0.53125600
C	-5.68557400	-0.56870100	-0.76280900
C	-5.61486900	1.81516200	-1.00443600
C	-6.88938600	-0.60247600	-1.44728100
H	-5.26558900	-1.49266500	-0.38450300
C	-6.81966100	1.78155400	-1.68772300
H	-5.11464400	2.76404900	-0.85528400
C	-7.46296300	0.57260800	-1.91361900
H	-7.38629700	-1.55074500	-1.60951200
H	-7.25420200	2.70393600	-2.05234100
H	-8.40414100	0.54626900	-2.44778200

Optimized S_3 state coordinates of 5.

Energy = -1439.69649 Ha

N	-0.00481700	1.65536700	2.34859400
H	-0.00585200	1.61666700	3.35798500
C	-1.20506100	1.16327200	1.80329900
C	-2.10759400	0.47323300	2.61902700
H	-1.87950800	0.35270200	3.67188500
C	-3.25779300	-0.07481600	2.10802800
H	-3.90440400	-0.62047800	2.78025100
C	-3.56288600	0.01472600	0.72314500
C	-2.65468000	0.76313200	-0.06712900
H	-2.86580500	0.91755400	-1.11555100
C	-1.51450500	1.35473800	0.44435500
C	-0.70446300	2.25704700	-0.40960900
C	-1.37990600	3.13906400	-1.25674300
H	-2.46210600	3.15537800	-1.23970900
C	-0.70255700	4.00287000	-2.09806400
H	-1.25568200	4.67798900	-2.73835000
C	0.68354000	4.01011400	-2.09624000
H	1.23128900	4.69082800	-2.73522100
C	1.36738300	3.15387200	-1.25253600
H	2.44935900	3.18115300	-1.23184200
C	0.69869000	2.26504400	-0.40702100
C	1.51674900	1.37385300	0.45033900
C	2.66595800	0.79597900	-0.05763500
H	2.85972400	0.92449800	-1.11284100
C	3.57138600	0.04486800	0.73204100
C	3.25303300	-0.06566000	2.11250500
H	3.92092300	-0.57691300	2.79129400
C	2.09944800	0.47670900	2.62169900
H	1.87180500	0.35885700	3.67504300
C	1.20011900	1.17057000	1.80584100
C	-4.73544800	-0.60286900	0.15798000
C	-4.93733900	-0.69266300	-1.24743300
C	-5.75988700	-1.15894700	0.96903500
C	-6.05243000	-1.27815000	-1.78445500
H	-4.18847400	-0.31400400	-1.92887700
C	-6.88708700	-1.74386900	0.43302100
H	-5.68569400	-1.11241700	2.04615300

C	-7.04996400	-1.81330700	-0.95381200
H	-6.17924500	-1.34367400	-2.85761900
H	-7.63840000	-2.14236100	1.10007200
C	4.75384000	-0.56100200	0.17429400
C	5.20191900	-0.27263700	-1.14461900
C	5.54589700	-1.47993500	0.91241600
C	6.33069800	-0.84343700	-1.66836800
H	4.66184400	0.43306100	-1.76010900
C	6.68153300	-2.05977300	0.38802800
H	5.25148900	-1.77038400	1.91088400
C	7.09215100	-1.74872300	-0.91132500
H	6.65772200	-0.60077600	-2.67146300
H	7.23963200	-2.75990400	0.99341800
O	8.18298800	-2.25700200	-1.51564600
O	-8.10780000	-2.36680200	-1.57697800
C	-9.14291300	-2.92483600	-0.78913600
H	-9.60688900	-2.16664100	-0.15278800
H	-9.87990000	-3.31039600	-1.48847200
H	-8.76829200	-3.74243200	-0.16762500
C	8.98942600	-3.17395100	-0.79966900
H	9.80180800	-3.44698800	-1.46776400
H	9.39968300	-2.71602200	0.10423200
H	8.42363900	-4.06952900	-0.52969800

Optimized S_3 state coordinates of 6.

Energy = -1517.90933 Ha

N	-0.00004400	-1.49843900	2.51392000
H	-0.00007200	-1.26162400	3.49559500
C	1.20557800	-1.12640800	1.89644800
C	2.10876500	-0.32300800	2.59092800
H	1.87701200	-0.02652500	3.60722400
C	3.27512100	0.11077500	2.00379700
H	3.95452100	0.71564800	2.58789200
C	3.58791300	-0.23392100	0.67165900
C	2.67897200	-1.07626500	0.00837100
H	2.87507600	-1.36368700	-1.01513800
C	1.51840800	-1.55271700	0.59396500
C	0.70104800	-2.56737000	-0.11302700
C	1.37342000	-3.57446900	-0.80978800
H	2.45547200	-3.59451600	-0.78815600
C	0.69315100	-4.55658900	-1.50555600
H	1.24361000	-5.32662200	-2.03038000
C	-0.69292100	-4.55659100	-1.50562300
H	-1.24332700	-5.32662600	-2.03050100
C	-1.37325900	-3.57447700	-0.80991600
H	-2.45531300	-3.59452800	-0.78837900
C	-0.70095600	-2.56737600	-0.11309300
C	-1.51840300	-1.55278600	0.59388500
C	-2.67897000	-1.07639300	0.00825900
H	-2.87495600	-1.36369300	-1.01530700
C	-3.58802100	-0.23416900	0.67156300
C	-3.27542800	0.11029800	2.00381000
H	-3.95497200	0.71499700	2.58792100
C	-2.10909000	-0.32345400	2.59099000
H	-1.87746500	-0.02710300	3.60735400

C	-1.20571200	-1.12657600	1.89643900
C	-4.79103800	0.25682500	0.02315900
C	-5.48770500	1.37053500	0.53296200
C	-5.31894700	-0.36723600	-1.13758400
C	-6.64852300	1.86264500	-0.07031000
H	-5.10640500	1.90006300	1.39630400
C	-6.45445800	0.08427600	-1.73959500
H	-4.83325000	-1.24371400	-1.54448600
C	-7.34211600	2.99855200	0.43467400
C	-7.16250000	1.21720500	-1.23596400
H	-6.84282600	-0.42393700	-2.61417800
C	-8.48050400	3.45806700	-0.17996700
H	-6.95587700	3.49555900	1.31689100
C	-8.32497400	1.70927500	-1.83725500
C	-8.98036400	2.81477900	-1.32036300
H	-8.99674500	4.32308100	0.21720000
H	-8.71003100	1.21352300	-2.72076600
H	-9.87879300	3.18370400	-1.79842300
C	4.79103900	0.25691600	0.02332600
C	5.48781900	1.37054500	0.53312000
C	5.31902800	-0.36736800	-1.13726100
C	6.64864200	1.86258400	-0.07021800
H	5.10660300	1.90011700	1.39647100
C	6.45451300	0.08409600	-1.73935300
H	4.83336000	-1.24392400	-1.54403000
C	7.34231400	2.99846300	0.43471600
C	7.16250400	1.21713400	-1.23591200
H	6.84290000	-0.42424900	-2.61385100
C	8.48060500	3.45800600	-0.18007800
H	6.95621100	3.49543100	1.31701500
C	8.32483900	1.70928600	-1.83741600
C	8.98026000	2.81480600	-1.32061900
H	8.99691400	4.32299600	0.21705300
H	8.70977700	1.21355200	-2.72098800
H	9.87858800	3.18378500	-1.79882600

Optimized S_2 state coordinates of 7.

Energy = -1395.11211 Ha

N	0.01101800	-1.42839000	1.91330800
H	0.02333200	-2.29970800	2.42380300
C	-1.25232800	-1.10659100	1.41320300
C	-2.19343900	-2.13766300	1.40866800
H	-1.91942200	-3.10764400	1.80460700
C	-3.44678800	-1.94653700	0.87517900
H	-4.14720100	-2.77095800	0.85818800
C	-3.78371800	-0.71815400	0.30385400
C	-2.85029700	0.30686100	0.34738400
H	-3.11230600	1.26898600	-0.07089500
C	-1.58997600	0.16498300	0.93678000
C	-0.73875400	1.35033900	1.11916700
C	-1.38328600	2.56253700	1.38433200
H	-2.45604700	2.56221100	1.52467100
C	-0.70020900	3.75609700	1.49655700
H	-1.23610000	4.67033400	1.71468400
C	0.68498800	3.76091200	1.33602700

H	1.24459000	4.68296300	1.42902700
C	1.34725700	2.58425500	1.08735200
H	2.42564000	2.59589100	1.00956400
C	0.67772500	1.34051000	0.99287200
C	1.50555800	0.16506000	0.78852900
C	2.74970000	0.27601800	0.13435200
H	2.91666300	1.14564900	-0.48030600
C	3.78017200	-0.69144200	0.27469200
C	3.48601800	-1.84116500	1.05354200
H	4.24166500	-2.59634200	1.21483100
C	2.26285000	-2.02390200	1.62144000
H	2.04898500	-2.92371000	2.18370700
C	1.22292100	-1.06825800	1.43159700
C	-5.10264400	-0.51241700	-0.33294400
C	-5.21880200	0.24335400	-1.50044300
C	-6.25492800	-1.07133100	0.22157600
C	-6.44713900	0.43903800	-2.10024100
H	-4.33521600	0.66763500	-1.95873200
C	-7.48955000	-0.88279200	-0.36749000
H	-6.19042600	-1.64469100	1.13692000
C	-7.58827100	-0.12510600	-1.53305300
H	-6.52413600	1.02022100	-3.00899200
H	-8.37703300	-1.31388200	0.07525600
C	5.08532800	-0.51415300	-0.29525400
C	5.49874000	0.75846100	-0.78741700
C	6.01138400	-1.58356800	-0.43753200
C	6.73310800	0.94378800	-1.35433300
H	4.84804500	1.61475900	-0.68486600
C	7.24058600	-1.40395200	-1.01072700
H	5.74413400	-2.58107500	-0.11840900
C	7.62855300	-0.13027500	-1.48160700
H	7.02404500	1.92609700	-1.70373400
H	7.91767300	-2.24177400	-1.11188600
C	-8.86476000	0.07347600	-2.14935600
N	-9.88989300	0.23284600	-2.64446300
C	8.89922300	0.06139300	-2.07674500
N	9.93712900	0.21645300	-2.55987300

Optimized S_3 state coordinates of 8.

Energy = -1884.83221 Ha

N	-0.00786300	-2.20060100	1.54248600
H	-0.02662000	-2.66572500	2.43872400
C	1.25656200	-1.73177100	1.19736000
C	2.20830800	-1.69357500	2.22052100
H	1.94487400	-2.06087800	3.20466600
C	3.45407500	-1.15537600	2.00227200
H	4.16029100	-1.10938700	2.82077700
C	3.77977100	-0.61488400	0.75628100
C	2.83817900	-0.70100700	-0.26081400
H	3.09150700	-0.31988500	-1.24076400
C	1.58672300	-1.29408400	-0.08937800
C	0.73867800	-1.54843800	-1.27022900
C	1.39114900	-1.89533800	-2.45450700
H	2.46406100	-2.03318900	-2.43835900
C	0.71177600	-2.08571000	-3.64372400

H	1.25234400	-2.36004300	-4.53993200
C	-0.66878800	-1.92425400	-3.66505200
H	-1.22420900	-2.07091000	-4.58272100
C	-1.33810600	-1.59919400	-2.50720000
H	-2.41587700	-1.51713800	-2.52824300
C	-0.67242600	-1.42589800	-1.27387300
C	-1.50441000	-1.15405800	-0.10579400
C	-2.74533000	-0.50896500	-0.26089300
H	-2.91462900	0.04243700	-1.17089100
C	-3.77169600	-0.56900800	0.73307700
C	-3.47228300	-1.27313400	1.92578400
H	-4.22058300	-1.37088600	2.69946100
C	-2.25589000	-1.85034500	2.13532500
H	-2.03921100	-2.35510300	3.06784000
C	-1.21878500	-1.72920700	1.15570300
C	5.09139700	0.02574500	0.52184700
C	5.19965200	1.15559500	-0.28660100
C	6.25139500	-0.48803600	1.10591900
C	6.42658600	1.75773200	-0.50951100
H	4.31334200	1.58667500	-0.73367100
C	7.47774800	0.10778800	0.88722200
H	6.19881100	-1.37669200	1.72126600
C	7.56724300	1.23350000	0.07683500
H	6.48674300	2.63750200	-1.13471000
H	8.36693200	-0.30990100	1.34177000
C	-5.06912300	0.00470700	0.52397300
C	-5.46562400	0.46415200	-0.76386800
C	-6.01181500	0.17745100	1.57357000
C	-6.70560400	1.02831100	-0.97788800
H	-4.80598700	0.34261000	-1.61037400
C	-7.23965100	0.74458900	1.35511100
H	-5.76251100	-0.11587800	2.58358200
C	-7.60648500	1.17726900	0.06966200
H	-6.97507000	1.35273800	-1.97424100
H	-7.92665900	0.86384000	2.18364300
C	8.91270200	1.86559600	-0.13541600
C	-8.94454300	1.80135800	-0.12965500
F	9.80852400	0.98588800	-0.61845100
F	8.87542600	2.89288800	-0.99190000
F	9.43069700	2.33589000	1.01475200
F	-9.95210300	1.00096400	0.28364900
F	-9.19795800	2.10610700	-1.41012200
F	-9.08912100	2.94881400	0.57300600

Additional data: HOMO and LUMO energies and MOs primarily involved in the formation of the lowest excited states. Calculation of CT, Δr and ISC details.

PC	IP (eV)	EA (eV)	Gap (HOMO-LUMO) (eV)
**4**	5.93	1.29	4.64
**5**	5.85	1.26	4.59
**6**	5.92	1.30	4.61
**7**	6.03	1.37	4.66
**8**	6.00	1.34	4.65

Table S13. HOMO-LUMO energy gaps.

PC	Excitation Energy (eV)	Oscillator strength	Corresponding excited state	Charge transfer number	Δr (Å)	$E_{0,0}$ Calculated (eV)
**4**	4.86	0.6461	S_3	0.26	3.60	3.30
**5**	4.83	0.9716	S_3	0.28	3.64	3.29
**6**	4.55	0.1529	S_3	0.18	3.55	3.29
**7**	4.59	1.3475	S_2	0.33	4.17	3.31
**8**	4.76	0.9078	S_3	0.31	3.91	---[a]

Table S14. Analysis of the excited states, calculation of charge transfer number, Δr, and $E_{0,0}$

[a] *Not determined due to the presence of an imaginary frequency*

	PC 4 (S_3)	PC 5 (S_3)	PC 6 (S_3)	PC 7 (S_2)	PC 8 (S_3)
T_1	2.8316	2.9342	2.7305	2.5489	2.6308
T_2	3.3421	2.9703	2.7458	2.883	2.9686
T_3	3.5629	3.5247	3.3345	3.3688	3.4458

Table S15. Calculation of E_T value and excited states involved in the ISC process.

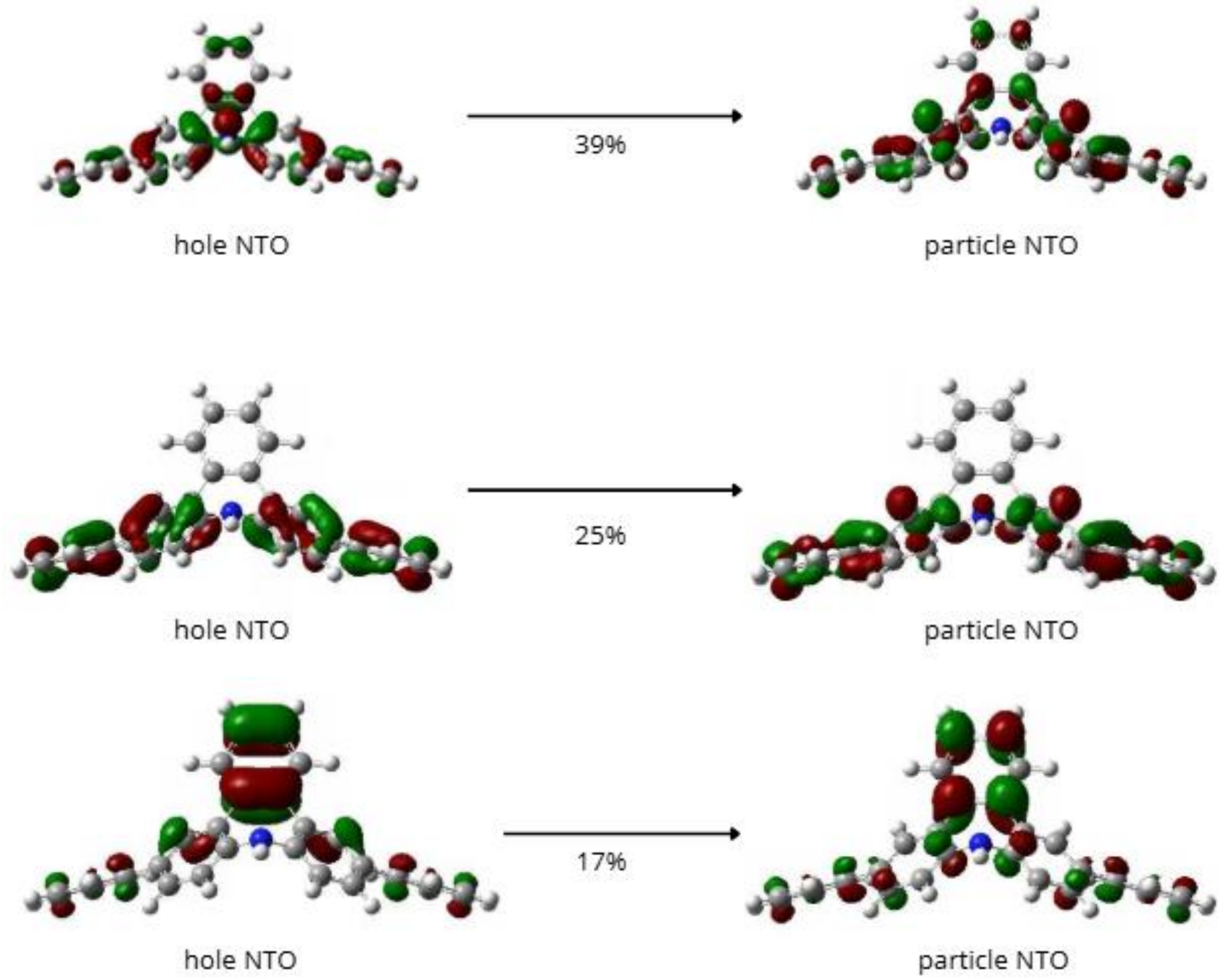

Figure S57: Natural Transition Orbitals of **4** of S_3 (isoval = 0.04 a. u.)

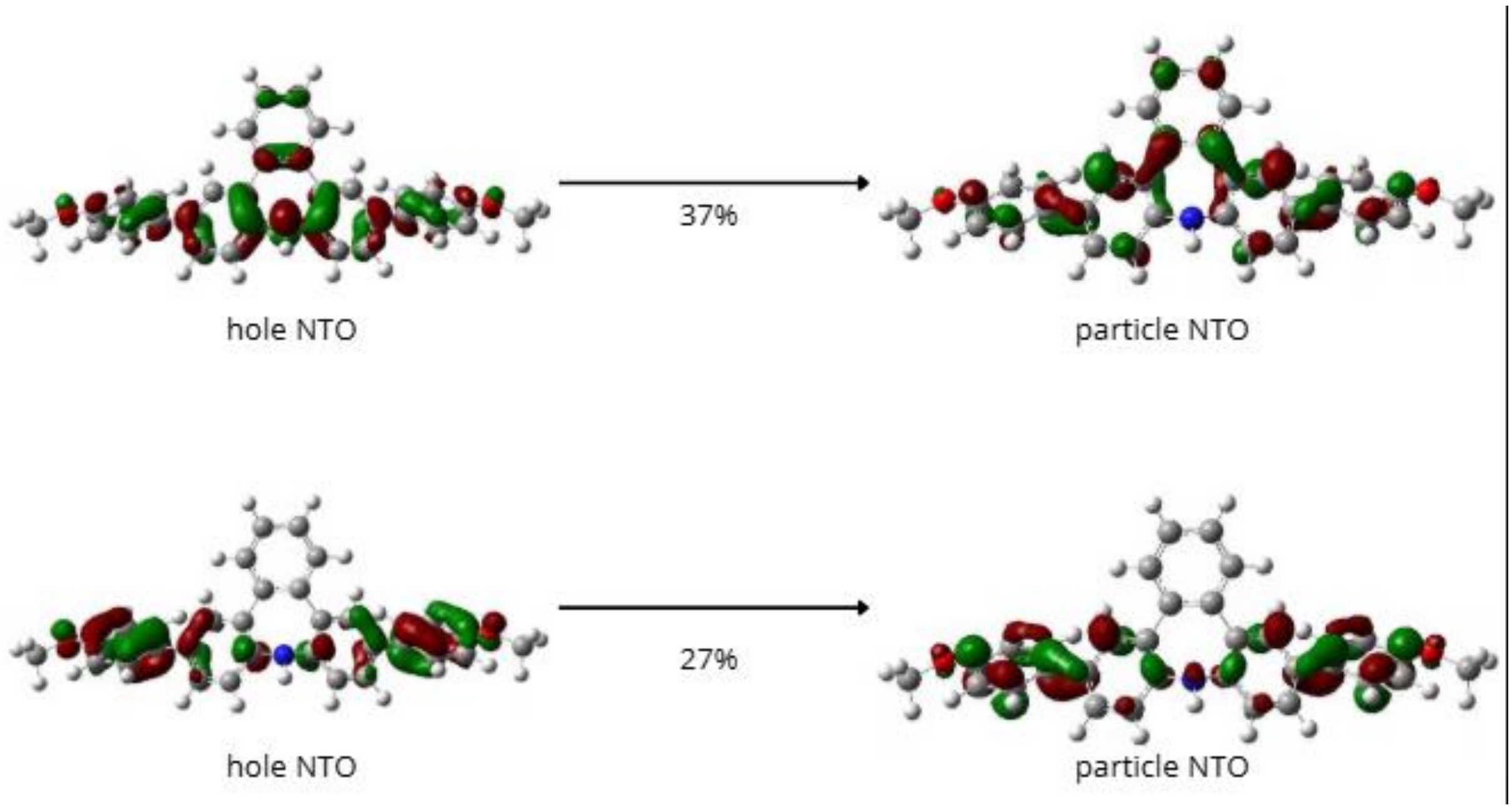

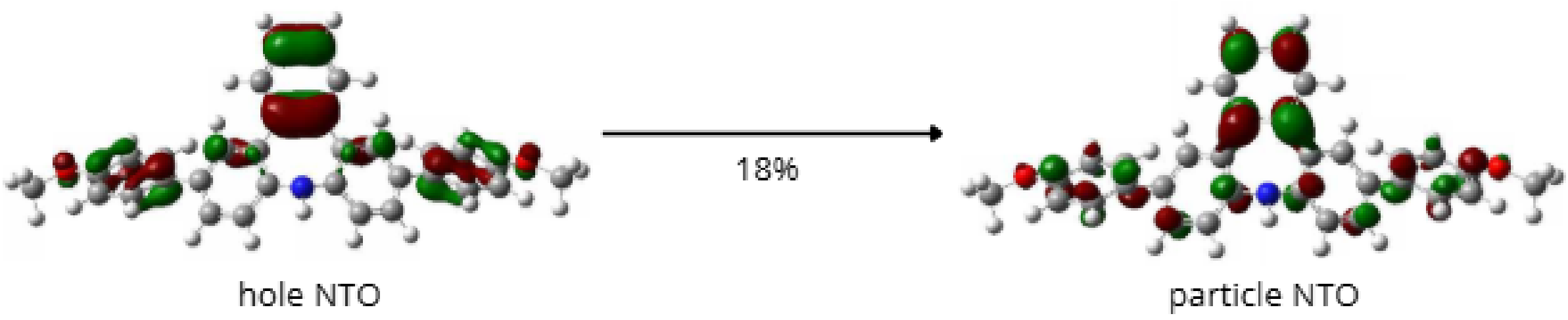

Figure S58: Natural Transition Orbitals of **5** of S_3 (isoval = 0.04 a. u.)

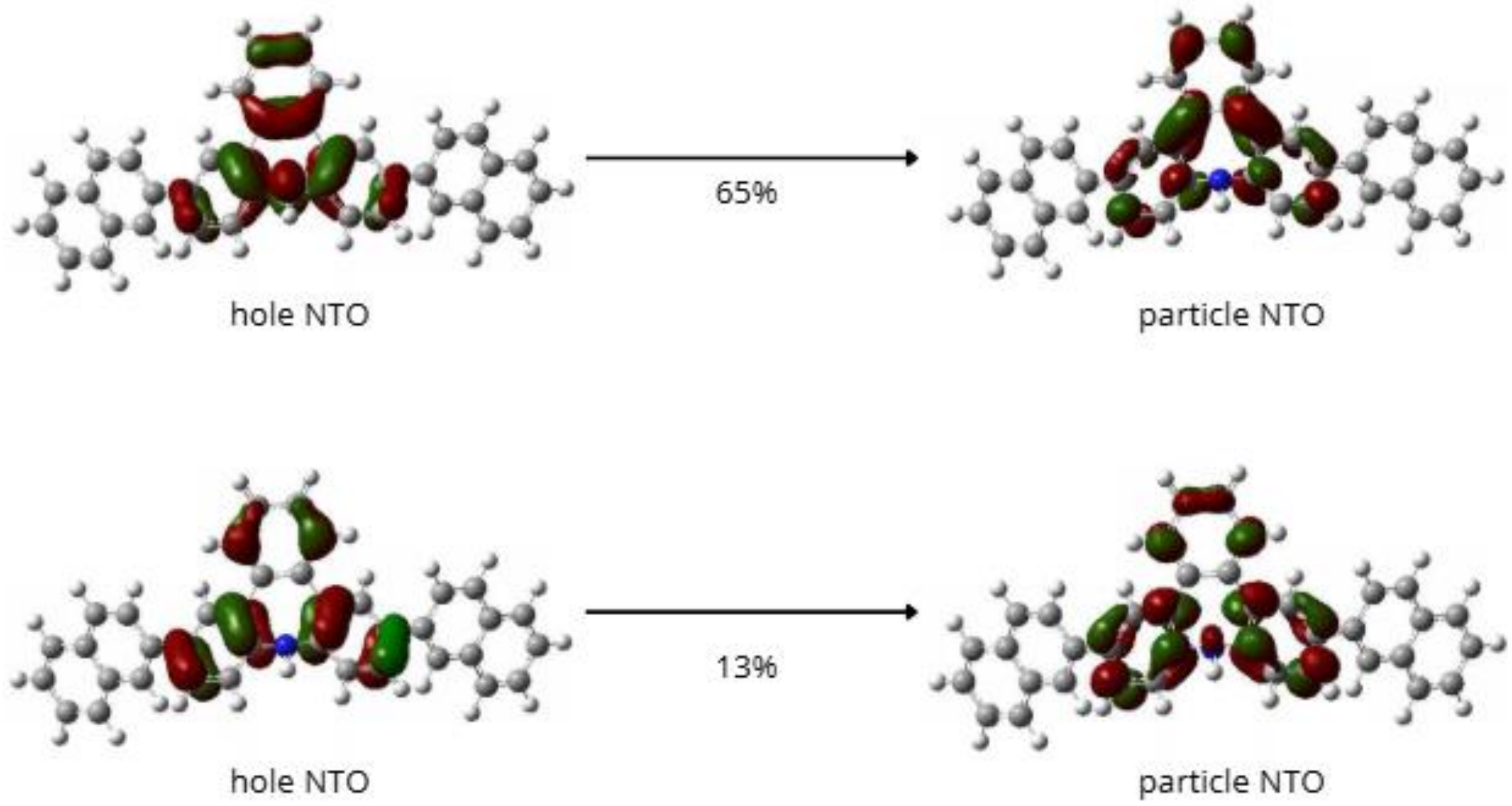

Figure S59: Natural Transition Orbitals of **6** of S_3 (isoval = 0.04 a. u.)

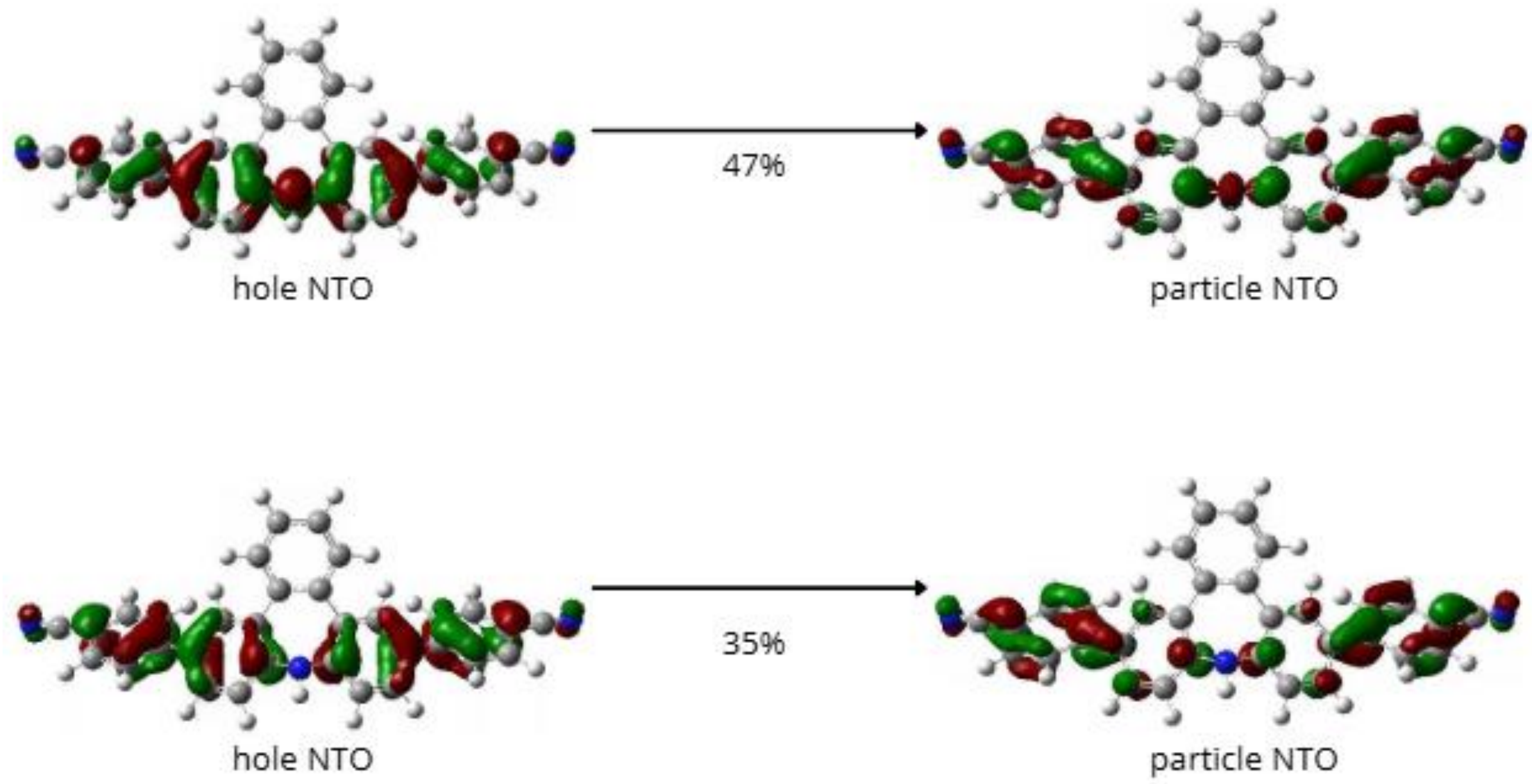

Figure S60: Natural Transition Orbitals of **7** of S_2 (isoval = 0.04 a. u.)

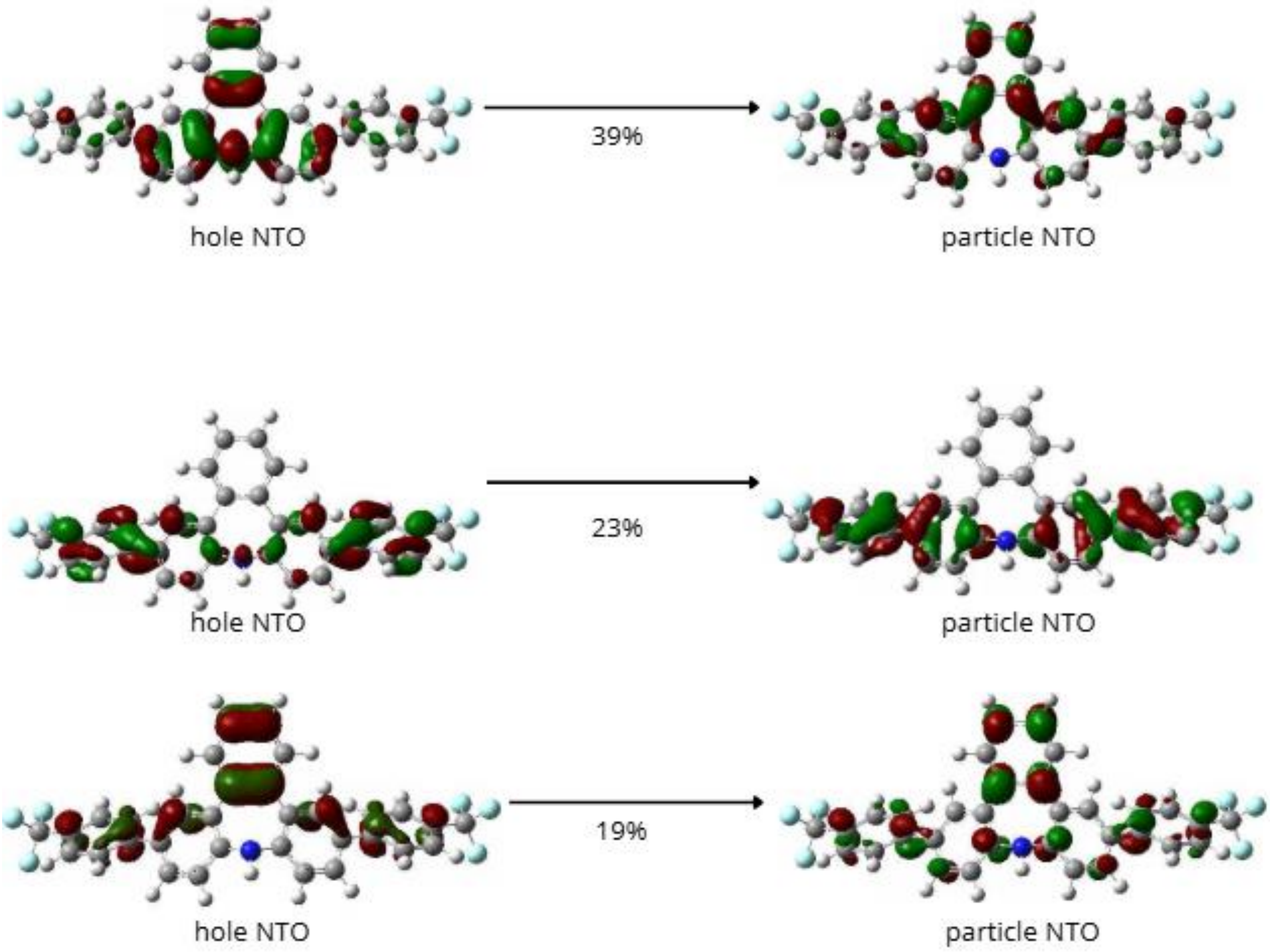

Figure S61: Natural Transition Orbitals of **8** of S_3 (isoval = 0.04 a. u.)

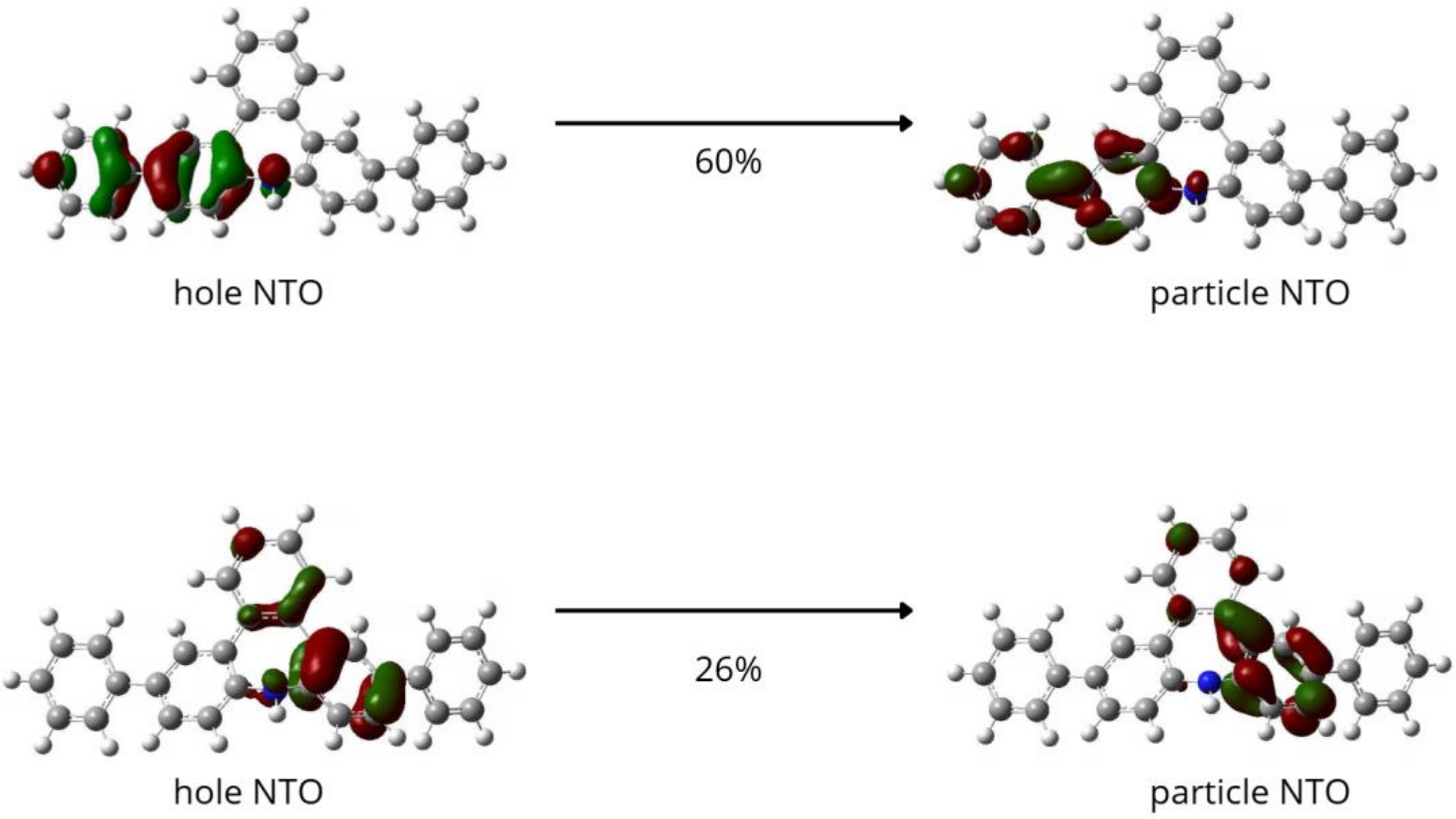

Figure S62: Natural Transition Orbitals of **4** of T_2 (isoval = 0.04 a. u.)

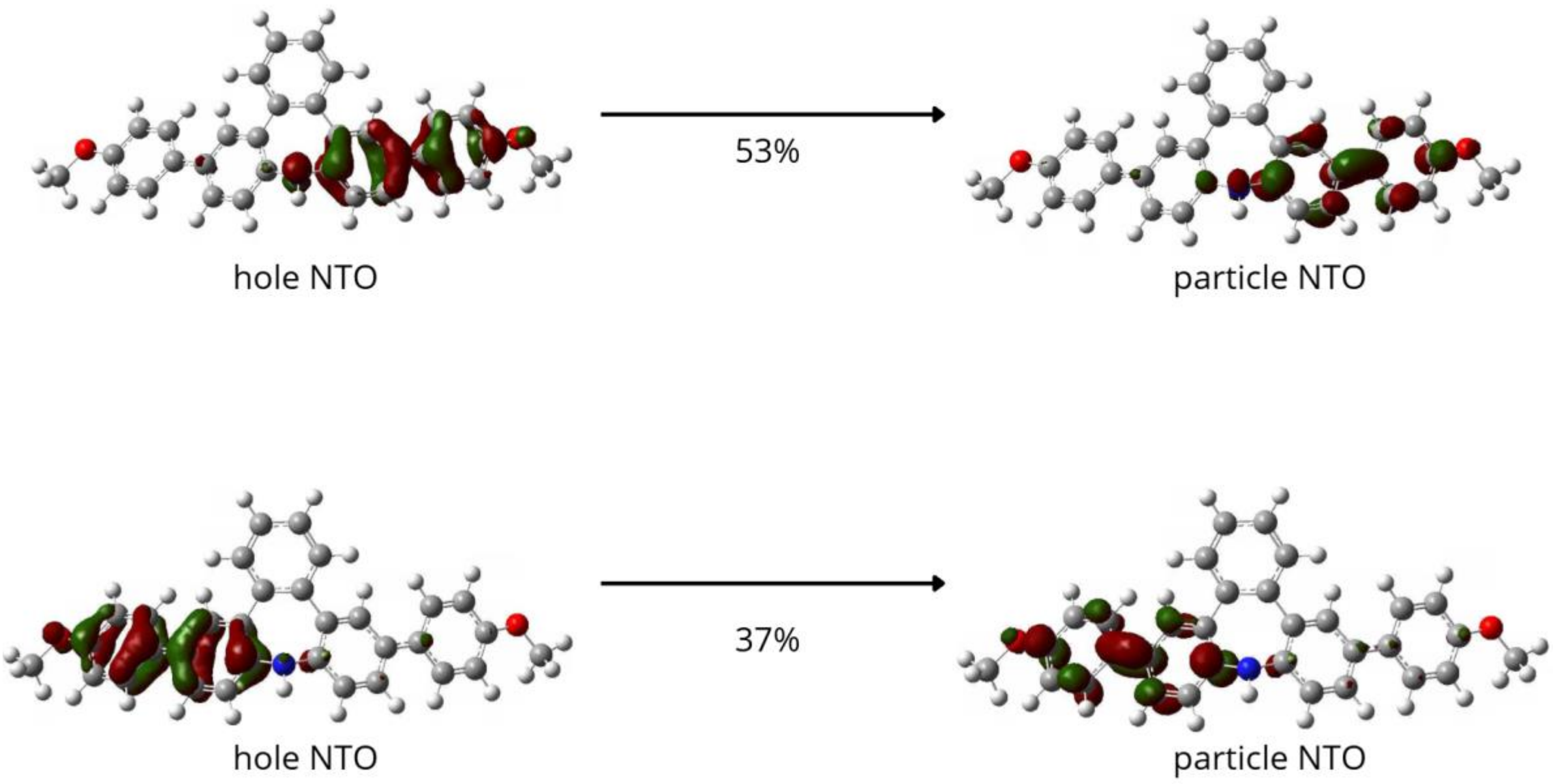

Figure S63: Natural Transition Orbitals of **5** of T_2 (isoval = 0.04 a. u.)

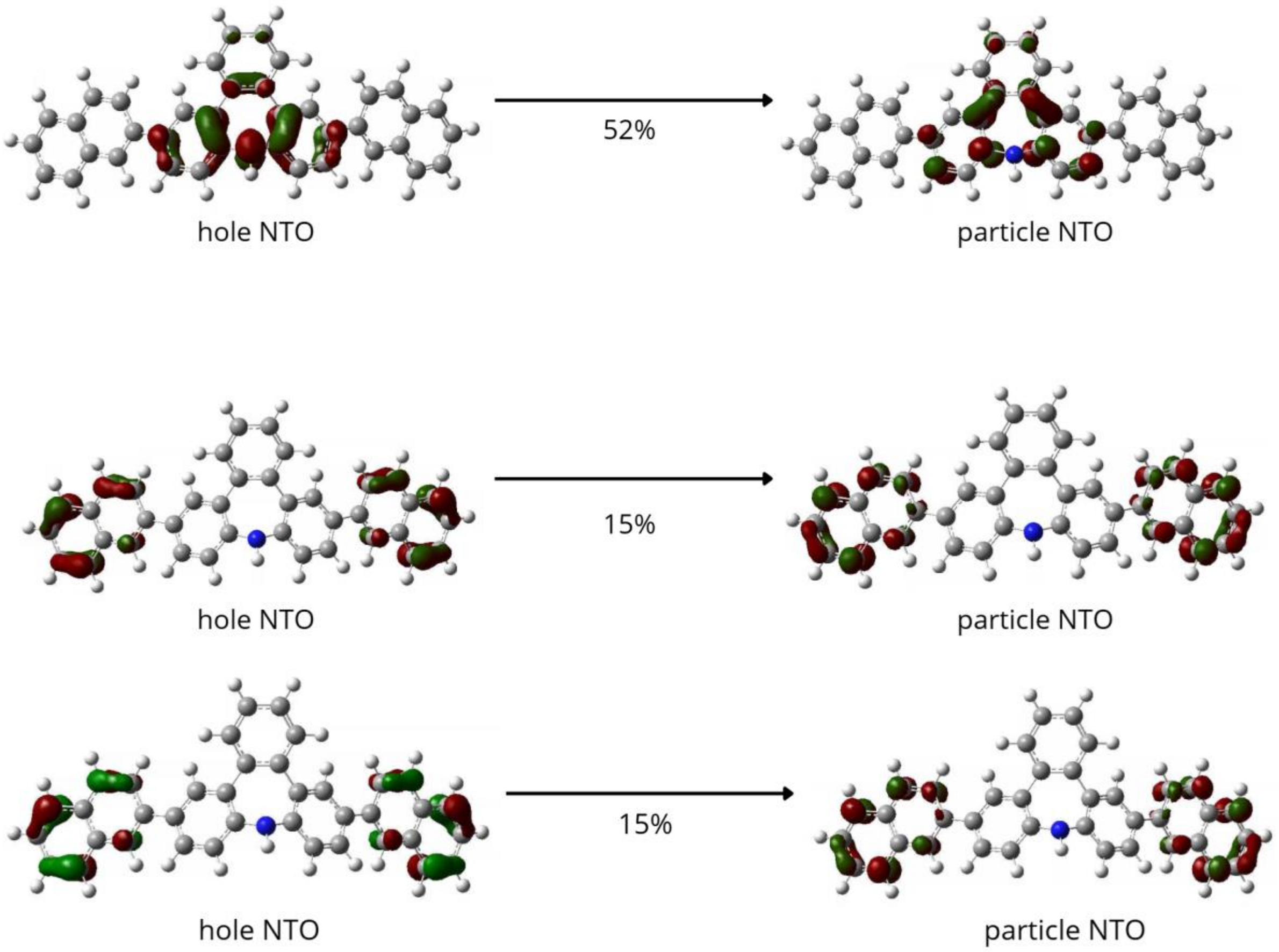

Figure S64: Natural Transition Orbitals of **6** of T_3 (isoval = 0.04 a. u.)

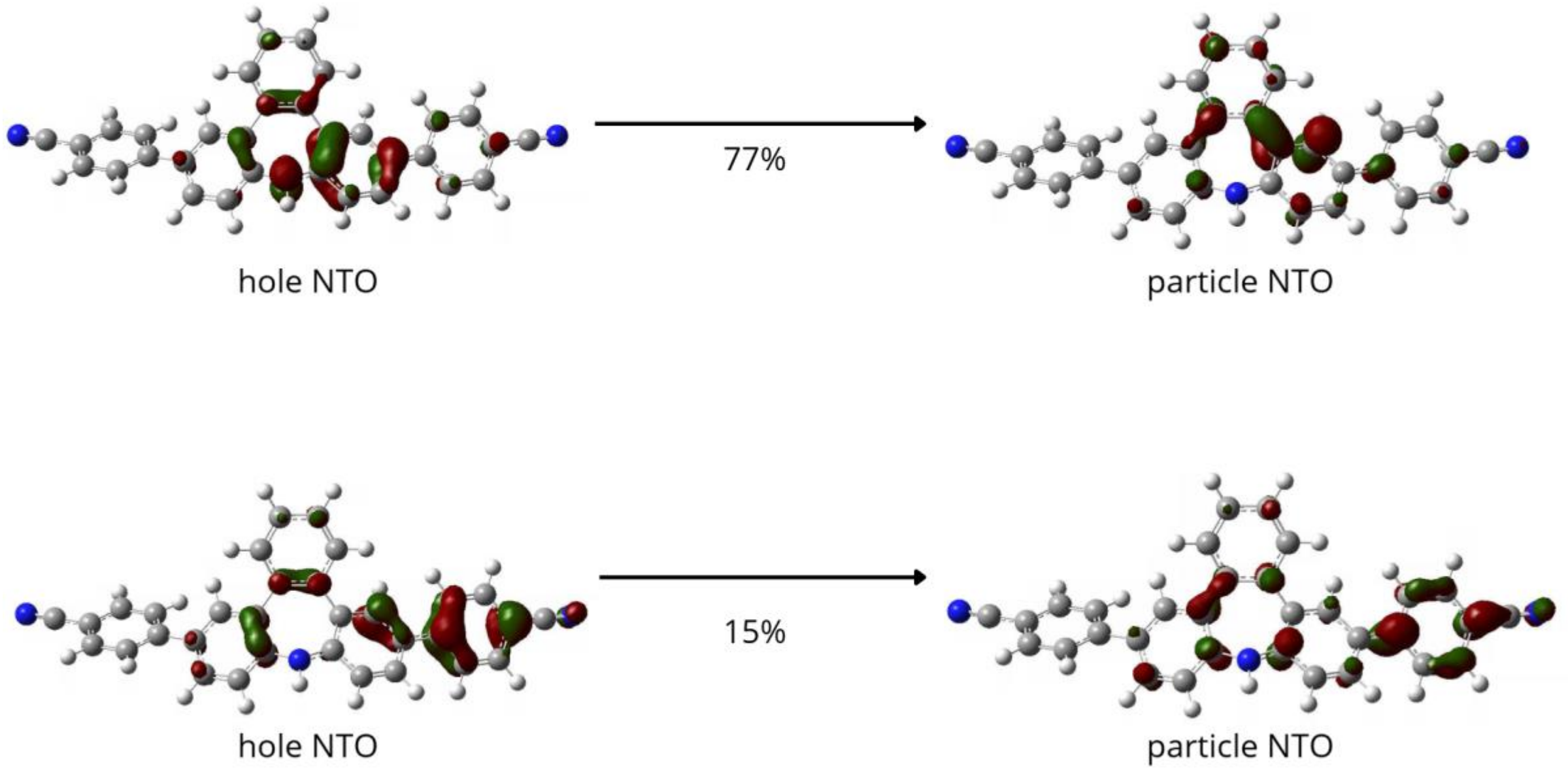

Figure S65: Natural Transition Orbitals of **7** of T_2 (isoval = 0.04 a. u.)

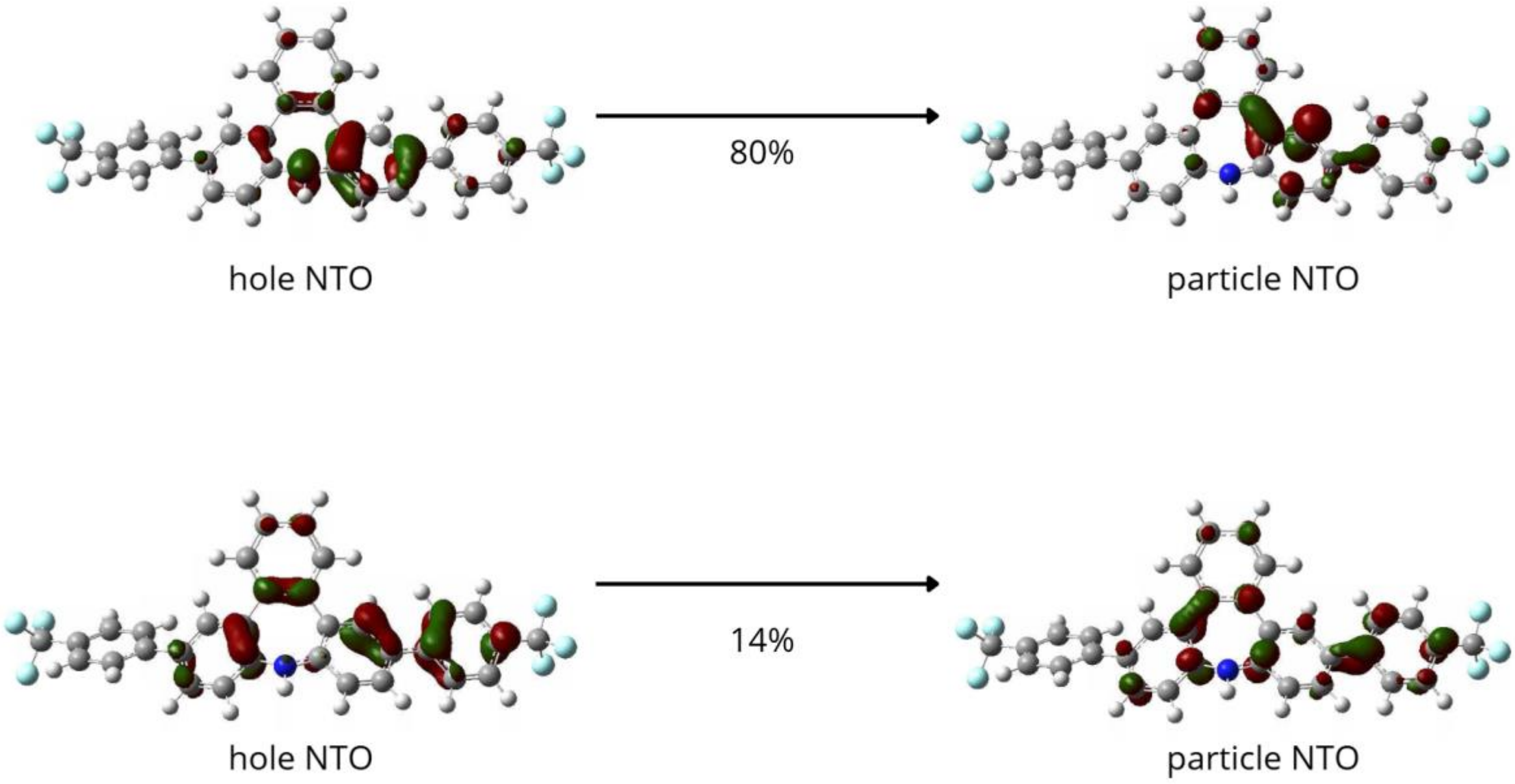

Figure S66: Natural Transition Orbitals of **8** of T_2 (isoval = 0.04 a. u.)

I. References

1. Fang, M. Y., Chen, L. P., Huang, L., Fang, D. M., Chen, X. Z., Wang, B. Q., Feng, C., Xiang, S. K. Synthesis of tribenzo[*b*,*d*,*f*]azepines via palladium-catalyzed annulation reaction of 2-iodobiphenyls with 2-halogenoanilines. *J. Org. Chem.*, **2021**, *86*, 9096–9106.
2. Kim, C. S., Eum, S. J., Cho, Y. J., Kwon, H. J., Kim, B. O., Kim, S. M., Yoo, S. S. Novel organic electroluminescent compounds and organic electroluminescent device using the same. WO2011/10839, 2011, A1
3. Würth, C., Grabolle, M., Pauli, J., Spieles, M., Resch-Genger, U. Relative and absolute determination of fluorescence quantum yields of transparent samples. *Nature Protocols*, **2013**, *8*, 1535–1550.
4. Romero, N. A., Nicewicz, D. A. Organic photoredox catalysis. *Chem. Rev.* **2016**, *116*, 10075–10166.
5. Wallace, A. M., Curiac, C., Delcamp, J. H., Fortenberry, R. C. Accurate determination of the onset wavelength (λonset) in optical spectroscopy. *J. of Quant. Spectrosc. and Radiat. Transf.*, **2021**, *265*, 107544.
6. *SAINT*, **2025**, *V8.42*, Bruker AXS SE.
7. L. Krause, R. Herbst-Irmer, G. M. Sheldrick, D. Stalke, *J. Appl. Cryst.* **2015**, *48*, 3–10.
8. G. M. Sheldrick, *Acta Cryst.* **2015**, *A71*, 3–8.
9. G. M. Sheldrick, *Acta Cryst.* **2015**, *C71*, 3–8.
10. C. R. Groom, I. J. Bruno, M. P. Lightfoot, S. C. Ward, *Acta Cryst.* **2016**, *B72*, 171–179.
11. Bortolato, T., Simionato, G., Vayer, M., Rosso, C., Paoloni, L., Benetti, E. M., Sartorel, A., Lebœuf, D., Dell'Amico, L. The Rational Design of Reducing Organophotoredox Catalysts Unlocks Proton-Coupled Electron-Transfer and Atom Transfer Radical Polymerization Mechanisms. *J. Am. Chem. Soc.*, **2023**, *145*, 1835–1846.
12. (a) Mateos, J., Rigodanza, F., Vega-Peñaloza, A., Sartorel, A., Natali, M., Bortolato, T., Pelosi, G., Companyó, X., Bonchio, M., Dell'Amico, L. Naphthochromenones: Organic Bimodal Photocatalysts Engaging in Both Oxidative and Reductive Quenching Processes. *Angew. Chem. Int. Ed.*, **2019**, *59*, 1302–1312. (b) Bryden, M. A., Millward, F., Matulaitis, T., Chen, D., Villa, M., Fermi, A., Cetin, S., Ceroni, P., Zysman-Colman, E. Moving Beyond Cyanoarene Thermally Activated Delayed Fluorescence Compounds as Photocatalysts: An Assessment of the Performance of a Pyrimidyl Sulfone Photocatalyst in Comparison to 4CzIPN. *J. Org. Chem.*, **2023**, *88*, 6364–6373.
13. Vijayakrishnan, S., Ward, J. W., Cooper, A. I. Discovery of a Covalent Triazine Framework Photocatalyst for Visible-Light-Driven Chemical Synthesis using High-Throughput Screening. *ACS Catalysis*, **2022**, *12*, 10057–10064.
14. Frisch, M. J.; Trucks, G. W.; Schlegel, H. B.; Scuseria, G. E.; Robb, M. A.; Cheeseman, J. R.; Scalmani, G.; Barone, V.; Petersson, G. A.; Nakatsuji, H.; Li, X.; Caricato, M.; Marenich, A. V.; Bloino, J.; Janesko, B. G.; Gomperts, R.; Mennucci, B.; Hratchian, H. P.; Ortiz, J. V.; Izmaylov, A. F.; Sonnenberg, J. L.; Williams-Young, D.; Ding, F.; Lipparini, F.; Egidi, F.; Goings, J.; Peng, B.; Petrone, A.; Henderson, T.; Ranasinghe, D.; Zakrzewski, V. G.; Gao, J.; Rega, N.; Zheng, G.; Liang, W.; Hada, M.; Ehara, M.; Toyota, K.; Fukuda, R.; Hasegawa, J.; Ishida, M.; Nakajima, T.; Honda, Y.; Kitao, O.; Nakai, H.; Vreven,

T.; Throssell, K.; Montgomery, J. A., Jr.; Peralta, J. E.; Ogliaro, F.; Bearpark, M. J.; Heyd, J. J.; Brothers, E. N.; Kudin, K. N.; Staroverov, V. N.; Keith, T. A.; Kobayashi, R.; Normand, J.; Raghavachari, K.; Rendell, A. P.; Burant, J. C.; Iyengar, S. S.; Tomasi, J.; Cossi, M.; Millam, J. M.; Klene, M.; Adamo, C.; Cammi, R.; Ochterski, J. W.; Martin, R. L.; Morokuma, K.; Farkas, O.; Foresman, J. B.; Fox, D. J. *Gaussian 16*, Revision B.01, Gaussian, Inc., Wallingford CT, **2016**.
15. Baerends, E. J., Gritsenko, O. v., van Meer, R.. The Kohn-Sham gap, the fundamental gap and the optical gap: The physical meaning of occupied and virtual Kohn-Sham orbital energies. *Physical Chemistry Chemical Physics,* **2013**, *15*, 16408–16425.
16. Santoro, F., Lami, A., Improta, R., Bloino, J., Barone, V. Effective method for the computation of optical spectra of large molecules at finite temperature including the Duschinsky and Herzberg-Teller effect: The Qx band of porphyrin as a case study. *Journal of Chemical Physics*, **2008**, *128*.
17. Guido, C. A.; Cortona, P.; Mennucci, B.; Adamo, C. On the metric of charge transfer molecular excitations: a simple chemical descriptor. *J. Chem. Theory Comput.* **2013**, 9, 3118−3126.
18. Iozzi, M. F., Mennucci, B., Tomasi, J., Cammi, R. Excitation energy transfer (EET) between molecules in condensed matter: A novel application of the polarizable continuum model (PCM). *J. Chem. Phys.*, **2004**, *120*, 7029–7040.
19. Gagliardi, L., Truhlar, D. G., Manni, G. L., Carlson, R. K., Hoyer, C. E., Bao, J. L. Multiconfiguration pair-density functional theory: A new way to treat strongly correlated systems. *Acc. Chem. Res.*, **2017**, *50*, 66–73.
20. Aquilante, F., Lindh, R., Bondo Pedersen, T. Unbiased auxiliary basis sets for accurate two-electron integral approximations. *J. Chem. Phys.*, **2007**, *127*, 114107
21. Widmark, P. O., Zobel, J. P., Vysotskiy, V. P., Tsuchiya, T., Veryazov, V. New compact density matrix averaged ANO basis sets for relativistic calculations. *J. Chem. Phys.*, **2018**, *149*, 194102.
22. Penfold, T. J., Gindensperger, E., Daniel, C., Marian, C. M. Spin-Vibronic Mechanism for Intersystem Crossing. *Chem. Rev.,* **2018**, *118*, 6975–7025.

J. NMR spectra

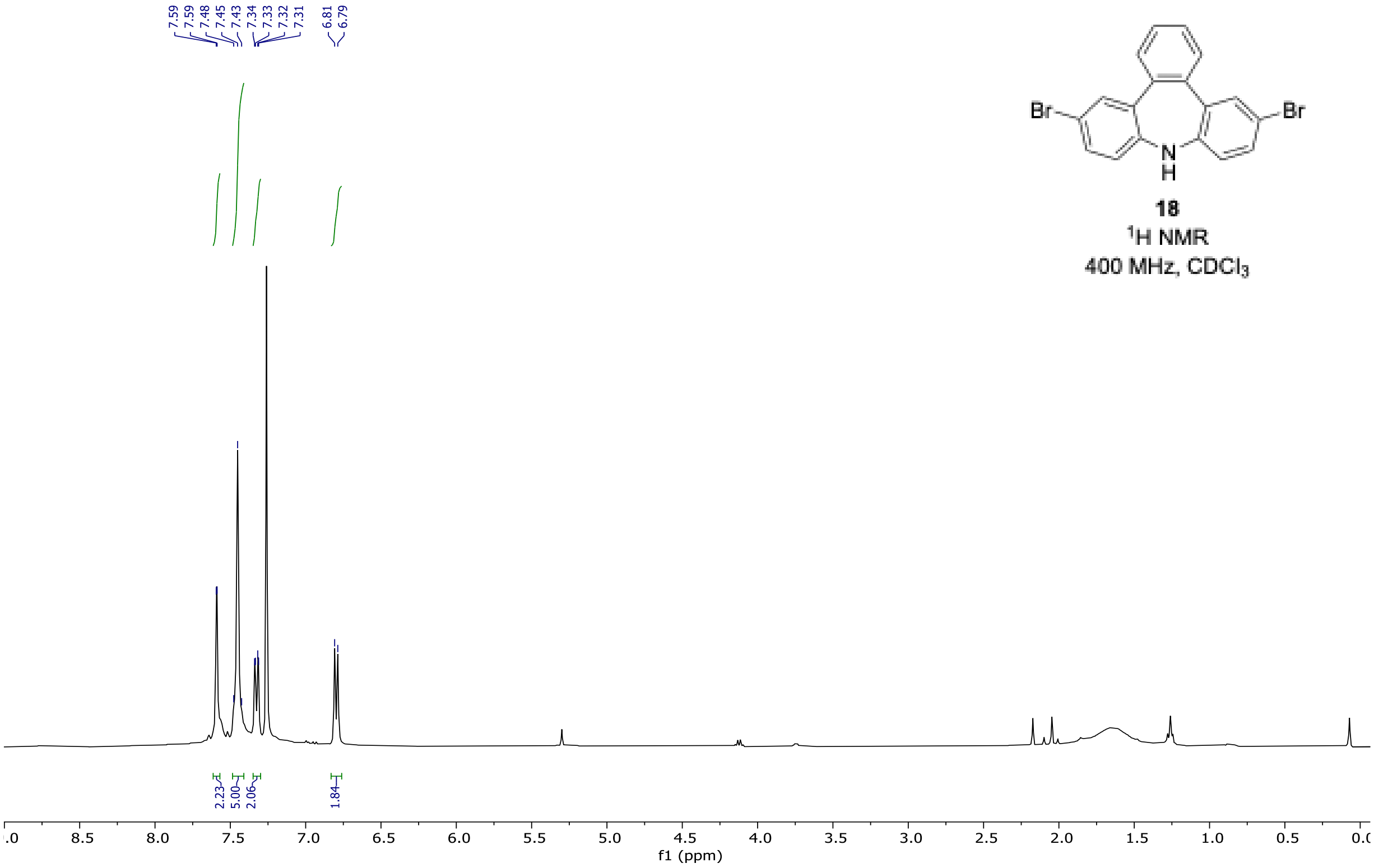

18
^1H NMR
400 MHz, $CDCl_3$

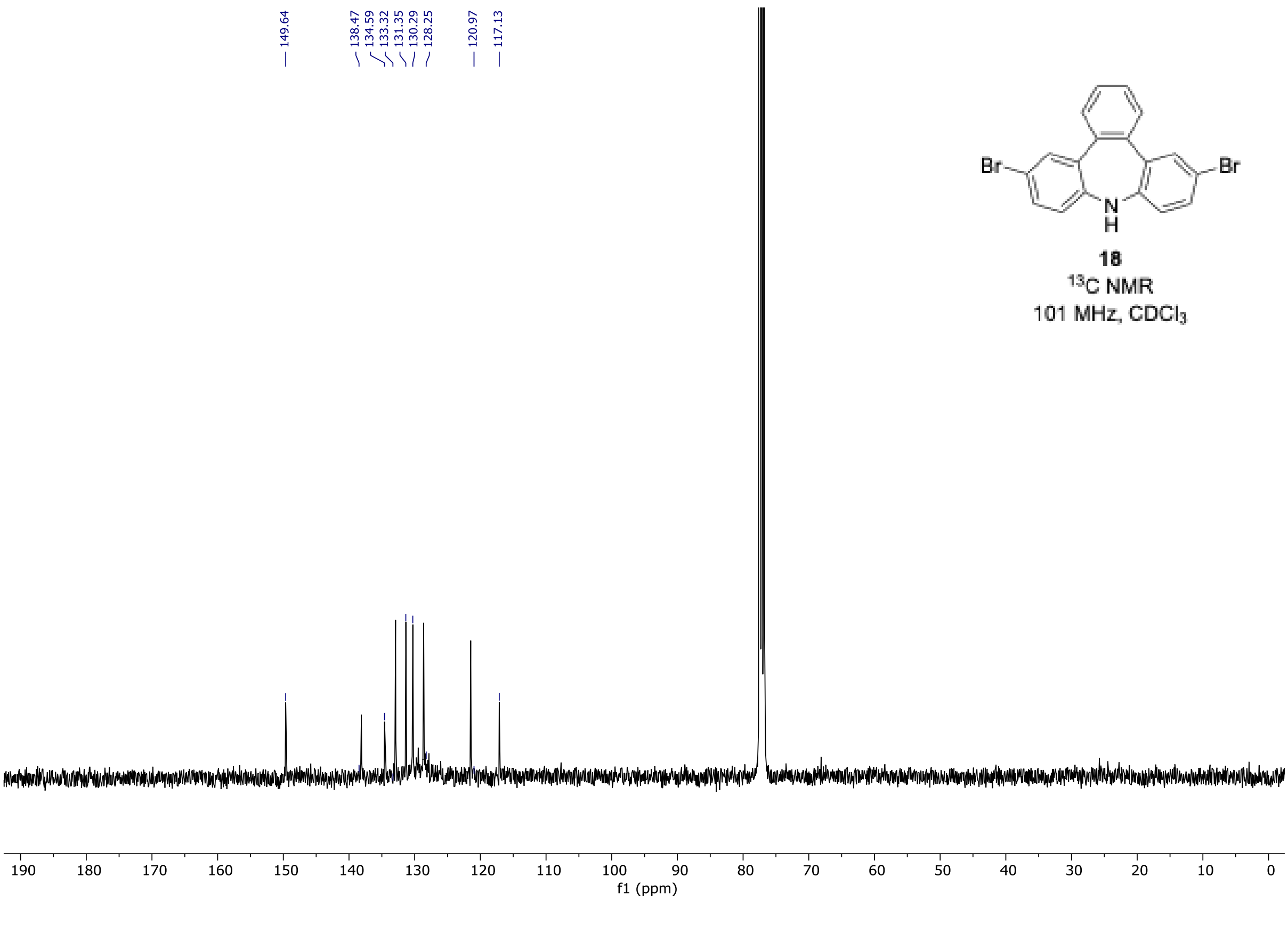

18
^{13}C NMR
101 MHz, $CDCl_3$

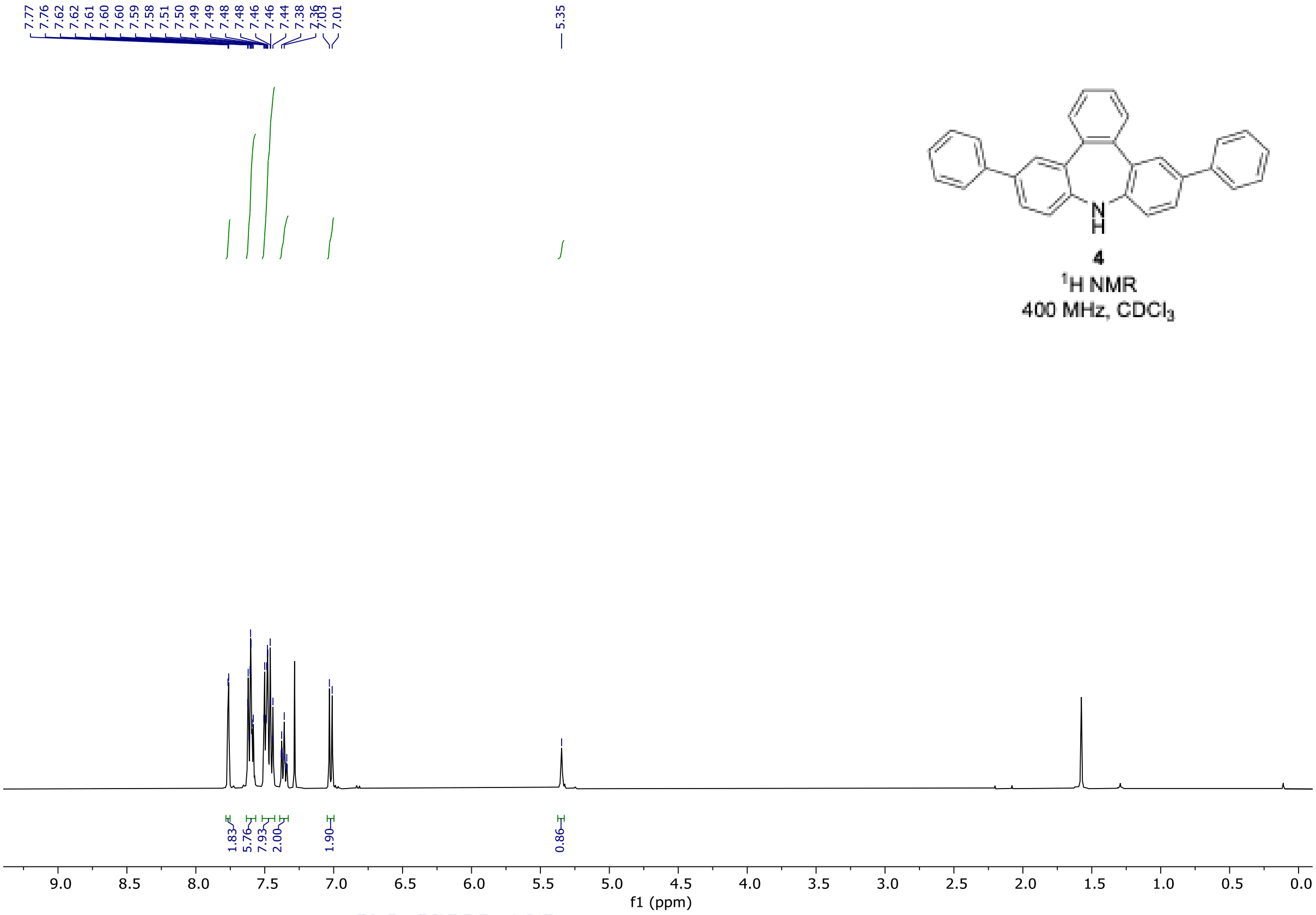
7.77
7.76
7.62
7.61
7.60
7.59
7.58
7.51
7.50
7.49
7.49
7.48
7.48
7.46
7.46
7.44
7.38
7.36
7.03
7.01
5.35
4
^{1}H NMR
400 MHz, CDCl$_3$
1.83
5.76
7.93
2.00
1.90
0.86
9.0
8.5
8.0
7.5
7.0
6.5
6.0
5.5
5.0
4.5
4.0
3.5
3.0
2.5
2.0
1.5
1.0
0.5
0.0
f1 (ppm)

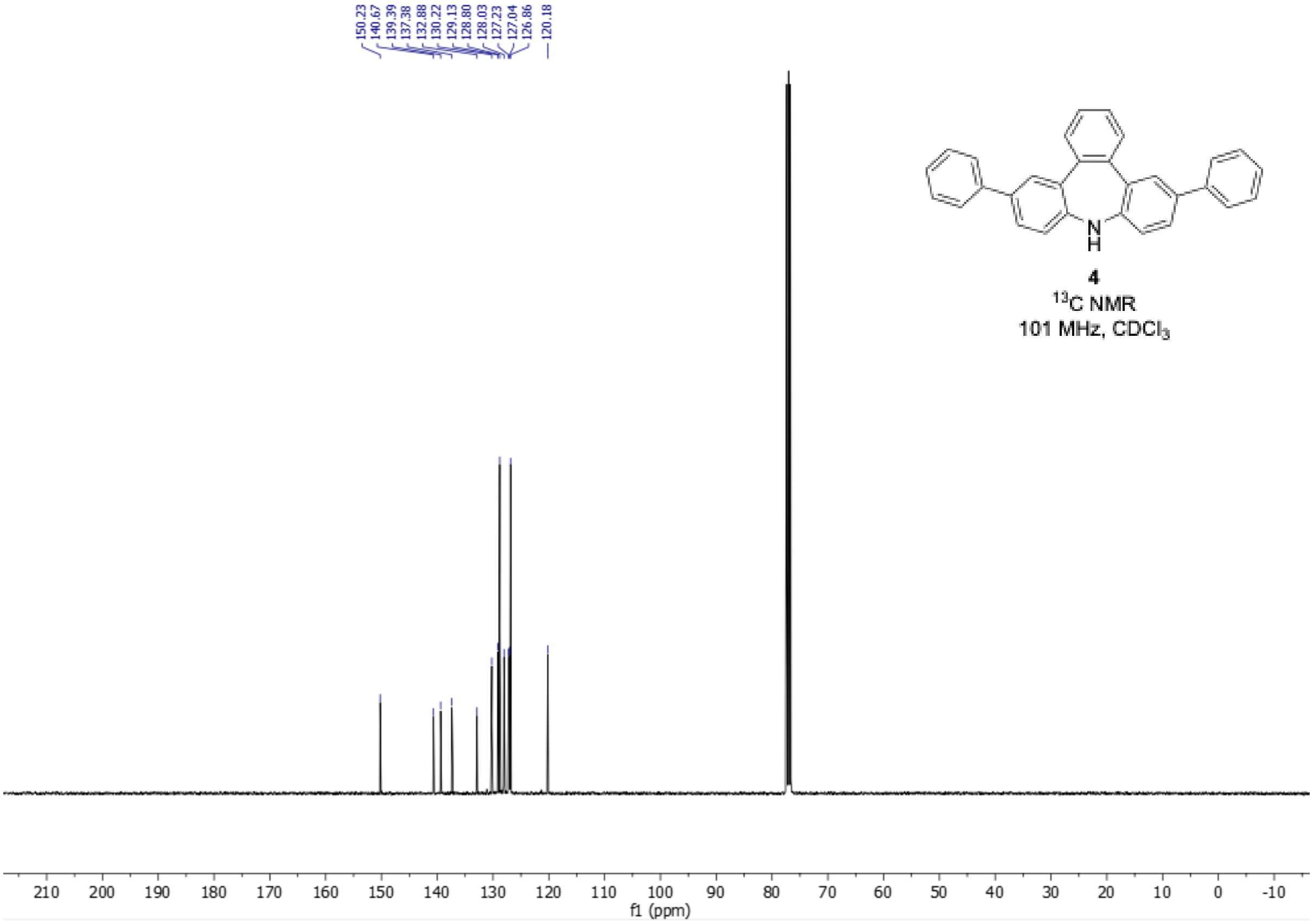
150.23
140.67
139.39
137.38
132.88
130.22
129.13
128.80
128.03
127.23
127.04
126.86
120.18
4
^{13}C NMR
101 MHz, CDCl$_3$
210
200
190
180
170
160
150
140
130
120
110
100
90
80
70
60
50
40
30
20
10
0
-10
f1 (ppm)

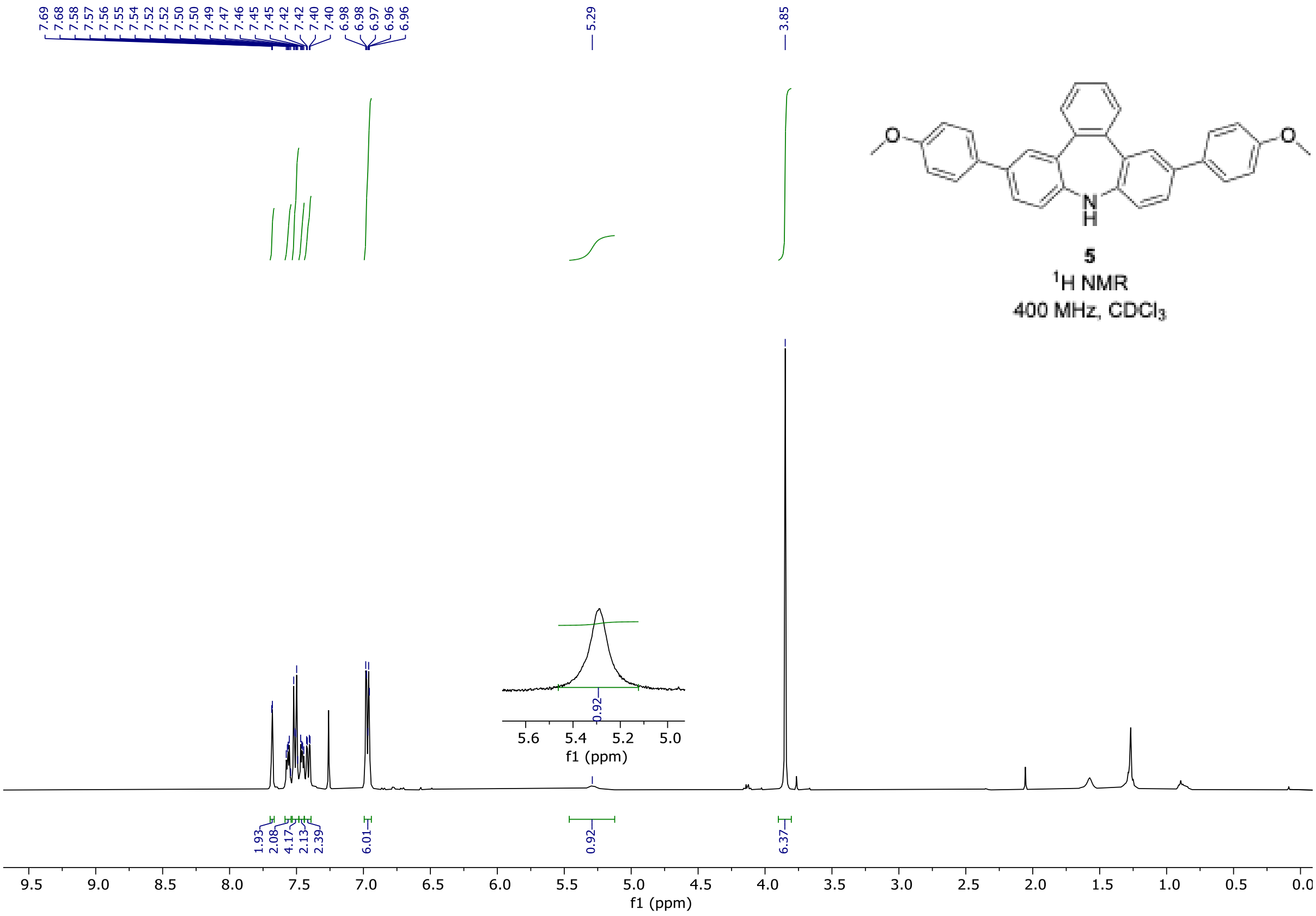
5
^{1}H NMR
400 MHz, $CDCl_3$
f1 (ppm)

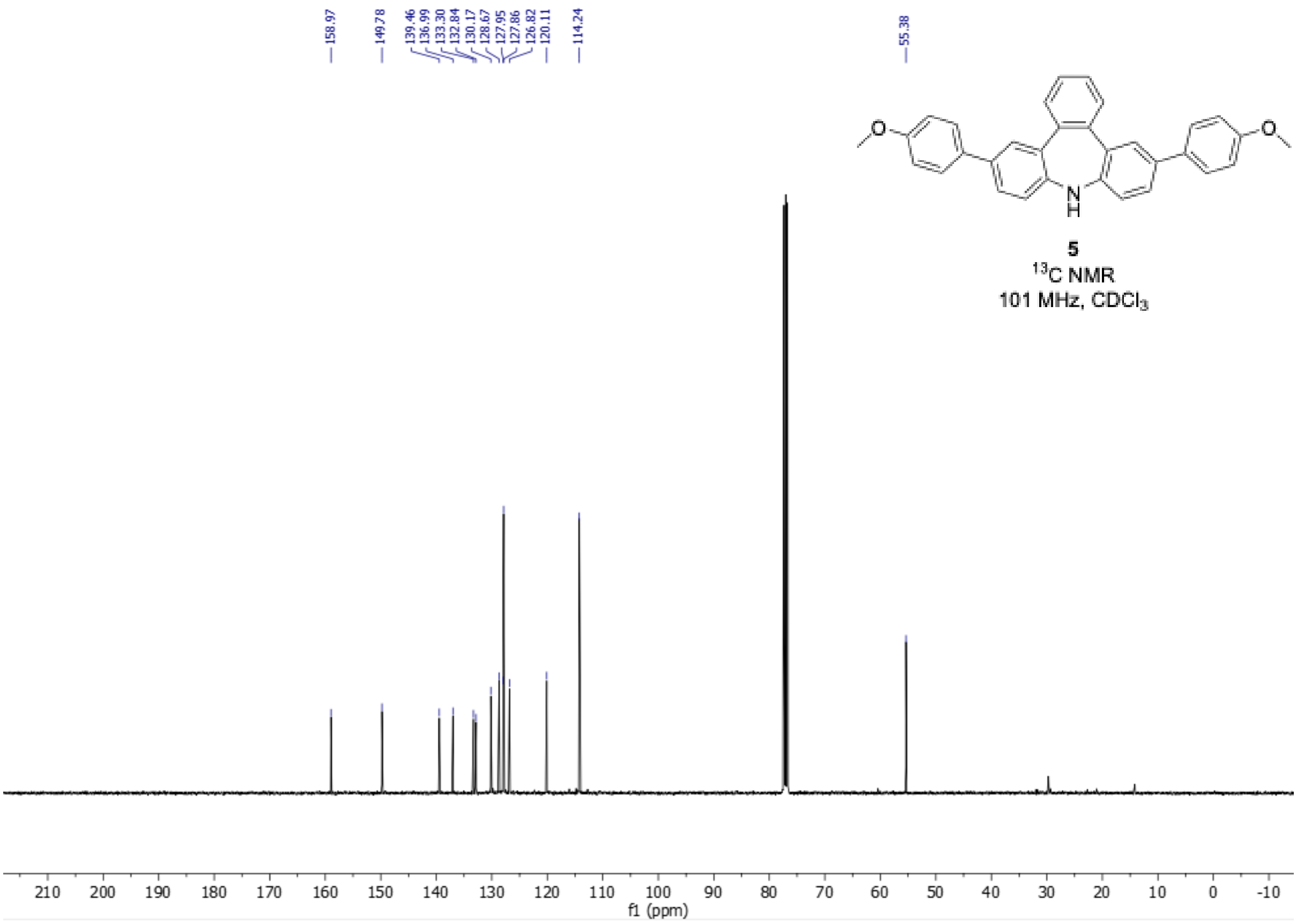
5
^{13}C NMR
101 MHz, $CDCl_3$
f1 (ppm)

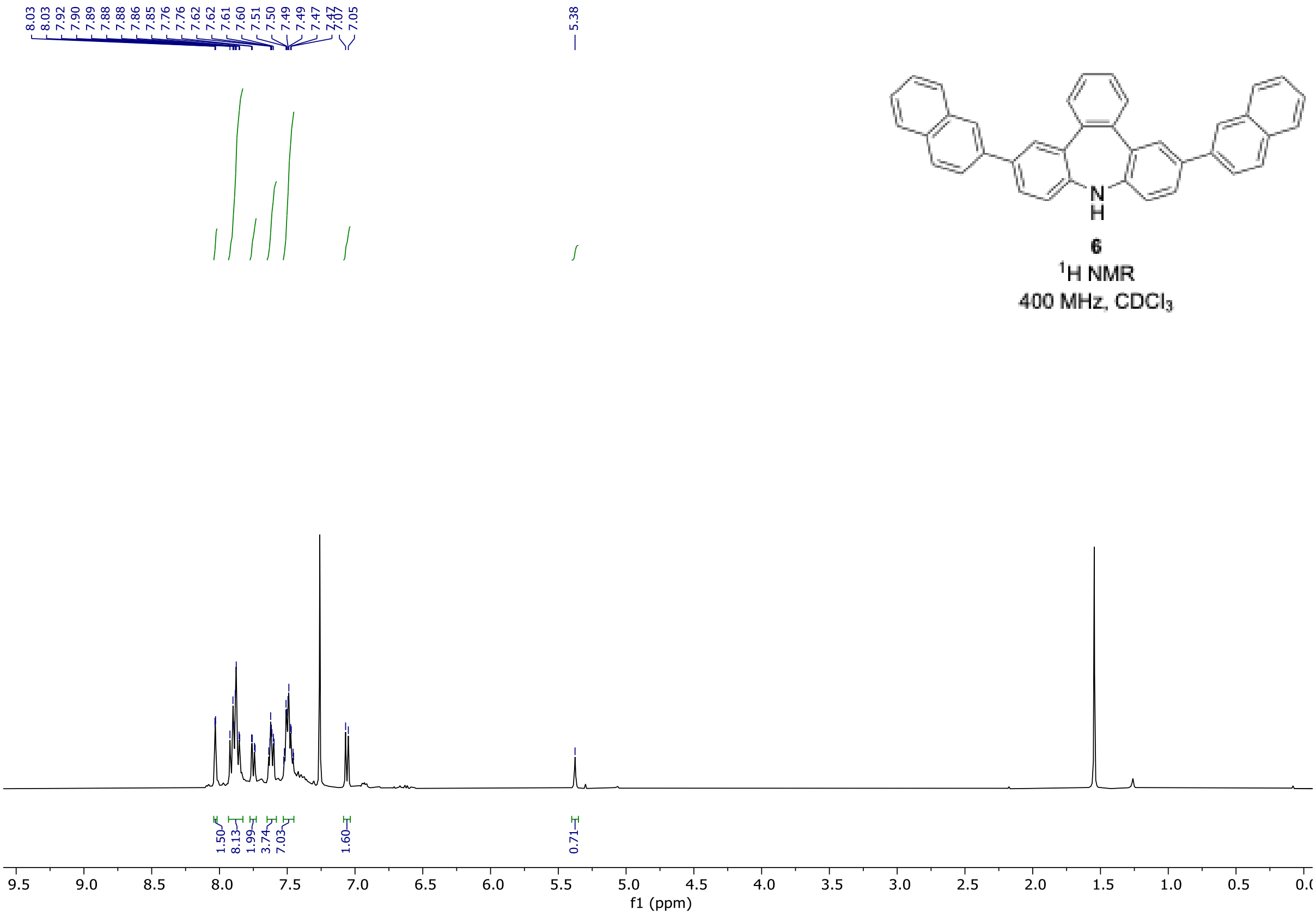
8.03
8.03
7.92
7.90
7.89
7.88
7.88
7.86
7.85
7.76
7.76
7.62
7.62
7.61
7.60
7.51
7.50
7.49
7.49
7.47
7.47
7.07
7.05
5.38
N
H
6
¹H NMR
400 MHz, CDCl₃
1.50
8.13
1.99
3.74
7.03
1.60
0.71
9.5 9.0 8.5 8.0 7.5 7.0 6.5 6.0 5.5 5.0 4.5 4.0 3.5 3.0 2.5 2.0 1.5 1.0 0.5 0.0
f1 (ppm)

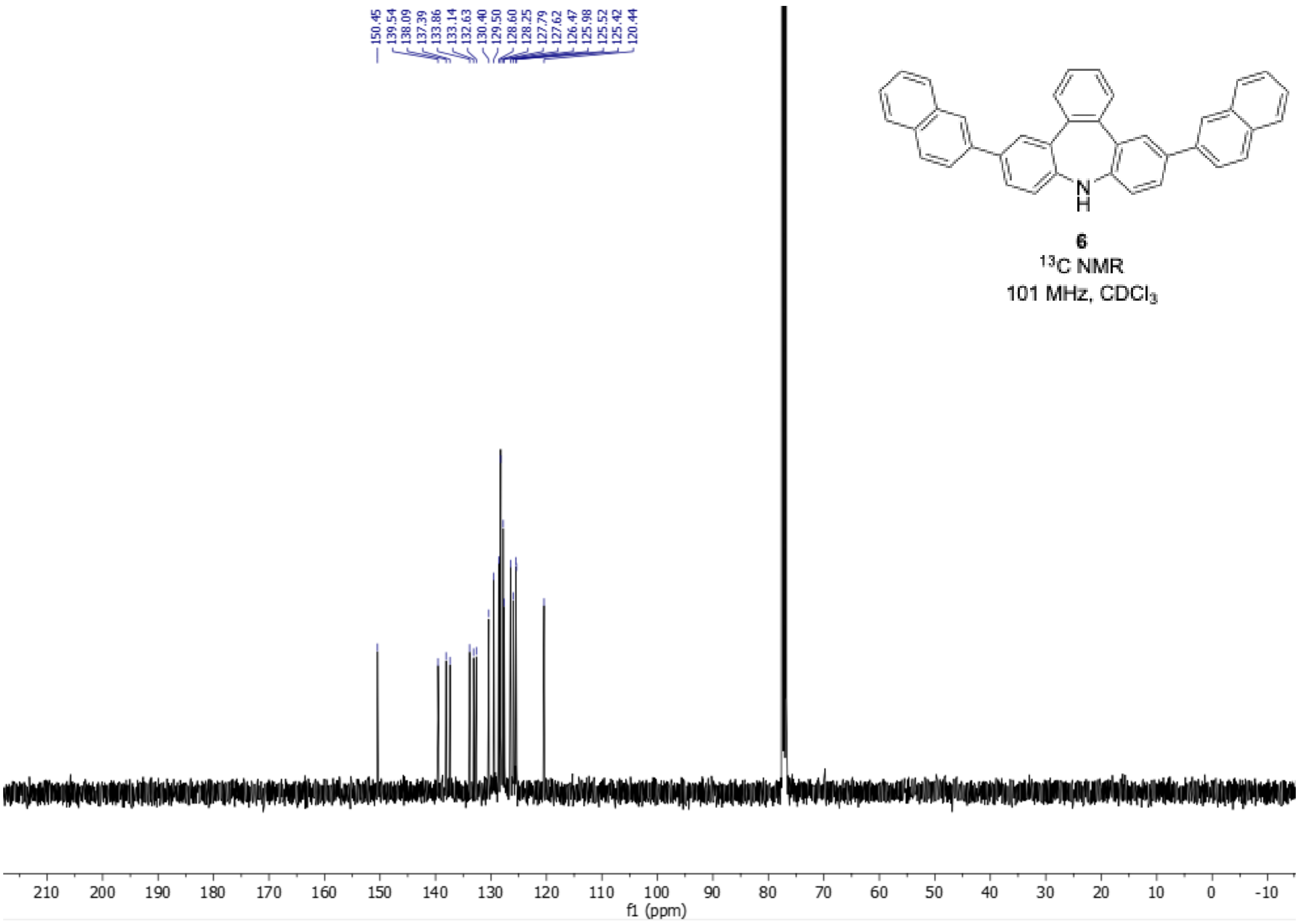
150.45
139.54
138.09
137.39
133.86
133.14
132.63
130.40
129.50
128.60
128.25
127.79
127.62
126.47
125.98
125.52
125.42
120.44
N
H
6
¹³C NMR
101 MHz, CDCl₃
210 200 190 180 170 160 150 140 130 120 110 100 90 80 70 60 50 40 30 20 10 0 -10
f1 (ppm)

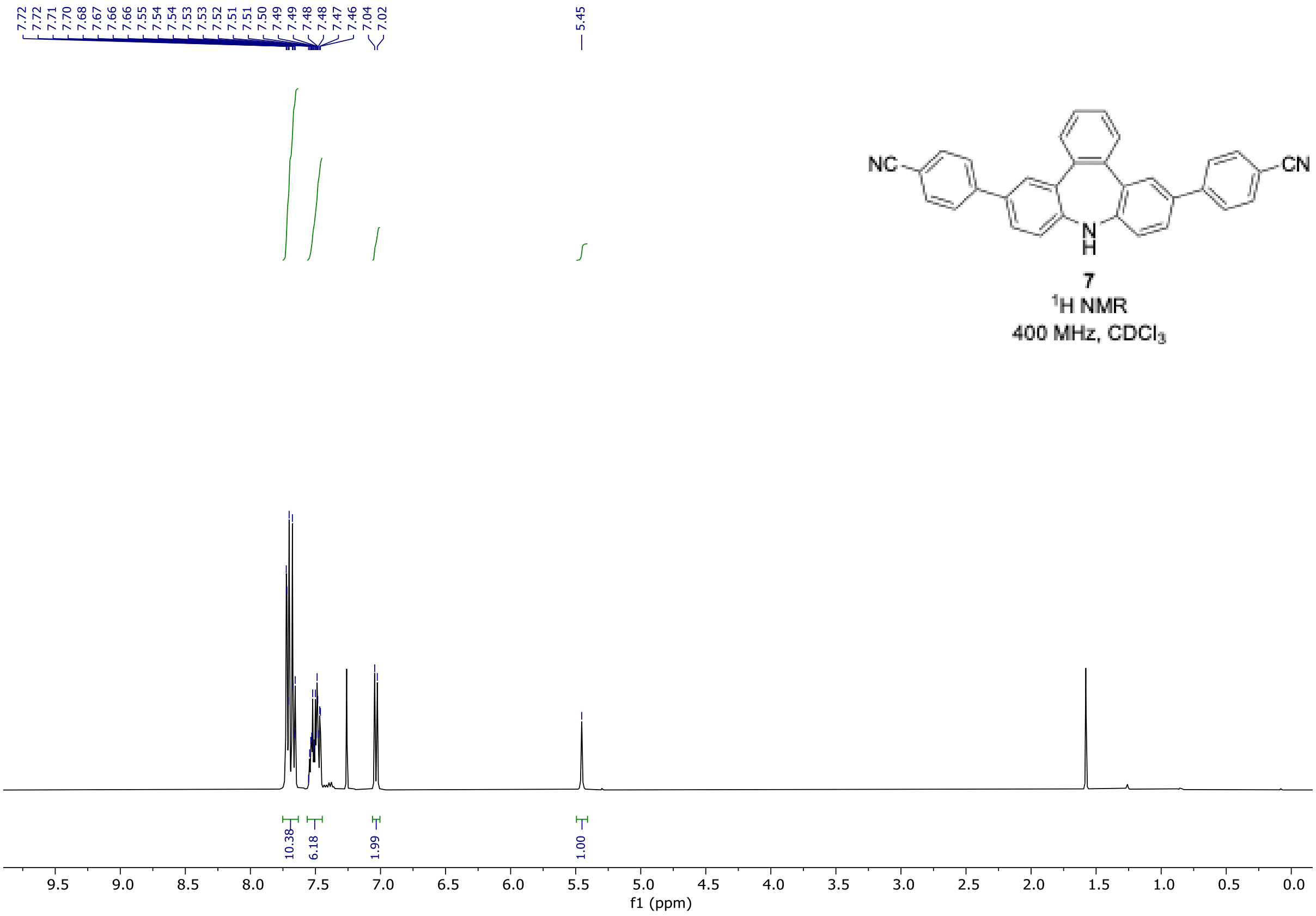

7
^{1}H NMR
400 MHz, $CDCl_3$

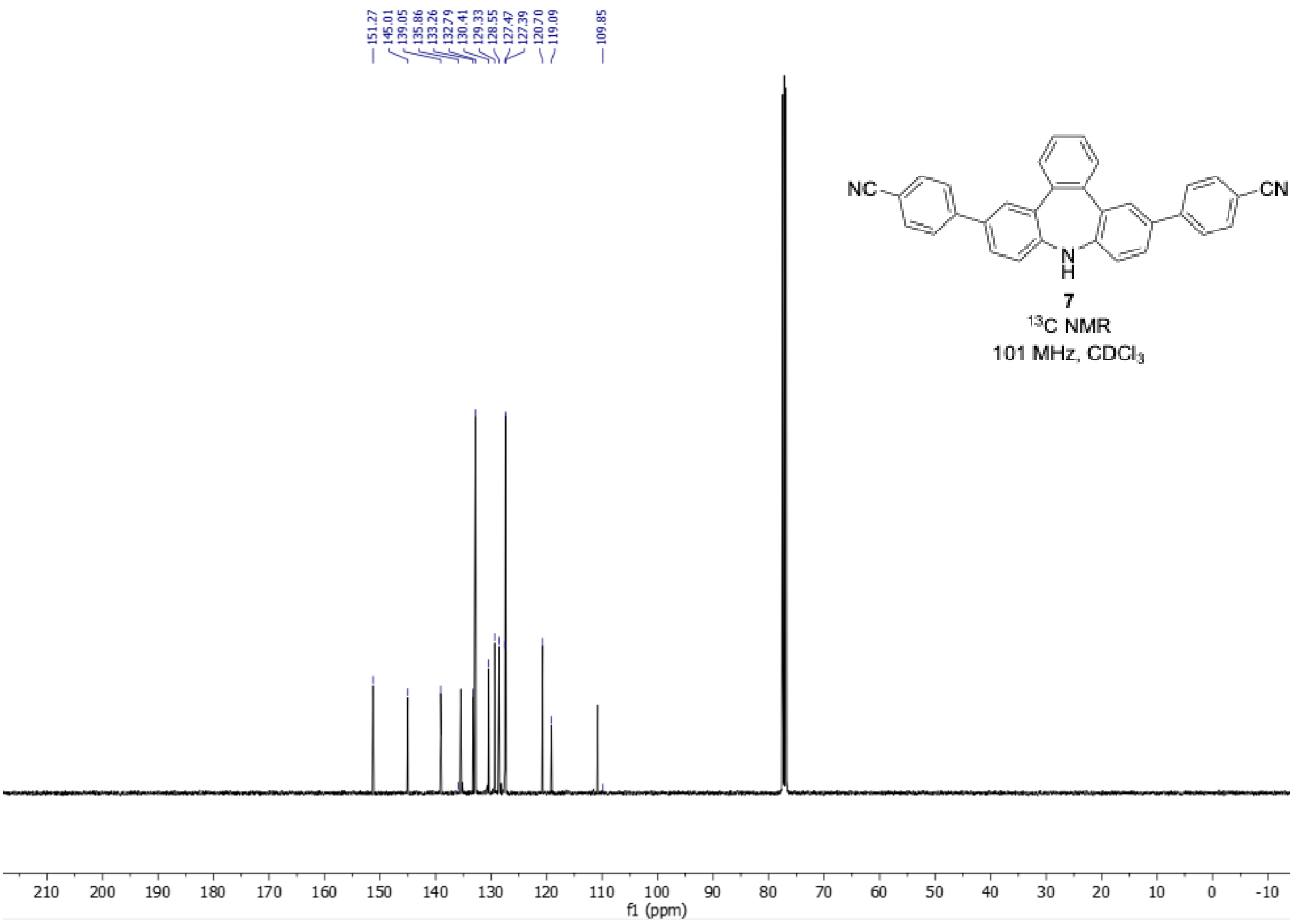

7
^{13}C NMR
101 MHz, $CDCl_3$

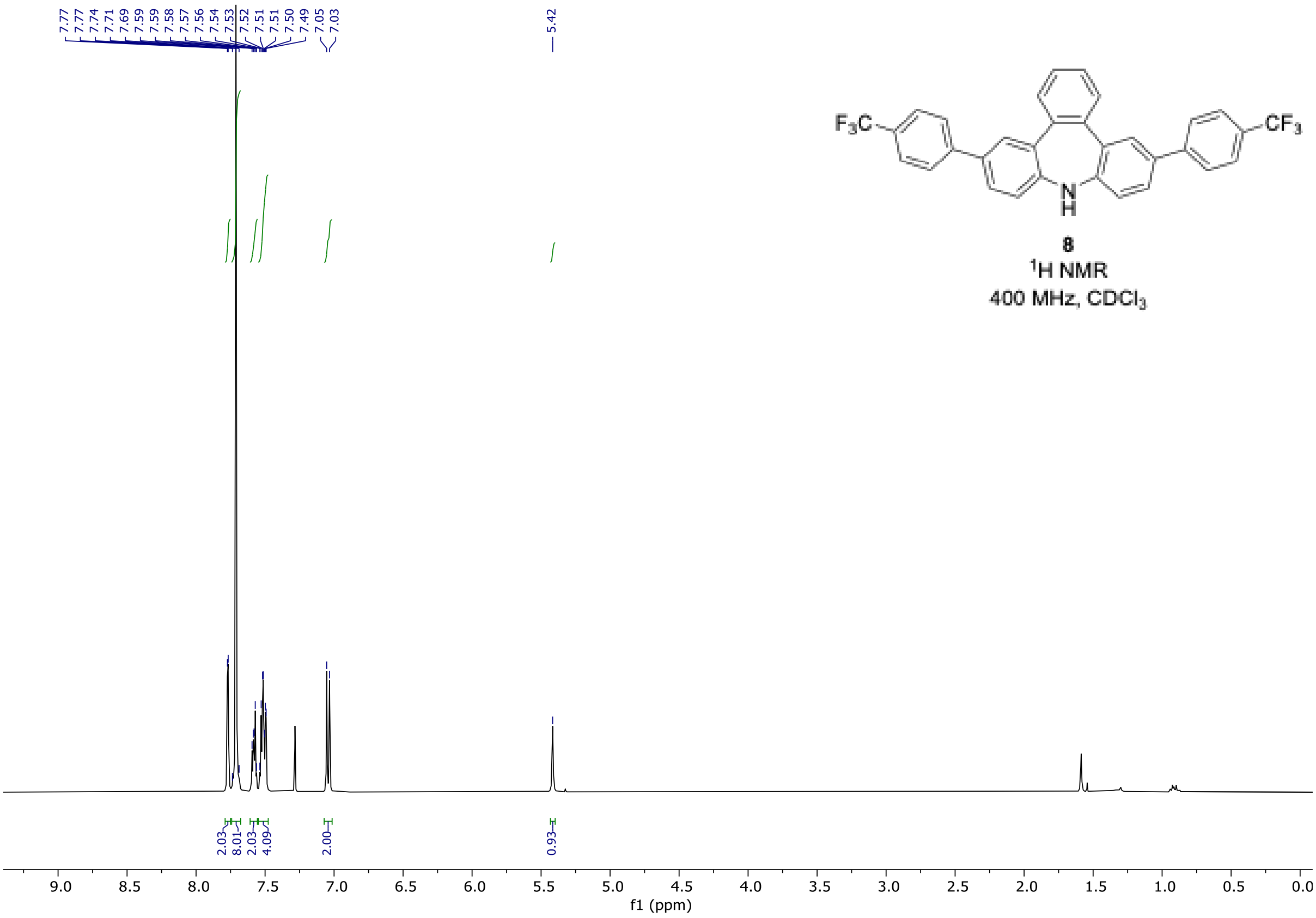
7.77
7.77
7.74
7.71
7.69
7.59
7.59
7.58
7.57
7.56
7.54
7.53
7.52
7.51
7.51
7.50
7.49
7.05
7.03
5.42
F3C
CF3
N
H
8
1H NMR
400 MHz, CDCl3
2.03
8.01
2.03
4.09
2.00
0.93
9.0 8.5 8.0 7.5 7.0 6.5 6.0 5.5 5.0 4.5 4.0 3.5 3.0 2.5 2.0 1.5 1.0 0.5 0.0
f1 (ppm)

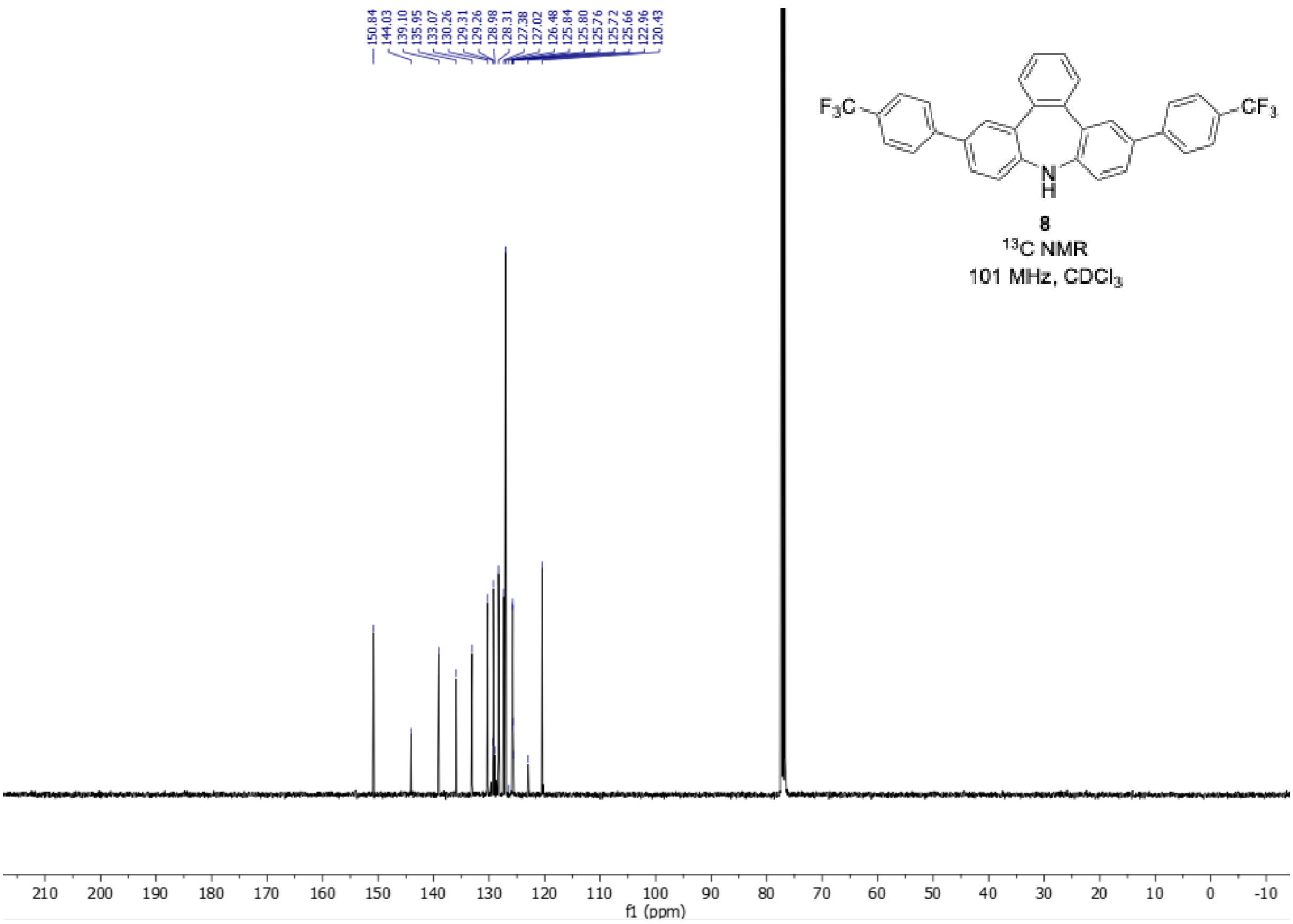
150.84
144.03
139.10
135.95
133.07
130.26
129.31
129.26
128.98
128.31
127.38
127.02
126.48
125.84
125.80
125.76
125.72
125.66
122.96
120.43
F3C
CF3
N
H
8
13C NMR
101 MHz, CDCl3
210 200 190 180 170 160 150 140 130 120 110 100 90 80 70 60 50 40 30 20 10 0 -10
f1 (ppm)

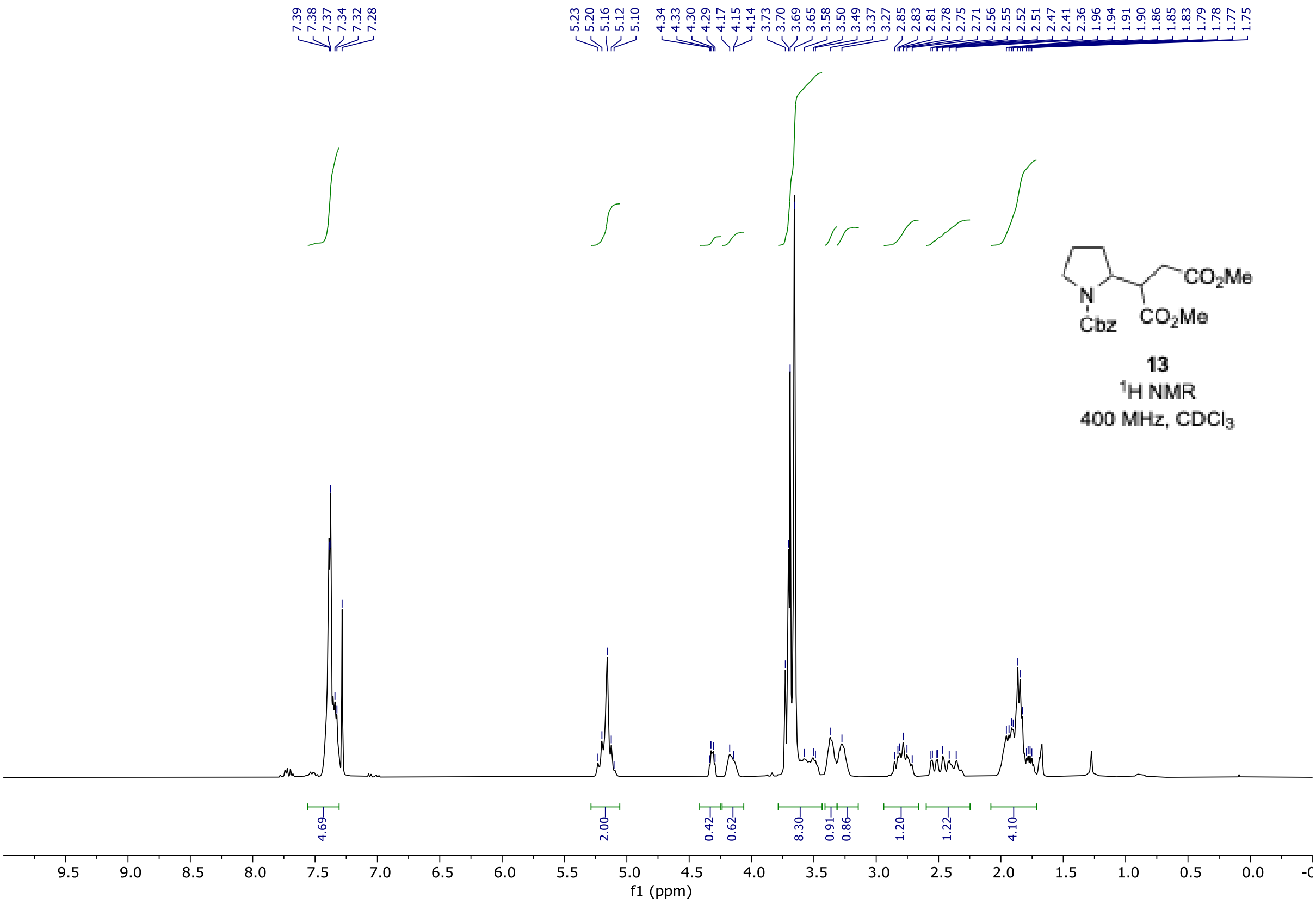
7.39 7.38 7.37 7.34 7.32 7.28
5.23 5.20 5.16 5.12 5.10
4.34 4.33 4.30 4.29 4.17 4.15 4.14 3.73 3.70 3.69 3.65 3.58 3.50 3.49 3.37 3.27 2.85 2.83 2.81 2.78 2.75 2.71 2.56 2.55 2.52 2.51 2.47 2.41 2.36 1.96 1.94 1.91 1.90 1.86 1.85 1.83 1.79 1.78 1.77 1.75
CO2Me
N
Cbz
CO2Me
13
1H NMR
400 MHz, CDCl3
4.69
2.00
0.42
0.62
8.30
0.91
0.86
1.20
1.22
4.10
f1 (ppm)

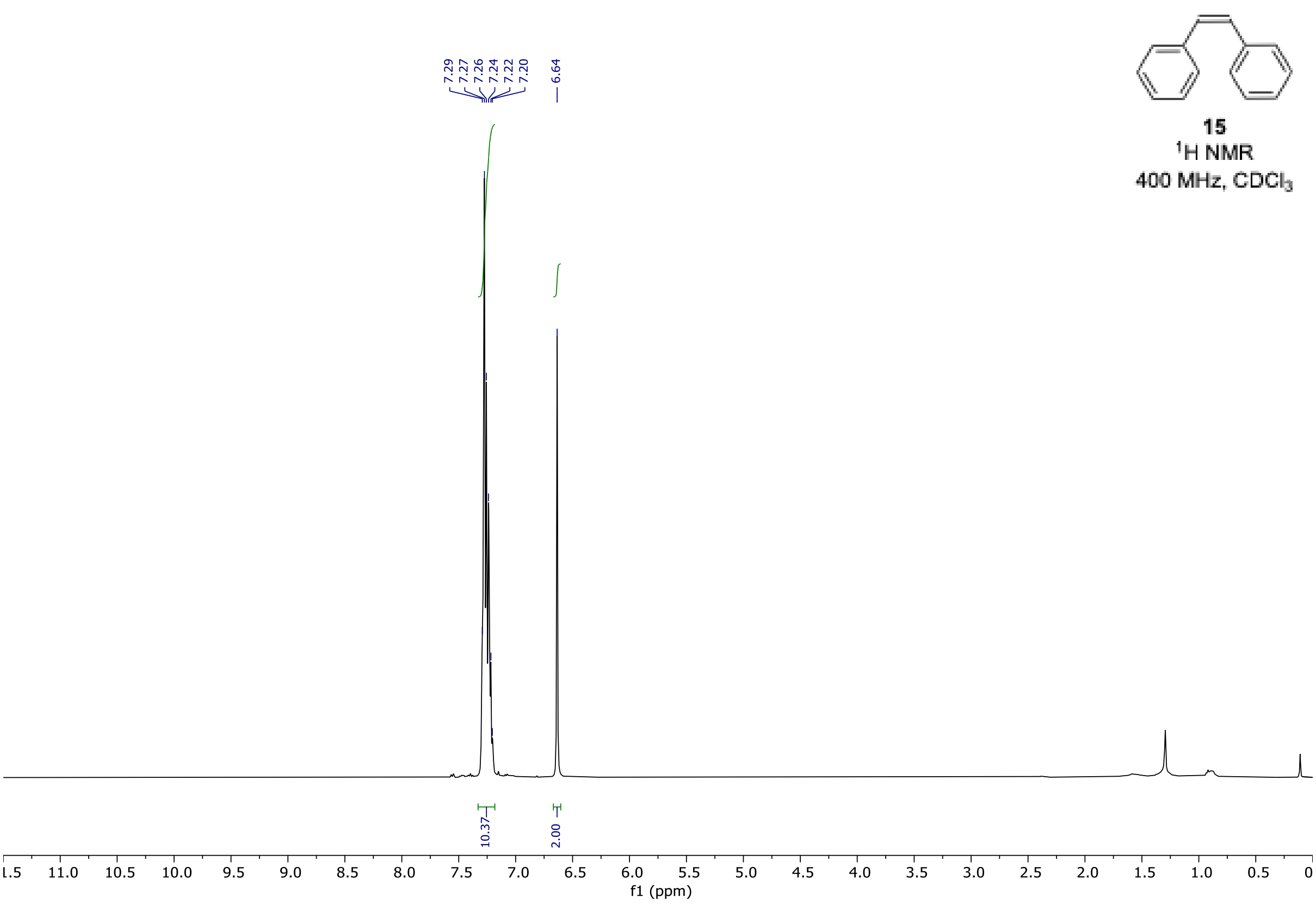
7.29 7.27 7.26 7.24 7.22 7.20
6.64
15
1H NMR
400 MHz, CDCl3
10.37
2.00
f1 (ppm)
